# An annotated genetic map of loblolly pine based on microsatellite and cDNA markers

**DOI:** 10.1186/1471-2156-12-17

**Published:** 2011-01-26

**Authors:** Craig S Echt, Surya Saha, Konstantin V Krutovsky, Kokulapalan Wimalanathan, John E Erpelding, Chun Liang, C Dana Nelson

**Affiliations:** 1 Southern Institute of Forest Genetics, Southern Research Station, USDA Forest Service, Saucier, MS 39574, USA; 2 Department of Plant & Soil Sciences, Mississippi State University, Mississippi State, Mississippi 39762, USA; 3 Current address: Department of Plant Pathology and Plant-Microbe Biology, Cornell University, Ithaca, New York 14853, USA; 4 Department of Ecosystem Science & Management, Texas A&M University, College Station, Texas 77843-2138, USA; 5 Crop Genetics Research Unit, USDA Agricultural Research Service, Stoneville, Mississippi, USA; 6 Department of Botany, Miami University, Oxford, Ohio, 45056, USA

## Abstract

**Background:**

Previous loblolly pine (*Pinus taeda *L.) genetic linkage maps have been based on a variety of DNA polymorphisms, such as AFLPs, RAPDs, RFLPs, and ESTPs, but only a few SSRs (simple sequence repeats), also known as simple tandem repeats or microsatellites, have been mapped in *P. taeda*. The objective of this study was to integrate a large set of SSR markers from a variety of sources and published cDNA markers into a composite *P. taeda *genetic map constructed from two reference mapping pedigrees. A dense genetic map that incorporates SSR loci will benefit complete pine genome sequencing, pine population genetics studies, and pine breeding programs. Careful marker annotation using a variety of references further enhances the utility of the integrated SSR map.

**Results:**

The updated *P. taeda *genetic map, with an estimated genome coverage of 1,515 cM_(Kosambi)_ across 12 linkage groups, incorporated 170 new SSR markers and 290 previously reported SSR, RFLP, and ESTP markers. The average marker interval was 3.1 cM. Of 233 mapped SSR loci, 84 were from cDNA-derived sequences (EST-SSRs) and 149 were from non-transcribed genomic sequences (genomic-SSRs). Of all 311 mapped cDNA-derived markers, 77% were associated with NCBI Pta UniGene clusters, 67% with RefSeq proteins, and 62% with functional Gene Ontology (GO) terms. Duplicate (*i.e.*, redundant accessory) and paralogous markers were tentatively identified by evaluating marker sequences by their UniGene cluster IDs, clone IDs, and relative map positions. The average gene diversity, *H_e_*, among polymorphic SSR loci, including those that were not mapped, was 0.43 for 94 EST-SSRs and 0.72 for 83 genomic-SSRs. The genetic map can be viewed and queried at http://www.conifergdb.org/pinemap.

**Conclusions:**

Many polymorphic and genetically mapped SSR markers are now available for use in *P. taeda *population genetics, studies of adaptive traits, and various germplasm management applications. Annotating mapped genes with UniGene clusters and GO terms allowed assessment of redundant and paralogous EST markers and further improved the quality and utility of the genetic map for *P. taeda*.

## Background

Loblolly pine (*Pinus taeda *L.) is an economically important native forest tree species in the southern United States, accounting for approximately 16% of the harvested industrial wood in the US and 5% worldwide [1,2]. Over 40% of this production occurs on 12 million hectares of sustainably managed forests, where essentially all of the planting stock is the product of genetic improvement [1,3]. In addition to its economic significance, *P. taeda *is ecologically important, as it is a frequent or predominant species on about 25 million hectares of non-planted forested land in the southern US [1]. *Pinus taeda *also is considered one of the major bioenergy feedstocks [4] and recently was selected as the first pine species for complete genome sequencing with the intent of using genome information to enhance its bioenergy potential [5]. Genetic maps, when integrated with physical maps, have played an important role in genome assembly for other plant species [6,7] and, when annotated for gene function, are important for mapping cellular regulatory and signalling networks [8].

Prior *P. taeda *genetic maps were based on multiple types and combinations of marker systems: RFLPs [9], AFLPs [10], RFLPs and RAPDs [11,12], ESTPs [13], ESTPs, RFLPs, and RAPDs [14], SSRs [15], and RFLPs, ESTPs and SNPs [16]. The RFLP, ESTP, and SNP markers in these maps were derived from cDNA sequence, although a few of the RFLP markers also were derived from genomic sequence. All mapping of RFLP and ESTP markers was conducted in one or both *P. taeda *reference mapping pedigrees, *base *and *qtl*. Of all the maps, only the AFLP map of 508 loci [10] and the RFLP-ESTP-SNP map of 373 loci [16] consolidated into 12 linkage groups (LGs) that correspond to the 12 chromosomes of *P. taeda *[17].

Comparative mapping with *P. taeda *using ESTP markers has been conducted with other pines, such as *Pinus elliottii *Englm. [14], *Pinus pinaster *Ait. [18], *Pinus sylvestris *L. [19], and with other conifers, such as *Pseudotsuga menziesii *Mirb. [20] and *Picea *species [21,22]. *Pinus taeda *maps were essential in comparative mapping studies that helped to understand chromosomal evolution and identify syntenic regions in Pinaceae (reviewed in [23,24]). Putative single-copy genes in conserved ortholog sets (COS) have been identified in *P. taeda *and *Picea glauca *(white spruce) [25,26] and segregating markers were developed [26]. As these prior reports point out, the development and mapping of COS markers is expected to be an additional mapping resource that would be particularly useful for more extensive comparative maps and detailed studies of syntenic and phylogenetic relationships between *P. taeda *and other *Pinus *and *Picea *species.

Simple sequence repeat (SSR) markers were integral components of the genetic maps used in prior plant genome projects [6,7] and are expected to be so for the pine genome project. Over 300 SSR markers for *P. taeda *previously were developed from EST sequences [27,28] and genomic DNA sequences [29-31]. While a dedicated SSR mapping study placed 51 genomic-SSR markers into 15 linkage groups [15], only a few SSR loci from any source were integrated into the more complete cDNA-based maps [16,19,32].

Here we describe SSR marker development, mapping, and annotation in *P. taeda*. From SSR-enriched pine genomic DNA libraries and from pine EST databases, we obtained sequences for SSR discovery and primer design. The resulting EST-SSR and genomic-SSR markers were evaluated for specificity and quality of amplification. Those segregating in one or both *P.taeda *mapping pedigrees, *base *and *qtl*, were used for genome mapping. We combined new SSR marker segregation data with prior ESTP and RFLP marker segregation data to construct a new integrated genetic map of *P. taeda*. We assigned UniGene and GO (gene ontology) annotations to all classes of cDNA markers (*e.g.*, EST-SSRs, ESTPs and RFLP). We did so because transcript mapping with UniGene clusters and GO terms can be used to facilitate gene discovery and help integrate positional and functional information for most genes [33], and thus aid future pine genome project efforts. We used the annotations to help identify redundant markers and potentially paralogous loci on the map. The result is the first functionally annotated, SSR-based, genetic map for a conifer. Map and annotation data were loaded into a new database with an enhanced graphic interface, PineMap [34], that was developed to contribute to the goals of the Pine Genome Initiative [35] and provide public access to the map, marker, and associated sequence information on ConiferGDB [36].

## Results and discussion

### Evaluating SSR sequences, primer pairs, and polymorphism

Using 197,931 pine EST sequences that were available July 2004 and represented in 52,911 assembled contigs and singletons, we found 1,485 (2.8%) that contained at least one SSR as defined by our selection criteria (see Methods - SSR selection and primer design). From SSR-enriched genomic DNA libraries, we selected and sequenced 887 SSR-positive clones from *P. taeda *libraries and 619 from *P. radiata *libraries. Marker amplification in *P. taeda *was evaluated for 830 EST-SSR primer pairs, 566 *P. taeda *genomic-SSR primer pairs, and 107 *P. radiata *genomic-SSR primer pairs. In addition, we evaluated 43 previously reported EST-SSRs [27] and 131 previously reported genomic-SSRs [29-31].

Determination of PCR amplification quality and marker polymorphism used different screening and evaluation strategies in different laboratories as each set of primer pairs became available. The final round of evaluation reported here, however, included all previously selected primer pairs, and used one PCR protocol to produce dye-labelled amplicons for allele detection. Following this evaluation, we submitted to NCBI's UniSTS database 165 EST-SSR markers and 203 genomic-SSR markers, as listed in Additional file 1. Detailed marker data for 517 SSR, ESTP and RFLP loci are in Additional file 2.

We genotyped 14 *P. taeda *individuals originating from across the natural geographic range of the species to obtain allele diversity heterozygosity estimates for 185 SSR loci (Additional file 3). This set included eight monomorphic EST-SSR loci that may prove useful for inter-species investigations if subsequent surveys find them to be polymorphic among species. We estimated mean unbiased gene diversity (*H_e_*) as 0.57 for the 177 SSR loci that were polymorphic, 46 of which did not segregate in either mapping pedigree. When examined by SSR class, *H_e_*estimates for 94 polymorphic EST-SSRs and 83 polymorphic genomic-SSRs were 0.43 and 0.72, respectively (difference of *H_e_*means = 0.29, 95% CI = 0.08 - 0.49, *t *= 8.16, *d.f*. = 175, *P *< 0.0000). This demonstrated that *P. taeda *SSRs contained within expressed genes have lower genetic diversity than those contained in anonymous genomic regions presumed to be selectively neutral. Prior studies that used many fewer *P. taeda *SSR markers to examine polymorphism in *P. radiata *or *P. contorta *did not observe this notable difference in gene diversity between transcribed and presumably non-transcribed SSR loci [27,28]. The general constraint on polymorphism that we observed most likely results from background selection or positive selection acting on the associated genes. The observed disparity in the level of polymorphism between the two classes of SSR markers is a clear signature of selection acting on expressed genes as a group [37]. SSRs markers linked with genes of known function and harbouring unusually low levels of polymorphism could thus help to identify candidate genes for adaptively important phenotypes. When considering the general level of polymorphism among all 177 SSR markers, we believe there is sufficient allelic diversity to allow selection of particularly informative sets of markers for use in detailed population genetic studies or tree improvement programs.

### Genetic map

From each pedigree, *base *and *qtl*, we found one progeny sample that had over 40% missing genotype data (Additional file 4, Tables S1 - S4). We excluded these two samples from linkage analyses, leaving 97 *base *samples and 170 *qtl *samples for map construction. Numbers of segregating and mapped loci, categorized by pedigree and marker type, are listed in Table 1. Segregation data for 590 marker loci (Additional file 4, Tables S1 - S4) were evaluated for linkage mapping based on criteria described under Methods - Linkage analysis and mapping. SSR markers NZPR0300, NZPR0440, and PtSIFG_0715 cosegregated on linkage group 5 (LG-5), as did PtRIP_0621 and PtRIP_0165 on LG-10. Because the JOINMAP program automatically retained only one marker from each set for subsequent map construction, we manually reinserted those loci in the final maps after confirming that the marker sequences in each set were not homologous. In total, 154 markers were in common between the two pedigrees to permit map integration, with genomic-SSR markers comprising the largest class (Table 1). Summaries, by pedigree, of the reasons for excluding individual markers from integration mapping are in Additional file 4, Tables S5 and S6.

**Table 1:** Summary counts of *Pinus taeda *marker evaluation and mapping results

Marker type	SSR eval ^1^	***base *seg^2^**	***qtl *seg^2^**	**Total seg^2^**	***base *map^3^**	***qtl *map^3^**	Both map^4^	Rnd 3 integ map^5^	Rnd 2 integ map^6^
genomic SSR	804	138	134	168	121	113	86	149	133
EST SSR	873	72	70	97	61	60	37	84	78
ESTP	n/a	69	104	159	49	65	10	104	98
RFLP	n/a	56	139	166	44	100	21	123	120
total	1677	335	447	590	275	338	154	460	429

n/a - not applicable.

^1 ^SSR primer pairs that were evaluated.

^2 ^Markers that segregated in the *base *pedigree, *qtl *pedigree, and either (Total seg).

^3 ^Markers in the respective pedigree maps used for map integration.

^4 ^Markers common to both *base *and *qtl *pedigrees and used for map integration.

^5 ^Non-redundant marker loci in the round-3 integrated map, which included ancillary markers, as listed in Additional File 2.

**Figure 1:**
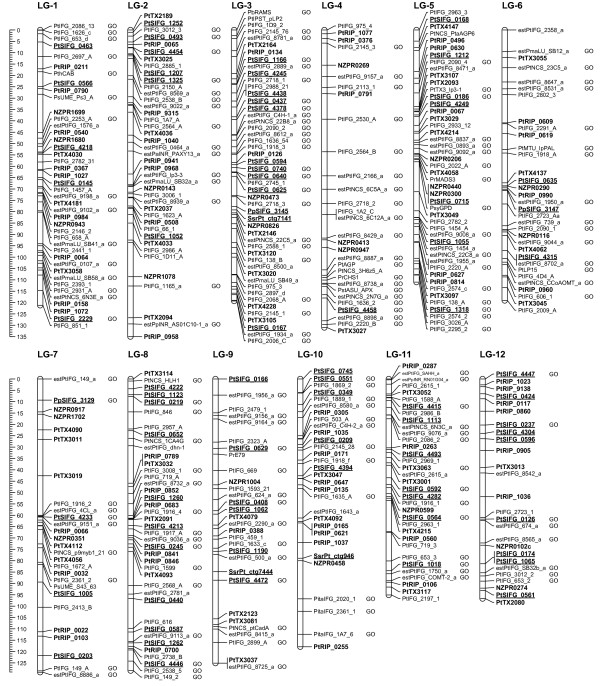
**A genetic map of *Pinus taeda***. The round-2 map of 429 marker loci constructed from the *base *and *qtl *pedigrees. The scales to the left are in cM units, marker names in bold font denote genomic-SSR loci, bold underline denotes EST-SSR loci, and plain font denotes ESTP and RFLP loci. A "GO" tag to the right of a locus denotes assignment of a functional Gene Ontology term. Supporting data are in Additional files 2, 5, 8

^6 ^Non-redundant marker loci in the round-2 integrated map, which excluded ancillary markers, as shown in Figure 1 and listed in Additional file 5.

Based upon the third round (round-3) of JOINMAP mapping calculations for integrated mapping, which included ancillary markers, we retained 460 non-redundant loci in 12 linkage groups that extended 1,416 cM, which provided an average marker interval of 3.1 cM. SSR loci comprised 50% of mapped loci. Approximately equal numbers of SSR primer pairs were evaluated for each marker type (Table 1), however, 53% fewer of the evaluated EST-SSR primer pairs produced mapped markers than did the genomic-SSR primer pairs (18% conversion to mapped loci for genomic-SSR primer pairs vs. 9.6% for EST-SSR primer pairs). We attributed this lower value to the lower genetic diversity observed for transcribed SSR loci, as discussed above.

**Figure 2:**
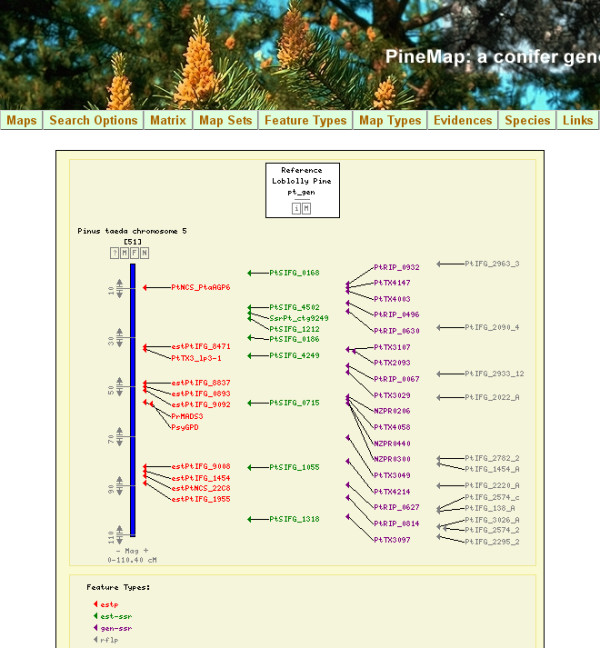
**PineMap user interface**. A web page screen capture of the CMap graphic display for LG-5 of the Map Set Pinus_Taeda_Base_&_QTL_SSR_Version_1 in PineMap [34]. Markers are colour-coded according to marker type. Each marker is hyperlinked to its mapping, annotation, and DNA sequence data. Map, annotation, and sequence data for all markers, and for map metadata, can be downloaded from links available through the Map Sets tab found on the top menu bar

Results from the second round (round-2) of JOINMAP mapping calculations, which exclude poorer fitting ancillary markers, are depicted in Figure 1 with details in Additional file 5. The round-2 map spanned 1,429 cM with 429 loci in 12 linkage groups, providing an average genomic marker interval of 3.3 cM (or 3.6 cM if averaged from individual linkage groups). The numbers of mapped loci in each integrated map, categorized by pedigree and marker type, are in Table 1. Using the more conservatively constructed round-2 map data, we estimated genome length as 1,515 cM using the (*m *+ 1)/(*m *- 1) method. Our round-2 map of 429 loci therefore covers 94% of the estimated genome length and provides a 99.6% probability that any locus in the genome lies within 10 cM of one of the mapped markers (as determined from *c*, defined in Methods - Linkage analysis and mapping). The observed distribution of markers among 10 cM genomic intervals was not different from the expected random distribution (K-S test *D *= 0.25, *P *= 0.786). Interactive graphical displays of these maps, and comparisons between them, can be viewed and queried from the PineMap database (Figure 2) [34]. The round-3 map is represented in PineMap as the map set named Pinus_Taeda_Base_&_QTL_SSR_Version_1, while the round-2 map is in map set Pinus_Taeda_Base_&_QTL_SSR_Version_2.

The prior integrated map of *P. taeda *from the *base *and *qtl *pedigrees contains 302 ESTP, RFLP, and isozyme markers in 12 linkage groups that span 1,274 cM [20], while a recent *qtl *pedigree map of 373 ESTP, RAPD, RFLP, SNP, SSR, and isozyme markers spans 1,228 cM [16]. A previous *base *pedigree map of 51 SSR markers spans 795 cM across 15 linkage groups [15]. Our two-pedigree integrated round-2 map of 429 markers incorporated 227 of the RFLP and ESTP markers and 30 of the SSR markers used in those prior studies. We observed that inclusion of 203 previously unmapped SSR markers extended the mapped *P. taeda *genome by about 150 cM. In particular, SSR loci extended one end of LG-7 by 52 cM and one end LG-12 by 42 cM compared to the prior two-pedigree integrated map of 302 markers [20]. Comparisons of the current and prior integrated maps are shown in Additional file 6. No RFLP or ESTP loci were included in these extended intervals, except for the terminal marker on LG-7, estPtIFG_0149_a. This marker is not present in prior maps, presumably because it had not been linked to other RFLP and ESTP markers [16,17].

Our round-2 map depicted in Figure 1 had overall colinearity with the prior two-pedigree integrated map (Additional file 6). Deviations in the order of loci generally occurred only among closely linked loci. A notable exception was at the end of LG-6, in which three markers in a ~28 cM interval on our map shifted positions relative to three other markers and mapped within a ~8 cM interval on the prior map (Additional file 6). We could not trace the cause of the discrepancy to the mapping process and attributed it to the addition of three SSR markers (PtTX_3055, PtRIP_0609, PtRIP_0619) and the exclusion of an ESTP marker (estPtIFG_8972_a) that altered linkage estimates in the region. Similar types of deviations from colinearity also occur between prior *P. taeda *maps [16,20], as shown in Additional file 7. We suggest that these types of deviations are not specific to our SSR markers or mapping protocols and may be a function of unresolved genotyping errors.

Obtaining consistent linkages and orders of loci were problematic for the distal half of LG-10 in our initial mapping sessions. We resolved the discrepancies by first mapping only the SSR markers in LG-10 using the round-2 JOINMAP map calculations, then adding ESTP and RFLP markers one by one, retaining only those that passed our exclusion criteria (see Methods - Linkage analysis and mapping). Difficulties in ordering RFLP and ESTP markers in this linkage group also arose in prior mapping studies [16,20], as is evident by the flipped orientation of six markers in the distal half of LG-10 (Additional file 7).

### Marker curation

**Figure 3:**
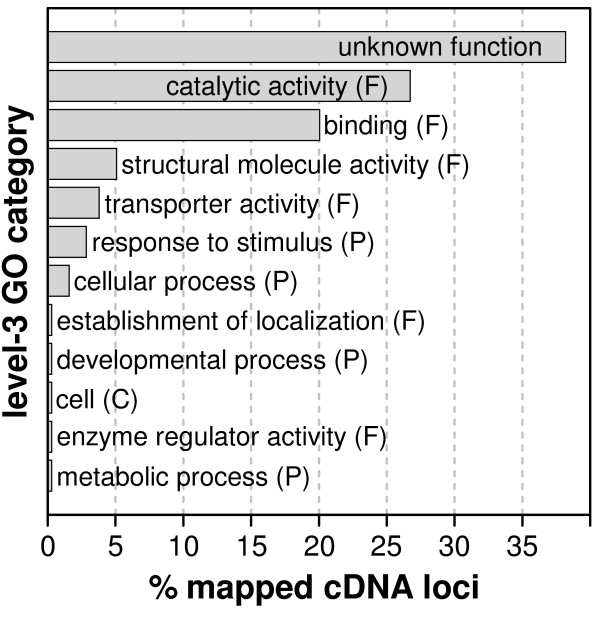
**Comparison of GO categories for mapped cDNA sequences**. Percentages of general GO term category assignments found among mapped cDNA marker loci in *P. taeda*. GO terms for similar functions, processes or cell compartments were grouped by standardizing them to level-3 of the GO lineage hierarchy, where level-1 is 'all' and level-2 comprises the three ontologies: 'molecular_function' (F), 'biological_process' (P) and 'cellular_compartment' (C). Assigned GO terms and the parental GO lineages (levels-3, -4 and -5) for individual markers are in Additional file 8

Each of the 369 cDNA-derived marker sequences was curated, *i.e.*, manually annotated, for a *P. taeda *UniGene cluster, reference protein, and GO term (Additional file 8). We assigned UniGene clusters to 240 of the 311 (77%) mapped cDNA loci and assigned RefSeq proteins to 208 (67%). Based on these assignments GO terms of a known function, process, or cell compartment were assigned to 193 loci (62%). Comparison of the relative proportions of the hierarchical level-3 GO categories for these mapped genes revealed that the categories "catalytic activity" and "binding" together included 46% of all GO term assignments (Figure 3). We flagged in Figure 1 mapped loci that had been assigned functional GO terms, that is, those with a GO term other than the stub assignment of "molecular_function". GO terms and UniGene IDs assigned to mapped markers can be queried as "free text" searches in PineMap [34].

PineMap users also can follow links from a marker in a PineMap or CMap display to its assigned UniGene cluster and associated ConiferGDB EST clusters and contigs [38]. ConiferGDB *P. taeda *EST clusters take advantage of NCBI *P. taeda *UniGene clusters (Build #11). Each NCBI UniGene cluster is intended to include mRNA sequences transcribed from a unique locus in the genome. Because of the particular clustering algorithm adopted by NCBI, however, it is possible that a UniGene cluster contains transcripts from paralogous loci. Conversely, transcript isoforms (alternative splice variants) derived from the same gene could possibly be grouped into different UniGene clusters. ConiferGDB adopts a unique approach to EST clustering by implementing the following protocol: (1) retrieve EST component lists of all UniGene clusters from NCBI, (2) use cleaned EST sequences annotated with cDNA termini, which delimit transcript ends, and (3) conduct EST clustering (ConiferEST clusters) for every individual UniGene cluster using CAP3 to create contig consensus sequences [38,39]. Consequently, one UniGene cluster could contain zero (*i.e.*, no suitable EST sequence are found), one, or multiple ConiferEST clusters and contigs. The result is that for functionally annotated cDNA loci displayed on PineMap, users can quickly obtain information on a marker's UniGene cluster, its ConiferEST cluster, and the consensus sequence of that cluster.

### Redundant markers and paralogous loci

We identified 28 mapped marker pairs that appeared to be derived from the same locus (as defined in Methods - Linkage analysis and mapping) and therefore would have provided redundant mapping information (Table 2). We excluded these markers from the final map integration session, although provide their segregation data to permit independent analysis (Additional file 4, Tables S3 and S4).

**Table 2:** Marker pairs with redundancy for a locus

Redundant marker	GenBank accession	UniSTS ID	Cit^a^	Mapped marker	LG	Evidence^b^
estPtIFG_0066_a	H75018	n/a	[13]	PtIFG_66_1	2	B
estPtIFG_0606_a	n/a	n/a	[13]	PtIFG_606_1	6	B
estPtIFG_1165_a	H75179	n/a	[13]	estPtIFG_1950_a	6	A
estPtIFG_1635_a	H75110	n/a	[13]	PtIFG_1635_A	10	B
estPtIFG_2009_a	n/a	n/a	[13]	PtIFG_2009_A	6	B
estPtIFG_2253_a	H75063	n/a	[13]	PtIFG_2253_A	1	B
estPtIFG_2610E(S)	H75220	n/a	[13]	estPtIFG_1950_a	6	A
estPtIFG_8496_a	AA739536	516058	[14]	estPtIFG_9198_a	1	A
estPtIFG_8596_a	AA739594	516051	[14]	estPtIFG_9092_a	5	A
estPtIFG_8843_a	AA739759	516049	[12]	estPtIFG_9053_a	1	A
estPtIFG_8907_a	AA739797	516254	[13]	PtSIFG_0219	8	A
estPtIFG_AGP-3	n/a	n/a	[12]	SsrPt_ctg7444	9	C
estPtNCS_20G2_a	AI812850	n/a	[12]	estPtIFG_9053_a	1	A
estPtNCS_6C12F_a	AA556811	516381	[14]	PpSIFG_3145	3	C
PtIFG_1576_1	n/a	n/a	[11]	estPtIFG_1576_a	1	B
PtIFG_1635_C	H75110	n/a	[11]	PtIFG_1635_A	10	B
PtIFG_1A2_1	n/a	n/a	[11]	PtIFG_1A2_C	4	B
PtIFG_2145_5	H75056	n/a	[18]	PtIFG_2145_76	3	B
PtIFG_2146_31	n/a	n/a	[11]	PtIFG_2146_2	2	B
PtIFG_2540_2	H75131	n/a	[11]	estPtIFG_0893_a	5	A
PtIFG_2610_A	H75221	n/a	[11]	estPtIFG_1950_a	6	A
PtIFG_2889_21	H75234	n/a	[11]	estPtIFG_2889_a	3	B
PtIFG_2931_b	H75250	n/a	[11]	PtIFG_2931_A	1	B
PtTX3118	AF277845	508481	[40]	PtSIFG_1325	2	C
SsrPt_AA739797	AA739797	516382	[27]	PtSIFG_0219	8	A
SsrPt_ctg4487a	DR159792	n/a	[27]	SsrPt_ctg7141	3	A
SsrPt_ctg4487b	DR159792	516389	[27]	SsrPt_ctg7141	3	A
SsrPt_ctg4698	BG275886	516390	[27]	PtSIFG_1325	2	A

n/a = not available.

^a ^The citation in which the redundant marker was first reported. Additional file 2 lists the citations, GenBank accession numbers, and UniSTS IDs of the mapped markers.

^b ^Evidence for assignment of redundant status: all markers mapped within 3 cM of each other, "A" denotes marker pairs assigned to the same UniGene, "B" denotes marker pairs derived from the same DNA clone, "C" denotes marker pair sequences with a BLASTn E-value less than 4 × 10^-43^.

We found it interesting that the duplicate marker PtTX3118, a SSR marker derived from low-copy, high-*C_o_T*, genomic DNA [40], appeared to be derived from the same gene as PtSIFG_1325, which was an EST-SSR marker derived from a root cDNA library, and which mapped to LG-2. The two clone sequences are nearly identical (99% identity over 188 bp, E-value = 2 × 10^-94^), differing only in the microsatellite region, and putatively code for a Ras-like GTP-binding protein (Additional file 8). Even though a number of the PtTX markers were developed with the intent of targeting gene regions [40], this match was the only instance that we found of a genomic-SSR marker for a transcribed gene.

We identified 21 sets of tentative paralogs that included 58 markers (Table 3). We assigned markers as paralogs if they shared a UniGene ID (or derived from the same DNA clone) and mapped greater than 3 cM apart. In fact, only two markers, found in paralog set #14 of Table 3, approached this distance limit: PtIFG_2574_c and PtIFG_2574_2 were separated by 8 cM near the end of LG-5. The next most closely separated markers assigned as paralogs, PtIFG_1636_3 and PtIFG_1636_54 in paralog set #11, spanned a distance of 13 cM on LG-3. All paralog sets contained markers that mapped to two or more LGs. While most of the paralog sets contained RFLP markers derived from the same clone, which share the clone ID in the marker name, some of the paralogous associations were not so easily identifiable. For example, the following five markers mapped to different LGs, but all were assigned to paralog set #16 and share the same UniGene cluster ID (Pta.598) and GO term (glyceraldehyde-3-phosphate dehydrogenase activity): PsyGPD (LG-5), PtIFG_1165_a (LG-2), PtIFG_2068_A (LG-3), PtIFG_2538_5 (LG-8), estPtIFG_1950_a (LG-6). This further exemplifies the utility of annotating *P. taeda *genetic maps with UniGene and GO assignments to explore the genetic structure of complex gene families that are a characteristic of pine genomes [41].

**Table 3:** Sets of assigned paralogous markers

Marker	Paralog set No.	LG	cM position^a^	GenBank accession	UniGene cluster ID of set members
PitaIFG_2361_1	1	10	102	H75090	Pta.1807
PtIFG_2361_2	1	7	81	H75090	
PitaIFG_1A7_6	2	10	112	AI812330	Pta.2426
PtIFG_1A7_A	2	2	33	AI812330	
PtSIFG_0206	2	1	98	DR160521	
PtIFG_2530_A	3	4	43	H75202	Pta.11556
PtIFG_2588_1	3	3	86	H75218	
PtIFG_653_d	4	1	2	H75097	Pta.1755
PtIFG_653_2	4	12	89	H75097	
PtIFG_653_3	4	11	81	H75097	
PtIFG_3012_2	5	12	87	H75261	Pta.1533
PtIFG_3012_3	5	2	3	H75262	
PtIFG_2393_1	6	1	102	H75095	Pta.18383
estPtIFG_2615_a	6	11	37	CO170994	
PtIFG_149_A	7	7	128	H75140	Pta.1913
PtIFG_149_2	7	8	132	H75141	
estPtIFG_149_a	7	7	0	H75140	
PtIFG_2220_A	8	5	90	DT626617	Pta.19510
PtIFG_2220_B	8	4	124	DT626617	
PtIFG_2145_76	9	3	9	H75056	Pta.2729
PtIFG_2145_1	9	3	115	H75056	
PtIFG_2145_28	9	10	31	H75056	
PtIFG_2145_3	9	4	0	H75056	
PtIFG_1916_1	10	11	48	H75030	Pta.2960
PtIFG_1916_2	10	7	60	H75030	
PtIFG_1916_4	10	8	63	H75030	
PtIFG_1636_2	11	4	111	H75187	Pta.478
PtIFG_1636_3	11	3	68	H75187	
PtIFG_1636_54	11	3	55	H75187	
PthCAB	12	1	19	X13407	Pta.4922
PtIFG_2006_C	12	3	120	H75042	
PtIFG_2718_1	13	3	31	H75230	Pta.4966
PtIFG_2718_2	13	4	78	H75230	
PtIFG_2718_3	13	3	74	H75230	
PtIFG_2574_c	14	5	99	H75217	Pta.575
PtIFG_2574_2	14	5	107	H75216	
estPtIFG_1934_a	14	3	120	DR095068	
PtIFG_2564_A	15	2	33	H75212	Pta.594
PtIFG_2564_B	15	4	52	H75213	
PsyGPD	16	5	57	L26923	Pta.598
PtIFG_1165_a	16	2	112	H75179	
PtIFG_2068_A	16	3	112	H75130	
PtIFG_2538_B	16	2	23	H75204	
PtIFG_2538_5	16	8	131	H75205	
estPtIFG_1950_a	16	6	59	H75126	
PtIFG_1918_A	17	6	42	H75102	Pta.84
PtIFG_1918_f	17	10	37	H75102	
PtIFG_1918_h	17	2	75	H75102	
PtIFG_1918_3	17	3	54	H75111	
PtIFG_975_3	17	3	109	H75119	Pta.9520
PtIFG_975_4	17	4	6	H75119	
PtIFG_138_A	19	5	102	H75134	Pta.11535
PtIFG_138_B	19	3	95	H75135	
PtIFG_2090_1	20	6	77	H75048	Pta.6038
PtIFG_2090_2	20	3	50	H75048	
PtIFG_2090_4	20	5	26	H75048	
PtIFG_2022_A	21	5	54	H75128	Pta.11545
PtIFG_2113_1	21	4	27	H75052	

^a ^Map position in LG from round-3 JOINMAP integrated mapping calculations.

The threshold map distance of 3 cM and other criteria that we used to distinguish redundant markers for a single locus from independent markers for paralogous loci were conservatively chosen to minimize redundant genetic information on our maps. We cannot exclude the possibility, however, that we wrongly categorized some markers as derived from the same gene sequence that were in fact derived from closely linked paralogous genes. Given the available *P. taeda *marker segregation data, we expect that higher density and higher resolution genetic maps, or nearly complete physical maps, will be required to make definitive statements about the fine scale organization of paralogous loci in *P. taeda*.

## Conclusions

We developed 170 SSR markers and mapped 233 in *P. taeda*, many more SSR markers than has been reported for any other conifer species. The integration of these markers with previously mapped ESTP and RFLP markers significantly extended the coverage of two *P. taeda *LGs, LG-7 and LG-12. With the map and marker information that we are providing here, there are now sufficient numbers of different classes of informative SSR markers for immediate use in a variety of areas: *P. taeda *population genetics, evolutionary analysis of candidate adaptive trait genes, and germplasm management applications that require unambiguous identification of parental and clonal genotypes. Through annotation of mapped genes with UniGene cluster IDs and GO terms, we initiated an assessment of redundant and paralogous EST markers, further improving the quality and utility of this and future *P. taeda *genetic maps.

The only two *P. taeda *reference mapping pedigree populations currently available in the public domain (*base *and *qtl*) are limited to 75 and 85 clonally archived full-sib progeny, respectively. The limited mapping resolution provided by this resource is insufficient to provide an extensive genetic mapping scaffold that could assist with physical assembly of the anticipated *P. taeda *genome sequence. For this purpose, a public domain high-resolution genetic mapping resource will be required. Nonetheless, the current map can provide immediate genome ordering of assembled sequence scaffolds by establishing homology between the sequence tagged markers reported here and their corresponding scaffold sequence.

## Methods

### SSR marker development

SSR-enriched genomic DNA libraries of *P. taeda *and *Pinus radiata *were obtained through the service provider GIS (Genetic Information Services, Inc, CA USA). Additional *P. taeda *libraries were obtained by the enrichment protocol of Ostrander *et al. *[42] as described by Echt *et al. *[43]. SSR motifs targeted for enrichment were: AC, AAC, AAG, AAT, ACC, ACG, AGG, ATC, AAAC, AAAT and AGAT. Protocols for DNA sequencing, PCR primer design and amplification, and preliminary evaluations of primer pairs were similar to those previously described [43].

*Pinus taeda *EST sequences and contigs were acquired in July 2004 from the University of Georgia Laboratory of Genomics and Bioinformatics [44]. Additional *P. taeda *ESTs were acquired at the same time from GenBank dbEST. *Pinus pinaster *ESTs were obtained from the INRA, Bordeaux, Laboratory of Forest Genetics and Tree Improvement [45]. From all sources, 197,931 ESTs with 52,911 assembled contigs were used.

Sequences were analyzed with the Gramene SSRIT PERL script SSR.pl [46] to find SSRs that met the following SSR length criteria (bp length); dinucleotides (12), trinucleotides (15), tetranucleotides (16), pentanucleotides (20) and hexanucleotides (24). Only perfect, that is, uninterrupted, SSRs were counted. Thus compound repeats, such as (TC)_8_(TA)_6_, were counted as two repeats, as were imperfect repeats, such as (TA)_21_G(AT)_6_. Parallel analyses were run with the web application BatchPrimer3 [47]. Both analyses gave the same SSR tallies when identical input parameters were used. From the SSR.pl reports, we did not count redundant entries for SSRs that were listed under multiple motif classes. For example, the dinucleotide (AT)*_n_*also would be reported by SSR.pl as a tetranucleotide of (ATAT)*_n_*and as a hexanucleotide of (ATATAT)*_n_*, but we counted all instances as a single dinucleotide SSR. Nomenclature for SSR motifs followed the convention of the alphabetically minimum form [48,49]. For example, all SSRs of the type (AAT)*_n_*, (ATA)*_n_*, (TAA)*n *and their reverse complements (ATT)*_n_*, (TAT)*_n_*, and (TTA)*_n_*were classified as an (AAT)*_n_*motif, or equivalently as AAT.

Primer pairs to identified SSRs were designed from non-redundant sequences using the STS_Pipeline 1.3 package, which is the STS_Pipeline1.2 package [50] that we modified by porting to the Redhat Linux 9.0 operating system and upgrading the primer design engine to Primer3. Similar SSR primer analysis functions are available on-line with BatchPrimer3 [47]. Input parameters used for primer design included minimum primer size = 18 nt, maximum primer size = 24 nt, optimal size = 20 nt, minimum GC content = 20%, maximum GC content = 80%, minimum Tm = 56° C, maximum Tm = 64° C, optimal Tm = 60° C, 3' end complementarities = 3 nt, any complementarities = 8 nt, minimum amplicon size = 100 bp, and maximum amplicon size = 500 bp.

For each sequence, three primer pairs having the three best PRIMER_PAIR_QUALITY tag values were reported. The STS_Pipeline reports were screened with custom PERL scripts to select the one primer pair per sequence that flanked the intended SSR target and had an optimal balance of primer quality score and amplicon size. For example, when considering dinucleotide motif SSRs, the primer pair with the best quality score was selected if it had an amplicon size from 100 bp to 299 bp, though if it did not, then the pair with the shortest amplicon greater than 299 bp was selected. Similar selection criteria were applied to the longer SSR motifs, except that the preference was for the primer pair with the best quality score and with an amplicon size greater than 299 bp. If those two criteria were not met, then the pair that produced the longest amplicon size less than 299 bp was selected. We used this size distributed selection strategy to increase the number of potential marker loci that could be grouped in multiplexed genotyping assays.

### Marker evaluation and genotyping

The resulting PCR primer pairs were evaluated for amplicon marker quality with DNA samples obtained from 14 unrelated *P. taeda *cloned individuals originating from across the natural geographic range, including the four parents of our two reference mapping pedigrees. The geographic provenance of each population sample is listed in Additional file 3. We selected markers for subsequent linkage mapping and population genetic analysis if they consistently amplified what appeared to be single loci in the expected amplicon size ranges. Gene diversity and related allele frequency statistics for the population samples were obtained using the software package GENALEX [51].

We detected and scored SSR alleles by sizing PCR amplicons with capillary electrophoresis. All PCR forward primer oligonucleotides were tailed on their 5' end with the M13forward(-29) sequence CACGACGTTGTAAAACGAC. All reverse primer oligonucleotides were tailed on their 5' end with GTTTCTT, forcing a non-templated dA addition to the amplicons [52]. Fluorescent dye (6-FAM, VIC, NED or PET) was incorporated in amplicons by including a 5' dye-labelled M13 forward (-29) primer in the PCR [53]. PCR composition in a 6 μl reaction volume was as follows: 10 ng pine DNA template, 40 nM forward primer, 160 nM reverse primer, 160 nM dye-labelled primer, 66 μM dNTPs, buffer (1.5 mM MgCl2, 10 mM Tris-Cl, 50 mM KCl, 0.1% Triton X-100, pH 9.0) and approximately 1 Unit *Taq *polymerase mixed with anti-*Taq *polymerase antibody. Equivalent results were obtained by substituting a suitable hot-start *Taq *polymerase and omitting the antibody. We used a hot-start PCR thermocycling protocol: 2 min at 94 °C; followed by 20 cycles of 30 s at 94 °C, 30 s at *x *°C, and 30 s at 72 °C, where *x *= 65 °C - 0.5 °C per cycle; followed by 15 cycles of 30 s at 92 °C, 30 s at 55 °C, 1 min at 72 °C; followed by 15 min at 72 °C. Completed PCR reactions were refrigerated until analyzed. PCR amplicons and ABI PRISM GS 600 LIZ internal size standards (Life Technologies Corporation, Carlsbad, CA, USA) were separated by capillary electrophoresis using ABI PRISM 3100 or 3130xl Genetic Analyzers (Life Technologies Corporation, Carlsbad, CA, USA). Markers were multiplexed four to a capillary channel, one of each different dye label, such that marker allele size ranges did not overlap. We used this conservative multiplex strategy to avoid allele detection complications that can arise when occasional instances of excessive concentrations of a marker yield spectral overlap ("bleed-through") with similarly size markers of different dye labels. Allele sizing by the third order least-squares algorithm and allele size binning were performed with ABI PRISM GENEMAPPER 3.7 software (Life Technologies Corporation, Carlsbad, CA, USA), followed by manual inspection and editing of the automated allele assignments as needed. A second person independently verified all allele assignments. We standardized SSR marker allele bins among capillary electrophoresis runs with the aid of control genotype samples from the *P. taeda *clones 7-56 and B-145-L Control genotypes for all SSR markers are listed in Additional file 4, Table S7.

Non-SSR marker segregation data for *base *and *qtl *pedigrees were provided by G. Brown [12,11]. We converted non-SSR marker data from a numerical genotype code format to an ABCDMN format prior to analysis. The PERL script genojoin.pl http://www.esd.ornl.gov/PGG/scripts.htm was used to properly format the converted genotype data to a format suitable for linkage analysis JOINMAP (see Linkage analysis and mapping section below). SSR genotypes in exported GENEMAPPER tables were analyzed for coding errors and converted to an ABCDMN format by the PERL script genomapper.pl http://www.esd.ornl.gov/PGG/scripts.htm. The corrected and reformatted SSR genotype tables subsequently were converted to a JOINMAP format using the genojoin.pl script.

### Marker nomenclature

SSR markers developed in the course of this study were given the prefix "PtSIFG" if they were derived from *P. taeda *ESTs, "PpSIFG" if from *P. pinaster *ESTs, "PtRIP" if from *P. taeda *genomic libraries, and "NZPR" if from *P. radiata *genomic libraries. RFLP and ESTP markers were named as previously published [20,54], as were existing microsatellite markers of the SsrPt and PtTX series [27,26]. Known aliases are listed in Additional file 2.

### Mapping populations

Mapping populations were from two unrelated *P. taeda *outbred pedigrees constructed by the Weyerhaeuser Company, referred to as *base *and *qtl*[11]. For the *base *pedigree (cross: 20-1010 × 11-1060, described in [9]), we mapped markers that had been genotyped in two sets of full-sib progeny: one set of 95 progeny that prior studies used for mapping RFLP and ESTP markers [9,14] and one set of 75 progeny that we used to genotype SSR markers. There were 72 progeny in common between these sets, providing 98 *base *progeny samples for mapping all markers. We genotyped six PtTX SSR markers in both progeny sets and used the consolidated segregation data for verifying sample identities between the sets and for mapping. For the *qtl *pedigree (cross: 6-1031 × 8-1070), we mapped markers that had been genotyped in four sets of full-sib progeny: the full set of 172 progeny and a subset of 48 progeny that prior studies used for mapping RFLP markers [11,55], a subset of 95 that prior studies used for mapping ESTP markers [13,14], and a subset of 85 progeny that we used to genotype SSR markers. We genotyped eleven PtTX and eight NZPR SSR markers in the latter two progeny sets and used the consolidated segregation data for verifying sample identities between the sets and for mapping. From scion material that we obtained from Weyerhaeuser Company, we established a clone archive of the *base *(*n *= 75) and *qtl *(*n *= 85) progeny sets used for SSR genotyping, which we named *Base2 *and *Qtl2*, respectively. Needle or DNA samples from these trees can be obtained from author CDN.

By comparing the multi-locus SSR genotypes of each sample that was in common between the overlapping progeny sets of each pedigree, we found and subsequently resolved progeny sample code discrepancies between the archival data and our newly generated data. For each mapping pedigree we then merged the correctly aligned genotype segregation matrices from the various progeny sets into an interleaved matrix that we used for subsequent linkage analyses and mapping (Additional file 4, Tables S1 - S4).

### Linkage analysis and mapping

We performed linkage analyses and consensus map integration using JOINMAP 3.0 [56,57]. We established the 12 linkage groups (LGs) reported in published *P. taeda *maps by inspecting LOD grouping of markers constructed at 0.5 LOD intervals from LOD 3 to LOD 7. The maximum recombination parameter for establishing linkage was set to 0.5, which imposed no restrictions on the LOD groupings. LG number identifiers were assigned based on previously reported marker locations on individual *P. taeda *linkage groups [14]. The JOINMAP mating type parameter CP (cross-pollination) was used for the allogamous outcrosses of both mapping pedigrees. Mapping parameters were set for a "jump" threshold of 5, a ripple value of 1, and the Kosambi mapping function. JOINMAP uses a reiterative process through three rounds of mapping calculations. During the first two rounds, JOINMAP excludes markers that exceed the assigned jump threshold (the normalized difference in the goodness-of-fit values for a marker ordered in a stepwise process), while during the third round they are placed into their most likely positions in the LG regardless of the jump threshold [57]. We retained markers placed on the round-3 maps for subsequent mapping sessions, except as noted below for the final integrated mapping session from which we report results for the round-2 and round-3 integrated maps.

We first constructed maps separately for the *base *and *qtl *pedigrees to assess the quality of the segregation data for individual markers and identify unsuitable or redundant markers. We excluded loci if they segregated null alleles, if they had excessive segregation distortion (*P *< 0.005 for *χ^2^*tests), if they altered locus orders, or if they appeared to be a redundant marker for a locus. We did not employ a Bonferroni adjustment of *P *for tests of segregation distortion because we empirically determined that markers within the adopted limit of *P *could be mapped with confidence if they had not been excluded by other criteria. We assigned two markers as redundant if they mapped within 3 cM of each other and either derived from the same cDNA clone, had the same UniGene ID, or had at least a 99% BLASTn sequence identity over more than 185 bp. The latter criterion was applied only in instances where one marker of the pair was derived from a pine EST sequence other than *P. taeda *or from a non-EST (genomic) sequence. The 3 cM distance limit was determined empirically from distances that we observed between non-cosegregating map positions of markers known to be for the same locus. This included SSR markers independently genotyped with the same primers in different labs or different mapping progeny subsets and RFLP and ESTP markers derived from the same gene. Adoption of this limit was supported by a report that ESTP, RFLP, SNP, and isozyme markers for the same single copy *cad *(cinnamyl alcohol dehydrogenase) gene mapped within 2.4 cM of one another in *P. taeda *[16]. Further, simulations show that a 3% genotype error rate can nearly double the map distance between loci separated by 2 cM and obscure their correct map order [58]. We therefore assumed that there may be some degree of undetected genotyping errors for marker loci that otherwise would be expected to cosegregate and we assigned such loci a redundant status if we found evidence that they covered the same gene and mapped less than 3 cM apart. The one marker of a redundant pair that we excluded was the one with the poorer goodness-of-fit, that is, the one with the larger *χ^2^*contribution to the ordered group. We repeated mapping sessions to assess marker suitability until all unsuitable markers were excluded.

We did not exclude markers based on missing data except for extreme instances of markers with >70% missing data, which we found only in the *qtl *pedigree segregation data. Markers with lower percentages of missing data did not warrant *a priori *exclusion because they were rejected by our other exclusion criteria during the reiterative mapping sessions. We were aware of potential negative effects on map order and distances that can arise from missing data [58], however, we used the efficiency of the mapping process to include markers that could be placed accurately and exclude those that could not. The rationale of this approach was to map SSR markers in the context of prior cDNA-based *P. taeda *maps by incorporating the same ESTP and RFLP marker segregation data used to construct those maps [14,16,20]. Because sets of data were consolidated from different mapping population cohorts, described above in the Mapping population section, the analysed matrix of segregating genotypes contained blocks of missing genotypes (*i.e.*, ranging from 1% to 40% for RFLP markers and 45% to 67% for ESTP markers in the 172-sample *qtl *mapping population). We therefore replicated in our mapping analyses the same levels of missing data for ESTP and RFLP loci that prior studies [14,16,20] used to construct *P. taeda *maps.

Markers retained from the individual pedigree mapping sessions were used for integrating the pedigree maps by applying the JOINMAP function 'Combine Groups for Map Integration' to individual linkage groups. We used an initial session of map integration to identify and subsequently exclude markers that caused inconsistency in the order of groups of loci, expanded the LG length by more than 5 cM, or appeared to be a redundant marker for a locus. Retained markers were used in a final session of map integration. Markers added during the round-3 JOINMAP map calculations are considered ancillary markers. Details of round-3 mapping data are listed in Additional file 2 and used for reporting summary map statistics, while round-2 map data are listed in Additional File 5 and depicted in Figure 1. We charted linkage maps using the MAPCHART v2.1 program [59].

We obtained an estimated genome length by summing adjusted map lengths for the 12 linkage groups according to method 4 of Chakravarti *et al. *[60]. In that method the length of each linkage group is adjusted by multiplying the observed cM length by the factor (*m *+ 1)/(*m *- 1), where *m *is the number of markers in the linkage group. We used this method because, when evaluating larger numbers of markers and segregating progeny, it performs better than an alternative maximum likelihood method based on pair-wise recombination values or a method-of-moments estimator based on pair-wise LOD scores [60]. Genome coverage was estimated as the proportion of marker coverage *c, *given that at least one marker is located within a specified mapping interval *d *(that is, adjacent markers should be at most 2*d *apart) in a genome of length *L *with *m *mapped markers, such that *c *= 1 - *e*^-2*dm*/*L *^[10,61]. These estimates for genome length and genome coverage assume a random distribution of mapped markers; therefore, we tested for random marker distribution in the integrated map. We parsed the concatenated map into 10 cM intervals and counted the number of intervals that contained an *x *number of markers, for *x *from zero to 11. To compare these results to a random distribution, we used a Poisson distribution function, *P(x) = μ*^*x*^*e*^*-μ*^*/x!*, where *P(x) *was the probability of *x *number of markers per interval and *μ *was the average number of markers per interval in the integrated map. We obtained the distribution of expected number of intervals containing *x *markers by multiplying *P(x) *by the total number of 10 cM intervals on the integrated map for each corresponding value of *x*, from zero to 11. We then used the Kolmogorov-Smirnov (K-S) test for two populations to determine whether the distribution of observed counts was likely the same as the distribution of expected random counts [62].

### Marker curation

We searched NCBI's UniGene database [63] for each marker's GenBank accession number and when a match was found we assigned the Pta (*P. taeda*) UniGene cluster ID to the marker (Additional file 8). If a marker's sequence was from a conifer species other than *P. taeda*, or was a *P. taeda *transcribed sequence not found in a Pta UniGene cluster, then we used it as the query in a BLASTn search to find in GenBank a homologous *P. taeda *EST target. We conducted searches with the NCBI BLAST engine http://blast.ncbi.nlm.nih.gov/Blast.cgi and selected target sequences that had the highest sequence identity alignment above 85% and spanned at least 220 nucleotides of the query sequence. If a selected target sequence was also a member of a Pta UniGene cluster, then that cluster ID was assigned to the marker. In the few cases of ESTP markers that had no available cDNA or EST sequence, we used the marker's primer pair sequences in a BLASTn search of GenBank's *P. taeda *ESTs. If both primers aligned exactly and in the expected orientation, then the accession number of the target EST was assigned to the marker and UniGene cluster assignment proceeded as described. Summary statistics of BLASTn alignments for individual markers are in the Notes column of Additional file 8.

We assigned RefSeq proteins [64] to markers as the main means to obtain GO term annotations. Using the RefSeq protein information provided by NCBI for each *P. taeda *(Pta) UniGene cluster, we assigned to the marker the RefSeq that had the highest reported amino acid identity and a functional GO term annotation (Additional file 8). Generally, *Arabidopsis thaliana *or *Oryza sativa *RefSeq proteins were selected. If no RefSeq proteins were listed for a UniGene cluster, but a functionally annotated *Pinus *mRNA (complete or partial cds) was in the cluster, then that mRNA's GenBank protein was assigned as the reference protein (8 cases). If we could not assign a UniGene cluster to a marker, then we based reference protein assignment on the results of a BLASTx search of the RefSeq protein database with the marker's EST sequence (16 cases). All reference protein assignments were contingent on a greater than 45% amino acid sequence identity that spanned more than 45% of the marker's translated amino acid sequence length, as reported by the NCBI BLAST search engine http://blast.ncbi.nlm.nih.gov/Blast.cgi. Details of assignments based on these later conditions are in the Notes column of Additional file 8.

For most markers, we assigned GO terms [65] based on the assigned RefSeq protein's GO annotation available either from NCBI records or from The Arabidopsis Information Resource http://www.arabidopsis.org/. If a marker's assigned reference protein was a *Pinus *GenBank protein (that is, not a RefSeq protein), then we adopted the GO term of the most homologous and descriptively annotated RefSeq protein that was listed for the marker's UniGene cluster (5 cases). If no RefSeq was listed, then we assigned a marker's GO term *de novo *from the AmiGO database [66] by searching on the functional annotation of the marker's assigned *Pinus *reference protein (3 cases). We assigned to a marker the "molecular_function" GO term as a stub if its reference protein had an unknown function or if it had neither a reference protein nor a UniGene ID assignment.

GO terms were obtained from the GO database version of October 2009. We assigned only one GO term for each gene marker. Our intent was to follow a conservative assignment of known biochemical function based on amino acid sequence homology or direct experimental demonstration. With this approach, we avoided inferring from taxonomically distant species any specific biological role of a gene that was not corroborated by direct metabolic, developmental, or cytological studies in pine. Given that intent, our preference of GO assignments was for the molecular_function ontology. If a molecular_function term was not available for a marker's assigned RefSeq protein, then the RefSeq protein's available biological_process ontology term was used (9 cases), or we used the RefSeq protein's cellular_compartment ontology term (1 case). Benjamin Figueroa kindly provided GO terms standardized through common lineages of parent GO terms to specific hierarchical levels through use of a custom PERL script running on the Dendrome TreeGenes database [67]. The standardized terms for each assignment are in Additional file 8, and are summarized in Figure 3.

### PineMap implementation on ConiferGDB

**Figure 4:**
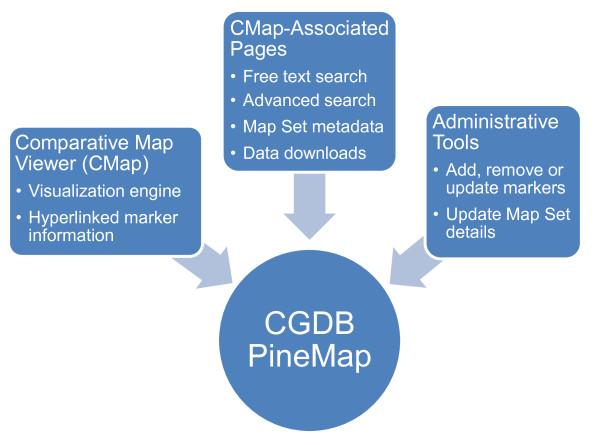
**Components of PineMap**. Diagrammatic representation of the main functional components of the PineMap user interface at ConiferGDB.

PineMap [34] was developed as an online tool to display the genetic map and marker database created by this project. In its current implementation, PineMap is based on customization and function extension of CMap, an open-source and PERL-based computational tool for displaying both genetic and physical maps [68]. Twenty-two categories of marker information, taken from Additional files 2 and 8, were inserted into the database. We modified CMap to suit the specific requirements of this project, such as including marker annotations. Using CMap as a core base system, we developed a wrapper system around it using AJAX technology based on HTML, CSS, JavaScript and PERL, that allowed us to design and implement custom pages, such as free-text searches and advanced searches. A separate web interface was created for administrative functions. This administrative page enables approved users with a username and password to add, delete and update individual marker data, as well as edit the metadata of a given map set. More modifications were incorporated to facilitate downloading of marker data and annotation for markers localised on each chromosome, which was not available in the original CMap software. A schematic diagram of the major PineMap system components is shown in Figure 4.

## Abbreviations

AFLP: amplified fragment length polymorphism; BLAST: basic local alignment search tool; cM: centiMorgan; EST: expressed sequence tag; EST-SSR: an SSR obtained from a cDNA sequence located in EST; ESTP: expressed sequence tag polymorphism; GO: Gene Ontology; LG: linkage group; LOD: log10 of the likelihood odds ratio; NCBI: National Center for Biotechnology Information; PCR: polymerase chain reaction; QTL: quantitative trait locus; SNP: single nucleotide polymorphism; SSR: simple sequence repeat; RAPD: random amplified polymorphic DNA; RFLP: restriction fragment length polymorphism.

## Authors' contributions

CSE conceived and coordinated the study, analyzed the data, annotated the markers and drafted the manuscript with contributions from all co-authors on subsequent drafts. SS mined SSR sequences from pine EST databases, wrote PERL scripts used in primer selection, and evaluated SSR primer pairs developed from *Pinus pinaster *ESTs. KVK compiled the RFLP and ESTP sequence and primer data. KW implemented PineMap at ConiferGDB and participated in designing the database and web interfaces. JEE constructed and selected SSR-enriched genomic libraries for *Pinus taeda*. CL designed, initiated, and managed the development of the PineMap database system. CDN co-conceived some of the individual SSR development projects and established the mapping population cohorts at SIFG. All authors read and approved the final manuscript.

## Supplementary Material

Additional file 1 **Table of all submitted markers' GenBank and dbSTS accession numbers**. This tab-delimited text file can be viewed with any web browser, word processor, or spreadsheet program. Click here for file

Additional file 2 **Table of data for 517 ***P. taeda ***marker loci. Data include: marker ID, map position and linkage data, database accession IDs, forward and reverse primer sequences, marker type, allele detection method, SSR type, expected and observed amplicon lengths, marker citations, aliases, and supplemental notes **This HTML data table conforms to the XHTML 1.1 standard of the World Wide Web Consortium (W3C), as determined at http://validator.w3.org, and can be viewed with any web browser, as well as with Microsoft Excel or Word. Click here for file

Additional file 3 **Provenances for 14 population samples and population genetic parameters for 185 ***P. taeda ***SSR marker loci. Data include: GenBank ID, allele frequency statistics, and assigned linkage group **This tab-delimited text file can be viewed with any web browser, word processor, or spreadsheet program. Click here for file

Additional file 4 **Marker genotype segregation codes in JoinMap format for the *base *and *qtl *pedigrees (Tables S1 - S4), reasons for excluding certain markers (Tables S5, S6), and size-estimated SSR allele genotypes of the four mapping pedigree parents and two control calibration standards (Table S7) **This tab-delimited text file can be viewed with any web browser, word processor, or spreadsheet program. Click here for file

Additional file 5 **Map data used in Figure **1, **formatted for MAPCHART input**. This tab-delimited text file can be viewed with any web browser, word processor, or spreadsheet program. Changing the file extension from .txt to .mct will allow the file to be opened by MAPCHART to generate the map graphic on a Windows platform. Click here for file

Additional file 6 Comparative *P. taeda *genetic maps: Round-2 map aligned with map of Krutovsky *et al. *2004 Click here for file

Additional file 7 **Comparative *P. taeda *genetic maps: Map of Krutovsky *et al. *2004 aligned with map of Eckert *et al. *2009**. Click here for file

Additional file 8 **Table of DNA sequence annotations for reported markers. Data include: marker and GenBank accession number, clone ID, species of origin, UniGene cluster ID, assigned GO term and GO lineage, assigned reference protein, marker type, ***P. taeda ***map status, and supplemental notes **This HTML data table conforms to the XHTML 1.1 standard of the World Wide Web Consortium (W3C), as determined at http://validator.w3.org, and can be viewed with any web browser, as well as with Microsoft Excel or Word. Click here for file
