# Supplementary material for: An annotated genetic map of loblolly pine based on microsatellite and cDNA markers
Source: BMC Genet. 2011 Jan 26;12:17. doi: 10.1186/1471-2156-12-17 (PMC3038140; doi:10.1186/1471-2156-12-17)
Supplement: Additional file 2 — Table of data for 517 P. taeda marker loci. Data include: marker ID, map position and linkage data, database accession IDs, forward and reverse primer sequences, marker type, allele detection method, SSR type, expected and observed amplicon lengths, marker citations, aliases, and supplemental notes This HTML data table conforms to the XHTML 1.1 standard of the World Wide Web Consortium (W3C), as determined at http://validator.w3.org, and can be viewed with any web browser, as well as with Microsoft Excel or Word. [file 1471-2156-12-17-S2.HTM]

Marker information for 517 loblolly pine SSR, ESTP and RFLP
loci.


Additional File 2

Acronyms used: ESTP, expressed sequence tag polymorphism; SSR, simple
sequence repeat; RFLP, restriction fragment length polymorphism; CE, denaturing
capillary electrophoresis; DGGE, denaturing gel gradient electrophoresis; SSCP,
single strand conformation polymorphism. Expected SSR, Noncontiguous SSRs are
separated by a comma; n/a, not available or unknown. Map positions are based on
round-3 mapping from JoinMap. Loci with round-2 jumps greater than 5 are noted in
the Chi-square column.

| ID | P. taeda linkage group | cM position | No. JoinMap linked pairs | Chi-square contribution to LG | GenBank Acc of STS | UniSTS ID or dbPROBE ID | F primer sequence | R primer sequence | marker type | allele detection method | expected SSR | expected marker or probe length, bp | observed amplimer length in P. taeda, bp | marker citation | PubMed ID | aliases | note1 | note2 | note3 | note4 |
| --- | --- | --- | --- | --- | --- | --- | --- | --- | --- | --- | --- | --- | --- | --- | --- | --- | --- | --- | --- | --- |
| estPaINR\_PAXY13\_a | 2 | 53.1 | 15 | 1.135 | gb|BV729064 | UniSTS:515992 | CCAGAAGCCCTACTATGACA | CCGCTCCAAAACTCCTT | ESTP | DGGE | n/a | 269 | n/a | Brown et al. (2003) | 12930758 | estPaINR\_PAXY13 | Primer sequences obtained from http://www.pierroton.inra.fr/genetics/pinus/primers.html. |  |  |  |
| estPmaLU\_SB12\_a | 6 | 10.8 | 6 | 0.694 | gb|BV729051 | UniSTS:515993 | TTATTGAGGATGTCCGTGTTC | AGAGGTAGACCATCTAGTCAC | ESTP | DGGE | n/a | 497 | n/a | Perry and Bousquet (1998) | 9611216 | estPmaLU\_SB12 |  |  |  |  |
| estPmaLU\_SB32\_a | 2 | 70.2 | 11 | 2.203 | gb|BV729050 | UniSTS:515994 | TGCTGTCTACACTGCTCAATG | CAGAAGCCTGAGGATGTTACC | ESTP | DGGE | n/a | 529 | n/a | Perry and Bousquet (1998) | 9611216 | estPmaLU\_SB32a; estPmarLU\_SB32a\_a |  |  |  |  |
| estPmaLU\_SB41\_a | 1 | 80.9 | 16 | 0.298 | gb|BV729052 | UniSTS:515995 | GCTGAGGGGAAGGATTGATAC | GCTTCGACAGGCATATTACAG | ESTP | DGGE | n/a | 404 | n/a | Perry and Bousquet (1998) | 9611216 | estPmarLU\_SB41\_a; estPmaLU\_SB41 |  |  |  |  |
| estPmaLU\_SB49\_a | 3 | 101 | 13 | 0.941 | gb|BV729054 | UniSTS:515996 | AGGTCCTCCAAAAGTTCTGTG | GCCTCATGTTCCCAAAGTCTC | ESTP | DGGE | n/a | 323 | n/a | Perry and Bousquet (1998) | 9611216 | estPmaLU\_SB49 |  |  |  |  |
| estPmaLU\_SB58\_a | 1 | 99.8 | 16 | 0.455 | gb|BV729053 | UniSTS:515997 | CCGACAATCAAATACACTGAG | TACCAGACCAGACCTTCAATG | ESTP | DGGE | n/a | 392 | n/a | Perry and Bousquet (1998) | 9611216 | estPmaLU\_SB58Pt\_a; estPmaLU\_SB58 |  |  |  |  |
| estPpINR\_AS01C10-1\_a | 2 | 129.3 | 6 | 1.233 | gb|BV729012 | UniSTS:515998 | GGCCCCCATCCTAAAATGAA | GAAGGGCCTCCAAAAGCACT | ESTP | DGGE | n/a | 298 | n/a | Brown et al. (2003) | 12930758 | estPpINR\_AS01C10 | Primer sequences obtained from http://www.pierroton.inra.fr/genetics/pinus/primers.html. |  |  |  |
| estPpINR\_RN01G04\_a | 11 | 2.8 | 9 | 0.507 | gb|BV729013 | UniSTS:515999 | GCGTCGCCGGTATCAAAATC | CACCCCATTGCACTGTGAGC | ESTP | DGGE | n/a | 175 | n/a | Krutovsky et al. (2004) | 15454556 | estPpaINRA\_RN01G04\_a; estPpINR\_RN01G04 |  |  |  |  |
| estPtIFG\_107\_a | 1 | 97.9 | 5 | 0.666 | gb|BV729025 | UniSTS:516000 | GCAGGACCTTCTGGACAATC | AGGTGGAGAAAGCCAAGCTC | ESTP | DGGE | n/a | 423 | 450 | Temesgen et al. (2001) | n/a | estPtIFG\_0107 |  |  |  |  |
| estPtIFG\_149\_a | 7 | 0.0 | 4 | 0.374 | gb|BV729058 | UniSTS:516001 | CCAATCCAACCGATACTTGG | CAAAGGTGATTGCGTCACC | ESTP | DGGE | n/a | 523 | 550 | Temesgen et al. (2001) | n/a | estPtIFG\_0149 | STS is split between Pinus taeda clones 0149e (gb|H75240) and 0149s (gb|H75141). | Both primers match homolgous Pinus taeda EST gb|DR743536, clone RTCU1\_16\_B12\_A029. |  |  |
| estPtIFG\_464\_a | 2 | 49.3 | 14 | 1.134 | gb|BV729015 | UniSTS:516002 | TGTCACTGCCCAGAGCTATTC | ATCACAGCCGCTCCAAAAC | ESTP | DGGE | n/a | 355 | 730 | Temesgen et al. (2001) | n/a | estPtIFG\_0464 | R primer does not match Pinus taeda clone 0464e (gb|H75150). | Both primers match the homolgous Pinus taeda EST gb|DR695311, clone PWAEN53. |  |  |
| estPtIFG\_500\_a | 9 | 77.4 | 14 | 1.048 | gb|BV729016 | UniSTS:516003 | GGCGAGTTGGCTTTCATTC | CAGCGAAGGTACCAGATTTGC | ESTP | DGGE | n/a | 498 | 500 | Temesgen et al. (2001) | n/a | estPtIFG\_0500 | STS is split between Pinus taeda clones 0500e (gb|H75151) and 0500s(gb|H75152). | Both primers match the homolgous Pinus taeda EST gb|DR101719, clone STRR1\_75\_G03\_A033. |  |  |
| estPtIFG\_624\_a | 9 | 53.2 | 9 | 0.545 | n/a | n/a | CACAATTGCCAGATGGGTC | CTTCTCTAGCAACGATCCGG | ESTP | DGGE | n/a | n/a | 940 | Temesgen et al. (2001) | n/a | estPtIFG\_0624 | Primers do not match either Pinus taeda clones 0624M (gb|H75105) or 0624T (gb|H75096). | Both primers match Plant Gene Index contig TC96720 which includes those clone sequences. |  |  |
| estPtIFG\_674\_a | 12 | 47.4 | 5 | 1.572 | gb|BV729063 | UniSTS:516004 | AGCAGAAGGAAGACAGAGTGG | CTCCGCATCTCCCCAATTAT | ESTP | DGGE | n/a | 272 | 270 | Temesgen et al. (2001) | n/a | estPtIFG\_0674 | The GenBank sequence of the clone is not available. | Both primers match the homologous Pinus taeda EST gb|CO160324, clone FLD1\_20\_F03\_A029. | R primer used with 5' gcaggcggcgcggggcgcgggccgggcggcgggggcgg clamp for DGGE analysis. |  |
| estPtIFG\_739\_a | 6 | 75.4 | 15 | 0.167 | gb|BV729059 | UniSTS:516005 | GTCAATGCCTAACAAGCCCTG | CCAATACCCGAGCCTTTGTA | ESTP | DGGE | n/a | 346 | 350 | Temesgen et al. (2001) | n/a | estPtIFG\_0739 | The GenBank sequence of the clone is not available. | Both primers match the homologous Pinus taeda EST gb|DR743097, clone RTCU1\_13\_C02\_A029. |  |  |
| estPtIFG\_893\_a | 5 | 49.8 | 16 | 0.455 | gb|BV729017 | UniSTS:516006 | CAGCTCAAATTCCATCGTC | GGACTGAAGGGATCTAGCTGG | ESTP | DGGE | n/a | 464 | 620 | Temesgen et al. (2001) | n/a | estPtIFG\_0893, estPtIFG\_893 | The GenBank sequence of the clone is not available. | Both primers match Pinus taeda EST gb|CO361162, clone NDL2\_3\_D09\_A029. |  |  |
| estPtIFG\_1454\_a | 5 | 84 | 16 | 0.335 | gb|BV729027 | UniSTS:516007 | ACATCAATCAAGTTGGCCTTG | ACGACCATCTCCAACCACTC | ESTP | DGGE | n/a | 338 | 350 | Temesgen et al. (2001) | n/a | estPtIFG\_1454 |  |  |  |  |
| estPtIFG\_1576\_a | 1 | 50.1 | 18 | 0.573 | gb|BV729024 | UniSTS:516008 | TGGTATGTGGAGGGAAGGC | TACAGCGTTTGCTCCTCCTG | ESTP | DGGE | n/a | 477 | 480 | Temesgen et al. (2001) | n/a | estPtIFG\_1576 | Sequence of Pinus taeda clone 1576s is not available. | Both primers match Pinus taeda EST gb|DR681980, clone PWAA474. |  |  |
| estPtIFG\_1643\_a | 10 | 61.2 | 12 | 2.675 | gb|BV729048 | UniSTS:516009 | AATGGAGGATGCCGTTACAG | AACCACTCTCGAATCCCCAC | ESTP | DGGE | n/a | 481 | 650 | Temesgen et al. (2001) | n/a | estPtIFG\_1643 | STS is split between Pinus taeda clones 1643e (gb|H75191) and 1643s (gb|H75192). | Both primers match Plant Gene Index pine contig TC113228 which includes those clone sequences. |  |  |
| estPtIFG\_1750\_a | 11 | 86.2 | 5 | 0.239 | gb|BV729060 | UniSTS:516010 | TAGCAAGCACTCTGACTGTGG | CCGCGTCAAGAACGTAAACA | ESTP | DGGE | n/a | 299 | 300 | Temesgen et al. (2001) | n/a | estPtIFG\_1750 | STS is split between Pinus taeda clones 1750s (gb|H75022) and 0739e (gb|H75195). | Both sequences match the homolgous Pinus taeda EST gb|DR102530, clone STRR1\_81\_F12\_A033. |  |  |
| estPtIFG\_1934\_a | 3 | 120.0 | 15 | 0.478 | gb|BV729018 | UniSTS:516011 | TTCTGTTTGTGCGCCTACTG | GACGAAGTTGGTGGCATAG | ESTP | DGGE | n/a | 841 | 850 | Temesgen et al. (2001) | n/a | estPtIFG\_1934 | STS is split between Pinus taeda clones 1934M (gb|H75112) and 1934T (gb|H75103). | Both ESTs are homolgous with Pinus taeda EST gb|DR095068, clone STRR1\_18\_C09\_A033. |  |  |
| estPtIFG\_1950\_a | 6 | 59.2 | 20 | 1.109 | gb|BV729026 | UniSTS:516012 | AAACCAGCAGCCACATGAG | TATTAAGAAGGCGGCGGTAC | ESTP | DGGE | n/a | 340 | 450 | Temesgen et al. (2001) | n/a | estPtIFG\_1950 |  |  |  |  |
| estPtIFG\_1955\_a | 5 | 88.8 | 14 | 0.301 | n/a | n/a | AGCCAATGCACCAAGAAGG | ATCCAACAACAGAACCCCTC | ESTP | DGGE | n/a | n/a | 290 | Temesgen et al. (2001) | n/a | estPtIFG\_1955 | R primer does not match Pinus taeda clone 1955e (gb|H75038) or any other P. taeda EST. |  |  |  |
| estPtIFG\_1956\_a | 9 | 7.1 | 5 | 0.092 | gb|BV729019 | UniSTS:516013 | GAAGCTAGCGAAGGCTTTGG | GGGTGTGACCATATAACACCG | ESTP | DGGE | n/a | 346 | 500 | Temesgen et al. (2001) | n/a | estPtIFG\_1956 |  | F and R primers may be reversed from what is in the NCBI UniSTS record. |  |  |
| estPtIFG\_2166\_a | 4 | 59.9 | 6 | 0.157 | gb|BV729020 | UniSTS:516014 | CTGCTGTTGAGCTTGTGTACG | TGCCCGTCGTAAAGATGACAG | ESTP | DGGE | n/a | 419 | 400 | Temesgen et al. (2001) | n/a | estPtIFG\_2166 | STS is split between Pinus taeda clones 2166s (gb|H75060) and 2166e (gb|H75059). | Both ESTs are homolgous with Pinus taeda EST gb|DT624335, clone PIMAD52. |  |  |
| estPtIFG\_2290\_a | 9 | 66 | 15 | 0.789 | gb|BV729021 | UniSTS:516015 | AGCTTGCAGCATCAACCG | GAACCAAACAGCTTCAGGACC | ESTP | DGGE | n/a | 425 | 840 | Temesgen et al. (2001) | n/a | estXUOU\_LHCA2; PsUMU\_p1NEab21; estPtIFG\_2290 | R primer does not match Pinus taeda clone 2290e (gb|H75067). | Both primers match the homolgous Pinus taeda EST gb|DR101524, clone STRR1\_73\_H05\_A033. | PsUMU\_p1NEab21 and estXUOU\_LHCA2 are from gb|X58516; Kumolainen et al. 2003 |  |
| estPtIFG\_2358\_a | 6 | 0.0 | 6 | 0.458 | gb|BV729049 | UniSTS:516016 | GTTAACCCTCGAGGAGACATG | GCTTCCACAGTCCACAATCTG | ESTP | DGGE | n/a | 327 | 330 | Temesgen et al. (2001) | n/a | estPtIFG\_2358 | Primers do not match Pinus taeda clone 2358e (gb|H75088), or any other pine EST. | Both primers match Plant Gene Index contig TC59982 which includes that clone sequence. |  |  |
| estPtIFG\_2615\_a | 11 | 37.5 | 9 | 1.575 | gb|BV729062 | UniSTS:516017 | TCGGTTAGGTAACGACTGGAC | CGAAGGGCAAGAATAAAGAGTG | ESTP | DGGE | n/a | 317 | 400 | Temesgen et al. (2001) | n/a | estPtIFG\_2615 |  |  |  |  |
| estPtIFG\_2781\_a | 8 | 100.8 | 16 | 0.464 | gb|BV729022 | UniSTS:516018 | GATGATGCCCTGAAGAGCC | ATGGAACCAAAGGAGATGCC | ESTP | DGGE | n/a | 448 | 450 | Temesgen et al. (2001) | n/a | estPtIFG\_2781 | R primer does not match Pinus taeda clone 2781e (gb|H75231). |  |  |  |
| estPtIFG\_2889\_a | 3 | 20.14 | 7 | 3.71 | gb|BV729023 | UniSTS:516019 | ACGCCAGCTCTGACTACCAG | GTTTCTTCTCGTGGTGCTCG | ESTP | DGGE | n/a | 453 | 750 | Temesgen et al. (2001) | n/a | estPtIFG\_2889 | STS is split between Pinus taeda clones 2889e (gb|H75234) and 2889s (gb|H75235). | Both ESTs are homolgous with Pinus taeda EST gb|DR744329, clone RTCU1\_21\_F07\_A029. |  |  |
| estPtIFG\_4CL\_a | 7 | 60.4 | 9 | 0.92 | gb|BV728981 | UniSTS:516020 | CCCCGTCAAATCTGGCTCCT | GGGCGCTTACTCTGCACCAC | ESTP | DGGE | n/a | 658 | n/a | Brown et al. (2003) | 12930758 | estPtIFG\_4CL | Primer sequences obtained from http://www.pierroton.inra.fr/genetics/pinus/primers.html. |  |  |  |
| estPtIFG\_8415\_a | 9 | 110.4 | 13 | 2.341 | gb|BV728982 | UniSTS:516021 | ACCTTGTTGTGGATGGCG | GCAACCTCCCATACCAAGAC | ESTP | DGGE | n/a | 245 | n/a | Brown et al. (2003) | 12930758 | estPtIFG\_8415 |  |  | F primer used with 5' cgcgcgg clamp for DGGE analysis. |  |
| estPtIFG\_8429\_a | 4 | 84.7 | 12 | 0.293 | gb|BV728998 | UniSTS:516022 | GAGGCATTTATGAGGGAACG | GTTGAAAGCGACTCCAAAGG | ESTP | DGGE | n/a | 251 | n/a | Brown et al. (2001) | 11606554 | estPtIFG\_8429 |  |  | R primer used with 5' cgcgcgg clamp for DGGE analysis. |  |
| estPtIFG\_8471\_a | 5 | 33.8 | 10 | 3.268 | gb|BV729009 | UniSTS:516023 | AGTCAGAGGCCATGTTTTGG | TGAACCACTACATTCCCCTCC | ESTP | DGGE | n/a | 194 | n/a | Brown et al. (2001) | 11606554 | estPtIFG\_8471 |  |  | F primer used with 5' ggcccggcgg clamp and R primer used with 5' ggc clamp for DGGE analysis. |  |
| estPtIFG\_8473\_a | 6 | 92.9 | 7 | 4.25, jump>5 | gb|BV729010 | UniSTS:516024 | CTCTGGAGAACGACTGCAAG | TACAGTAGCCTCTCTGGCCC | ESTP | DGGE | n/a | 193 | n/a | Brown et al. (2001) | 11606554 | estPtIFG\_8473 |  |  | F primer used with 5' ccggccggg clamp for DGGE analysis. |  |
| estPtIFG\_8500\_a | 3 | 96.8 | 19 | 1.277 | gb|BV729004 | UniSTS:516025 | GCAGATCGGACGATTAAAGG | TCTGTACAAAACCGGATGGG | ESTP | DGGE | n/a | 223 | n/a | Brown et al. (2001) | 11606554 | estPtIFG\_8500 |  |  | F primer used with 5' ggcccggcgg clamp and R primer used with 5' gcc clamp for DGGE analysis. |  |
| estPtIFG\_8531\_a | 6 | 17.6 | 6 | 0.789 | gb|BV729000 | UniSTS:516026 | TTCAGCTGGAAACACCTCAC | CTCGTGATAGCACAGCAAATACTC | ESTP | DGGE | n/a | 239 | n/a | Brown et al. (2001) | 11606554 | estPtIFG\_8531 |  |  | F primer used with 5' cccggccgcg clamp for DGGE analysis. |  |
| estPtIFG\_8542\_a | 12 | 42.9 | 12 | 0.816 | gb|BV729006 | UniSTS:516027 | TTGGCATATGGAGGCATG | GATGCCCAAAGCATGACATC | ESTP | DGGE | n/a | 222 | n/a | Brown et al. (2001) | 11606554 | estPtIFG\_8542 |  |  | F primer used with 5' ggcccggcgg clamp and R primer used with 5' gc clamp for DGGE analysis. |  |
| estPtIFG\_8564\_a | 6 | 83.6 | 11 | 4.2, jump>5 | gb|BV729044 | UniSTS:516028 | CACCAGGGCAAAAAGTTGG | GCAGTTATAGGTTTCCTGGCC | ESTP | DGGE | n/a | 232 | 230 | Temesgen et al. (2001) | n/a | estPtIFG\_8564 |  |  |  |  |
| estPtIFG\_8565\_a | 12 | 71.8 | 5 | 2.292 | gb|BV729047 | UniSTS:516029 | ATTTGTGGCTGCGGAAAG | CACCAAGTACACCACAACACC | ESTP | DGGE | n/a | 201 | 220 | Temesgen et al. (2001) | n/a | estPtIFG\_8565 |  |  |  |  |
| estPtIFG\_8569\_a | 2 | 20.1 | 14 | 0.213 | gb|BV729061 | UniSTS:516030 | TCGTCTCCCTCATCACCTTC | CCTCTGCAACACTGGTCGA | ESTP | DGGE | n/a | 209 | 210 | Temesgen et al. (2001) | n/a | estPtIFG\_8569 | F primer does not match Pinus taeda clone 8569M (gb|AA739585). | Both primers match the homologous Pinus taeda EST gb|DR745022, clone RTCU1\_26\_B08\_A029. |  |  |
| estPtIFG\_8580\_a | 10 | 17 | 13 | 0.214 | gb|BV729011 | UniSTS:516031 | ACTGGATTCCGGAGGATCAC | TGGAAACCGTCTACAGTCGC | ESTP | DGGE | n/a | 180 | n/a | Brown et al. (2001) | 11606554 | estPtIFG\_8580 |  |  |  |  |
| estPtIFG\_8612\_a | 3 | 52.53 | 24 | 0.595 | gb|BV729007 | UniSTS:516032 | GAAGGGCACTATGAAGCTGC | AACTAGGAATCCCAAATTCCC | ESTP | DGGE | n/a | 218 | n/a | Brown et al. (2001) | 11606554 | estPtIFG\_8612 |  |  | R primer used with 5' gcggccgg clamp for DGGE analysis. |  |
| estPtIFG\_8647\_a | 6 | 23.8 | 8 | 2.298 | gb|BV729008 | UniSTS:516033 | TTGGTCCGCTGATTGGAG | ACTTACGGTGGAGACCTTACAC | ESTP | DGGE | n/a | 208 | n/a | Brown et al. (2001) | 11606554 | estPtIFG\_8647 |  |  | F primer used with 5' ggccggg clamp for DGGE analysis. |  |
| estPtIFG\_8702\_a | 6 | 87.6 | 18 | 1.338 | gb|BV729033 | UniSTS:516034 | GTTGCAGAAAAGGGTGGC | AGTCGCACTTGCTCCAGTTC | ESTP | DGGE | n/a | 291 | 350 | Temesgen et al. (2001) | n/a | estPtIFG\_8702 |  |  |  |  |
| estPtIFG\_8725\_a | 9 | 125.4 | 6 | 0.353 | gb|BV728997 | UniSTS:516035 | AGCGCTGAATGATGTCTTGG | CCAAACTTACACCATGCTCG | ESTP | DGGE | n/a | 259 | n/a | Brown et al. (2001) | 11606554 | estPtIFG\_8725 |  |  | R primer used with 5' gccgggcccggc clamp for DGGE analysis. |  |
| estPtIFG\_8732\_a | 8 | 57.2 | 17 | 1.689 | gb|BV729003 | UniSTS:516036 | TGAAGTTCTGAGTTTGGCGG | ATGCACAGGATGAACCCTTC | ESTP | DGGE | n/a | 228 | n/a | Brown et al. (2001) | 11606554 | estPtIFG\_8732 |  |  | F primer used with 5' ggcccggcggccgg clamp and R primer used with 5' gcc clamp for DGGE analysis. |  |
| estPtIFG\_8738\_a | 4 | 106.4 | 6 | 1.247 | gb|BV728999 | UniSTS:516037 | TCACAGACCTGAACACTGCG | CCAAAACCGACTATCTTGGG | ESTP | DGGE | n/a | 242 | n/a | Brown et al. (2001) | 11606554 | estPtIFG\_8738 |  |  | F primer used with 5' g clamp and R primer used with 5' ggcccggcgg clamp for DGGE analysis. |  |
| estPtIFG\_8781\_a | 3 | 11.9 | 11 | 0.583 | gb|BV729005 | UniSTS:516038 | GGTTTGCTCACATGTTCGTG | TCCTTCATAAATGCATTCTCGTC | ESTP | DGGE | n/a | 223 | n/a | Brown et al. (2001) | 11606554 | estPtIFG\_8781 |  |  | F primer used with 5' ccggccggg clamp for DGGE analysis. |  |
| estPtIFG\_8837\_a | 5 | 48.5 | 13 | 0.757 | gb|BV729002 | UniSTS:516039 | TCGGTTACTTGGGACCTACTG | TCCCATGCAATATCATTTGTG | ESTP | DGGE | n/a | 230 | n/a | Brown et al. (2001) | 11606554 | estPtIFG\_8837 |  |  | F primer used with 5' ccggccggg clamp for DGGE analysis. |  |
| estPtIFG\_8886\_a | 7 | 129.1 | 4 | 0.525 | gb|BV729029 | UniSTS:516040 | TTCCGGAAGGTGTGGTGG | AGTCACTCCCTGTCACCGAC | ESTP | DGGE | n/a | 315 | 310 | Temesgen et al. (2001) | n/a | estPtIFG\_8886 |  |  |  |  |
| estPtIFG\_8887\_a | 4 | 100.8 | 8 | 0.358 | gb|BV729028 | UniSTS:516041 | TGGGGTTGGTGAGATACTGC | CATATATTGGGAAAACGTTCGC | ESTP | DGGE | n/a | 317 | 500 | Temesgen et al. (2001) | n/a | estPtIFG\_8887 |  |  |  |  |
| estPtIFG\_8898\_a | 4 | 123 | 10 | 2.213 | gb|BV729045 | UniSTS:516042 | GGGATGGCAACAACAAAAAG | ATGGGGGTGCAGCATAAAC | ESTP | DGGE | n/a | 216 | 1400 | Temesgen et al. (2001) | n/a | estPtIFG\_8898 |  |  |  |  |
| estPtIFG\_8939\_a | 2 | 74.1 | 15 | 2.375 | gb|BV729030 | UniSTS:516043 | ACGTGGACGAGCAGTCAAAG | AACCACGAGCTTGGCATG | ESTP | DGGE | n/a | 314 | 300 | Temesgen et al. (2001) | n/a | estPtIFG\_8939 |  |  |  |  |
| estPtIFG\_8972\_a | 6 | 25.7 | 16 | 1.97, jump>5 | gb|BV729031 | UniSTS:516044 | TTGGTCCCCTTGTTGGAG | GCCTCCATTCGACTCACTTG | ESTP | DGGE | n/a | 307 | 310 | Temesgen et al. (2001) | n/a | estPtIFG\_8972 |  |  |  |  |
| estPtIFG\_9008\_a | 5 | 82.2 | 15 | 0.8 | gb|BV729035 | UniSTS:516045 | GGTAAACTGGGATGGATTGC | TCTCGGATAGGGCAATATGC | ESTP | DGGE | n/a | 288 | 290 | Temesgen et al. (2001) | n/a | estPtIFG\_9008 |  |  | F primer used with 5' ggcgccc clamp for DGGE analysis. |  |
| estPtIFG\_9022\_a | 2 | 29.4 | 18 | 0.293 | gb|BV729046 | UniSTS:516046 | CGGTGTGTTTCATGTGCTG | GGATTTGCATTTTGCATGCC | ESTP | DGGE | n/a | 209 | 210 | Temesgen et al. (2001) | n/a | estPtIFG\_9022 |  |  |  |  |
| estPtIFG\_9036\_a | 8 | 72.1 | 17 | 0.463 | gb|BV729038 | UniSTS:516047 | CAGGACGAATGAGATACCTGC | GTCATCCGATACAACCTCAATC | ESTP | DGGE | n/a | 280 | 350 | Temesgen et al. (2001) | n/a | estPtIFG\_9036 |  |  |  |  |
| estPtIFG\_9044\_a | 6 | 77.9 | 6 | 0.547 | gb|BV729041 | UniSTS:516048 | AACTGGAGGAAAAGCACGAC | CATCGCATCAGTCATACTCACC | ESTP | DGGE | n/a | 266 | 280 | Temesgen et al. (2001) | n/a | estPtIFG\_9044 |  |  | F primer used with 5' cgcggccc clamp for DGGE analysis. |  |
| estPtIFG\_9053\_a | 1 | 25.8 | 18 | 4.58, jump>5 | gb|BV729036 | UniSTS:516049 | TGCATGATGACGGCTCTATG | CCACCGAAATATATGCCTGTC | ESTP | DGGE | n/a | 283 | 280 | Temesgen et al. (2001) | n/a | estPtIFG\_9053 |  |  | F primer used with 5' ggcgg clamp for DGGE analysis. |  |
| estPtIFG\_9076\_a | 11 | 16.7 | 9 | 0.908 | gb|BV729042 | UniSTS:516050 | AGAATTTACTGGCCGCTCG | CTCTATTGCAAAAATGTGCCAC | ESTP | DGGE | n/a | 253 | 250 | Temesgen et al. (2001) | n/a | estPtIFG\_9076 |  |  |  |  |
| estPtIFG\_9092\_a | 5 | 51.5 | 15 | 0.731 | gb|BV729040 | UniSTS:516051 | TCACTGACCTTAACGTCCC | AGCTAAAGTTGGCTGGCATC | ESTP | DGGE | n/a | 267 | 400 | Temesgen et al. (2001) | n/a | estPtIFG\_9092 |  |  | F primer used with 5' ggccggg clamp for DGGE analysis. |  |
| estPtIFG\_9102\_a | 1 | 73.5 | 16 | 0.982 | gb|BV729043 | UniSTS:516052 | CCCAGAGATCTTCCGCTATG | AGAAAGGAGCATTTCCCGAC | ESTP | DGGE | n/a | 238 | 240 | Temesgen et al. (2001) | n/a | estPtIFG\_9102 |  |  | R primer used with 5' ggccgcggc clamp for DGGE analysis. |  |
| estPtIFG\_9113\_a | 8 | 117.8 | 12 | 0.749 | gb|BV729032 | UniSTS:516053 | AGGAAAAGGTTCTCCAAGCG | ACAGCTTAGGCATTACAGCCC | ESTP | DGGE | n/a | 303 | 300 | Temesgen et al. (2001) | n/a | estPtIFG\_9113 |  |  | R primer used with 5' gccggccg clamp for DGGE analysis. |  |
| estPtIFG\_9151\_a | 7 | 60.8 | 16 | 0.701 | gb|BV729034 | UniSTS:516054 | TAGTGAGCCCTGGAGCGTAC | GCAGAATCTCAGCAGCAATG | ESTP | DGGE | n/a | 291 | 290 | Temesgen et al. (2001) | n/a | estPtIFG\_9151 |  |  |  |  |
| estPtIFG\_9156\_a | 9 | 16.3 | 11 | 0.462 | gb|BV729037 | UniSTS:516055 | TAAGCTTCGTGCAACAGGAG | GACAATCCCTCTAAACCTCGC | ESTP | DGGE | n/a | 281 | 400 | Temesgen et al. (2001) | n/a | estPtIFG\_9156 |  |  | F primer used with 5' ggcccgcgcccg clamp for DGGE analysis. |  |
| estPtIFG\_9157\_a | 4 | 25.9 | 6 | 0.543 | gb|BV729039 | UniSTS:516056 | TTCCAGTTTCCCTGAGCATC | AATACGCTGCTTAATCGTGTC | ESTP | DGGE | n/a | 273 | 275 | Temesgen et al. (2001) | n/a | estPtIFG\_9157 |  |  | R primer used with 5' ccgcgggccgcggcc clamp for DGGE analysis. |  |
| estPtIFG\_9164\_a | 9 | 17.9 | 8 | 0.653 | gb|BV728996 | UniSTS:516057 | TACTGCGAATGCAACTCCAG | CAGCAAAGAGGCTTCAAAGG | ESTP | DGGE | n/a | 219 | n/a | Brown et al. (2001) | 11606554 | estPtIFG\_9164 | R primer does not match Pinus taeda clone 9164M (gb|AA739966). | Both primers match the homolgous Pinus taeda EST gb|DR168058, clone RTPHOS1\_22\_H07\_A029. | F primer used with 5' gcggcc clamp for DGGE analysis. |  |
| estPtIFG\_9198\_a | 1 | 66.8 | 16 | 0.615 | gb|BV729001 | UniSTS:516058 | CGGCGGTGGCATAAGTTAC | TCCAAGTCTTCTCAATCCGG | ESTP | DGGE | n/a | 236 | n/a | Brown et al. (2001) | 11606554 | estPtIFG\_9198 |  |  | R primer used with 5' gcggccggg clamp for DGGE analysis. |  |
| estPtIFG\_C4H-1\_a | 3 | 47.2 | 25 | 0.646 | n/a | n/a | n/a | n/a | ESTP | DGGE | n/a | n/a | n/a | Chagne et al. (2003) | n/a | estPtIFG\_4CH-1\_a; estPtIFG\_C4H-1 |  |  |  |  |
| estPtIFG\_C4H-2\_a | 10 | 26.4 | 13 | 0.452 | n/a | n/a | n/a | n/a | ESTP | DGGE | n/a | n/a | n/a | Chagne et al. (2003) | n/a | estPtIFG\_4CH-2\_a; estPtIFG\_C4H-2 |  |  |  |  |
| estPtIFG\_COMT-2\_a | 11 | 89.3 | 11 | 1.202 | gb|BV729056 | UniSTS:516059 | AACTAATAATTCGCTTTGTGAAACATACAT | TACCGACTCTGCTTGGCCTT | ESTP | DGGE | n/a | 223 | n/a | Brown et al. (2003) | 12930758 | PtNCS\_5c9a; estPtIFG\_COMT-2 |  |  | F primer used with 5' gcgggcggcgggcgg clamp and R primer used with 5' cggcggcggcgg clamp for DGGE analysis. |  |
| estPtIFG\_lp3-3\_a | 2 | 68.7 | 15 | 0.753 | n/a | n/a | GGAAGATGAAAACGACAACC | AGACGTAACCCCCTTCTCC | ESTP | FP-TDI | n/a | n/a | n/a | Gonzalez-Martinez et al. (2006) | 16387885 | estPtIFG\_F3R3; estPtIFG\_lp3-3 |  |  |  |  |
| estPtIFG\_SAHH\_a | 11 | 0.9 | 9 | 0.325 | gb|BV728983 | UniSTS:516060 | TGGGTGCCAAGCTAACAAAGC | TGCCGACAAAGAGAGGCGA | ESTP | pyrosequencing | n/a | 322 | n/a | Brown et al. (2003) | 12930758 | estPtIFG\_SAHH |  |  |  |  |
| estPtIFG\_SB32b\_a | 12 | 85 | 10 | 0.87 | n/a | n/a | n/a | n/a | ESTP | DGGE | n/a | n/a | n/a | Krutovsky et al. (2004) | 15454556 | estPmarLU\_SB32b\_a; estPtIFG\_SB32b |  |  |  |  |
| estPtNCS\_22B8\_a | 3 | 46.37 | 22 | 1.57 | gb|BV728984 | UniSTS:516062 | CCACACAACCACCAGATTGC | CAGGTCACACACTTTCTCCACC | ESTP | DGGE | n/a | 228 | n/a | Krutovsky et al. (2004) | 15454556 | PtDOE\_22B8; estPtNCS\_22B8 |  |  | F primer used with 5' gccgccgccc clamp and R primer used with 5' gc clamp for DGGE analysis. |  |
| estPtNCS\_22C5\_a | 3 | 83.59 | 20 | 0.956 | gb|BV728985 | UniSTS:516063 | ATCCTAAGTGGCATGGGTTTATTC | CAATCACCAAAATGAACATATACGC | ESTP | DGGE | n/a | 245 | n/a | Brown et al. (2003) | 12930758 | PtDOE\_22C5; estPtNCS\_22C5 | Primers do not match Pinus taeda clone 22C5 (gb|AI812900). | Both primers the match the homologous Pinus taeda EST gb|AA556727, clone 2NA11H. | F primer used with 5' ggcgggcggcgggc clamp and R primer used with 5' cggcggg clamp for DGGE analysis. |  |
| estPtNCS\_22C8\_a | 5 | 86.1 | 29 | 0.534 | gb|BV728986 | UniSTS:516064 | TGCACAGGACGAAGACGC | GCGCTTGATAGAACACAACCAG | ESTP | DGGE | n/a | 286 | n/a | Brown et al. (2003) | 12930758 | estPtIFG\_22C8; estPtIFG\_22C8(perox); estPtNCS\_22C8 | Primers do not match Pinus taeda clone 22C8 (gb|AI812903). | Both primers match the homologous Pinus taeda EST gb|DR016450, clone STRS1\_10\_E09\_A034. | F primer used with 5' ggcgggcggcgg clamp and R primer used with 5' gcgg clamp for DGGE analysis. |  |
| estPtNCS\_23C5\_a | 6 | 9.9 | 5 | 0.715 | gb|BV728987 | UniSTS:516065 | CCCAACAGAATCAAGGACTGC | TAGAAATAAGGGAGCCTCACGC | ESTP | DGGE | n/a | 265 | n/a | Krutovsky et al. (2004) | 15454556 | PAL-2; estPtIFG\_23C5; estPtNCS\_23C5 | Primers do not match Pinus taeda clone 23C5 (gb|AI813128). | Both primers match the homologous Pinus taeda EST gb|CF473870, clone RTWW2\_19\_D07\_A021. | F primer used with 5' gccgccgccg clamp for DGGE analysis. |  |
| estPtNCS\_2N7G\_a | 4 | 107.7 | 13 | 0.908 | gb|BV728988 | UniSTS:516066 | TGAGGGAGACGAGGATGAGG | GCATTCAAGTGCGAGAGTCG | ESTP | DGGE | n/a | 260 | n/a | Brown et al. (2003) | 12930758 | estPtIFG\_2N7G(tub); estPtNCS\_2N7G |  |  | F primer used with 5' gcgggcggcggg clamp for DGGE analysis. |  |
| estPtNCS\_6C12A\_a | 4 | 74.7 | 7 | 0.338 | gb|BV728989 | UniSTS:516067 | GACGGACTCCAAATAGATTGCC | GATCGAATAACAAATGTAGGTGGCTA | ESTP | DGGE | n/a | 305 | n/a | Brown et al. (2003) | 12930758 | estPtNCS\_6C12A |  |  | F primer used with 5' ggcggcggc clamp and R primer used with 5' gcg clamp for DGGE analysis. |  |
| estPtNCS\_6C5A\_a | 4 | 64.7 | 11 | 0.169 | n/a | n/a | TCTACCCAGATGTCCAATACACTACTG | TTCCAAAACACTCACAGACAAAGAG | ESTP | DGGE | n/a | n/a | n/a | Chagne et al. (2003) | n/a | estPtIFG\_6C5A; estPtNCS\_6C5A |  |  | F primer used with 5' ggcgggcgggcg clamp for DGGE analysis. |  |
| estPtNCS\_6N3C\_a | 11 | 16.3 | 9 | 0.607 | gb|BV729057 | UniSTS:516068 | GGCCAGATAGCCAAGACCAATA | CATAATAATCACGGTGGTTAATCCTG | ESTP | DGGE | n/a | 232 | n/a | Brown et al. (2003) | 12930758 | estPtIFG\_6N3C(cesA); estPtNCS\_6N3C |  |  | F primer used with 5' ggcggggc clamp for DGGE analysis. |  |
| estPtNCS\_6N3E\_a | 1 | 105 | 10 | 0.194 | gb|BV728990 | UniSTS:516069 | CTTATGATGTTCCACCTGGCATT | ACTGAAGTGATGTTGTTACAGCAAAGT | ESTP | DGGE | n/a | 342 | n/a | Brown et al. (2003) | 12930758 | estPtNCS\_6N3E |  |  | F primer used with 5' ggcggcgg clamp and R primer used with 5' gcgg clamp for DGGE analysis. |  |
| estPtNCS\_CCoAOMT\_a | 6 | 93.5 | 14 | 0.727 | gb|BV728992 | UniSTS:516071 | TTTGCAGGCGTGTCTATTGA | CGAAATGGCGAAGAAAACAT | ESTP | DGGE | n/a | 195 | n/a | Brown et al. (2003) | 12930758 | estPtNCS\_CCoAOMT |  |  | F primer used with 5' gggcggcggcggg clamp for DGGE analysis. |  |
| NZPR0102c | 12 | 73 | 19 | 1.89 | n/a | n/a | ATTCACTCACATCGGCAACTC | GCTCCAAGGTGATTGAAATCTC | SSR | CE | (GA)21 | n/a | 94 | Echt et al. (this paper) | n/a |  |  |  | F primer was evaluated with a 5' dye-CACGACGTTGTAAAACGAC tail. R primer was evaluated with a 5' GTTTCTT tail. |  |
| NZPR0116 | 6 | 75.5 | 13 | 0.73 | n/a | n/a | ATACCTAGTTATCATTTAAATAAATGC | CTCTCAGTAAGTCGAAGAGAGTATC | SSR | CE | (GC)5, (CA)13, (TA)6 | n/a | 125 | Echt et al. (this paper) | n/a |  |  |  | F primer was evaluated with a 5' dye-CACGACGTTGTAAAACGAC tail. R primer was evaluated with a 5' GTTTCTT tail. |  |
| NZPR0143 | 2 | 71.7 | 11 | 1.56 | n/a | n/a | GAAAGCATTAGCCATCTACATTCA | TCATTGTGCATGCATTTATAATCTC | SSR | CE | (CA)18 | n/a | 102 | Echt et al. (this paper) | n/a |  |  |  | F primer was evaluated with a 5' dye-CACGACGTTGTAAAACGAC tail. R primer was evaluated with a 5' GTTTCTT tail. |  |
| NZPR0206 | 5 | 53.8 | 24 | 5.93 | gb|BV728907 | UniSTS:516211 | TTGATGGGCTTGGTCCATAT | ATGCAAGGTAAGGTGGTTGG | SSR | CE | (CA)19, (TTA)5 | 252 | 239 | Echt et al. (this paper) | n/a |  |  |  | F primer was evaluated with a 5' dye-CACGACGTTGTAAAACGAC tail. R primer was evaluated with a 5' GTTTCTT tail. |  |
| NZPR0269 | 4 | 20.9 | 8 | 0.09 | gb|BV728908 | UniSTS:516212 | TTCATCACATATCAACATCTTCCA | TTAAAAGCCACGTCTCCCAG | SSR | CE | (CA)12, (CA)9 | 250 | 278 | Echt et al. (this paper) | n/a |  |  |  | F primer was evaluated with a 5' dye-CACGACGTTGTAAAACGAC tail. R primer was evaluated with a 5' GTTTCTT tail. | Locus segregates a null allele. |
| NZPR0274 | 12 | 92.1 | 9 | 1.15 | gb|BV728909 | UniSTS:516213 | CGACATCAAAGTGACGATGG | TGGGATTGTATGCATGTCTCA | SSR | CE | (GT)23 | 321 | 304 | Echt et al. (this paper) | n/a |  |  |  | F primer was evaluated with a 5' dye-CACGACGTTGTAAAACGAC tail. R primer was evaluated with a 5' GTTTCTT tail. |  |
| NZPR0290 | 6 | 57.1 | 28 | 2.11 | gb|BV728910 | UniSTS:516214 | TGGGTTTTATTTCCACCTGC | AACCCAAAAATGATGCCTTG | SSR | CE | (AC)36 | 251 | 210 | Echt et al. (this paper) | n/a |  |  |  | F primer was evaluated with a 5' dye-CACGACGTTGTAAAACGAC tail. R primer was evaluated with a 5' GTTTCTT tail. | Locus segregates a null allele. |
| NZPR0300 | 5 | 56.4 | 30 | n/a | gb|BV728911 | UniSTS:516215 | CACTACACACTGCACACATGC | TTGGTGTTTGTTTTGTGGGA | SSR | CE | (AC)6, (AC)7, (CA)5, (CA)10(CACACG)8 | 333 | 304 | Echt et al. (this paper) | n/a |  |  |  | F primer was evaluated with a 5' dye-CACGACGTTGTAAAACGAC tail. R primer was evaluated with a 5' GTTTCTT tail. |  |
| NZPR0351 | 7 | 63.1 | 18 | 0.51 | gb|BV728912 | UniSTS:516216 | GGGCGAACAACCAACTCTAG | TTCCAAGATGCATGAACACA | SSR | CE | (TG)6(GT)16 | 280 | 272 | Echt et al. (this paper) | n/a |  |  |  | F primer was evaluated with a 5' dye-CACGACGTTGTAAAACGAC tail. R primer was evaluated with a 5' GTTTCTT tail. | Locus segregates a 1bp allele. |
| NZPR0413 | 4 | 90.2 | 19 | 3.78 | gb|BV728913 | UniSTS:516217 | TGAACCTCGATGGAATAGCC | CCCGCCTTGCATCAATTA | SSR | CE | (TG)23, (GT)6 | 253 | 209 | Chagne et al. (2004) | 15448894 | NZPR413 |  |  | F primer was evaluated with a 5' dye-CACGACGTTGTAAAACGAC tail. R primer was evaluated with a 5' GTTTCTT tail. |  |
| NZPR0440 | 5 | 56.4 | 30 | 1.42 | gb|BV728914 | UniSTS:516218 | GGGGAGGCTATCTCATCTGC | GGATTGCAGTGGCATTGTTA | SSR | CE | (TG)15 | 175 | 160 | Echt et al. (this paper) | n/a |  |  |  | F primer was evaluated with a 5' dye-CACGACGTTGTAAAACGAC tail. R primer was evaluated with a 5' GTTTCTT tail. |  |
| NZPR0458 | 10 | 80.0 | 15 | 24.543 | gb|BV728929 | UniSTS:516219 | TGGATCATGATTGTCTCTATGC | TCCTCATAGCCAGGAACCTC | SSR | CE | (TG)10 | 253 | 254 | Echt et al. (this paper) | n/a |  |  |  | F primer was evaluated with a 5' dye-CACGACGTTGTAAAACGAC tail. R primer was evaluated with a 5' GTTTCTT tail. | Locus segregates a null allele. |
| NZPR0473 | 3 | 73.97 | 28 | 3.18 | gb|BV728915 | UniSTS:516220 | TGTATCCATGTTCAAGGGCA | ACCTCAGTCCAACCAGCATC | SSR | CE | (CA)12(AT)5 | 161 | 167 | Echt et al. (this paper) | n/a |  |  |  | F primer was evaluated with a 5' dye-CACGACGTTGTAAAACGAC tail. R primer was evaluated with a 5' GTTTCTT tail. | Locus segregates a null allele. |
| NZPR0563 | 8 | 9.4 | 21 | 2.34, jump>5 | gb|BV728916 | UniSTS:516221 | GCATTTCTTGTTGCTATTTTCAA | GCACAAGTCCCATTTCCATT | SSR | CE | (CA)26, (CA)7 | 293 | 265 | Echt et al. (this paper) | n/a |  |  |  | F primer was evaluated with a 5' dye-CACGACGTTGTAAAACGAC tail. R primer was evaluated with a 5' GTTTCTT tail. | Locus segregates a null allele. |
| NZPR0599 | 11 | 51.1 | 10 | 0.42 | gb|BV728917 | UniSTS:516222 | TTAATGGAAGGGGGTGGAGT | GAGAGGAGGAATGGGGAAAT | SSR | CE | (TG)6, (AG)18 | 328 | 337 | Echt et al. (this paper) | n/a |  |  |  | F primer was evaluated with a 5' dye-CACGACGTTGTAAAACGAC tail. R primer was evaluated with a 5' GTTTCTT tail. |  |
| NZPR0826 | 3 | 81.6 | 42 | 1.45 | gb|BV728918 | UniSTS:516223 | TCAATATTACCTCGCATATTGAAA | AGGGCATACTCTAAGCCCAA | SSR | CE | (CT)12, (AC)10 | 306 | 314 | Echt et al. (this paper) | n/a |  |  |  | F primer was evaluated with a 5' dye-CACGACGTTGTAAAACGAC tail. R primer was evaluated with a 5' GTTTCTT tail. |  |
| NZPR0917 | 7 | 14.5 | 6 | 0.17 | gb|BV728919 | UniSTS:516224 | CCAATGTTGATCTCTCGCAA | AATACACCCACATCGAGGGA | SSR | CE | (AC)12 | 209 | 225 | Echt et al. (this paper) | n/a |  |  |  | F primer was evaluated with a 5' dye-CACGACGTTGTAAAACGAC tail. R primer was evaluated with a 5' GTTTCTT tail. |  |
| NZPR0933 | 8 | 78.5 | 31 | 16.9, jump>5 | gb|BV728920 | UniSTS:516225 | GGATGCACCCTGAGCTAAAT | GAGGCATCAACCACAAACCT | SSR | CE | (AC)9 | 126 | 127 | Echt et al. (this paper) | n/a |  |  |  | F primer was evaluated with a 5' dye-CACGACGTTGTAAAACGAC tail. R primer was evaluated with a 5' GTTTCTT tail. | Locus segregates a null allele. |
| NZPR0943 | 1 | 73.4 | 23 | 1.09 | gb|BV728921 | UniSTS:516226 | ATTCACCGTCAATCCAAAGC | AAGAGGAGCTCCCATTCCAT | SSR | CE | (AC)10(TA)8 | 231 | 230 | Echt et al. (this paper) | n/a |  |  |  | F primer was evaluated with a 5' dye-CACGACGTTGTAAAACGAC tail. R primer was evaluated with a 5' GTTTCTT tail. |  |
| NZPR0947 | 4 | 88.7 | 16 | 2.44 | gb|BV728922 | UniSTS:516227 | GCAATGCCTATCCCTCTTGA | TGTCCTTGCTTAAATTCCTGC | SSR | CE | (AC)14(TA)5 | 224 | 222 | Echt et al. (this paper) | n/a |  |  |  | F primer was evaluated with a 5' dye-CACGACGTTGTAAAACGAC tail. R primer was evaluated with a 5' GTTTCTT tail. | Locus segregates a null allele. |
| NZPR1004 | 9 | 46.7 | 22 | 0.55 | gb|BV728923 | UniSTS:516228 | GCTTCATGATTTCACGAGCA | AAAGGGACTCTCTCCTTATCACT | SSR | CE | (TG)21, (GT)5 | 219 | 169 | Echt et al. (this paper) | n/a |  |  |  | F primer was evaluated with a 5' dye-CACGACGTTGTAAAACGAC tail. R primer was evaluated with a 5' GTTTCTT tail. |  |
| NZPR1078 | 2 | 107.7 | 17 | 0.61 | gb|BV728924 | UniSTS:516229 | TGGTGATCAAGCCTTTTTCC | GTTGATGAGTGATGGCATGG | SSR | CE | (GT)10 | 342 | 336 | Chagne et al. (2004) | 15448894 |  |  |  | F primer was evaluated with a 5' dye-CACGACGTTGTAAAACGAC tail. R primer was evaluated with a 5' GTTTCTT tail. | Locus segregates a 1bp allele. |
| NZPR1680 | 1 | 54 | 19 | 0.96 | gb|BV728925 | UniSTS:516230 | CACACTCACGCACTCACACA | ATTGGATTGACCCCCTCTTC | SSR | CE | (CA)14 | 215 | 207 | Echt et al. (this paper) | n/a |  |  |  | F primer was evaluated with a 5' dye-CACGACGTTGTAAAACGAC tail. R primer was evaluated with a 5' GTTTCTT tail. |  |
| NZPR1682 | 4 | 72.5 | 13 | 3.03 | gb|BV728926 | UniSTS:516231 | CATTGCCACATCACCAACTC | AAGGATCCCTCGCCACTATT | SSR | CE | (AT)6(TG)21, (TGTA)5 | 243 | 243 | Echt et al. (this paper) | n/a |  |  |  | F primer was evaluated with a 5' dye-CACGACGTTGTAAAACGAC tail. R primer was evaluated with a 5' GTTTCTT tail. |  |
| NZPR1699 | 1 | 45.8 | 13 | 0.91 | gb|BV728927 | UniSTS:516232 | ATGAAGAGAAACCAAAGGTCA | AGCCGATTGAGTGTTTGAGAA | SSR | CE | (TG)13(GA)10 | 286 | 326 | Echt et al. (this paper) | n/a |  |  |  | F primer was evaluated with a 5' dye-CACGACGTTGTAAAACGAC tail. R primer was evaluated with a 5' GTTTCTT tail. |  |
| NZPR1702\_b | 7 | 15.5 | 7 | 0.33 | gb|BV728928 | UniSTS:516233 | TATGATTGGACCATTGGGGT | CCAAACCCTCCTCCACATATC | SSR | CE | (AC)15(CA)13, (AT)5 | 187 | 145 | Chagne et al. (2004) | 15448894 | NZPR1702 |  |  | F primer was evaluated with a 5' dye-CACGACGTTGTAAAACGAC tail. R primer was evaluated with a 5' GTTTCTT tail. |  |
| PbRAMS | 3 | 0.0 | 9 | 0.193 | n/a | n/a | n/a | n/a | ESTP | heteroduplexes | n/a | n/a | n/a | Cato et al. (2001) | n/a | estPbFR\_RAMS(D) |  |  |  |  |
| PpSIFG\_3116 | not mapped | not mapped | not mapped | not mapped | gb|BV728700 | UniSTS:516234 | CAAGTTCGGTTCCTGGTCAT | GTCATATTTCTGGTCCGCGT | SSR | CE | (CGG)5 | 127 | 120 | Echt et al. (this paper) | n/a |  |  |  | F primer was evaluated with a 5' dye-CACGACGTTGTAAAACGAC tail. R primer was evaluated with a 5' GTTTCTT tail. |  |
| PpSIFG\_3129 | 7 | 9.3 | 4 | 0.35 | gb|BV728650 | UniSTS:516235 | AGAAAAACAGCGGCAGAAAA | GCAATCCTCGGCATGTTAAT | SSR | CE | (TTC)4(TCC)5 | 313 | 310 | Echt et al. (this paper) | n/a |  |  |  | F primer was evaluated with a 5' dye-CACGACGTTGTAAAACGAC tail. R primer was evaluated with a 5' GTTTCTT tail. |  |
| PpSIFG\_3145 | 3 | 78.9 | 42 | 0.836 | gb|BV728651 | UniSTS:516236 | TGTATATTCGCCCTGGTGGT | ATCAAATCCAGAATCAGGCG | SSR | CE | (GAG)5(CAG)5(GCA)6 | 372 | 379 | Echt et al. (this paper) | n/a |  |  |  | F primer was evaluated with a 5' dye-CACGACGTTGTAAAACGAC tail. R primer was evaluated with a 5' GTTTCTT tail. |  |
| PpSIFG\_3147 | 6 | 59.4 | 16 | 2.35 | gb|BV728652 | UniSTS:516237 | CACGTGGTTTCCTCCAGTTT | CAATGCGTTCTGCATATTGG | SSR | CE | (AT)9 | 151 | 158 | Echt et al. (this paper) | n/a |  |  |  | F primer was evaluated with a 5' dye-CACGACGTTGTAAAACGAC tail. R primer was evaluated with a 5' GTTTCTT tail. |  |
| PpSIFG\_3168 | not mapped | not mapped | not mapped | not mapped | gb|BV728701 | UniSTS:516238 | ACGGGAGCTGAGCTTAAACA | CAATGCAGCAGAGTGCAAGT | SSR | CE | (TCC)6(AGC)4 | 193 | 173 | Echt et al. (this paper) | n/a |  |  |  | F primer was evaluated with a 5' dye-CACGACGTTGTAAAACGAC tail. R primer was evaluated with a 5' GTTTCTT tail. |  |
| PrCHS1 | 4 | 105 | 8 | 1.357 | n/a | n/a | n/a | n/a | ESTP | heteroduplexes | n/a | n/a | n/a | Cato et al. (2001) | n/a | estPrFR\_CHS1(A) |  |  |  |  |
| PrE79 | 9 | 33.2 | 12 | 1.021 | n/a | n/a | n/a | n/a | ESTP | heteroduplexes | n/a | n/a | n/a | Cato et al. (2001) | n/a | estPrFR\_E79(S) |  |  |  |  |
| PrMADS3 | 5 | 56.1 | 12 | 1.075 | n/a | n/a | n/a | n/a | ESTP | heteroduplexes | n/a | n/a | n/a | Cato et al. (2001) | n/a | estPrFR\_MADS3(D) |  |  |  |  |
| PstASU\_APX | 4 | 106.7 | 13 | 0.653 | gb|BV729014 | UniSTS:516075 | GGCTGCTGGAACCCATCA | GACGTCCATGCACCTTCAAA | ESTP | SSCP | n/a | 304 | n/a | Komulainen et al. (2003) | 12827250 | estPstASU\_APX; estPstUOU\_14; estXUOU\_APX |  |  |  |  |
| PsUME\_Ps3\_A | 1 | 28.6 | 20 | 1.417 | n/a | Pr009397073 | n/a | n/a | RFLP | Southern blot | n/a | 821 | n/a | Sewell et al. (1999) | 9872970 | PtUME\_Ps3\_A |  |  |  |  |
| PsUME\_S43\_63 | 7 | 81.7 | 18 | 0.495 | n/a | n/a | n/a | n/a | RFLP | Southern blot | n/a | n/a | n/a | Sewell et al. (1999) | 9872970 | PtUME\_S43\_63 |  |  |  |  |
| PsUPS2\_PST13 | 10 | not positioned | 3 | not positioned | n/a | n/a | TCCGTTTGACAGGATTGACT | CCCCAGGTCATCCTCTAACT | ESTP | SSCP | n/a | 950 | n/a | Komulainen et al. (2003) | 12827250 | SODchl; sod-chl; CGLSCO\_SODchl\_a |  |  |  |  |
| PsyGPD | 5 | 56.8 | 16 | 2.103 | n/a | n/a | n/a | n/a | ESTP | heteroduplexes | n/a | n/a | n/a | Cato et al. (2001) | n/a | estPsyFR\_GPD(D) |  |  |  |  |
| PtAGP | 4 | 102.9 | 14 | 0.476 | n/a | n/a | n/a | n/a | ESTP | heteroduplexes | n/a | n/a | n/a | Cato et al. (2001) | n/a | estPtFR\_AGP(A) |  |  |  |  |
| PthCAB | 1 | 19 | 7 | 0.413 | n/a | n/a | n/a | n/a | ESTP | heteroduplexes | n/a | n/a | n/a | Cato et al. (2001) | n/a | estPthFR\_CAB(D) |  |  |  |  |
| PitaIFG\_1A7\_6 | 10 | 112.4 | 4 | 0.032 | n/a | n/a | n/a | n/a | RFLP | Southern blot | n/a | 510 | n/a | Eckert et al. (2009) | n/a | PtIFG\_1A7\_6 |  |  |  |  |
| PitaIFG\_2020\_1 | 10 | 96.8 | 5 | 1.322 | n/a | n/a | n/a | n/a | RFLP | Southern blot | n/a | 176 | n/a | Eckert et al. (2009) | n/a | PtIFG\_2020\_1 |  |  |  |  |
| PitaIFG\_2361\_1 | 10 | 102.3 | 4 | 0.110 | n/a | n/a | n/a | n/a | RFLP | Southern blot | n/a | 202 | n/a | Eckert et al. (2009) | n/a | PtIFG\_2361\_1 |  |  |  |  |
| PtIFG\_1165\_a | 2 | 112.5 | 5 | 0.198 | n/a | Pr009397074 | n/a | n/a | RFLP | Southern blot | n/a | 219 | n/a | Devey et al. (1994) | n/a |  |  |  |  |  |
| PtIFG\_138\_A | 5 | 100.2 | 6 | 0.232 | n/a | Pr009397075 | n/a | n/a | RFLP | Southern blot | n/a | 268 | n/a | Sewell et al. (1999) | 9872970 |  |  |  |  |  |
| PtIFG\_138\_B | 3 | 94.7 | 33 | 1.61 | n/a | Pr009397076 | n/a | n/a | RFLP | Southern blot | n/a | 133 | n/a | Sewell et al. (1999) | 9872970 |  |  |  |  |  |
| PtIFG\_1454\_A | 5 | 81.3 | 16 | 0.382 | n/a | Pr009397077 | n/a | n/a | RFLP | Southern blot | n/a | 545 | n/a | Devey et al. (1994) | n/a |  |  |  |  |  |
| PtIFG\_1457\_A | 1 | 69.3 | 16 | 0.69 | n/a | Pr009397078 | n/a | n/a | RFLP | Southern blot | n/a | 338 | n/a | Devey et al. (1994) | n/a |  |  |  |  |  |
| PtIFG\_149\_2 | 8 | 131.6 | 7 | 0.561 | n/a | Pr009397079 | n/a | n/a | RFLP | Southern blot | n/a | 235 | n/a | Sewell et al. (1999) | 9872970 |  |  |  |  |  |
| PtIFG\_149\_A | 7 | 128.5 | 4 | 0.247 | n/a | Pr009397080 | n/a | n/a | RFLP | Southern blot | n/a | 207 | n/a | Sewell et al. (1999) | 9872970 | PtIFG\_149 |  |  |  |  |
| PtIFG\_1588\_A | 11 | 8.7 | 9 | 0.687 | n/a | Pr009397081 | n/a | n/a | RFLP | Southern blot | n/a | 303 | n/a | Devey et al. (1994) | n/a |  |  |  |  |  |
| PtIFG\_1593\_21 | 9 | 48.4 | 19 | 0.438 | n/a | n/a | n/a | n/a | RFLP | Southern blot | n/a | n/a | n/a | Chagne et al. (2003) | n/a |  |  |  |  |  |
| PtIFG\_1599 | 8 | 76 | 15 | 2.1 | n/a | Pr009397082 | n/a | n/a | RFLP | Southern blot | n/a | 206 | n/a | Devey et al. (1994) | n/a |  |  |  |  |  |
| PtIFG\_1623\_A | 2 | 77.5 | 21 | 0.804 | n/a | Pr009397083 | n/a | n/a | RFLP | Southern blot | n/a | 248 | n/a | Devey et al. (1994) | n/a |  |  |  |  |  |
| PtIFG\_1626\_c | 1 | 0.1 | 2 | 0.001 | n/a | Pr009397084 | n/a | n/a | RFLP | Southern blot | n/a | 220 | n/a | Devey et al. (1994) | n/a |  |  |  |  |  |
| PtIFG\_1633\_c | 9 | 74.5 | 10 | 1.343 | n/a | Pr009397085 | n/a | n/a | RFLP | Southern blot | n/a | 263 | n/a | Devey et al. (1994) | n/a |  |  |  |  |  |
| PtIFG\_1635\_A | 10 | 48.2 | 21 | 1.01 | n/a | Pr009397086 | n/a | n/a | RFLP | Southern blot | n/a | 259 | n/a | Sewell et al. (1999) | 9872970 |  |  |  |  |  |
| PtIFG\_1636\_2 | 4 | 111.2 | 10 | 1.018 | n/a | Pr009397087 | n/a | n/a | RFLP | Southern blot | n/a | 257 | n/a | Sewell et al. (1999) | 9872970 |  |  |  |  |  |
| PtIFG\_1636\_3 | 3 | 68.47 | 26 | 7.64, jump>5 | n/a | Pr009397088 | n/a | n/a | RFLP | Southern blot | n/a | 257 | n/a | Sewell et al. (1999) | 9872970 |  |  |  |  |  |
| PtIFG\_1636\_54 | 3 | 55.4 | 27 | 0.804 | n/a | Pr009397089 | n/a | n/a | RFLP | Southern blot | n/a | 257 | n/a | Chagne et al. (2003) | n/a |  |  |  |  |  |
| PtIFG\_1672\_A | 7 | 72.6 | 19 | 1.105 | n/a | Pr009397090 | n/a | n/a | RFLP | Southern blot | n/a | 233 | n/a | Sewell et al. (1999) | 9872970 |  |  |  |  |  |
| PtIFG\_1869\_2 | 10 | 6.3 | 14 | 0.27 | n/a | Pr009397091 | n/a | n/a | RFLP | Southern blot | n/a | 278 | n/a | Sewell et al. (1999) | 9872970 |  |  |  |  |  |
| PtIFG\_1889\_1 | 10 | 14.4 | 16 | 0.339 | n/a | Pr009397092 | n/a | n/a | RFLP | Southern blot | n/a | 229 | n/a | Sewell et al. (1999) | 9872970 |  |  |  |  |  |
| PtIFG\_1902\_1 | 6 | 82.5 | 11 | 3.313 | n/a | Pr009397093 | n/a | n/a | RFLP | Southern blot | n/a | 211 | n/a | Sewell et al. (1999) | 9872970 |  |  |  |  |  |
| PtIFG\_1916\_1 | 11 | 48.4 | 14 | 0.595 | n/a | Pr009397094 | n/a | n/a | RFLP | Southern blot | n/a | 275 | n/a | Sewell et al. (1999) | 9872970 |  |  |  |  |  |
| PtIFG\_1916\_2 | 7 | 60 | 12 | 0.441 | n/a | Pr009397095 | n/a | n/a | RFLP | Southern blot | n/a | 275 | n/a | Sewell et al. (1999) | 9872970 |  |  |  |  |  |
| PtIFG\_1916\_4 | 8 | 62.8 | 11 | 1.907 | n/a | Pr009397096 | n/a | n/a | RFLP | Southern blot | n/a | 275 | n/a | Sewell et al. (1999) | 9872970 |  |  |  |  |  |
| PtIFG\_1917\_A | 8 | 68.4 | 29 | 0.83 | n/a | Pr009397097 | n/a | n/a | RFLP | Southern blot | n/a | 418 | n/a | Devey et al. (1994) | n/a |  |  |  |  |  |
| PtIFG\_1918\_3 | 3 | 54.03 | 19 | 3.20 | n/a | Pr009397098 | n/a | n/a | RFLP | Southern blot | n/a | 249 | n/a | Sewell et al. (1999) | 9872970 |  |  |  |  |  |
| PtIFG\_1918\_A | 6 | 42.5 | 16 | 0.334 | n/a | Pr009397099 | n/a | n/a | RFLP | Southern blot | n/a | 249 | n/a | Devey et al. (1994) | n/a |  |  |  |  |  |
| PtIFG\_1918\_b | 10 | not positioned | 12 | not positioned | n/a | Pr009397100 | n/a | n/a | RFLP | Southern blot | n/a | 249 | n/a | Devey et al. (1994) | n/a |  |  |  |  |  |
| PtIFG\_1918\_f | 10 | 37.3 | 12 | 0.839 | n/a | Pr009397101 | n/a | n/a | RFLP | Southern blot | n/a | 249 | n/a | Devey et al. (1994) | n/a |  |  |  |  |  |
| PtIFG\_1918\_h | 2 | 75.2 | 14 | 3.69, jump>5 | n/a | Pr009397102 | n/a | n/a | RFLP | Southern blot | n/a | 281 | n/a | Devey et al. (1994) | n/a |  |  |  |  |  |
| PtIFG\_1A2\_C | 4 | 75.5 | 10 | 0.288 | n/a | n/a | n/a | n/a | RFLP | Southern blot | n/a | n/a | n/a | Sewell et al. (1999) | 9872970 |  |  |  |  |  |
| PtIFG\_1A7\_A | 2 | 32.8 | 21 | 0.463 | n/a | n/a | n/a | n/a | RFLP | Southern blot | n/a | n/a | n/a | Devey et al. (1994) | n/a |  |  |  |  |  |
| PtIFG\_1D11\_A | 2 | 93.6 | 13 | 1.041 | n/a | n/a | n/a | n/a | RFLP | Southern blot | n/a | n/a | n/a | Devey et al. (1994) | n/a |  |  |  |  |  |
| PtIFG\_1D9\_2 | 3 | 3.69 | 6 | 0.482 | n/a | n/a | n/a | n/a | RFLP | Southern blot | n/a | n/a | n/a | Sewell et al. (1999) | 9872970 |  |  |  |  |  |
| PtIFG\_2006\_C | 3 | 120.4 | 24 | 0.822 | n/a | Pr009397103 | n/a | n/a | RFLP | Southern blot | n/a | 159 | n/a | Sewell et al. (1999) | 9872970 |  |  |  |  |  |
| PtIFG\_2009\_A | 6 | 108.9 | 19 | 0.667 | n/a | n/a | n/a | n/a | RFLP | Southern blot | n/a | n/a | n/a | Sewell et al. (1999) | 9872970 |  |  |  |  |  |
| PtIFG\_2022\_A | 5 | 54.2 | 16 | 1.17 | n/a | Pr009397104 | n/a | n/a | RFLP | Southern blot | n/a | 974 | n/a | Devey et al. (1994) | n/a |  |  |  |  |  |
| PtIFG\_2068\_A | 3 | 112.1 | 18 | 0.780 | n/a | Pr009397105 | n/a | n/a | RFLP | Southern blot | n/a | 382 | n/a | Devey et al. (1994) | n/a |  |  |  |  |  |
| PtIFG\_2086\_13 | 1 | 0.0 | 10 | 0.449 | n/a | n/a | n/a | n/a | RFLP | Southern blot | n/a | n/a | n/a | Brown et al. (2001) | 11606554 |  |  |  |  |  |
| PtIFG\_2086\_2 | 11 | 18.1 | 12 | 1.166 | n/a | n/a | n/a | n/a | RFLP | Southern blot | n/a | n/a | n/a | Brown et al. (2001) | 11606554 |  |  |  |  |  |
| PtIFG\_2090\_1 | 6 | 76.9 | 15 | 0.612 | n/a | Pr009397106 | n/a | n/a | RFLP | Southern blot | n/a | 206 | n/a | Sewell et al. (1999) | 9872970 |  |  |  |  |  |
| PtIFG\_2090\_2 | 3 | 49.9 | 26 | 0.723 | n/a | Pr009397107 | n/a | n/a | RFLP | Southern blot | n/a | 206 | n/a | Sewell et al. (1999) | 9872970 |  |  |  |  |  |
| PtIFG\_2090\_4 | 5 | 26.1 | 19 | 0.734 | n/a | Pr009397108 | n/a | n/a | RFLP | Southern blot | n/a | 206 | n/a | Sewell et al. (1999) | 9872970 |  |  |  |  |  |
| PtIFG\_2113\_1 | 4 | 26.7 | 11 | 0.875 | n/a | Pr009397109 | n/a | n/a | RFLP | Southern blot | n/a | 133 | n/a | Sewell et al. (1999) | 9872970 |  |  |  |  |  |
| PtIFG\_2145\_1 | 3 | 115.1 | 6 | 0.453 | n/a | Pr009397110 | n/a | n/a | RFLP | Southern blot | n/a | 156 | n/a | Sewell et al. (1999) | 9872970 |  |  |  |  |  |
| PtIFG\_2145\_28 | 10 | 31.3 | 18 | 0.277 | n/a | Pr009397111 | n/a | n/a | RFLP | Southern blot | n/a | 156 | n/a | Sewell et al. (1999) | 9872970 |  |  |  |  |  |
| PtIFG\_2145\_3 | 4 | 0.0 | 6 | 1.021 | n/a | Pr009397112 | n/a | n/a | RFLP | Southern blot | n/a | 156 | n/a | Sewell et al. (1999) | 9872970 |  |  |  |  |  |
| PtIFG\_2145\_76 | 3 | 9.13 | 15 | 0.418 | n/a | Pr009397113 | n/a | n/a | RFLP | Southern blot | n/a | 156 | n/a | Chagne et al. (2003) | n/a |  |  |  |  |  |
| PtIFG\_2146\_2 | 1 | 75.9 | 26 | 1.692 | n/a | n/a | n/a | n/a | RFLP | Southern blot | n/a | n/a | n/a | Sewell et al. (1999) | 9872970 |  |  |  |  |  |
| PtIFG\_2150\_A | 2 | 12.5 | 12 | 0.35 | n/a | Pr009397114 | n/a | n/a | RFLP | Southern blot | n/a | 171 | n/a | Devey et al. (1994) | n/a |  |  |  |  |  |
| PtIFG\_2197\_1 | 11 | 95.7 | 8 | 0.68 | n/a | n/a | n/a | n/a | RFLP | Southern blot | n/a | n/a | n/a | Sewell et al. (1999) | 9872970 |  |  |  |  |  |
| PtIFG\_2220\_A | 5 | 90 | 33 | 1.575 | n/a | n/a | n/a | n/a | RFLP | Southern blot | n/a | n/a | n/a | Devey et al. (1994) | n/a |  |  |  |  |  |
| PtIFG\_2220\_B | 4 | 124.3 | 9 | 1.435 | n/a | n/a | n/a | n/a | RFLP | Southern blot | n/a | n/a | n/a | Devey et al. (1994) | n/a |  |  |  |  |  |
| PtIFG\_2253\_A | 1 | 47.9 | 33 | 2.838 | n/a | Pr009397115 | n/a | n/a | RFLP | Southern blot | n/a | 187 | n/a | Sewell et al. (1999) | 9872970 |  |  |  |  |  |
| PtIFG\_2291\_A | 6 | 29.4 | 13 | 0.415 | n/a | Pr009397116 | n/a | n/a | RFLP | Southern blot | n/a | 274 | n/a | Sewell et al. (1999) | 9872970 |  |  |  |  |  |
| PtIFG\_2295\_2 | 5 | 110.4 | 8 | 1.387 | n/a | Pr009397117 | n/a | n/a | RFLP | Southern blot | n/a | 226 | n/a | Sewell et al. (1999) | 9872970 |  |  |  |  |  |
| PtIFG\_2323\_A | 9 | 27.9 | 16 | 0.252 | n/a | Pr009397118 | n/a | n/a | RFLP | Southern blot | n/a | 246 | n/a | Sewell et al. (1999) | 9872970 |  |  |  |  |  |
| PtIFG\_2361\_2 | 7 | 80.7 | 12 | 0.801 | n/a | Pr009397119 | n/a | n/a | RFLP | Southern blot | n/a | 202 | n/a | Sewell et al. (1999) | 9872970 |  |  |  |  |  |
| PtIFG\_2393\_1 | 1 | 102.4 | 16 | 0.719 | n/a | Pr009397120 | n/a | n/a | RFLP | Southern blot | n/a | 108 | n/a | Sewell et al. (1999) | 9872970 |  |  |  |  |  |
| PtIFG\_2413\_b | 7 | 100.6 | 8 | 0.918 | n/a | n/a | n/a | n/a | RFLP | Southern blot | n/a | n/a | n/a | Sewell et al. (1999) | 9872970 |  |  |  |  |  |
| PtIFG\_2441\_1 | 1 | 95.2 | 16 | 0.428 | n/a | n/a | n/a | n/a | RFLP | Southern blot | n/a | n/a | n/a | Sewell et al. (1999) | 9872970 |  |  |  |  |  |
| PtIFG\_2479\_1 | 9 | 15 | 11 | 0.246 | n/a | n/a | n/a | n/a | RFLP | Southern blot | n/a | n/a | n/a | Sewell et al. (1999) | 9872970 |  |  |  |  |  |
| PtIFG\_2530\_A | 4 | 43.5 | 8 | 0.941 | n/a | Pr009397121 | n/a | n/a | RFLP | Southern blot | n/a | 205 | n/a | Devey et al. (1994) | n/a |  |  |  |  |  |
| PtIFG\_2538\_5 | 8 | 131.2 | 6 | 0.456 | n/a | Pr009397122 | n/a | n/a | RFLP | Southern blot | n/a | 260 | n/a | Sewell et al. (1999) | 9872970 |  |  |  |  |  |
| PtIFG\_2538\_B | 2 | 21.7 | 25 | 0.26 | n/a | Pr009397123 | n/a | n/a | RFLP | Southern blot | n/a | 195 | n/a | Devey et al. (1994) | n/a |  |  |  |  |  |
| PtIFG\_2564\_A | 2 | 33.2 | 20 | 0.7 | n/a | Pr009397124 | n/a | n/a | RFLP | Southern blot | n/a | 226 | n/a | Devey et al. (1994) | n/a |  |  |  |  |  |
| PtIFG\_2564\_B | 4 | 52.2 | 19 | 1.157 | n/a | Pr009397125 | n/a | n/a | RFLP | Southern blot | n/a | 211 | n/a | Devey et al. (1994) | n/a |  |  |  |  |  |
| PtIFG\_2568\_A | 8 | 94.4 | 33 | 2.612 | n/a | Pr009397126 | n/a | n/a | RFLP | Southern blot | n/a | 208 | n/a | Devey et al. (1994) | n/a |  |  |  |  |  |
| PtIFG\_2574\_2 | 5 | 107.3 | 9 | 0.649 | n/a | Pr009397127 | n/a | n/a | RFLP | Southern blot | n/a | 184 | n/a | Sewell et al. (1999) | 9872970 |  |  |  |  |  |
| PtIFG\_2574\_c | 5 | 99.1 | 11 | 0.218 | n/a | Pr009397128 | n/a | n/a | RFLP | Southern blot | n/a | 239 | n/a | Devey et al. (1994) | n/a |  |  |  |  |  |
| PtIFG\_2588\_1 | 3 | 86.46 | 28 | 1.50 | n/a | Pr009397129 | n/a | n/a | RFLP | Southern blot | n/a | 254 | n/a | Sewell et al. (1999) | 9872970 |  |  |  |  |  |
| PtIFG\_2615\_1 | 11 | 7.1 | 17 | 0.642 | n/a | Pr009397130 | n/a | n/a | RFLP | Southern blot | n/a | 147 | n/a | Sewell et al. (1999) | 9872970 |  |  |  |  |  |
| PtIFG\_2697\_A | 1 | 11.6 | 17 | 0.336 | n/a | Pr009397131 | n/a | n/a | RFLP | Southern blot | n/a | 215 | n/a | Devey et al. (1994) | n/a |  |  |  |  |  |
| PtIFG\_2718\_1 | 3 | 30.9 | 12 | 4.87 | n/a | Pr009397132 | n/a | n/a | RFLP | Southern blot | n/a | 199 | n/a | Devey et al. (1994) | n/a |  |  |  |  |  |
| PtIFG\_2718\_2 | 4 | 77.8 | 10 | 3.111 | n/a | Pr009397133 | n/a | n/a | RFLP | Southern blot | n/a | 199 | n/a | Sewell et al. (1999) | 9872970 |  |  |  |  |  |
| PtIFG\_2718\_3 | 3 | 74.5 | 23 | 1.38 | n/a | Pr009397134 | n/a | n/a | RFLP | Southern blot | n/a | 199 | n/a | Sewell et al. (1999) | 9872970 |  |  |  |  |  |
| PtIFG\_2723\_1 | 12 | 60.5 | 12 | 1.152 | n/a | n/a | n/a | n/a | RFLP | Southern blot | n/a | n/a | n/a | Sewell et al. (1999) | 9872970 |  |  |  |  |  |
| PtIFG\_2723\_Aa | 6 | 65.5 | 5 | 0.261 | n/a | n/a | n/a | n/a | RFLP | Southern blot | n/a | n/a | n/a | Devey et al. (1994) | n/a |  |  |  |  |  |
| PtIFG\_2738\_B | 8 | 121 | 13 | 1.027 | n/a | n/a | n/a | n/a | RFLP | Southern blot | n/a | n/a | n/a | Devey et al. (1994) | n/a |  |  |  |  |  |
| PtIFG\_2745\_1 | 3 | 63.6 | 22 | 0.930 | n/a | n/a | n/a | n/a | RFLP | Southern blot | n/a | n/a | n/a | Sewell et al. (1999) | 9872970 |  |  |  |  |  |
| PtIFG\_2782\_2 | 5 | 79 | 18 | 1.822 | n/a | n/a | n/a | n/a | RFLP | Southern blot | n/a | n/a | n/a | Sewell et al. (1999) | 9872970 |  |  |  |  |  |
| PtIFG\_2782\_31 | 1 | 58.4 | 28 | 2.266 | n/a | n/a | n/a | n/a | RFLP | Southern blot | n/a | n/a | n/a | Chagne et al. (2003) | n/a |  |  |  |  |  |
| PtIFG\_2802\_3 | 6 | 16.3 | 7 | 0.139 | n/a | n/a | n/a | n/a | RFLP | Southern blot | n/a | n/a | n/a | Sewell et al. (1999) | 9872970 |  |  |  |  |  |
| PtIFG\_2885\_1 | 2 | 10.5 | 13 | 0.276 | n/a | n/a | n/a | n/a | RFLP | Southern blot | n/a | n/a | n/a | Sewell et al. (1999) | 9872970 |  |  |  |  |  |
| PtIFG\_2897\_d | 3 | 108.8 | 19 | 0.377 | n/a | Pr009397136 | n/a | n/a | RFLP | Southern blot | n/a | 314 | n/a | Sewell et al. (1999) | 9872970 |  |  |  |  |  |
| PtIFG\_2899\_A | 9 | 116.3 | 12 | 0.28 | n/a | Pr009397137 | n/a | n/a | RFLP | Southern blot | n/a | 273 | n/a | Sewell et al. (1999) | 9872970 |  |  |  |  |  |
| PtIFG\_2931\_A | 1 | 103.4 | 18 | 1.756 | n/a | Pr009397138 | n/a | n/a | RFLP | Southern blot | n/a | 239 | n/a | Sewell et al. (1999) | 9872970 |  |  |  |  |  |
| PtIFG\_2933\_12 | 5 | 44.7 | 26 | 1.127 | n/a | n/a | n/a | n/a | RFLP | Southern blot | n/a | n/a | n/a | Chagne et al. (2003) | n/a |  |  |  |  |  |
| PtIFG\_2957\_A | 8 | 21.3 | 16 | 1.453 | n/a | n/a | n/a | n/a | RFLP | Southern blot | n/a | n/a | n/a | Sewell et al. (1999) | 9872970 |  |  |  |  |  |
| PtIFG\_2963\_1 | 11 | 53.1 | 12 | 0.841 | n/a | n/a | n/a | n/a | RFLP | Southern blot | n/a | n/a | n/a | Sewell et al. (1999) | 9872970 |  |  |  |  |  |
| PtIFG\_2963\_3 | 5 | 0.0 | 9 | 0.967 | n/a | n/a | n/a | n/a | RFLP | Southern blot | n/a | n/a | n/a | Sewell et al. (1999) | 9872970 |  |  |  |  |  |
| PtIFG\_2969\_1 | 11 | 32.8 | 13 | 0.607 | n/a | n/a | n/a | n/a | RFLP | Southern blot | n/a | n/a | n/a | Sewell et al. (1999) | 9872970 |  |  |  |  |  |
| PtIFG\_2986\_A | 2 | 92.3 | 21 | 1.012 | n/a | n/a | n/a | n/a | RFLP | Southern blot | n/a | n/a | n/a | Sewell et al. (1999) | 9872970 | PtIFG\_29B6\_A |  |  |  |  |
| PtIFG\_2986\_B | 11 | 10 | 12 | 1.267 | n/a | n/a | n/a | n/a | RFLP | Southern blot | n/a | n/a | n/a | Sewell et al. (1999) | 9872970 |  |  |  |  |  |
| PtIFG\_2988\_21 | 3 | 34.1 | 31 | 1.39 | n/a | n/a | n/a | n/a | RFLP | Southern blot | n/a | n/a | n/a | Chagne et al. (2003) | n/a |  |  |  |  |  |
| PtIFG\_3006\_1 | 2 | 71 | 11 | 1.558 | n/a | n/a | n/a | n/a | RFLP | Southern blot | n/a | n/a | n/a | Sewell et al. (1999) | 9872970 |  |  |  |  |  |
| PtIFG\_3008\_1 | 8 | 50 | 22 | 0.54 | n/a | Pr009397139 | n/a | n/a | RFLP | Southern blot | n/a | 296 | n/a | Sewell et al. (1999) | 9872970 |  |  |  |  |  |
| PtIFG\_3012\_2 | 12 | 86.7 | 11 | 1.064 | n/a | Pr009397140 | n/a | n/a | RFLP | Southern blot | n/a | 256 | n/a | Sewell et al. (1999) | 9872970 |  |  |  |  |  |
| PtIFG\_3012\_3 | 2 | 2.7 | 12 | 0.709 | n/a | Pr009397141 | n/a | n/a | RFLP | Southern blot | n/a | 248 | n/a | Sewell et al. (1999) | 9872970 | PtIFG\_3012\_43 |  |  |  |  |
| PtIFG\_3021\_1 | 10 | not positioned | 5 | not positioned | n/a | Pr009397142 | n/a | n/a | RFLP | Southern blot | n/a | 219 | n/a | Sewell et al. (1999) | 9872970 |  |  |  |  |  |
| PtIFG\_3026\_A | 5 | 106.4 | 7 | 0.368 | n/a | n/a | n/a | n/a | RFLP | Southern blot | n/a | n/a | n/a | Sewell et al. (1999) | 9872970 |  |  |  |  |  |
| PtIFG\_459\_1 | 9 | 70.6 | 16 | 0.401 | n/a | n/a | n/a | n/a | RFLP | Southern blot | n/a | n/a | n/a | Sewell et al. (1999) | 9872970 |  |  |  |  |  |
| PtIFG\_4D4\_A | 6 | 91.3 | 6 | 5.036 | n/a | n/a | n/a | n/a | RFLP | Southern blot | n/a | n/a | n/a | Devey et al. (1994) | n/a |  |  |  |  |  |
| PtIFG\_503\_A | 10 | 26.2 | 13 | 0.985 | n/a | Pr009397143 | n/a | n/a | RFLP | Southern blot | n/a | 238 | n/a | Sewell et al. (1999) | 9872970 |  |  |  |  |  |
| PtIFG\_606\_1 | 6 | 98.8 | 11 | 0.611 | n/a | n/a | n/a | n/a | RFLP | Southern blot | n/a | n/a | n/a | Sewell et al. (1999) | 9872970 |  |  |  |  |  |
| PtIFG\_616 | 8 | 116.4 | 17 | 1.327 | n/a | Pr009397144 | n/a | n/a | RFLP | Southern blot | n/a | 515 | n/a | Devey et al. (1994) | n/a |  |  |  |  |  |
| PtIFG\_653\_2 | 12 | 89 | 10 | 2.452 | n/a | Pr009397145 | n/a | n/a | RFLP | Southern blot | n/a | 226 | n/a | Sewell et al. (1999) | 9872970 |  |  |  |  |  |
| PtIFG\_653\_3 | 11 | 80.7 | 11 | 0.266 | n/a | Pr009397146 | n/a | n/a | RFLP | Southern blot | n/a | 226 | n/a | Sewell et al. (1999) | 9872970 |  |  |  |  |  |
| PtIFG\_653\_d | 1 | 1.7 | 3 | 0.156 | n/a | Pr009397147 | n/a | n/a | RFLP | Southern blot | n/a | 226 | n/a | Devey et al. (1994) | n/a |  |  |  |  |  |
| PtIFG\_658\_A | 1 | 79.9 | 30 | 0.837 | n/a | n/a | n/a | n/a | RFLP | Southern blot | n/a | n/a | n/a | Devey et al. (1994) | n/a |  |  |  |  |  |
| PtIFG\_66\_1 | 2 | 77.9 | 10 | 1.239 | n/a | Pr009397148 | n/a | n/a | RFLP | Southern blot | n/a | 227 | n/a | Sewell et al. (1999) | 9872970 |  |  |  |  |  |
| PtIFG\_669 | 9 | 40.4 | 20 | 0.252 | n/a | Pr009397149 | n/a | n/a | RFLP | Southern blot | n/a | 185 | n/a | Devey et al. (1994) | n/a | PtIFG\_669\_b; PtIFG\_669\_c |  |  |  |  |
| PtIFG\_719\_3 | 11 | 68.1 | 11 | 0.337 | n/a | n/a | n/a | n/a | RFLP | Southern blot | n/a | n/a | n/a | Sewell et al. (1999) | 9872970 |  |  |  |  |  |
| PtIFG\_719\_A | 8 | 52.7 | 24 | 0.71 | n/a | n/a | n/a | n/a | RFLP | Southern blot | n/a | n/a | n/a | Sewell et al. (1999) | 9872970 |  |  |  |  |  |
| PtIFG\_846 | 8 | 13.5 | 8 | 1.026 | n/a | Pr009397150 | n/a | n/a | RFLP | Southern blot | n/a | 562 | n/a | Devey et al. (1994) | n/a | PtIFG\_846\_a |  |  |  |  |
| PtIFG\_851\_1 | 1 | 122.1 | 9 | 1.205 | n/a | n/a | n/a | n/a | RFLP | Southern blot | n/a | n/a | n/a | Sewell et al. (1999) | 9872970 |  |  |  |  |  |
| PtIFG\_975\_3 | 3 | 108.6 | 20 | 1.17 | n/a | Pr009397151 | n/a | n/a | RFLP | Southern blot | n/a | 483 | n/a | Sewell et al. (1999) | 9872970 |  |  |  |  |  |
| PtIFG\_975\_4 | 4 | 5.8 | 5 | 1.066 | n/a | Pr009397152 | n/a | n/a | RFLP | Southern blot | n/a | 483 | n/a | Sewell et al. (1999) | 9872970 |  |  |  |  |  |
| estPtIFG\_dhn-1 | 8 | 26.4 | 13 | 0.566 | n/a | n/a | ATCTGCACTCGCTCTTTGAT | TAAGCAAATCCCTGAAGGAG | ESTP | DGGE | n/a | n/a | n/a | Gonzalez-Martinez et al. (2006) | 16387885 | estPtIFG\_F4R5(dhn) |  |  |  |  |
| PtIPST\_pLP2 | 3 | 2.51 | 9 | 0.272 | n/a | n/a | n/a | n/a | ESTP | SSCP | n/a | n/a | n/a | Komulainen et al. (2003) | 12827250 | estPtIPST\_LP2; estPtIPST\_\_pLP2; estPtIPST\_pLP2\_a |  |  |  |  |
| PtLP15 | 6 | 85.3 | 18 | 0.87 | n/a | n/a | n/a | n/a | ESTP | heteroduplexes | n/a | n/a | n/a | Cato et al. (2001) | n/a | estPtX\_LP15(A) |  |  |  |  |
| PtLP3-1 | 2 | 67.4 | 11 | 4.32, jump>5 | n/a | n/a | n/a | n/a | ESTP | heteroduplexes | n/a | n/a | n/a | Cato et al. (2001) | n/a | estPtX\_LP3-1(S); estPtIFG\_LP3-1 |  |  |  |  |
| PtMTU\_lpPAL | 6 | 44.4 | 9 | 1.064 | gb|BV729055 | UniSTS:516061 | TAGCCAAGAAAACCCTGAG | ACTGATAGCGTCGTAAACCA | ESTP | DGGE | n/a | 496 | n/a | Brown et al. (2003) | 12930758 | estPtINR\_PAL-1; estPtINR\_1\_a; PALI-F3+R3-NlaIII | Primer sequences obtained from http://www.pierroton.inra.fr/genetics/pinus/primers.html. |  |  |  |
| PtNCS\_1CA4G | 8 | 27.1 | 19 | 0.389 | n/a | n/a | TAAATGAGGTGCTCTTACAA | GCAAACTTCTAGCCACTTA | ESTP | SSCP | n/a | n/a | n/a | Komulainen et al. (2003) | 12827250 | estXUOU\_thymsynt |  |  |  |  |
| PtNCS\_3H6z5\_A | 4 | 103.7 | 25 | 4.14 | n/a | n/a | n/a | n/a | RFLP | Southern blot | n/a | n/a | n/a | Sewell et al. (1999) | 9872970 |  |  |  |  |  |
| PtNCS\_HLH1 | 8 | 0.7 | 8 | 1.314 | gb|BV728993 | UniSTS:516072 | ACAGTTTTGGCACCTCTCA | ATTCTTTTGCACGCTTTCTT | ESTP | SSCP | n/a | 851 | n/a | Komulainen et al. (2003) | 12827250 | estPtNCS\_HLH1 |  |  |  |  |
| PtNCS\_p9myb1\_21 | 7 | 64.7 | 8 | 0.278 | n/a | Pr009397153 | n/a | n/a | RFLP | Southern blot | n/a | 461 | n/a | Sewell et al. (1999) | 9872970 |  |  |  |  |  |
| PtNCS\_PtaAGP6 | 5 | 9.8 | 12 | 1.663 | gb|BV728991 | UniSTS:516070 | TCAGGGTCAACAATGGCGTTC | GGGCTTTTCAGTGCGGACG | ESTP | DGGE | n/a | 560 | n/a | Brown et al. (2003) | 12930758 | estPtNCS\_AGP6-3p; AGP6-F1; PtNCS\_PtaAGP6\_1 |  |  |  |  |
| PtNCS\_ptCadA | 9 | 108.96 | 14 | 0.539 | gb|BV728994 | UniSTS:516073 | ACGTGACGGTTATCAGTTC | AAGACTTGCCATTGGATTA | ESTP | SSCP | n/a | 686 | n/a | Komulainen et al. (2003) | 12827250 | estPtNCS\_ptCadA; PtNCS\_CAD-08\_A |  |  |  |  |
| PtRIP\_0022 | 7 | 104.4 | 7 | 0.19 | gb|BV683041 | UniSTS:513453 | CTCAGTTTCATAATCTTTGTCGC | TTTTAGAAAAGAAGGAAATCTTCA | SSR | CE | (ACC)6(TCA)4 | 250 | 248 | Echt et al. (this paper) | n/a |  |  |  | F primer was evaluated with a 5' dye-CACGACGTTGTAAAACGAC tail. R primer was evaluated with a 5' GTTTCTT tail. | Locus segregates a null allele. |
| PtRIP\_0032 | 7 | 74.3 | 13 | 0.55 | gb|BV683044 | UniSTS:513456 | TAGCAGGTTACAACCTGGGG | AGCCCAATTGATGGGAAATT | SSR | CE | (TAT)7 | 188 | 184 | Echt et al. (this paper) | n/a |  |  |  | F primer was evaluated with a 5' dye-CACGACGTTGTAAAACGAC tail. R primer was evaluated with a 5' GTTTCTT tail. | Locus segregates a null allele. |
| PtRIP\_0064 | 1 | 95.6 | 24 | 0.52 | gb|BV683046 | UniSTS:513458 | GCAGCGTAATCAGATGGTCA | CGGAAGGCGAGTTGAAGATA | SSR | CE | (A)6, (A)5(AAAC)5(A)5 | 258 | 265 | Echt et al. (this paper) | n/a |  |  |  | F primer was evaluated with a 5' dye-CACGACGTTGTAAAACGAC tail. R primer was evaluated with a 5' GTTTCTT tail. | Locus segregates a null allele. |
| PtRIP\_0065 | 2 | 6.4 | 19 | 0.47 | gb|BV683047 | UniSTS:513459 | CCAACAGCACTTACCCAAAA | AGCCTCATGAAAGCCCAGTA | SSR | CE | (AAAC)5(A)7 | 142 | 131 | Echt et al. (this paper) | n/a |  |  |  | F primer was evaluated with a 5' dye-CACGACGTTGTAAAACGAC tail. R primer was evaluated with a 5' GTTTCTT tail. | Locus segregates a 1bp allele. |
| PtRIP\_0066 | 7 | 62.1 | 10 | 0.92 | gb|BV683048 | UniSTS:513460 | GTTGATAGAGTTTCATGTGGTGC | TGGATGAAGAATTTTGTAGTCAA | SSR | CE | (AAAT)8 | 114 | 94 | Echt et al. (this paper) | n/a |  |  |  | F primer was evaluated with a 5' dye-CACGACGTTGTAAAACGAC tail. R primer was evaluated with a 5' GTTTCTT tail. | Locus segregates a null allele. |
| PtRIP\_0067 | 5 | 41.4 | 33 | 1.3 | gb|BV683049 | UniSTS:513461 | AGCCCTCCAAGACCAAGATT | CCATTTGCAAATACCCCAAC | SSR | CE | (AAAT)4 | 227 | 223 | Echt et al. (this paper) | n/a |  |  |  | F primer was evaluated with a 5' dye-CACGACGTTGTAAAACGAC tail. R primer was evaluated with a 5' GTTTCTT tail. |  |
| PtRIP\_0079 | 12 | 43.5 | 15 | 2.65, jump>5 | gb|BV683053 | UniSTS:513465 | TGATTTGATCCCTCTAGGCG | AATCTTGAAAAGAAATTCAATATGAGA | SSR | CE | (ATT)12 | 153 | 131 | Echt et al. (this paper) | n/a |  |  |  | F primer was evaluated with a 5' dye-CACGACGTTGTAAAACGAC tail. R primer was evaluated with a 5' GTTTCTT tail. |  |
| PtRIP\_0103 | 7 | 106.2 | 10 | 0.26 | gb|BV683057 | UniSTS:513469 | CCCCTTGGTGGAACAACATA | TTGGAAAATGGCGGAATTTA | SSR | CE | (TG)26, (AG)6 | 210 | 181 | Echt et al. (this paper) | n/a |  |  |  | F primer was evaluated with a 5' dye-CACGACGTTGTAAAACGAC tail. R primer was evaluated with a 5' GTTTCTT tail. | Locus segregates a null allele, and 1bp allele. |
| PtRIP\_0106 | 11 | 90.1 | 7 | 0.19 | gb|BV683059 | UniSTS:513471 | ATCAGATTGGTGGATCGGAG | TGACTGATAAGGGTTTCGCC | SSR | CE | (AT)7, (TA)8(TG)11, (GT)9 | 180 | 168 | Echt et al. (this paper) | n/a |  |  |  | F primer was evaluated with a 5' dye-CACGACGTTGTAAAACGAC tail. R primer was evaluated with a 5' GTTTCTT tail. |  |
| PtRIP\_0117 | 12 | 10 | 10 | 0.96 | gb|BV683060 | UniSTS:513472 | GCTTCATGATTTCTCGATCG | TCTGCGTGGATAAAGGAATTT | SSR | CE | (AC)12 | 208 | 229 | Echt et al. (this paper) | n/a |  |  |  | F primer was evaluated with a 5' dye-CACGACGTTGTAAAACGAC tail. R primer was evaluated with a 5' GTTTCTT tail. | Locus segregates a null allele. |
| PtRIP\_0126 | 3 | 59.6 | 37 | 1.01 | gb|BV683062 | UniSTS:513474 | TCATACCGAGAGAGGTCTTTG | GAGCTTAATTTGTGCCTGCC | SSR | CE | (TG)12, (TG)5 | 174 | 165 | Echt et al. (this paper) | n/a |  |  |  | F primer was evaluated with a 5' dye-CACGACGTTGTAAAACGAC tail. R primer was evaluated with a 5' GTTTCTT tail. |  |
| PtRIP\_0134 | 3 | 19.6 | 34 | 1.42 | gb|BV683065 | UniSTS:513477 | GTTTACATTTTCCTGGGGCA | GATTTACAAAAATCCCTGCCA | SSR | CE | (AC)15 | 145 | 139 | Echt et al. (this paper) | n/a |  |  |  | F primer was evaluated with a 5' dye-CACGACGTTGTAAAACGAC tail. R primer was evaluated with a 5' GTTTCTT tail. |  |
| PtRIP\_0135 | 10 | 46.6 | 20 | 0.277 | gb|BV683066 | UniSTS:513478 | CACGCATGAGCTGAGTCATAA | TGTGTTTCCCACTATGCTAAGC | SSR | CE | (TG)41 | 218 | 202 | Echt et al. (this paper) | n/a |  |  |  | F primer was evaluated with a 5' dye-CACGACGTTGTAAAACGAC tail. R primer was evaluated with a 5' GTTTCTT tail. |  |
| PtRIP\_0158 | 1 | 105.9 | 21 | 1.48 | gb|BV683068 | UniSTS:513480 | GTGTGCCACGGATGTATGAG | TTGCTGAAAGGGCCAGTAGT | SSR | CE | (AT)7(TG)13 | 211 | 212 | Echt et al. (this paper) | n/a |  |  |  | F primer was evaluated with a 5' dye-CACGACGTTGTAAAACGAC tail. R primer was evaluated with a 5' GTTTCTT tail. |  |
| PtRIP\_0165 | 10 | 63.4 | 16 | 1.295 | gb|BV683070 | UniSTS:513482 | TGGAAGCCACAATTTGTTGA | TGCAATAAAACCATGCAACAA | SSR | CE | (TC)27(AC)11 | 220 | 202 | Echt et al. (this paper) | n/a |  |  |  | F primer was evaluated with a 5' dye-CACGACGTTGTAAAACGAC tail. R primer was evaluated with a 5' GTTTCTT tail. |  |
| PtRIP\_0171 | 10 | 35.7 | 23 | 16.1 | gb|BV683072 | UniSTS:513484 | TGATCCTAAGCCTTAGAAACCC | TTTTGTCACCCATGCATATGA | SSR | CE | (TG)15 | 207 | 197 | Echt et al. (this paper) | n/a |  |  |  | F primer was evaluated with a 5' dye-CACGACGTTGTAAAACGAC tail. R primer was evaluated with a 5' GTTTCTT tail. |  |
| PtRIP\_0179 | 3 | 30.4 | 21 | 12.5, jump>5 | gb|BV683073 | UniSTS:513485 | TGTAGGAGCACAAGCCATTG | AACACAGTTGGACCGTTTGA | SSR | CE | (ATT)8 | 170 | 166 | Echt et al. (this paper) | n/a |  |  |  | F primer was evaluated with a 5' dye-CACGACGTTGTAAAACGAC tail. R primer was evaluated with a 5' GTTTCTT tail. | Locus segregates a 1bp allele. |
| PtRIP\_0211 | 1 | 17.7 | 18 | 0.5 | gb|BV683076 | UniSTS:513488 | GAGGGGGTCTCATACACCAA | TGCATAGAGGATGTATTTCTTGGA | SSR | CE | (ATA)13 | 159 | 142 | Echt et al. (this paper) | n/a |  |  |  | F primer was evaluated with a 5' dye-CACGACGTTGTAAAACGAC tail. R primer was evaluated with a 5' GTTTCTT tail. | Locus segregates a null allele. |
| PtRIP\_0255 | 10 | 117.9 | 4 | 5.477 | gb|BV683147 | UniSTS:513489 | TCCTCCTGAGTGGTCCCATA | TATGGATATGAGGCCTGTTGG | SSR | CE | (AAT)8, (AAAAT)5 | 123 | 132 | Echt et al. (this paper) | n/a |  |  |  | F primer was evaluated with a 5' dye-CACGACGTTGTAAAACGAC tail. R primer was evaluated with a 5' GTTTCTT tail. |  |
| PtRIP\_0263 | 11 | 30.8 | 11 | 1.31 | gb|BV683148 | UniSTS:513490 | TTGGATTGGACCTGAATCAA | TTGGCAGTCTTCGAGGTCTT | SSR | CE | (AAAT)6 | 183 | 154 | Echt et al. (this paper) | n/a |  |  |  | F primer was evaluated with a 5' dye-CACGACGTTGTAAAACGAC tail. R primer was evaluated with a 5' GTTTCTT tail. |  |
| PtRIP\_0287 | 11 | 0.5 | 15 | 1.33 | gb|BV683077 | UniSTS:513492 | GGAATGTATTCCCGGTTCCT | CTCCCGGATATTGAGGAGGT | SSR | CE | (TTTA)5 | 224 | 218 | Echt et al. (this paper) | n/a |  |  |  | F primer was evaluated with a 5' dye-CACGACGTTGTAAAACGAC tail. R primer was evaluated with a 5' GTTTCTT tail. |  |
| PtRIP\_0305 | 10 | 25.0 | 20 | 0.406 | gb|BV683080 | UniSTS:513495 | TCAATCACCAATTATTTGGCT | GGAGTGGATGAAACTATGCCA | SSR | CE | (TTC)6, (CTC)6 | 230 | 226 | Echt et al. (this paper) | n/a |  |  |  | F primer was evaluated with a 5' dye-CACGACGTTGTAAAACGAC tail. R primer was evaluated with a 5' GTTTCTT tail. | Locus segregates a 1bp allele. |
| PtRIP\_0367 | 1 | 61.7 | 27 | 1.6 | gb|BV683081 | UniSTS:513496 | CCAATGCATAATGCAACCAC | TAGCCATGGTGCTCAGTCTG | SSR | CE | (TG)9, (TG)10 | 209 | 190 | Echt et al. (this paper) | n/a |  |  |  | F primer was evaluated with a 5' dye-CACGACGTTGTAAAACGAC tail. R primer was evaluated with a 5' GTTTCTT tail. |  |
| PtRIP\_0376 | 4 | 2.7 | 6 | 0.08 | gb|BV683083 | UniSTS:513498 | AGGAATTGGTGATTCATGTGG | ATAAAAGAATCGGCCCTGGT | SSR | CE | (AC)14 | 189 | 180 | Echt et al. (this paper) | n/a |  |  |  | F primer was evaluated with a 5' dye-CACGACGTTGTAAAACGAC tail. R primer was evaluated with a 5' GTTTCTT tail. | Locus segregates a null allele. |
| PtRIP\_0388 | 9 | 68.7 | 22 | 1.1 | gb|BV683084 | UniSTS:513499 | CACAACACTCAAACATGCTCAA | AAGAGGATGTGAGGTCCCAA | SSR | CE | (AC)12 | 203 | 193 | Echt et al. (this paper) | n/a |  |  |  | F primer was evaluated with a 5' dye-CACGACGTTGTAAAACGAC tail. R primer was evaluated with a 5' GTTTCTT tail. |  |
| PtRIP\_0496 | 5 | 16 | 22 | 1.61 | gb|BV683151 | UniSTS:513501 | GTAAGAGTGCCTCGGGTCTG | GGTGGTAGGTAGATCGGCAA | SSR | CE | (TG)12 | 203 | 202 | Echt et al. (this paper) | n/a |  |  |  | F primer was evaluated with a 5' dye-CACGACGTTGTAAAACGAC tail. R primer was evaluated with a 5' GTTTCTT tail. |  |
| PtRIP\_0508 | 2 | 78.3 | 21 | 0.8 | gb|BV683085 | UniSTS:513502 | GGCACAGGTTGGACATCTCT | GTGGTGGAAGGGAGATTTCA | SSR | CE | (CA)11 | 90 | 81 | Echt et al. (this paper) | n/a |  |  |  | F primer was evaluated with a 5' dye-CACGACGTTGTAAAACGAC tail. R primer was evaluated with a 5' GTTTCTT tail. |  |
| PtRIP\_0540 | 1 | 51.6 | 10 | 0.65 | gb|BV683152 | UniSTS:513504 | TGTTGTCATTAGTGGTAGGATCA | AAGCGATGTCACTTGTTGAGAA | SSR | CE | (CA)10 | 200 | 204 | Echt et al. (this paper) | n/a |  |  |  | F primer was evaluated with a 5' dye-CACGACGTTGTAAAACGAC tail. R primer was evaluated with a 5' GTTTCTT tail. | Locus segregates a null allele. |
| PtRIP\_0560 | 11 | 67.7 | 18 | 0.53 | gb|BV683088 | UniSTS:513507 | CATTGGAACTTCACCGAAGG | GTGCTATTGGGTCCAGCAAT | SSR | CE | (AC)18, (AT)6 | 108 | 86 | Echt et al. (this paper) | n/a |  |  |  | F primer was evaluated with a 5' dye-CACGACGTTGTAAAACGAC tail. R primer was evaluated with a 5' GTTTCTT tail. | Locus segregates a null allele. |
| PtRIP\_0567 | 6 | 6.8 | 14 | 2.6, jump>5 | gb|BV683089 | UniSTS:513508 | GTTGGTGAGGAGACTTGGGA | AAGAACAATTCCAATATGGATGA | SSR | CE | (AC)16, (TG)6 | 152 | 140 | Echt et al. (this paper) | n/a |  |  |  | F primer was evaluated with a 5' dye-CACGACGTTGTAAAACGAC tail. R primer was evaluated with a 5' GTTTCTT tail. |  |
| PtRIP\_0609 | 6 | 36.7 | 12 | 1.95 | gb|BV683090 | UniSTS:513510 | CAAAATGCAGAGGGGCTTAA | CCAGTCCATCGAATCACGTA | SSR | CE | (AC)12 | 154 | 143 | Echt et al. (this paper) | n/a |  |  |  | F primer was evaluated with a 5' dye-CACGACGTTGTAAAACGAC tail. R primer was evaluated with a 5' GTTTCTT tail. | Locus segregates a null allele, and 1bp allele. |
| PtRIP\_0619 | 6 | 33.2 | 16 | 0.94 | gb|BV683091 | UniSTS:513511 | CAGCTCTCTTAATAGCCTCGG | GCACATAGCAACGCTGAAGA | SSR | CE | (TG)14 | 191 | 199 | Echt et al. (this paper) | n/a |  |  |  | F primer was evaluated with a 5' dye-CACGACGTTGTAAAACGAC tail. R primer was evaluated with a 5' GTTTCTT tail. |  |
| PtRIP\_0621 | 10 | 63.42 | 16 | n/a | gb|BV683092 | UniSTS:513512 | GCAAAGGGAAGCAAAGTCAT | TTCGTCCTCTTTTGAACGAGT | SSR | CE | (TG)15(AG)13 | 154 | 197 | Echt et al. (this paper) | n/a |  |  |  | F primer was evaluated with a 5' dye-CACGACGTTGTAAAACGAC tail. R primer was evaluated with a 5' GTTTCTT tail. |  |
| PtRIP\_0627 | 5 | 89.6 | 22 | 3.05 | gb|BV683093 | UniSTS:513513 | GACAAACAACCCTTGCGTTT | GACCCATCAAGCCAACATG | SSR | CE | (CA)13 | 168 | 169 | Echt et al. (this paper) | n/a |  |  |  | F primer was evaluated with a 5' dye-CACGACGTTGTAAAACGAC tail. R primer was evaluated with a 5' GTTTCTT tail. |  |
| PtRIP\_0630 | 5 | 19.3 | 19 | 0.27 | gb|BV683095 | UniSTS:513515 | CGCAAGCTATGATACAACGC | TGTTGGCTGAGTGTGAAAGC | SSR | CE | (AC)12 | 157 | 151 | Echt et al. (this paper) | n/a |  |  |  | F primer was evaluated with a 5' dye-CACGACGTTGTAAAACGAC tail. R primer was evaluated with a 5' GTTTCTT tail. |  |
| PtRIP\_0647 | 10 | 43.8 | 17 | 0.310 | gb|BV683097 | UniSTS:513517 | TGGCCATCGAACTTGTGTTA | CACGACCACCAGTCACCTTA | SSR | CE | (TG)14 | 214 | 215 | Echt et al. (this paper) | n/a |  |  |  | F primer was evaluated with a 5' dye-CACGACGTTGTAAAACGAC tail. R primer was evaluated with a 5' GTTTCTT tail. | Locus segregates a null allele. |
| PtRIP\_0649 | 3 | 31.8 | 31 | 9.6, jump>5 | gb|BV683098 | UniSTS:513518 | TAGTCGAATCGGGCCTGTAC | TTGCTCCTCTGTGTCCTTCA | SSR | CE | (GT)15 | 218 | 205 | Echt et al. (this paper) | n/a |  |  |  | F primer was evaluated with a 5' dye-CACGACGTTGTAAAACGAC tail. R primer was evaluated with a 5' GTTTCTT tail. |  |
| PtRIP\_0658 | 8 | 102.4 | 8 | 4.63, jump>5 | gb|BV683099 | UniSTS:513519 | TGCATGCATTACAAATGTCA | CGCTTTTAAATCAACCAAACG | SSR | CE | (TG)13 | 219 | 216 | Echt et al. (this paper) | n/a |  |  |  | F primer was evaluated with a 5' dye-CACGACGTTGTAAAACGAC tail. R primer was evaluated with a 5' GTTTCTT tail. |  |
| PtRIP\_0675 | 8 | 12.6 | 20 | 22.2, jump>5 | gb|BV683100 | UniSTS:513520 | ACAGATGTCAAGGCCAAAGG | CTGCATTCAAATTACCCGCT | SSR | CE | (AT)6(TG)12 | 172 | 165 | Echt et al. (this paper) | n/a |  |  |  | F primer was evaluated with a 5' dye-CACGACGTTGTAAAACGAC tail. R primer was evaluated with a 5' GTTTCTT tail. | Locus segregates a null allele. |
| PtRIP\_0683 | 8 | 64.5 | 19 | 0.95 | gb|BV683101 | UniSTS:513521 | TGAAACCAATCCTTCTGCAA | CTGATTCCTCTGGCTTCTCG | SSR | CE | (TG)13 | 187 | 184 | Echt et al. (this paper) | n/a |  |  |  | F primer was evaluated with a 5' dye-CACGACGTTGTAAAACGAC tail. R primer was evaluated with a 5' GTTTCTT tail. |  |
| PtRIP\_0689 | 8 | 19.1 | 21 | 2.34, jump>5 | gb|BV683103 | UniSTS:513523 | GAAACTTTCCCCTACGAGCC | TTCCCCAAAAGTTCACAGGT | SSR | CE | (TG)11 | 158 | 148 | Echt et al. (this paper) | n/a |  |  |  | F primer was evaluated with a 5' dye-CACGACGTTGTAAAACGAC tail. R primer was evaluated with a 5' GTTTCTT tail. |  |
| PtRIP\_0700 | 8 | 124.3 | 15 | 0.42 | gb|BV683106 | UniSTS:513526 | TTGCAATTGCGATTAACTGC | ATAATGGCATAGCCGAATCG | SSR | CE | (AC)15 | 180 | 183 | Echt et al. (this paper) | n/a |  |  |  | F primer was evaluated with a 5' dye-CACGACGTTGTAAAACGAC tail. R primer was evaluated with a 5' GTTTCTT tail. |  |
| PtRIP\_0789 | 8 | 45.1 | 28 | 0.26 | gb|BV683108 | UniSTS:513528 | CATCCCAAGCATCCTCAAGT | TCAAAAATGTGGTTTAATGGAAAA | SSR | CE | (CA)18 | 170 | 183 | Echt et al. (this paper) | n/a |  |  |  | F primer was evaluated with a 5' dye-CACGACGTTGTAAAACGAC tail. R primer was evaluated with a 5' GTTTCTT tail. |  |
| PtRIP\_0790 | 1 | 26.8 | 17 | 0.64 | gb|BV683109 | UniSTS:513529 | TTGTGAATTGTGTCCATGGG | ATCGGTGAGGCTTAAACACG | SSR | CE | (CA)26(AT)4 | 182 | 163 | Echt et al. (this paper) | n/a |  |  |  | F primer was evaluated with a 5' dye-CACGACGTTGTAAAACGAC tail. R primer was evaluated with a 5' GTTTCTT tail. | Locus segregates a null allele. |
| PtRIP\_0791 | 4 | 33.9 | 8 | 0.22 | gb|BV683110 | UniSTS:513530 | ATGGAAGGATCCACAACCAA | GGGCTTGTTGCTGGTCTATG | SSR | CE | (AT)4(TG)15 | 168 | 161 | Echt et al. (this paper) | n/a |  |  |  | F primer was evaluated with a 5' dye-CACGACGTTGTAAAACGAC tail. R primer was evaluated with a 5' GTTTCTT tail. | Locus segregates a null allele. |
| PtRIP\_0814 | 5 | 94.6 | 21 | 1.84 | gb|BV683112 | UniSTS:513533 | AAAAAGAATGAGGCGCACAC | CCCGTTTATGGCATTGATTC | SSR | CE | (AC)12, (AT)9 | 100 | 83 | Echt et al. (this paper) | n/a |  |  |  | F primer was evaluated with a 5' dye-CACGACGTTGTAAAACGAC tail. R primer was evaluated with a 5' GTTTCTT tail. |  |
| PtRIP\_0841 | 8 | 74 | 27 | 1.42 | gb|BV683113 | UniSTS:513535 | GTGCTTCCCTTGCTTCAGAC | GCAAATGCAAACTTTGGGTA | SSR | CE | (CA)17 | 202 | 196 | Echt et al. (this paper) | n/a |  |  |  | F primer was evaluated with a 5' dye-CACGACGTTGTAAAACGAC tail. R primer was evaluated with a 5' GTTTCTT tail. |  |
| PtRIP\_0846 | 8 | 74.1 | 22 | 2.82 | gb|BV683114 | UniSTS:513536 | CATTCATGGTTCCAATGTGG | TGATAAGCGTGGATCTCGTG | SSR | CE | (AT)8(TG)15 | 109 | 94 | Echt et al. (this paper) | n/a |  |  |  | F primer was evaluated with a 5' dye-CACGACGTTGTAAAACGAC tail. R primer was evaluated with a 5' GTTTCTT tail. |  |
| PtRIP\_0852 | 8 | 59.3 | 26 | 1.42 | gb|BV683115 | UniSTS:513537 | GTTATCCCCCATGTTGTTGC | GGGTAGAAGCACTATGCTTTCATT | SSR | CE | (TG)18 | 213 | 202 | Echt et al. (this paper) | n/a |  |  |  | F primer was evaluated with a 5' dye-CACGACGTTGTAAAACGAC tail. R primer was evaluated with a 5' GTTTCTT tail. |  |
| PtRIP\_0860 | 12 | 16 | 11 | 0.97 | gb|BV683116 | UniSTS:513538 | TTGAGCAGACATCATCAACACT | CCAGGTTATGCCTCAAAGAG | SSR | CE | (TG)13 | 217 | 214 | Echt et al. (this paper) | n/a |  |  |  | F primer was evaluated with a 5' dye-CACGACGTTGTAAAACGAC tail. R primer was evaluated with a 5' GTTTCTT tail. | Locus segregates a null allele. |
| PtRIP\_0905 | 12 | 33.2 | 12 | 0.75 | gb|BV683117 | UniSTS:513539 | CACGGATCTCTGGAAACCAT | CGCTGGTTTCCCTCAGAATA | SSR | CE | (AC)16 | 194 | 209 | Echt et al. (this paper) | n/a |  |  |  | F primer was evaluated with a 5' dye-CACGACGTTGTAAAACGAC tail. R primer was evaluated with a 5' GTTTCTT tail. |  |
| PtRIP\_0932 | 5 | 8.3 | 5 | 5.71, jump>5 | gb|BV683119 | UniSTS:513541 | GCAAGACCGACTGGATTAGC | GAGGTCATGATATGTGGTGGG | SSR | CE | (TG)6, (TG)7(GT)8 | 130 | 122 | Echt et al. (this paper) | n/a |  |  |  | F primer was evaluated with a 5' dye-CACGACGTTGTAAAACGAC tail. R primer was evaluated with a 5' GTTTCTT tail. |  |
| PtRIP\_0941 | 2 | 63.5 | 22 | 1.05 | gb|BV683120 | UniSTS:513542 | CTGCGTAGCAAATCACTGGA | TGATCTGATGTGGGATCAACA | SSR | CE | (AT)6(TG)10 | 151 | 153 | Echt et al. (this paper) | n/a |  |  |  | F primer was evaluated with a 5' dye-CACGACGTTGTAAAACGAC tail. R primer was evaluated with a 5' GTTTCTT tail. |  |
| PtRIP\_0958 | 2 | 133.8 | 9 | 1 | gb|BV683122 | UniSTS:513544 | TGGAGTCTCGAACACTGTGG | AATCATCCCAATGGCAACAT | SSR | CE | (AC)15 | 111 | 98 | Echt et al. (this paper) | n/a |  |  |  | F primer was evaluated with a 5' dye-CACGACGTTGTAAAACGAC tail. R primer was evaluated with a 5' GTTTCTT tail. | Locus segregates a null allele. |
| PtRIP\_0960 | 6 | 97.2 | 22 | 0.85 | gb|BV683123 | UniSTS:513545 | GCATCCATCTTCAGCATCCT | TTCATACGACACCTTTGAAATG | SSR | CE | (AC)18, (AT)4 | 188 | 180 | Echt et al. (this paper) | n/a |  |  |  | F primer was evaluated with a 5' dye-CACGACGTTGTAAAACGAC tail. R primer was evaluated with a 5' GTTTCTT tail. |  |
| PtRIP\_0968 | 2 | 67.5 | 30 | 1.27 | gb|BV683124 | UniSTS:513547 | TCTACGACAAAACCACGTAGTG | CATGTGGCTTTGTGGCATAT | SSR | CE | (TG)22 | 201 | 196 | Echt et al. (this paper) | n/a |  |  |  | F primer was evaluated with a 5' dye-CACGACGTTGTAAAACGAC tail. R primer was evaluated with a 5' GTTTCTT tail. |  |
| PtRIP\_0984 | 1 | 72.3 | 26 | 2.26 | gb|BV683125 | UniSTS:513548 | TGTGACCTGAAAATTCCCCT | GGCTTGCAACCAGTTCCATA | SSR | CE | (TG)18 | 220 | 216 | Echt et al. (this paper) | n/a |  |  |  | F primer was evaluated with a 5' dye-CACGACGTTGTAAAACGAC tail. R primer was evaluated with a 5' GTTTCTT tail. |  |
| PtRIP\_0990 | 6 | 57.9 | 29 | 2.13 | gb|BV683126 | UniSTS:513549 | GACCTAAAGAGGTTCACGCG | TCAAATCTTGGGTTAGTATGCAGA | SSR | CE | (CA)25 | 220 | 207 | Echt et al. (this paper) | n/a |  |  |  | F primer was evaluated with a 5' dye-CACGACGTTGTAAAACGAC tail. R primer was evaluated with a 5' GTTTCTT tail. |  |
| PtRIP\_1023 | 12 | 4 | 9 | 0.64 | gb|BV683128 | UniSTS:513551 | GAACCCGATGGATTTTCAAA | CAAACTGTAAGCTCAGGAGGA | SSR | CE | (AC)18 | 175 | 153 | Echt et al. (this paper) | n/a |  |  |  | F primer was evaluated with a 5' dye-CACGACGTTGTAAAACGAC tail. R primer was evaluated with a 5' GTTTCTT tail. |  |
| PtRIP\_1027 | 1 | 62.1 | 19 | 1.48 | gb|BV683129 | UniSTS:513552 | CAGTGTTGATTGTGTGCCAG | TCTGCCACAATTTGGAAACA | SSR | CE | (TG)7, (TG)12 | 220 | 218 | Echt et al. (this paper) | n/a |  |  |  | F primer was evaluated with a 5' dye-CACGACGTTGTAAAACGAC tail. R primer was evaluated with a 5' GTTTCTT tail. |  |
| PtRIP\_1035 | 10 | 28.8 | 18 | 0.452 | gb|BV683130 | UniSTS:513553 | AGCATAATGAGCCCTTCTCG | AGAATATGTGTCCCTCCCCC | SSR | CE | (GT)11 | 174 | 170 | Echt et al. (this paper) | n/a |  |  |  | F primer was evaluated with a 5' dye-CACGACGTTGTAAAACGAC tail. R primer was evaluated with a 5' GTTTCTT tail. |  |
| PtRIP\_1036 | 12 | 51.9 | 24 | 2.18 | gb|BV683131 | UniSTS:513554 | TGGTTGTGCGAGATCACAAT | TTGAGGGAATTGAAATTGGG | SSR | CE | (GT)29 | 211 | 184 | Echt et al. (this paper) | n/a |  |  |  | F primer was evaluated with a 5' dye-CACGACGTTGTAAAACGAC tail. R primer was evaluated with a 5' GTTTCTT tail. |  |
| PtRIP\_1037 | 10 | 72.0 | 4 | 6.062 | gb|BV683132 | UniSTS:513555 | TGCTCAATATAGACCACTTGCA | AGCCATAATTCAACAAAAGGAA | SSR | CE | (GC)5(CA)12 | 152 | 145 | Echt et al. (this paper) | n/a |  |  |  | F primer was evaluated with a 5' dye-CACGACGTTGTAAAACGAC tail. R primer was evaluated with a 5' GTTTCTT tail. |  |
| PtRIP\_1040 | 2 | 46 | 22 | 0.3 | gb|BV683133 | UniSTS:513556 | TCAAGGAATTCATTGGAGCC | TTTGGCCATATCAAACCCAT | SSR | CE | (TG)11 | 192 | 191 | Echt et al. (this paper) | n/a |  |  |  | F primer was evaluated with a 5' dye-CACGACGTTGTAAAACGAC tail. R primer was evaluated with a 5' GTTTCTT tail. |  |
| PtRIP\_1072 | 1 | 107.1 | 16 | 0.96 | gb|BV683135 | UniSTS:513558 | TTTCATGACCTTGGAGTGGA | ATTGATCCCATTGTTGCTCC | SSR | CE | (CA)15 | 209 | 213 | Echt et al. (this paper) | n/a |  |  |  | F primer was evaluated with a 5' dye-CACGACGTTGTAAAACGAC tail. R primer was evaluated with a 5' GTTTCTT tail. |  |
| PtRIP\_1077 | 4 | 5 | 10 | 0.14 | gb|BV683137 | UniSTS:513560 | AACATTCTAGCATGCCCCAC | TTGTGGTGGATGTCTCTCCTC | SSR | CE | (TG)15(AT)6 | 220 | 219 | Echt et al. (this paper) | n/a |  |  |  | F primer was evaluated with a 5' dye-CACGACGTTGTAAAACGAC tail. R primer was evaluated with a 5' GTTTCTT tail. |  |
| PtRIP\_9138 | 12 | 0.0 | 5 | 0.46 | gb|BV683142 | UniSTS:513565 | TGAAACCAATTTTTCCCCTTT | CCAAGAAAGACAAGGAGCCA | SSR | CE | (TGA)5 | 229 | 226 | Echt et al. (this paper) | n/a |  |  |  | F primer was evaluated with a 5' dye-CACGACGTTGTAAAACGAC tail. R primer was evaluated with a 5' GTTTCTT tail. |  |
| PtRIP\_9315 | 2 | 32.4 | 28 | 0.71 | gb|BV683144 | UniSTS:513567 | GGCTTAGGCATAGAGGGACC | AACAAGTTGGAAGCCACCAT | SSR | CE | (TG)13 | 219 | 207 | Echt et al. (this paper) | n/a |  |  |  | F primer was evaluated with a 5' dye-CACGACGTTGTAAAACGAC tail. R primer was evaluated with a 5' GTTTCTT tail. |  |
| PtSIFG\_0100 | not mapped | not mapped | not mapped | not mapped | gb|BV728771 | UniSTS:516239 | CGTATTTTGCAGTATTCATACCA | TCAAGAATCGGTGGGCTATC | SSR | CE | (AT)19 | 187 | 173 | Echt et al. (this paper) | n/a |  |  | F and R primers may be reversed from what is in the NCBI UniSTS record. | F primer was evaluated with a 5' dye-CACGACGTTGTAAAACGAC tail. R primer was evaluated with a 5' GTTTCTT tail. |  |
| PtSIFG\_0126 | 12 | 44.3 | 5 | 0.8 | gb|BV728738 | UniSTS:516240 | AGAAAAATGTATGGGCGTGC | TTTTTGGAGGAATGATTGGC | SSR | CE | (AT)8 | 207 | 205 | Echt et al. (this paper) | n/a |  |  | F and R primers may be reversed from what is in the NCBI UniSTS record. | F primer was evaluated with a 5' dye-CACGACGTTGTAAAACGAC tail. R primer was evaluated with a 5' GTTTCTT tail. |  |
| PtSIFG\_0133 | not mapped | not mapped | not mapped | not mapped | gb|BV728783 | UniSTS:516241 | TTATGAAGGCTTGGATTGGC | GCAAAGCCTTTCAGTCCTCT | SSR | CE | (TA)9 | 367 | 494 | Echt et al. (this paper) | n/a |  |  | F and R primers may be reversed from what is in the NCBI UniSTS record. | F primer was evaluated with a 5' dye-CACGACGTTGTAAAACGAC tail. R primer was evaluated with a 5' GTTTCTT tail. |  |
| PtSIFG\_0141 | not mapped | not mapped | not mapped | not mapped | gb|BV728774 | UniSTS:516242 | TTCTCAGGAGATTCTCAGCG | GGGTTTGATTTCTGGCTTCA | SSR | CE | (AT)18 | 113 | 116 | Echt et al. (this paper) | n/a |  |  | F and R primers may be reversed from what is in the NCBI UniSTS record. | F primer was evaluated with a 5' dye-CACGACGTTGTAAAACGAC tail. R primer was evaluated with a 5' GTTTCTT tail. |  |
| PtSIFG\_0145 | 1 | 65.2 | 23 | 4.41 | gb|BV728739 | UniSTS:516243 | GGGTCTATATCCCCACGGTT | GCCTTGAGAAACAGCCAGAC | SSR | CE | (TA)10 | 267 | 267 | Echt et al. (this paper) | n/a |  |  | F and R primers may be reversed from what is in the NCBI UniSTS record. | F primer was evaluated with a 5' dye-CACGACGTTGTAAAACGAC tail. R primer was evaluated with a 5' GTTTCTT tail. | Locus segregates a 1bp allele. |
| PtSIFG\_0166 | 9 | 0.0 | 3 | 0.39 | gb|BV728653 | UniSTS:516244 | ATCCGACCCATGTTCAATTC | AGTTCCTGCACGAGTAACCG | SSR | CE | (AT)11 | 222 | 223 | Echt et al. (this paper) | n/a |  |  |  | F primer was evaluated with a 5' dye-CACGACGTTGTAAAACGAC tail. R primer was evaluated with a 5' GTTTCTT tail. |  |
| PtSIFG\_0167 | 3 | 117.6 | 23 | 1.29 | gb|BV728654 | UniSTS:516245 | TAGAGAACACAGGCCAGGCT | GCCCATAGCGACCTAACAAG | SSR | CE | (AT)10 | 195 | 302 | Echt et al. (this paper) | n/a |  |  |  | F primer was evaluated with a 5' dye-CACGACGTTGTAAAACGAC tail. R primer was evaluated with a 5' GTTTCTT tail. |  |
| PtSIFG\_0168 | 5 | 4 | 13 | 0.52 | gb|BV728740 | UniSTS:516246 | GGTTTGAACCCAGGTCGATA | CATCGAAATGAGGGGAAGTG | SSR | CE | (TA)8 | 350 | 348 | Echt et al. (this paper) | n/a |  |  | F and R primers may be reversed from what is in the NCBI UniSTS record. | F primer was evaluated with a 5' dye-CACGACGTTGTAAAACGAC tail. R primer was evaluated with a 5' GTTTCTT tail. |  |
| PtSIFG\_0174 | 12 | 81 | 18 | 0.53 | gb|BV728655 | UniSTS:516247 | GCCACCTCTGTTTTCTCAGC | ATGAGAACACGCCACCATTA | SSR | CE | (TA)11 | 318 | 318 | Echt et al. (this paper) | n/a |  |  |  | F primer was evaluated with a 5' dye-CACGACGTTGTAAAACGAC tail. R primer was evaluated with a 5' GTTTCTT tail. |  |
| PtSIFG\_0186 | 5 | 30 | 6 | 7.13 | gb|BV728741 | UniSTS:516248 | ACCCACATGGGATGATGTTT | ATGGAAAAGACAGGGCATTG | SSR | CE | (TA)9 | 221 | 217 | Echt et al. (this paper) | n/a |  |  | F and R primers may be reversed from what is in the NCBI UniSTS record. | F primer was evaluated with a 5' dye-CACGACGTTGTAAAACGAC tail. R primer was evaluated with a 5' GTTTCTT tail. |  |
| PtSIFG\_0193 | 11 | 0.0 | 6 | 5.95, jump>5 | gb|BV728742 | UniSTS:516249 | CCCATGCATCAATTCAAGTT | TGTGCGTGGATATGGAAAAA | SSR | CE | (AT)8 | 238 | 230 | Echt et al. (this paper) | n/a |  |  | F and R primers may be reversed from what is in the NCBI UniSTS record. | F primer was evaluated with a 5' dye-CACGACGTTGTAAAACGAC tail. R primer was evaluated with a 5' GTTTCTT tail. |  |
| PtSIFG\_0198 | not mapped | not mapped | not mapped | not mapped | gb|BV728724 | UniSTS:516250 | ACCTACTTGTGAGGGGCCTT | CCTGCTCCTCAAGTCCAGTT | SSR | CE | (TA)8 | 277 | 276 | Echt et al. (this paper) | n/a |  |  |  | F primer was evaluated with a 5' dye-CACGACGTTGTAAAACGAC tail. R primer was evaluated with a 5' GTTTCTT tail. |  |
| PtSIFG\_0203 | 7 | 121.9 | 10 | 0.38 | gb|BV728743 | UniSTS:516251 | GATGGCTACTGTTCGGTGGT | GGAGTACAGTGAGCAACTGAAGG | SSR | CE | (TA)8 | 118 | 110 | Echt et al. (this paper) | n/a |  |  | F and R primers may be reversed from what is in the NCBI UniSTS record. | F primer was evaluated with a 5' dye-CACGACGTTGTAAAACGAC tail. R primer was evaluated with a 5' GTTTCTT tail. |  |
| PtSIFG\_0206 | 1 | 98.1 | 26 | 3.95, jump>5 | gb|BV728656 | UniSTS:516252 | GCATACGAGAGAGGAGTGCC | GCTACCAAGCTCAAAGCAAA | SSR | CE | (AT)13 | 392 | 381 | Echt et al. (this paper) | n/a |  | Duplicates UniSTS marker PtSIFG\_4381. |  | F primer was evaluated with a 5' dye-CACGACGTTGTAAAACGAC tail. R primer was evaluated with a 5' GTTTCTT tail. |  |
| PtSIFG\_0209 | 10 | 30.1 | 10 | 1.00 | gb|BV728744 | UniSTS:516253 | CTGCATCTTCTCCAATGCAA | CCCATAATCCACAGACCGAT | SSR | CE | (AT)10 | 215 | 208 | Echt et al. (this paper) | n/a |  |  | F and R primers may be reversed from what is in the NCBI UniSTS record. | F primer was evaluated with a 5' dye-CACGACGTTGTAAAACGAC tail. R primer was evaluated with a 5' GTTTCTT tail. |  |
| PtSIFG\_0219 | 8 | 11 | 9 | 0.88 | gb|BV728745 | UniSTS:516254 | AGGCTGCTTGCATGAGAAAT | GGAAGCAGAGGCATCTCAAG | SSR | CE | (TA)9 | 129 | 121 | Echt et al. (this paper) | n/a |  |  | F and R primers may be reversed from what is in the NCBI UniSTS record. | F primer was evaluated with a 5' dye-CACGACGTTGTAAAACGAC tail. R primer was evaluated with a 5' GTTTCTT tail. |  |
| PtSIFG\_0237 | 12 | 22.4 | 15 | 1.38 | gb|BV728657 | UniSTS:516255 | GATCCCGAATCTGCGTAGAA | TCGGATCCACATTCACAAAA | SSR | CE | (AT)13 | 385 | 383 | Echt et al. (this paper) | n/a |  | F primer tested as GATCCCGAATTTGCGTAGAA. |  | F primer was evaluated with a 5' dye-CACGACGTTGTAAAACGAC tail. R primer was evaluated with a 5' GTTTCTT tail. |  |
| PtSIFG\_0245 | 8 | 70.9 | 21 | 12.58 | gb|BV728746 | UniSTS:516256 | TTTCAAGGGTGTGAGCACTG | GAGGAGGAAGAAGGTTTGGG | SSR | CE | (GCC)7 | 195 | 183 | Echt et al. (this paper) | n/a |  |  | F and R primers may be reversed from what is in the NCBI UniSTS record. | F primer was evaluated with a 5' dye-CACGACGTTGTAAAACGAC tail. R primer was evaluated with a 5' GTTTCTT tail. |  |
| PtSIFG\_0249 | not mapped | not mapped | not mapped | not mapped | gb|BV728720 | UniSTS:516257 | GGCCTGCAACAAAATGAAAT | CCCTCTGAAAGCAGAATTGC | SSR | CE | (CAG)5 | 303 | 297 | Echt et al. (this paper) | n/a |  |  |  | F primer was evaluated with a 5' dye-CACGACGTTGTAAAACGAC tail. R primer was evaluated with a 5' GTTTCTT tail. |  |
| PtSIFG\_0265 | 4 | 53.9 | 12 | 7.02, jump>5 | gb|BV728747 | UniSTS:516258 | CTGCTCATCATGCTTTTGGA | GAAGCCCTCAAGTGTTCTGC | SSR | CE | (CAG)5 | 405 | 395 | Echt et al. (this paper) | n/a |  | Duplicates UniSTS marker PtSIFG\_1035. | F and R primers may be reversed from what is in the NCBI UniSTS record. | F primer was evaluated with a 5' dye-CACGACGTTGTAAAACGAC tail. R primer was evaluated with a 5' GTTTCTT tail. |  |
| PtSIFG\_0306 | not mapped | not mapped | not mapped | not mapped | gb|BV728728 | UniSTS:516259 | ATCGGTGTTCCAGGATTGAG | GTATTCCCTGGGGTGATCCT | SSR | CE | (GCA)6 | 433 | 269 | Echt et al. (this paper) | n/a |  |  |  | F primer was evaluated with a 5' dye-CACGACGTTGTAAAACGAC tail. R primer was evaluated with a 5' GTTTCTT tail. |  |
| PtSIFG\_0307 | not mapped | not mapped | not mapped | not mapped | gb|BV728772 | UniSTS:516260 | AGTAGCTTCGAGGACCGACA | TCATAAGAAGGGATTTGGATTGA | SSR | CE | (ATG)5 | 305 | 301 | Echt et al. (this paper) | n/a |  |  | F and R primers may be reversed from what is in the NCBI UniSTS record. | F primer was evaluated with a 5' dye-CACGACGTTGTAAAACGAC tail. R primer was evaluated with a 5' GTTTCTT tail. |  |
| PtSIFG\_0338 | not mapped | not mapped | not mapped | not mapped | gb|BV728719 | UniSTS:516261 | TGCTTTCGCTGACCAATAGA | AGCATTACAGGCATTGGAGG | SSR | CE | (TGA)5 | 269 | 267 | Echt et al. (this paper) | n/a |  |  |  | F primer was evaluated with a 5' dye-CACGACGTTGTAAAACGAC tail. R primer was evaluated with a 5' GTTTCTT tail. |  |
| PtSIFG\_0349 | 10 | 11.3 | 9 | 0.974 | gb|BV728658 | UniSTS:516262 | AATTGCAGAGAGGGTGATGG | CAGCCCCATTAAGGACAGAA | SSR | CE | (TAA)7 | 416 | 414 | Echt et al. (this paper) | n/a |  |  |  | F primer was evaluated with a 5' dye-CACGACGTTGTAAAACGAC tail. R primer was evaluated with a 5' GTTTCTT tail. |  |
| PtSIFG\_0371 | 10 | not positioned | 8 | not positioned | gb|BV728748 | UniSTS:516264 | TGAGCAACTCCAGATCTCAAA | GGTCTCTTGGTGCAGGGTTA | SSR | CE | (AGC)5 | 411 | 408 | Echt et al. (this paper) | n/a |  |  | F and R primers may be reversed from what is in the NCBI UniSTS record. | F primer was evaluated with a 5' dye-CACGACGTTGTAAAACGAC tail. R primer was evaluated with a 5' GTTTCTT tail. |  |
| PtSIFG\_0408 | 9 | 58.1 | 17 | 0.85 | gb|BV728749 | UniSTS:516265 | ACATCCCTCAATCATGCAAA | TGAGGCCAAGCTCGATAACT | SSR | CE | (TAA)5 | 323 | 316 | Echt et al. (this paper) | n/a |  |  | F and R primers may be reversed from what is in the NCBI UniSTS record. | F primer was evaluated with a 5' dye-CACGACGTTGTAAAACGAC tail. R primer was evaluated with a 5' GTTTCTT tail. |  |
| PtSIFG\_0418 | not mapped | not mapped | not mapped | not mapped | gb|BV728722 | UniSTS:516266 | TTACGATACCCGACTCTGGC | ACCCCACTTATATCCCCAGC | SSR | CE | (AGG)5 | 246 | 243 | Echt et al. (this paper) | n/a |  |  |  | F primer was evaluated with a 5' dye-CACGACGTTGTAAAACGAC tail. R primer was evaluated with a 5' GTTTCTT tail. |  |
| PtSIFG\_0424 | 12 | 8.5 | 13 | 0.61 | gb|BV728750 | UniSTS:516267 | CAGATTTGGGGGAACGTAGA | ACGGACGCTTCGAGATCTTA | SSR | CE | (CTG)7 | 374 | 368 | Echt et al. (this paper) | n/a |  |  | F and R primers may be reversed from what is in the NCBI UniSTS record. | F primer was evaluated with a 5' dye-CACGACGTTGTAAAACGAC tail. R primer was evaluated with a 5' GTTTCTT tail. |  |
| PtSIFG\_0436 | not mapped | not mapped | not mapped | not mapped | gb|BV728768 | UniSTS:516268 | TGACCCTCCTGTTGTTGTGA | TTGGAGTTAGGCTCCGCTAA | SSR | CE | (TGT)6 | 418 | 415 | Echt et al. (this paper) | n/a |  |  | F and R primers may be reversed from what is in the NCBI UniSTS record. | F primer was evaluated with a 5' dye-CACGACGTTGTAAAACGAC tail. R primer was evaluated with a 5' GTTTCTT tail. |  |
| PtSIFG\_0437 | 3 | 36.09 | 7 | 7.64 | gb|BV728751 | UniSTS:516269 | TCTATGATGGAAGGCCCAAC | GTTCTGCTTGCCCTCTCAAC | SSR | CE | (CTG)6 | 180 | 176 | Echt et al. (this paper) | n/a |  | Duplicates UniSTS marker PtSIFG\_0541. | F and R primers may be reversed from what is in the NCBI UniSTS record. | F primer was evaluated with a 5' dye-CACGACGTTGTAAAACGAC tail. R primer was evaluated with a 5' GTTTCTT tail. | Locus segregates a 1bp allele. |
| PtSIFG\_0440 | 8 | 99.9 | 24 | 0.55 | gb|BV728659 | UniSTS:516270 | CTGATCGAATCTTCCCCAAA | AGTTCCAGTTGGGTTTGCAC | SSR | CE | (GCG)6 | 314 | 309 | Echt et al. (this paper) | n/a |  |  |  | F primer was evaluated with a 5' dye-CACGACGTTGTAAAACGAC tail. R primer was evaluated with a 5' GTTTCTT tail. |  |
| PtSIFG\_0443 | not mapped | not mapped | not mapped | not mapped | gb|BV728729 | UniSTS:516271 | GATCCCTGTGCCAAAAATCT | AAAATATGTCTACCGGGGGC | SSR | CE | (TAA)6 | 104 | 97 | Echt et al. (this paper) | n/a |  |  |  | F primer was evaluated with a 5' dye-CACGACGTTGTAAAACGAC tail. R primer was evaluated with a 5' GTTTCTT tail. |  |
| PtSIFG\_0463 | 1 | 5.3 | 3 | 0.19 | gb|BV728660 | UniSTS:516272 | GCGAGCAAATTACTTCGTCC | CCCTGCACAAGTAGTCACGA | SSR | CE | (TGC)5 | 449 | 445 | Echt et al. (this paper) | n/a |  | Duplicates UniSTS marker PtSIFG\_1041. |  | F primer was evaluated with a 5' dye-CACGACGTTGTAAAACGAC tail. R primer was evaluated with a 5' GTTTCTT tail. |  |
| PtSIFG\_0477 | not mapped | not mapped | not mapped | not mapped | gb|BV728770 | UniSTS:516273 | GCCACAAAAGAAGATCCAGC | CATTCCCCCTCTCAATCTCA | SSR | CE | (AAT)5 | 173 | 168 | Echt et al. (this paper) | n/a |  |  | F and R primers may be reversed from what is in the NCBI UniSTS record. | F primer was evaluated with a 5' dye-CACGACGTTGTAAAACGAC tail. R primer was evaluated with a 5' GTTTCTT tail. |  |
| PtSIFG\_0490 | not mapped | not mapped | not mapped | not mapped | gb|BV728784 | UniSTS:516274 | CCAATTGAGGCAAAGGTCAT | CGAGAGGTGAGCGAGGTAAC | SSR | CE | (ATA)5 | 367 | 365 | Echt et al. (this paper) | n/a |  |  | F and R primers may be reversed from what is in the NCBI UniSTS record. | F primer was evaluated with a 5' dye-CACGACGTTGTAAAACGAC tail. R primer was evaluated with a 5' GTTTCTT tail. |  |
| PtSIFG\_0493 | 2 | 5.3 | 19 | 1.11 | gb|BV728661 | UniSTS:516275 | GAGAACATCTGCCTTGAGCC | CTGGCATGATGGGTTTCTCT | SSR | CE | (CTG)6 | 298 | 289 | Echt et al. (this paper) | n/a |  |  |  | F primer was evaluated with a 5' dye-CACGACGTTGTAAAACGAC tail. R primer was evaluated with a 5' GTTTCTT tail. |  |
| PtSIFG\_0551 | 10 | 6.044 | 14 | 0.424 | gb|BV728752 | UniSTS:516277 | ATCATGTGTGCACTTGCCAT | TGCTGTTTGTGAGCACCTTT | SSR | CE | (AAAAAT)4 | 460 | 444 | Echt et al. (this paper) | n/a |  |  | F and R primers may be reversed from what is in the NCBI UniSTS record. | F primer was evaluated with a 5' dye-CACGACGTTGTAAAACGAC tail. R primer was evaluated with a 5' GTTTCTT tail. |  |
| PtSIFG\_0561 | 12 | 93.8 | 11 | 1.13 | gb|BV728753 | UniSTS:516278 | GCCAACTGCAATAACAGCAA | CCGGCAAGAGCATCATTATT | SSR | CE | (GCAGAA)4 | 434 | 434 | Echt et al. (this paper) | n/a |  |  | F and R primers may be reversed from what is in the NCBI UniSTS record. | F primer was evaluated with a 5' dye-CACGACGTTGTAAAACGAC tail. R primer was evaluated with a 5' GTTTCTT tail. |  |
| PtSIFG\_0563 | not mapped | not mapped | not mapped | not mapped | gb|BV728782 | UniSTS:516279 | TACACAGAAGCCCCATAGCC | CGAGAGCCCTTATTACGCAG | SSR | CE | (TGGCC)4 | 354 | 254 | Echt et al. (this paper) | n/a |  |  | F and R primers may be reversed from what is in the NCBI UniSTS record. | F primer was evaluated with a 5' dye-CACGACGTTGTAAAACGAC tail. R primer was evaluated with a 5' GTTTCTT tail. |  |
| PtSIFG\_0564 | 11 | 52.3 | 18 | 0.97 | gb|BV728754 | UniSTS:516280 | CATCTTCTTCCTCTCTGCGG | ATTATTGTTGCCCGTGGTTG | SSR | CE | (ATTT)4 | 467 | 464 | Echt et al. (this paper) | n/a |  |  | F and R primers may be reversed from what is in the NCBI UniSTS record. | F primer was evaluated with a 5' dye-CACGACGTTGTAAAACGAC tail. R primer was evaluated with a 5' GTTTCTT tail. |  |
| PtSIFG\_0566 | 1 | 25.1 | 9 | 2.09 | gb|BV728755 | UniSTS:516281 | ACTTAGTGGGAAAGGGGGAA | TTCCTCAGCCAAAAGCTCTC | SSR | CE | (GGGAAG)4 | 107 | 109 | Echt et al. (this paper) | n/a |  |  | F and R primers may be reversed from what is in the NCBI UniSTS record. | F primer was evaluated with a 5' dye-CACGACGTTGTAAAACGAC tail. R primer was evaluated with a 5' GTTTCTT tail. |  |
| PtSIFG\_0573 | not mapped | not mapped | not mapped | not mapped | gb|BV728775 | UniSTS:516282 | GCATGAGTTTCTGATGAGGGA | TGGTGATTGGTTATGCTTGC | SSR | CE | (AATT)4 | 486 | 482 | Echt et al. (this paper) | n/a |  |  | F and R primers may be reversed from what is in the NCBI UniSTS record. | F primer was evaluated with a 5' dye-CACGACGTTGTAAAACGAC tail. R primer was evaluated with a 5' GTTTCTT tail. |  |
| PtSIFG\_0587 | 8 | 115.1 | 10 | 0.85 | gb|BV728756 | UniSTS:516283 | TACGGTCACACTTCACCCAA | TCCTCCTAGTGCAAATGGCT | SSR | CE | (CTTCAT)4 | 286 | 282 | Echt et al. (this paper) | n/a |  |  | F and R primers may be reversed from what is in the NCBI UniSTS record. | F primer was evaluated with a 5' dye-CACGACGTTGTAAAACGAC tail. R primer was evaluated with a 5' GTTTCTT tail. |  |
| PtSIFG\_0592 | 11 | 44 | 19 | 1.25 | gb|BV728662 | UniSTS:516284 | TCGGACGAATGCGAAATTAT | CCTGGTGTTGCCTGAAAACT | SSR | CE | (CACACC)4 | 401 | 395 | Echt et al. (this paper) | n/a |  |  |  | F primer was evaluated with a 5' dye-CACGACGTTGTAAAACGAC tail. R primer was evaluated with a 5' GTTTCTT tail. |  |
| PtSIFG\_0594 | 3 | 61.6 | 24 | 7.74 | gb|BV728663 | UniSTS:516285 | ATGAGGAGGAACGTTTGGTG | TGGCAATGGCATTACGAATA | SSR | CE | (CCTTGA)4 | 381 | 375 | Echt et al. (this paper) | n/a |  |  |  | F primer was evaluated with a 5' dye-CACGACGTTGTAAAACGAC tail. R primer was evaluated with a 5' GTTTCTT tail. |  |
| PtSIFG\_0596 | 12 | 28 | 11 | 1.16 | gb|BV728664 | UniSTS:516286 | GACTGAACTCTCCCTGTGGC | GATGGGTTTGAGATCGTGCT | SSR | CE | (AATA)4 | 344 | 335 | Echt et al. (this paper) | n/a |  |  |  | F primer was evaluated with a 5' dye-CACGACGTTGTAAAACGAC tail. R primer was evaluated with a 5' GTTTCTT tail. | Locus segregates a null allele. |
| PtSIFG\_0625 | 3 | 65.4 | 17 | 2.66 | gb|BV728665 | UniSTS:516287 | TAGCAGTGCACCGAAGTCAC | TTCTCCCAGTGGAGTTTTGG | SSR | CE | (TGCA)4 | 391 | 385 | Echt et al. (this paper) | n/a |  |  |  | F primer was evaluated with a 5' dye-CACGACGTTGTAAAACGAC tail. R primer was evaluated with a 5' GTTTCTT tail. |  |
| PtSIFG\_0629 | 9 | 30.7 | 13 | 0.44 | gb|BV728666 | UniSTS:516288 | CATGGGCGAGATCAAGAGAT | GAAAGGAAAGGAAACCTCCG | SSR | CE | (AACGGA)5 | 143 | 131 | Echt et al. (this paper) | n/a |  |  |  | F primer was evaluated with a 5' dye-CACGACGTTGTAAAACGAC tail. R primer was evaluated with a 5' GTTTCTT tail. |  |
| PtSIFG\_0635 | 6 | 57.7 | 22 | 0.72 | gb|BV728757 | UniSTS:516289 | AAATGTGAAGGGTTTGCCAC | CAATCCATGTTGTGCTCCAG | SSR | CE | (GATA)4 | 416 | 418 | Echt et al. (this paper) | n/a |  | Duplicates UniSTS marker PtSIFG\_1274. | F and R primers may be reversed from what is in the NCBI UniSTS record. | F primer was evaluated with a 5' dye-CACGACGTTGTAAAACGAC tail. R primer was evaluated with a 5' GTTTCTT tail. |  |
| PtSIFG\_0636 | not mapped | not mapped | not mapped | not mapped | gb|BV728769 | UniSTS:516290 | CAGCGGCAGTACATGAAAAA | AGGTTGCGAAAGAAAAGCAA | SSR | CE | (GACTG)4 | 289 | 285 | Echt et al. (this paper) | n/a |  | Duplicates UniSTS marker PtSIFG\_1271. | F and R primers may be reversed from what is in the NCBI UniSTS record. | F primer was evaluated with a 5' dye-CACGACGTTGTAAAACGAC tail. R primer was evaluated with a 5' GTTTCTT tail. |  |
| PtSIFG\_0639 | not mapped | not mapped | not mapped | not mapped | gb|BV728708 | UniSTS:516291 | ACGGCAAAATATCCAAGTCG | TGAGAGTTGAGGTAGGGGAA | SSR | CE | (AATA)4 | 491 | 485 | Echt et al. (this paper) | n/a |  |  |  | F primer was evaluated with a 5' dye-CACGACGTTGTAAAACGAC tail. R primer was evaluated with a 5' GTTTCTT tail. |  |
| PtSIFG\_0640 | 3 | 63.7 | 34 | 1.88 | gb|BV728667 | UniSTS:516292 | GCGACATCGATTTTGGTTTT | TGTCAATCAATACTGGGAATAAA | SSR | CE | (ATCTGT)4 | 362 | 345 | Echt et al. (this paper) | n/a |  |  |  | F primer was evaluated with a 5' dye-CACGACGTTGTAAAACGAC tail. R primer was evaluated with a 5' GTTTCTT tail. |  |
| PtSIFG\_0652 | 8 | 26.5 | 15 | 0.22 | gb|BV728668 | UniSTS:516293 | TTCTCAGAATTTGCTGGGCT | TGCTATCATCGGAATCTCCC | SSR | CE | (GCA)4(AGCAAG)4 | 152 | 142 | Echt et al. (this paper) | n/a |  |  |  | F primer was evaluated with a 5' dye-CACGACGTTGTAAAACGAC tail. R primer was evaluated with a 5' GTTTCTT tail. |  |
| PtSIFG\_0653 | not mapped | not mapped | not mapped | not mapped | gb|BV728727 | UniSTS:516294 | AATGCTTCCATCCATTCAGC | TTCTGGGTACATGCCTCTCC | SSR | CE | (TC)4(AAAC)4 | 389 | 379 | Echt et al. (this paper) | n/a |  |  |  | F primer was evaluated with a 5' dye-CACGACGTTGTAAAACGAC tail. R primer was evaluated with a 5' GTTTCTT tail. |  |
| PtSIFG\_0662 | not mapped | not mapped | not mapped | not mapped | gb|BV728711 | UniSTS:516295 | CATCCACCCCTTCTCTTCAA | TTTGAATGAATGCTCTCCCA | SSR | CE | (AGTCGC)4 | 474 | 465 | Echt et al. (this paper) | n/a |  |  |  | F primer was evaluated with a 5' dye-CACGACGTTGTAAAACGAC tail. R primer was evaluated with a 5' GTTTCTT tail. |  |
| PtSIFG\_0668 | not mapped | not mapped | not mapped | not mapped | gb|BV728779 | UniSTS:516296 | TAATCGCCTATGTTCAGGGG | TGTCATCCGGAGTTAGCACA | SSR | CE | (GAG)4(TAAA)4 | 364 | 384 | Echt et al. (this paper) | n/a |  |  | F and R primers may be reversed from what is in the NCBI UniSTS record. | F primer was evaluated with a 5' dye-CACGACGTTGTAAAACGAC tail. R primer was evaluated with a 5' GTTTCTT tail. |  |
| PtSIFG\_0715 | 5 | 56.4 | 30 | n/a | gb|BV728781 | UniSTS:516297 | TTCTTTTGAGGGATTGGACG | GGAAATGGCATCATTGCTTT | SSR | CE | (AT)21, (CTT)5(AGC)5 | 234 | 225 | Echt et al. (this paper) | n/a |  |  | F and R primers may be reversed from what is in the NCBI UniSTS record. | F primer was evaluated with a 5' dye-CACGACGTTGTAAAACGAC tail. R primer was evaluated with a 5' GTTTCTT tail. | Locus segregates a 1bp allele. |
| PtSIFG\_0737 | 10 | not positioned | 3 | not positioned | gb|BV728669 | UniSTS:516298 | GCAAGGGGAATTGCTTATGA | GGGATCGCATCAGCTGTAAT | SSR | CE | (CAG)6 (CAGCAT)6 | 429 | 424 | Echt et al. (this paper) | n/a |  |  |  | F primer was evaluated with a 5' dye-CACGACGTTGTAAAACGAC tail. R primer was evaluated with a 5' GTTTCTT tail. |  |
| PtSIFG\_0740 | 3 | 61.4 | 34 | 1.79 | gb|BV728670 | UniSTS:516299 | CCAAGAAACGTGCAAGGAAT | TGGTACTGGAGATTCTGGGC | SSR | CE | (AT)18(TA)13 | 354 | 331 | Echt et al. (this paper) | n/a |  |  |  | F primer was evaluated with a 5' dye-CACGACGTTGTAAAACGAC tail. R primer was evaluated with a 5' GTTTCTT tail. |  |
| PtSIFG\_0745 | 10 | 0.0 | 11 | 1.196 | gb|BV728671 | UniSTS:516300 | AAGAAGGGCGGACTAGGAGC | GTGAACCCACAATTCCCAAC | SSR | CE | (AGGTTG)4(GGCTGA)5 | 480 | 479 | Echt et al. (this paper) | n/a |  | F primer tested as AAGAAAGGCGGACTAGGAGC. |  | F primer was evaluated with a 5' dye-CACGACGTTGTAAAACGAC tail. R primer was evaluated with a 5' GTTTCTT tail. |  |
| PtSIFG\_1005 | 7 | 89.4 | 13 | 0.61 | gb|BV728672 | UniSTS:516302 | GACCTGCCTTTAATTATATTCATCG | TACAATTGTGTGAGCGTCTCG | SSR | CE | (TTTCC)4 | 142 | 132 | Echt et al. (this paper) | n/a |  |  |  | F primer was evaluated with a 5' dye-CACGACGTTGTAAAACGAC tail. R primer was evaluated with a 5' GTTTCTT tail. |  |
| PtSIFG\_1008 | not mapped | not mapped | not mapped | not mapped | gb|BV728723 | UniSTS:516303 | CCCTCAAAAACACGTAGACGA | TCTTGCATTCCACATTTCACA | SSR | CE | (GAC)7 | 199 | 195 | Echt et al. (this paper) | n/a |  | Duplicates UniSTS marker SsrPt\_ctg7024, PtSIFG\_1069, PtSIFG\_1008. |  | F primer was evaluated with a 5' dye-CACGACGTTGTAAAACGAC tail. R primer was evaluated with a 5' GTTTCTT tail. |  |
| PtSIFG\_1018 | 11 | 85 | 10 | 0.7 | gb|BV728673 | UniSTS:516304 | CTCGTTGTGGCTGGTATTTGT | CTCTTCTGCACGATATCTCCG | SSR | CE | (CGG)6 | 317 | 311 | Echt et al. (this paper) | n/a |  | Duplicates UniSTS marker PtSIFG\_1051, SsrPt\_BF778306, RPtest8 |  | F primer was evaluated with a 5' dye-CACGACGTTGTAAAACGAC tail. R primer was evaluated with a 5' GTTTCTT tail. |  |
| PtSIFG\_1030 | not mapped | not mapped | not mapped | not mapped | gb|BV728702 | UniSTS:516305 | TGAATTTCACAAATACACTAAAGGTT | CACCGGTTGTGCTAATGAGAT | SSR | CE | (ATTT)3 | 184 | 179 | Echt et al. (this paper) | n/a |  |  |  | F primer was evaluated with a 5' dye-CACGACGTTGTAAAACGAC tail. R primer was evaluated with a 5' GTTTCTT tail. |  |
| PtSIFG\_1032 | not mapped | not mapped | not mapped | not mapped | gb|BV728714 | UniSTS:516306 | TTTTGTTTGGGTTCGTCTGTT | TGAGACCATAAGAGCAGCGAT | SSR | CE | (CAG)3 | 285 | 282 | Echt et al. (this paper) | n/a |  |  |  | F primer was evaluated with a 5' dye-CACGACGTTGTAAAACGAC tail. R primer was evaluated with a 5' GTTTCTT tail. |  |
| PtSIFG\_1052 | 2 | 81.9 | 13 | 1.86 | gb|BV728674 | UniSTS:516310 | GGTCGAATCAAATTGGGAAAA | GGAAAAATCTATGCCTACGCC | SSR | CE | (TC)6 | 122 | 116 | Echt et al. (this paper) | n/a |  |  |  | F primer was evaluated with a 5' dye-CACGACGTTGTAAAACGAC tail. R primer was evaluated with a 5' GTTTCTT tail. |  |
| PtSIFG\_1055 | 5 | 82.6 | 25 | 0.5 | gb|BV728675 | UniSTS:516311 | CGGAGAAAACAGCCAGTATCA | TTTGAGCATTGTTTTGCTCCT | SSR | CE | (AGA)5 | 109 | 102 | Echt et al. (this paper) | n/a |  |  |  | F primer was evaluated with a 5' dye-CACGACGTTGTAAAACGAC tail. R primer was evaluated with a 5' GTTTCTT tail. | Locus segregates a null allele. |
| PtSIFG\_1060 | not mapped | not mapped | not mapped | not mapped | gb|BV728696 | UniSTS:516312 | GAAAATCCGGACGAAAACAGT | GTGTCATCTGGCTTCTGCTTC | SSR | CE | (AT)8 | 182 | 176 | Echt et al. (this paper) | n/a |  | R primer tested as TGTGTCATCTGGCTTCTGCTTC. |  | F primer was evaluated with a 5' dye-CACGACGTTGTAAAACGAC tail. R primer was evaluated with a 5' GTTTCTT tail. |  |
| PtSIFG\_1062 | 9 | 59.5 | 13 | 1.09 | gb|BV728676 | UniSTS:516313 | ATTGAAAAATACAGCGGCTCA | GCGAGCCACAGTTGATTATGT | SSR | CE | (TA)9 | 209 | 205 | Echt et al. (this paper) | n/a |  |  |  | F primer was evaluated with a 5' dye-CACGACGTTGTAAAACGAC tail. R primer was evaluated with a 5' GTTTCTT tail. |  |
| PtSIFG\_1065 | 12 | 81.4 | 12 | 0.51 | gb|BV728677 | UniSTS:516314 | AAACTTACAGTCTCGTCGCCA | CACTCTTCTGGTTGTGCATGA | SSR | CE | (AT)6 | 103 | 98 | Echt et al. (this paper) | n/a |  |  |  | F primer was evaluated with a 5' dye-CACGACGTTGTAAAACGAC tail. R primer was evaluated with a 5' GTTTCTT tail. |  |
| PtSIFG\_1066 | not mapped | not mapped | not mapped | not mapped | gb|BV728706 | UniSTS:516315 | GACAGATGCCCATACAACAGG | TCTCTTCGCGCAAATTATACTC | SSR | CE | (AGC)5(CAA)3(CAG)4(CAA)3 | 132 | 122 | Echt et al. (this paper) | n/a |  |  |  | F primer was evaluated with a 5' dye-CACGACGTTGTAAAACGAC tail. R primer was evaluated with a 5' GTTTCTT tail. |  |
| PtSIFG\_1102 | not mapped | not mapped | not mapped | not mapped | gb|BV728715 | UniSTS:516317 | ACGGAGATATATTGCAGGCG | AAAGAATAACCTGAAACAAACCC | SSR | CE | (TA)16 | 119 | 140 | Echt et al. (this paper) | n/a |  |  |  | F primer was evaluated with a 5' dye-CACGACGTTGTAAAACGAC tail. R primer was evaluated with a 5' GTTTCTT tail. |  |
| PtSIFG\_1110 | not mapped | not mapped | not mapped | not mapped | gb|BV728703 | UniSTS:516318 | GGACAGTCCTTACTGCCCAA | CCCATGGTTTTCCATTGTTC | SSR | CE | (AT)26 | 182 | 152 | Echt et al. (this paper) | n/a |  |  |  | F primer was evaluated with a 5' dye-CACGACGTTGTAAAACGAC tail. R primer was evaluated with a 5' GTTTCTT tail. |  |
| PtSIFG\_1113 | 11 | 13.1 | 8 | 0.48 | gb|BV728678 | UniSTS:516319 | TAATAATTCAAGCCACCCCG | GGGTTGCAGCCTCTGAAATA | SSR | CE | (AT)10 | 121 | 111 | Echt et al. (this paper) | n/a |  | Duplicates UniSTS marker PtSIFG\_1002. |  | F primer was evaluated with a 5' dye-CACGACGTTGTAAAACGAC tail. R primer was evaluated with a 5' GTTTCTT tail. | Locus segregates a 1bp allele. |
| PtSIFG\_1123 | 8 | 3.1 | 12 | 9.56 | gb|BV728758 | UniSTS:516321 | TGGTTCAACGGAAACCCTTA | GTTTTCTCAGCCTTGCGTTC | SSR | CE | (AT)8 | 118 | 108 | Echt et al. (this paper) | n/a |  |  | F and R primers may be reversed from what is in the NCBI UniSTS record. | F primer was evaluated with a 5' dye-CACGACGTTGTAAAACGAC tail. R primer was evaluated with a 5' GTTTCTT tail. |  |
| PtSIFG\_1166 | 3 | 19.9 | 8 | 1.49 | gb|BV728759 | UniSTS:516322 | CCTGTTCGGACTGCTGAGAA | CCTCTGAGCCTTCAAAGAGG | SSR | CE | (AGG)5 | 320 | 318 | Echt et al. (this paper) | n/a |  | F primer tested as CATGTTCGGACTGCTGAGAA; R primer tested as CCTGTGAGCCTTCAAAGAGG. | F and R primers may be reversed from what is in the NCBI UniSTS record. | F primer was evaluated with a 5' dye-CACGACGTTGTAAAACGAC tail. R primer was evaluated with a 5' GTTTCTT tail. | Locus segregates a 1bp allele. |
| PtSIFG\_1184 | not mapped | not mapped | not mapped | not mapped | gb|BV728698 | UniSTS:516324 | AAGCCCTTGCACTTTGTGAG | CCTCTTTTCTTTCAATCTTTGCC | SSR | CE | (AAG)7 | 147 | 140 | Echt et al. (this paper) | n/a |  |  |  | F primer was evaluated with a 5' dye-CACGACGTTGTAAAACGAC tail. R primer was evaluated with a 5' GTTTCTT tail. |  |
| PtSIFG\_1185 | not mapped | not mapped | not mapped | not mapped | gb|BV728716 | UniSTS:516325 | GATTATCCACGGCGAAAAGA | GGGAATTCGACCTGTGAAGA | SSR | CE | (AGC)7 | 379 | 377 | Echt et al. (this paper) | n/a |  |  |  | F primer was evaluated with a 5' dye-CACGACGTTGTAAAACGAC tail. R primer was evaluated with a 5' GTTTCTT tail. |  |
| PtSIFG\_1190 | 9 | 76 | 14 | 0.93 | gb|BV728679 | UniSTS:516327 | CAGGTGGCTTGGATTTCATT | TCATTCAAGCGTCCTGCTTA | SSR | CE | (TCC)7 | 290 | 284 | Echt et al. (this paper) | n/a |  |  |  | F primer was evaluated with a 5' dye-CACGACGTTGTAAAACGAC tail. R primer was evaluated with a 5' GTTTCTT tail. |  |
| PtSIFG\_1207 | 2 | 10.7 | 12 | 0.38 | gb|BV728789 | UniSTS:516328 | TTGAAAGACCTGAGGGAACG | ACAGTGCTTCAACGTGCATC | SSR | CE | (TTA)6 | 221 | 214 | Echt et al. (this paper) | n/a |  |  |  | F primer was evaluated with a 5' dye-CACGACGTTGTAAAACGAC tail. R primer was evaluated with a 5' GTTTCTT tail. |  |
| PtSIFG\_1212 | 5 | 22.1 | 2 | 0.21 | n/a | n/a | CCGGAAACATCTCTTTGGAA | TGCCACTTTAATTCCATTCCTC | SSR | CE | (TTA)6 | 314 | 315 | Echt et al. (this paper) | n/a |  |  |  | F primer was evaluated with a 5' dye-CACGACGTTGTAAAACGAC tail. R primer was evaluated with a 5' GTTTCTT tail. |  |
| PtSIFG\_1217 | not mapped | not mapped | not mapped | not mapped | gb|BV728713 | UniSTS:516329 | TAAATTCAGTTGGGCCCTTG | GATCAATCATGGCTGCAGAA | SSR | CE | (GAA)5 | 187 | 183 | Echt et al. (this paper) | n/a |  |  |  | F primer was evaluated with a 5' dye-CACGACGTTGTAAAACGAC tail. R primer was evaluated with a 5' GTTTCTT tail. |  |
| PtSIFG\_1241 | not mapped | not mapped | not mapped | not mapped | gb|BV728726 | UniSTS:516330 | CCTCGCTGCCAATTTGTTAT | AAAGTTGCATCTCCGAATTG | SSR | CE | (CGGTGG)5 | 169 | 155 | Echt et al. (this paper) | n/a |  | R primer tested as AAAGTTGCATCTCCGAATTG. |  | F primer was evaluated with a 5' dye-CACGACGTTGTAAAACGAC tail. R primer was evaluated with a 5' GTTTCTT tail. |  |
| PtSIFG\_1252 | 2 | 1 | 7 | 0.72 | gb|BV728680 | UniSTS:516331 | AAGCCCCTTCCTCGTACATT | CCAAGTGAACACCATCATCG | SSR | CE | (GCTGAT)4 | 370 | 357 | Echt et al. (this paper) | n/a |  |  |  | F primer was evaluated with a 5' dye-CACGACGTTGTAAAACGAC tail. R primer was evaluated with a 5' GTTTCTT tail. | Locus segregates a null allele. |
| PtSIFG\_1260 | 8 | 62.9 | 9 | 1.09 | gb|BV728792 | UniSTS:516332 | TTCAGTGATTTTACTCCTTCGTTG | GATTATTGCAAGGAGGGGATG | SSR | CE | (TTCCT)4 | 108 | 102 | Echt et al. (this paper) | n/a |  |  |  | F primer was evaluated with a 5' dye-CACGACGTTGTAAAACGAC tail. R primer was evaluated with a 5' GTTTCTT tail. |  |
| PtSIFG\_1262 | 8 | 122 | 10 | 0.37 | gb|BV728681 | UniSTS:516333 | AGGCGAAAAGATTTGAAGCA | TCCTTAAGCCGATCAACGAC | SSR | CE | (AAGAA)4 | 403 | 490 | Echt et al. (this paper) | n/a |  |  |  | F primer was evaluated with a 5' dye-CACGACGTTGTAAAACGAC tail. R primer was evaluated with a 5' GTTTCTT tail. | Locus segregates a null allele. |
| PtSIFG\_1295 | not mapped | not mapped | not mapped | not mapped | gb|BV728699 | UniSTS:516335 | TTCGGCTCTATCTCAGGGAA | GATTGTGATTGAGGTTGGGG | SSR | CE | (CAA)5 | 267 | 259 | Echt et al. (this paper) | n/a |  |  |  | F primer was evaluated with a 5' dye-CACGACGTTGTAAAACGAC tail. R primer was evaluated with a 5' GTTTCTT tail. |  |
| PtSIFG\_1318 | 5 | 103.5 | 8 | 0.51 | gb|BV728682 | UniSTS:516336 | GGACTGGCAAAACTGTTCGT | AAGACGAAGATGAGCCCAGA | SSR | CE | (AT)6(TCT)5 | 242 | 238 | Echt et al. (this paper) | n/a |  |  |  | F primer was evaluated with a 5' dye-CACGACGTTGTAAAACGAC tail. R primer was evaluated with a 5' GTTTCTT tail. |  |
| PtSIFG\_1325 | 2 | 11.4 | 19 | 0.72 | gb|BV728683 | UniSTS:516337 | GCGTGGAGGTTACACCAAAC | TTTTCCGCTGGATTTACCAC | SSR | CE | (GAT)10(TGA)5 | 302 | 298 | Echt et al. (this paper) | n/a |  | Duplicates UniSTS marker PtSIFG\_0358, SsrPt\_ctg4698, PtTX3118 |  | F primer was evaluated with a 5' dye-CACGACGTTGTAAAACGAC tail. R primer was evaluated with a 5' GTTTCTT tail. |  |
| PtSIFG\_2229 | 1 | 114.6 | 11 | 0.68 | gb|BV728684 | UniSTS:516338 | TCAATCAGTTTTCCTTCCGC | GGTCTCCAAATACGGGGAGT | SSR | CE | (CAG)5 | 439 | 435 | Echt et al. (this paper) | n/a |  |  |  | F primer was evaluated with a 5' dye-CACGACGTTGTAAAACGAC tail. R primer was evaluated with a 5' GTTTCTT tail. |  |
| PtSIFG\_2461 | not mapped | not mapped | not mapped | not mapped | gb|BV728721 | UniSTS:516339 | AAACTGCGTGAGGTGCTCTT | CTTCTCGATGATGTGCCTGA | SSR | CE | (GCAG)4 | 433 | 435 | Echt et al. (this paper) | n/a |  |  |  | F primer was evaluated with a 5' dye-CACGACGTTGTAAAACGAC tail. R primer was evaluated with a 5' GTTTCTT tail. |  |
| PtSIFG\_2510 | not mapped | not mapped | not mapped | not mapped | gb|BV728710 | UniSTS:516340 | CCACGGAACTGGTCTCAGAT | GGCATCTCCTGGATTGTCAT | SSR | CE | (ACGGCA)4 | 459 | 452 | Echt et al. (this paper) | n/a |  |  |  | F primer was evaluated with a 5' dye-CACGACGTTGTAAAACGAC tail. R primer was evaluated with a 5' GTTTCTT tail. |  |
| PtSIFG\_4102 | not mapped | not mapped | not mapped | not mapped | gb|BV728704 | UniSTS:516341 | CTTTGTTGACCCCTGCATTT | TTGGCTTAGCTAAAAGGGTGA | SSR | CE | (TA)6 | 200 | 195 | Echt et al. (this paper) | n/a |  |  |  | F primer was evaluated with a 5' dye-CACGACGTTGTAAAACGAC tail. R primer was evaluated with a 5' GTTTCTT tail. |  |
| PtSIFG\_4110 | not mapped | not mapped | not mapped | not mapped | gb|BV728705 | UniSTS:516342 | TGATTGAGGTCTCGACTCCC | GGATGTGGCTGTTCCAGATT | SSR | CE | (TG)6 | 412 | 408 | Echt et al. (this paper) | n/a |  |  |  | F primer was evaluated with a 5' dye-CACGACGTTGTAAAACGAC tail. R primer was evaluated with a 5' GTTTCTT tail. |  |
| PtSIFG\_4133 | not mapped | not mapped | not mapped | not mapped | gb|BV728776 | UniSTS:516343 | GATGATGGGGGATCAGAAGA | GTACTGACGTGACCCTGCAA | SSR | CE | (TA)7 | 287 | 285 | Echt et al. (this paper) | n/a |  |  | F and R primers may be reversed from what is in the NCBI UniSTS record. | F primer was evaluated with a 5' dye-CACGACGTTGTAAAACGAC tail. R primer was evaluated with a 5' GTTTCTT tail. |  |
| PtSIFG\_4160 | not mapped | not mapped | not mapped | not mapped | gb|BV728773 | UniSTS:516344 | AAAGGGATGCTTTTAAGGCT | CATCGGCAGTTGTTCAGCTA | SSR | CE | (TA)7 | 464 | 454 | Echt et al. (this paper) | n/a |  |  | F and R primers may be reversed from what is in the NCBI UniSTS record. | F primer was evaluated with a 5' dye-CACGACGTTGTAAAACGAC tail. R primer was evaluated with a 5' GTTTCTT tail. |  |
| PtSIFG\_4177 | not mapped | not mapped | not mapped | not mapped | gb|BV728717 | UniSTS:516345 | CCAACGCATTTGTGATTTTG | TTCCTAAAGTGTGTGTGAAACAA | SSR | CE | (AC)6 | 151 | 145 | Echt et al. (this paper) | n/a |  |  |  | F primer was evaluated with a 5' dye-CACGACGTTGTAAAACGAC tail. R primer was evaluated with a 5' GTTTCTT tail. |  |
| PtSIFG\_4192 | not mapped | not mapped | not mapped | not mapped | gb|BV728725 | UniSTS:516346 | CCATTCGTGTATGCCAACAG | TAGCCTTGGGAAAGGGAAGT | SSR | CE | (AT)7 | 446 | 442 | Echt et al. (this paper) | n/a |  |  |  | F primer was evaluated with a 5' dye-CACGACGTTGTAAAACGAC tail. R primer was evaluated with a 5' GTTTCTT tail. |  |
| PtSIFG\_4202 | not mapped | not mapped | not mapped | not mapped | gb|BV728780 | UniSTS:516347 | AAGCAACTGCCAATTTGACC | TGGCTGAAATATGCTACCCC | SSR | CE | (AG)6 | 328 | 325 | Echt et al. (this paper) | n/a |  |  | F and R primers may be reversed from what is in the NCBI UniSTS record. | F primer was evaluated with a 5' dye-CACGACGTTGTAAAACGAC tail. R primer was evaluated with a 5' GTTTCTT tail. |  |
| PtSIFG\_4207 | not mapped | not mapped | not mapped | not mapped | gb|BV728718 | UniSTS:516348 | ACGGCCGTAGTTTTTCCTCT | AGTACCACAAGCAGCATCCC | SSR | CE | (AT)6 | 294 | 536 | Echt et al. (this paper) | n/a |  |  |  | F primer was evaluated with a 5' dye-CACGACGTTGTAAAACGAC tail. R primer was evaluated with a 5' GTTTCTT tail. |  |
| PtSIFG\_4213 | 8 | 66.9 | 19 | 0.94 | gb|BV728760 | UniSTS:516349 | AACGCAAACGCAACCTATCT | GGCACTCACCTGCATTCTTT | SSR | CE | (TA)6 | 201 | 199 | Echt et al. (this paper) | n/a |  |  | F and R primers may be reversed from what is in the NCBI UniSTS record. | F primer was evaluated with a 5' dye-CACGACGTTGTAAAACGAC tail. R primer was evaluated with a 5' GTTTCTT tail. |  |
| PtSIFG\_4218 | 1 | 54.4 | 18 | 1.3 | gb|BV728761 | UniSTS:516350 | AAAGGCAGCAGTCGGTAGAA | AAACCAAGTTTGCCTGATCG | SSR | CE | (TA)6 | 201 | 200 | Echt et al. (this paper) | n/a |  |  | F and R primers may be reversed from what is in the NCBI UniSTS record. | F primer was evaluated with a 5' dye-CACGACGTTGTAAAACGAC tail. R primer was evaluated with a 5' GTTTCTT tail. | Locus segregates a null allele. |
| PtSIFG\_4222 | 8 | 7.1 | 13 | 2.63 | gb|BV728762 | UniSTS:516351 | CACCCTTTTCACGCAAGAAT | GCCTACGCATTATCCTTCCA | SSR | CE | (AT)6 | 317 | 319 | Echt et al. (this paper) | n/a |  |  | F and R primers may be reversed from what is in the NCBI UniSTS record. | F primer was evaluated with a 5' dye-CACGACGTTGTAAAACGAC tail. R primer was evaluated with a 5' GTTTCTT tail. |  |
| PtSIFG\_4232 | not mapped | not mapped | not mapped | not mapped | gb|BV728785 | UniSTS:516352 | GAAAAAGAAGAGAAGAATCAACGC | CTTCAAATGCCCTTCGACAT | SSR | CE | (AT)7, (AT)8 | 267 | 265 | Echt et al. (this paper) | n/a |  |  | F and R primers may be reversed from what is in the NCBI UniSTS record. | F primer was evaluated with a 5' dye-CACGACGTTGTAAAACGAC tail. R primer was evaluated with a 5' GTTTCTT tail. |  |
| PtSIFG\_4233 | 7 | 60.7 | 13 | 1.02 | gb|BV728685 | UniSTS:516353 | AGGGAAACCGCGGATTATAG | CCGGAATGAAGATTGCAGTT | SSR | CE | (TA)7 | 102 | 96 | Echt et al. (this paper) | n/a |  |  |  | F primer was evaluated with a 5' dye-CACGACGTTGTAAAACGAC tail. R primer was evaluated with a 5' GTTTCTT tail. |  |
| PtSIFG\_4239 | not mapped | not mapped | not mapped | not mapped | gb|BV728777 | UniSTS:516354 | AGAGCGAAACCAGCAATACA | GAATGCTGCAGAGGAAGGAG | SSR | CE | (TA)6 | 214 | 209 | Echt et al. (this paper) | n/a |  |  | F and R primers may be reversed from what is in the NCBI UniSTS record. | F primer was evaluated with a 5' dye-CACGACGTTGTAAAACGAC tail. R primer was evaluated with a 5' GTTTCTT tail. |  |
| PtSIFG\_4245 | 3 | 26.2 | 19 | 0.345 | gb|BV728686 | UniSTS:516355 | TTCAAGGGGCAAGATTCAAC | TTTGCTCTCCAATTCCAACC | SSR | CE | (TA)7 | 418 | 414 | Echt et al. (this paper) | n/a |  |  |  | F primer was evaluated with a 5' dye-CACGACGTTGTAAAACGAC tail. R primer was evaluated with a 5' GTTTCTT tail. |  |
| PtSIFG\_4249 | 5 | 37.5 | 11 | 0.93 | gb|BV728687 | UniSTS:516356 | TCCTTGGTTTGTGCTTTTCC | ATATGCCCGTTGGCAGTAAC | SSR | CE | (AT)6 | 359 | 356 | Echt et al. (this paper) | n/a |  |  |  | F primer was evaluated with a 5' dye-CACGACGTTGTAAAACGAC tail. R primer was evaluated with a 5' GTTTCTT tail. |  |
| PtSIFG\_4282 | 11 | 47 | 12 | 0.25 | gb|BV728763 | UniSTS:516357 | AACAAAATTTATGGCGACCG | TTCTTGAGGTCGGTTGTTCC | SSR | CE | (AT)6 | 321 | 327 | Echt et al. (this paper) | n/a |  |  | F and R primers may be reversed from what is in the NCBI UniSTS record. | F primer was evaluated with a 5' dye-CACGACGTTGTAAAACGAC tail. R primer was evaluated with a 5' GTTTCTT tail. |  |
| PtSIFG\_4304 | 12 | 25.8 | 12 | 1.7 | gb|BV728793 | UniSTS:516358 | CATGCATGTGTGGAGGAGTT | CTCATGTGCTTTGATCCCCT | SSR | CE | (CT)6 | 394 | 393 | Echt et al. (this paper) | n/a |  | R primer matches nothing in pine NCBI dbEST. |  | F primer was evaluated with a 5' dye-CACGACGTTGTAAAACGAC tail. R primer was evaluated with a 5' GTTTCTT tail. |  |
| PtSIFG\_4315 | 6 | 86.3 | 15 | 0.65 | gb|BV728688 | UniSTS:516359 | GGCCTAGATCTTGTGGAAAGAA | TCCTTGTCACGCTGAGATTG | SSR | CE | (TA)8(TA)6 | 194 | 188 | Echt et al. (this paper) | n/a |  |  |  | F primer was evaluated with a 5' dye-CACGACGTTGTAAAACGAC tail. R primer was evaluated with a 5' GTTTCTT tail. |  |
| PtSIFG\_4378 | 3 | 34.2 | 30 | 1.11 | gb|BV728764 | UniSTS:516360 | TGGTGGTGGGGGATACTAAA | TATTACCGGCACACAACGAA | SSR | CE | (TA)6 | 263 | 262 | Echt et al. (this paper) | n/a |  |  | F and R primers may be reversed from what is in the NCBI UniSTS record. | F primer was evaluated with a 5' dye-CACGACGTTGTAAAACGAC tail. R primer was evaluated with a 5' GTTTCTT tail. |  |
| PtSIFG\_4380 | not mapped | not mapped | not mapped | not mapped | gb|BV728709 | UniSTS:516361 | CCTATCCCACAAAGACGGAA | AACTCAAAAACCTGGGGCTT | SSR | CE | (AT)6 | 420 | 418 | Echt et al. (this paper) | n/a |  |  |  | F primer was evaluated with a 5' dye-CACGACGTTGTAAAACGAC tail. R primer was evaluated with a 5' GTTTCTT tail. |  |
| PtSIFG\_4391 | not mapped | not mapped | not mapped | not mapped | gb|BV728707 | UniSTS:516363 | CAGACTGGCAGCAAAATGAA | GTAGGGCGTTGACAATCCAT | SSR | CE | (GA)6 | 175 | 171 | Echt et al. (this paper) | n/a |  |  |  | F primer was evaluated with a 5' dye-CACGACGTTGTAAAACGAC tail. R primer was evaluated with a 5' GTTTCTT tail. |  |
| PtSIFG\_4393 | not mapped | not mapped | not mapped | not mapped | gb|BV728778 | UniSTS:516364 | ATCGTTGCCTGCATGTTTTT | TCTTGCTGAATATCCCGGTC | SSR | CE | (AT)6 | 402 | 401 | Echt et al. (this paper) | n/a |  |  | F and R primers may be reversed from what is in the NCBI UniSTS record. | F primer was evaluated with a 5' dye-CACGACGTTGTAAAACGAC tail. R primer was evaluated with a 5' GTTTCTT tail. |  |
| PtSIFG\_4394 | 10 | 42.6 | 21 | 0.515 | gb|BV728765 | UniSTS:516365 | GCTTGCCAAGTCACTGTTGA | CTGCAGCCCTTTCTCAAAAC | SSR | CE | (AT)7 | 252 | 424 | Echt et al. (this paper) | n/a |  |  | F and R primers may be reversed from what is in the NCBI UniSTS record. | F primer was evaluated with a 5' dye-CACGACGTTGTAAAACGAC tail. R primer was evaluated with a 5' GTTTCTT tail. |  |
| PtSIFG\_4407 | not mapped | not mapped | not mapped | not mapped | gb|BV728712 | UniSTS:516366 | AAATACCCCTCCACCCATTT | TCATTGGCCTACTTTCCCAC | SSR | CE | (TC)6 | 181 | 176 | Echt et al. (this paper) | n/a |  |  |  | F primer was evaluated with a 5' dye-CACGACGTTGTAAAACGAC tail. R primer was evaluated with a 5' GTTTCTT tail. |  |
| PtSIFG\_4415 | 11 | 8.8 | 10 | 0.57 | gb|BV728689 | UniSTS:516367 | GCGCAAAGTGTCTGTGTGTT | AGGCATTACAGAAACACGGG | SSR | CE | (AT)6 | 340 | 339 | Echt et al. (this paper) | n/a |  |  |  | F primer was evaluated with a 5' dye-CACGACGTTGTAAAACGAC tail. R primer was evaluated with a 5' GTTTCTT tail. | Locus segregates a null allele. |
| PtSIFG\_4438 | 3 | 43.1 | 21 | 2.01 | gb|BV728690 | UniSTS:516368 | ACATAAGCACCGGTGAGGTC | CCTTGCTCTTATGCCTCCAA | SSR | CE | (TA)7 | 302 | 301 | Echt et al. (this paper) | n/a |  |  |  | F primer was evaluated with a 5' dye-CACGACGTTGTAAAACGAC tail. R primer was evaluated with a 5' GTTTCTT tail. | Locus segregates a 1bp allele. |
| PtSIFG\_4446 | 8 | 126.4 | 6 | 0.61 | gb|BV728691 | UniSTS:516369 | TCATGGCTTTGGACATGAAA | ATGGGGCTCAAGTGTACTGC | SSR | CE | (TC)6 | 131 | 115 | Echt et al. (this paper) | n/a |  |  |  | F primer was evaluated with a 5' dye-CACGACGTTGTAAAACGAC tail. R primer was evaluated with a 5' GTTTCTT tail. |  |
| PtSIFG\_4447 | 12 | 2.7 | 12 | 0.53 | gb|BV728692 | UniSTS:516370 | TTCTCGTTGGCTTCCTGAAT | AAATCCAGAATGAACCACATTT | SSR | CE | (AT)6 | 149 | 147 | Echt et al. (this paper) | n/a |  |  |  | F primer was evaluated with a 5' dye-CACGACGTTGTAAAACGAC tail. R primer was evaluated with a 5' GTTTCTT tail. |  |
| PtSIFG\_4454 | 2 | 7.7 | 18 | 0.42 | gb|BV728693 | UniSTS:516371 | CTTGCTATGCCAACCAGACA | CCCACACCAGCTCCATTTTA | SSR | CE | (AT)6 | 294 | 297 | Echt et al. (this paper) | n/a |  |  |  | F primer was evaluated with a 5' dye-CACGACGTTGTAAAACGAC tail. R primer was evaluated with a 5' GTTTCTT tail. |  |
| PtSIFG\_4458 | 4 | 113 | 8 | 2.39 | gb|BV728766 | UniSTS:516372 | GGATTTGATTCGATCCCCTT | GGTGCCCTTCAAATCAAAGA | SSR | CE | (AT)7 | 207 | 202 | Echt et al. (this paper) | n/a |  |  | F and R primers may be reversed from what is in the NCBI UniSTS record. | F primer was evaluated with a 5' dye-CACGACGTTGTAAAACGAC tail. R primer was evaluated with a 5' GTTTCTT tail. |  |
| PtSIFG\_4472 | 9 | 88.6 | 12 | 1.46 | gb|BV728694 | UniSTS:516373 | CCAAGAGAGTTGCCTTACGC | GGTCGTCCGCTAACAGAGAG | SSR | CE | (TA)6 | 253 | 252 | Echt et al. (this paper) | n/a |  |  |  | F primer was evaluated with a 5' dye-CACGACGTTGTAAAACGAC tail. R primer was evaluated with a 5' GTTTCTT tail. |  |
| PtSIFG\_4493 | 11 | 31.5 | 11 | 2.18 | gb|BV728767 | UniSTS:516374 | TTTGAACCTTTTTGGCTTGG | AACCCTCATTTGCTGCACTC | SSR | CE | (AT)6 | 487 | 489 | Echt et al. (this paper) | n/a |  |  | F and R primers may be reversed from what is in the NCBI UniSTS record. | F primer was evaluated with a 5' dye-CACGACGTTGTAAAACGAC tail. R primer was evaluated with a 5' GTTTCTT tail. |  |
| PtSIFG\_4502 | 5 | 17.9 | 6 | 26.7, jump>5 | gb|BV728695 | UniSTS:516375 | AGCGTAATCAACTGGGAACG | GTTCATTCATGCTCAGCGAA | SSR | CE | (AT)7 | 326 | 323 | Echt et al. (this paper) | n/a |  |  |  | F primer was evaluated with a 5' dye-CACGACGTTGTAAAACGAC tail. R primer was evaluated with a 5' GTTTCTT tail. | Locus segregates a null allele. |
| PtTX2037 | 2 | 73.9 | 20 | 1.91 | gb|BV728860 | UniSTS:508444 | GCCTTTAGATGAATGAACCCA | TAAGCGGGATATTATAGAGTTT | SSR | CE | (GTAG)8(GT)14 | 177 | 178 | Elsik et al. (2000) | 10902720 |  |  |  | F primer was evaluated with a 5' dye-CACGACGTTGTAAAACGAC tail. R primer was evaluated with a 5' GTTTCTT tail. |  |
| PtTX2080 | 12 | 89.1 | 5 | 1.75 | gb|BV728818 | UniSTS:508445 | AAAGATGGTCGGTTGTAAAGTT | TTGTCAGGCGGATAAGGTT | SSR | CE | (CAT)7 | 161 | 156 | Auckland et al. (2002) | n/a |  |  |  | F primer was evaluated with a 5' dye-CACGACGTTGTAAAACGAC tail. R primer was evaluated with a 5' GTTTCTT tail. | Locus segregates a null allele. |
| PtTX2091 | 8 | 64.1 | 25 | 2.48 | gb|BV728845 | UniSTS:508446 | ACCAAATCTCCCCACAT | AATCATACCCGTTTCAGT | SSR | CE | (GTTT)3TT(GTT)5, (GTT)7 | 267 | 267 | Auckland et al. (2002) | n/a |  |  |  | F primer was evaluated with a 5' dye-CACGACGTTGTAAAACGAC tail. R primer was evaluated with a 5' GTTTCTT tail. |  |
| PtTX2093 | 5 | 35.5 | 15 | 1.3 | gb|BV728819 | UniSTS:508447 | AATTTGACGGGTTTTAC | GTGGCACATGGATTTCT | SSR | CE | (CAA)9 | 343 | 343 | Auckland et al. (2002) | n/a |  |  |  | F primer was evaluated with a 5' dye-CACGACGTTGTAAAACGAC tail. R primer was evaluated with a 5' GTTTCTT tail. |  |
| PtTX2094 | 2 | 125.5 | 7 | 0.61 | gb|BV728820 | UniSTS:508448 | CAACTGTGCCTGTGCTGTGT | ATGGGTGGGTTGGTTATCTGA | SSR | CE | (TTTG)9(T)11 | 324 | 260 | Auckland et al. (2002) | n/a |  |  |  | F primer was evaluated with a 5' dye-CACGACGTTGTAAAACGAC tail. R primer was evaluated with a 5' GTTTCTT tail. |  |
| PtTX2123 | 9 | 105.6 | 12 | 0.81 | gb|BV728861 | UniSTS:508449 | GAAGAACCCACAAACACAAG | GGGCAAGAATTCAATGATAA | SSR | CE | (AGC)8 | 198 | 194 | Elsik et al. (2000) | 10902720 |  |  |  | F primer was evaluated with a 5' dye-CACGACGTTGTAAAACGAC tail. R primer was evaluated with a 5' GTTTCTT tail. |  |
| PtTX2146 | 3 | 82.4 | 39 | 7.42 | gb|BV728863 | UniSTS:508451 | CCTGGGGATTTGGATTGGGTATTTG | ATATTTTCCTTGCCCCTTCCAGACA | SSR | CE | (TGC)5, (TGC)7, (TGC)7 | 180 | 180 | Elsik et al. (2000) | 10902720 |  |  |  | F primer was evaluated with a 5' dye-CACGACGTTGTAAAACGAC tail. R primer was evaluated with a 5' GTTTCTT tail. |  |
| PtTX2164 | 3 | 18.3 | 27 | 1.47 | gb|BV728862 | UniSTS:508452 | TCAAATATTAAGAAGGTAACAATAC | GAAAATGAAAATCTTAAAAAAATTC | SSR | CE | (TCG)5, (CCG)4, (TCG)4, (TCG)5, (TCG)5(TCA)10 | 252 | 252 | Elsik et al. (2000) | 10902720 |  |  | F and R primers may be reversed from what is in the NCBI UniSTS record. | F primer was evaluated with a 5' dye-CACGACGTTGTAAAACGAC tail. R primer was evaluated with a 5' GTTTCTT tail. |  |
| PtTX2189 | 2 | 0.0 | 12 | 0.42 | gb|BV728821 | UniSTS:508453 | ATGAGCCTTTATTTATTGTTTTTG | ATAGGATTTAAGTAGTTTTTCATT | SSR | CE | (A)15(AACC)6 | 289 | 289 | Auckland et al. (2002) | n/a |  |  |  | F primer was evaluated with a 5' dye-CACGACGTTGTAAAACGAC tail. R primer was evaluated with a 5' GTTTCTT tail. |  |
| PtTX3\_lp3-1 | 5 | 34.9 | 16 | 1.063 | gb|BV728995 | UniSTS:516074 | GGAGGAGAAACAGCACCAC | CGGAAATCACACGAAAAGAA | ESTP | SSCP | n/a | 716 | n/a | Komulainen et al. (2003) | 12827250 | estPtTX3\_lp3-1 |  |  |  |  |
| PtTX3001 | 11 | 38.6 | 14 | 0.56 | gb|BV728822 | UniSTS:508454 | ATAAAGGCAGAGGATGAACA | CCCAATTGTTATTTCTGATT | SSR | CE | (CAA)4, (CAA)3, (CAA)4 | 313 | 300 | Auckland et al. (2002) | n/a |  |  |  | F primer was evaluated with a 5' dye-CACGACGTTGTAAAACGAC tail. R primer was evaluated with a 5' GTTTCTT tail. |  |
| PtTX3011 | 7 | 19.6 | 12 | 0.43 | gb|BV728852 | UniSTS:508455 | AATTTGGGTGTATTTTTCTTAGA | AAAAGTTGAAGGAGTTGGTGATC | SSR | CE | (GAT)15 | 186 | 185 | Elsik et al. (2000) | 10902720 |  |  |  | F primer was evaluated with a 5' dye-CACGACGTTGTAAAACGAC tail. R primer was evaluated with a 5' GTTTCTT tail. |  |
| PtTX3013 | 12 | 40.4 | 5 | 4.83 | gb|BV728853 | UniSTS:508456 | GCTTCTCCATTAACTAATTCTA | TCAAAATTGTTCGTAAAACCTC | SSR | CE | (GTT)10 | 134 | 128 | Elsik et al. (2000) | 10902720 |  |  |  | F primer was evaluated with a 5' dye-CACGACGTTGTAAAACGAC tail. R primer was evaluated with a 5' GTTTCTT tail. |  |
| PtTX3019 | 7 | 35.7 | 16 | 0.49 | gb|BV728864 | UniSTS:508457 | AAGAATATCAAGCACTCC | CAAAGGCATAAAGAAACT | SSR | CE | (CAA)10 | 208 | 215 | Elsik et al. (2000) | 10902720 |  |  |  | F primer was evaluated with a 5' dye-CACGACGTTGTAAAACGAC tail. R primer was evaluated with a 5' GTTTCTT tail. |  |
| PtTX3020 | 3 | 99.4 | 24 | 0.63 | gb|BV728854 | UniSTS:508458 | GTCGGGGAAGTGAAAGTA | CTAGGTGCAAGAAAAGAGTAT | SSR | CE | (A)16(CAA)9 | 211 | 211 | Elsik et al. (2000) | 10902720 |  |  |  | F primer was evaluated with a 5' dye-CACGACGTTGTAAAACGAC tail. R primer was evaluated with a 5' GTTTCTT tail. |  |
| PtTX3025 | 2 | 7.9 | 12 | 0.56 | gb|BV728855 | UniSTS:508459 | CACGCTGTATAATAACAATCTA | TTCTATATTCGCTTTTAGTTTC | SSR | CE | (CAA)10 | 266 | 260 | Elsik et al. (2000) | 10902720 |  |  |  | F primer was evaluated with a 5' dye-CACGACGTTGTAAAACGAC tail. R primer was evaluated with a 5' GTTTCTT tail. |  |
| PtTX3027 | 4 | 81.4 | 6 | 8.56 | gb|BV728823 | UniSTS:508460 | TCCATTTGAGAACTTTTT | AGGAGCCACAACATAATA | SSR | CE | (CAT)10 | 280 | 280 | Auckland et al. (2002) | n/a |  |  |  | F primer was evaluated with a 5' dye-CACGACGTTGTAAAACGAC tail. R primer was evaluated with a 5' GTTTCTT tail. |  |
| PtTX3029 | 5 | 44 | 23 | 0.34 | gb|BV728824 | UniSTS:508461 | CTTGTTGCTGCTTCTGC | AACAAAATAATATAAATGCTCTGC | SSR | CE | (GCT)8 | 255 | 259 | Auckland et al. (2002) | n/a |  |  |  | F primer was evaluated with a 5' dye-CACGACGTTGTAAAACGAC tail. R primer was evaluated with a 5' GTTTCTT tail. |  |
| PtTX3032 | 8 | 45.5 | 17 | 0.69 | gb|BV728856 | UniSTS:508462 | CTGCCACACTACCAACC | AACATTAAGATCTCATTTCAA | SSR | CE | (ACT)6, (GTC)6, (GTC)8, (TCA)36 | 335 | 330 | Elsik et al. (2000) | 10902720 |  |  |  | F primer was evaluated with a 5' dye-CACGACGTTGTAAAACGAC tail. R primer was evaluated with a 5' GTTTCTT tail. |  |
| PtTX3034 | 8 | 102.7 | 24 | 2.09, jump>5 | gb|BV728857 | UniSTS:508463 | TCAAAATGCAAAAGACG | ATTAGGACTGGGGATGAT | SSR | CE | (GA)13 | 207 | 201 | Elsik et al. (2000) | 10902720 |  |  |  | F primer was evaluated with a 5' dye-CACGACGTTGTAAAACGAC tail. R primer was evaluated with a 5' GTTTCTT tail. |  |
| PtTX3037 | 9 | 123.7 | 9 | 0.63 | gb|BV728858 | UniSTS:508464 | CGTTTGGAGCACTACTT | AAGTCACTTAATGCAATATGTA | SSR | CE | (CAA)15 | 144 | 110 | Elsik et al. (2000) | 10902720 |  |  |  | F primer was evaluated with a 5' dye-CACGACGTTGTAAAACGAC tail. R primer was evaluated with a 5' GTTTCTT tail. |  |
| PtTX3045 | 6 | 101.9 | 7 | 0.2 | gb|BV728846 | UniSTS:508465 | CATCGCATATCGCAATCAGG | AAGGCAAGAGGGAAATGTAATAGA | SSR | CE | (CA)12 | 256 | 255 | Auckland et al. (2002) | n/a |  |  |  | F primer was evaluated with a 5' dye-CACGACGTTGTAAAACGAC tail. R primer was evaluated with a 5' GTTTCTT tail. | Locus segregates a null allele. |
| PtTX3047 | 10 | 43.8 | 21 | 0.860 | gb|BV728825 | UniSTS:508466 | TTGGAATACTTGCACGATGAC | ATTTAGATAGGAGATGGTTGTTTA | SSR | CE | (AC)21 | 354 | 347 | Auckland et al. (2002) | n/a |  |  |  | F primer was evaluated with a 5' dye-CACGACGTTGTAAAACGAC tail. R primer was evaluated with a 5' GTTTCTT tail. |  |
| PtTX3049 | 5 | 70.5 | 28 | 1.98 | gb|BV728826 | UniSTS:508467 | GAAGTGATAATGGCATAGCAAAAT | CAGACCCGTGAAAGTAATAAACAT | SSR | CE | (TG)16 | 311 | 300 | Auckland et al. (2002) | n/a |  |  |  | F primer was evaluated with a 5' dye-CACGACGTTGTAAAACGAC tail. R primer was evaluated with a 5' GTTTCTT tail. | Locus segregates a null allele. |
| PtTX3052 | 11 | 7.6 | 9 | 0.21 | gb|BV728827 | UniSTS:508468 | CCTCACTAGGAGGCTACGGAAGAG | AAAGACTCCTTGATGTTGTGAACA | SSR | CE | (ATC)8 | 242 | 240 | Auckland et al. (2002) | n/a |  |  |  | F primer was evaluated with a 5' dye-CACGACGTTGTAAAACGAC tail. R primer was evaluated with a 5' GTTTCTT tail. |  |
| PtTX3055 | 6 | 5.4 | 5 | 1.1 | gb|BV728828 | UniSTS:508469 | AGCAGACTTGAAGGGAAAAA | ATCATCTATATTACCAGGGAGTT | SSR | CE | (GAT)5, (GAT)8, (GAT)6 | 402 | 402 | Auckland et al. (2002) | n/a |  |  |  | F primer was evaluated with a 5' dye-CACGACGTTGTAAAACGAC tail. R primer was evaluated with a 5' GTTTCTT tail. |  |
| PtTX3058 | 1 | 98.2 | 22 | 1.63 | gb|BV728829 | UniSTS:508470 | ACTCTACTACTTGTTCTACCTCA | ATTATATTTCGGCATTGT | SSR | CE | (CAA)8, (CAA)4, (CAA)3, (CAA)8, (CAA)5 | 423 | 250 | Auckland et al. (2002) | n/a |  |  |  | F primer was evaluated with a 5' dye-CACGACGTTGTAAAACGAC tail. R primer was evaluated with a 5' GTTTCTT tail. |  |
| PtTX3063 | 11 | 35.2 | 26 | 0.45 | gb|BV728830 | UniSTS:508471 | CAATCAGAATCAGCGGCAAACAAA | TTCAACAACATTCATCACACTA | SSR | CE | (CAA)15 | 268 | 259 | Auckland et al. (2002) | n/a |  |  |  | F primer was evaluated with a 5' dye-CACGACGTTGTAAAACGAC tail. R primer was evaluated with a 5' GTTTCTT tail. |  |
| PtTX3081 | 9 | 108.13 | 18 | 0.28 | gb|BV728831 | UniSTS:508472 | GCCGAGGAAGCAAGCAACCAA | CCTCGGCAGCCAAATCCTTCA | SSR | CE | (GTT)13 | 234 | 237 | Auckland et al. (2002) | n/a |  |  |  | F primer was evaluated with a 5' dye-CACGACGTTGTAAAACGAC tail. R primer was evaluated with a 5' GTTTCTT tail. |  |
| PtTX3097 | 5 | 102.6 | 6 | 0.23 | gb|BV728832 | UniSTS:508474 | TTTTTAGAAGATGATTGGATA | TCATATAGTAGCATCAAAACAAAT | SSR | CE | (GTT)7 | 134 | 172 | Auckland et al. (2002) | n/a |  |  |  | F primer was evaluated with a 5' dye-CACGACGTTGTAAAACGAC tail. R primer was evaluated with a 5' GTTTCTT tail. |  |
| PtTX3105 | 3 | 117.0 | 24 | 0.558 | gb|BV728847 | UniSTS:508475 | TGTCGGTGGAGTTGGCAGTAGACT | AGGGCCCAGCGTTTCCTG | SSR | CE | (GTT)9 | 201 | 180 | Auckland et al. (2002) | n/a |  |  |  | F primer was evaluated with a 5' dye-CACGACGTTGTAAAACGAC tail. R primer was evaluated with a 5' GTTTCTT tail. |  |
| PtTX3107 | 5 | 34.6 | 15 | 1.63 | gb|BV728833 | UniSTS:508476 | AAACAAGCCCACATCGTCAATC | TCCCCTGGATCTGAGGA | SSR | CE | (CAT)14 | 179 | 182 | Auckland et al. (2002) | n/a |  |  |  | F primer was evaluated with a 5' dye-CACGACGTTGTAAAACGAC tail. R primer was evaluated with a 5' GTTTCTT tail. |  |
| PtTX3110 | 4 | 63.3 | 20 | 2.46, jump>5 | gb|BV728834 | UniSTS:508477 | CTCCTAGGACTTTCTTTGTTG | TGGGGTGGAGGAGGAATCATA | SSR | CE | (GTT)8, (CTT)8 | 286 | 287 | Auckland et al. (2002) | n/a |  |  |  | F primer was evaluated with a 5' dye-CACGACGTTGTAAAACGAC tail. R primer was evaluated with a 5' GTTTCTT tail. | Locus segregates a null allele. |
| PtTX3114 | 8 | 0.0 | 13 | 0.97 | gb|BV728835 | UniSTS:508478 | CATATAAATTTGTGAGGTA | CATTATGTCCATTTAGTCC | SSR | CE | (GTT)11(T)17 | 189 | 180 | Auckland et al. (2002) | n/a |  |  |  | F primer was evaluated with a 5' dye-CACGACGTTGTAAAACGAC tail. R primer was evaluated with a 5' GTTTCTT tail. |  |
| PtTX3116 | 3 | 23.3 | 36 | 2.1, jump>5 | gb|BV728848 | UniSTS:508479 | CCTCCCAAAGCCTAAAGAAT | CATACAAGGCCTTATCTTACAGAA | SSR | CE | (TTG)7 | 141 | 126 | Auckland et al. (2002) | n/a |  |  |  | F primer was evaluated with a 5' dye-CACGACGTTGTAAAACGAC tail. R primer was evaluated with a 5' GTTTCTT tail. | Locus segregates a 1bp allele. |
| PtTX3117 | 11 | 92.2 | 14 | 0.35 | gb|BV728836 | UniSTS:508480 | GTGATTGATGAGGAGGCTTACT | TAGGGACTGGCACCGATGAA | SSR | CE | (CAT)9 | 208 | 196 | Auckland et al. (2002) | n/a |  |  |  | F primer was evaluated with a 5' dye-CACGACGTTGTAAAACGAC tail. R primer was evaluated with a 5' GTTTCTT tail. |  |
| PtTX3120 | 3 | 88.5 | 40 | 0.785 | gb|BV728837 | UniSTS:508482 | CCCACAAACAAGGAGGTC | TAGCAGTCGAGTTAGAAGATTAGA | SSR | CE | (CAA)25 | 282 | 236 | Auckland et al. (2002) | n/a |  |  |  | F primer was evaluated with a 5' dye-CACGACGTTGTAAAACGAC tail. R primer was evaluated with a 5' GTTTCTT tail. |  |
| PtTX4001 | 4 | 55.5 | 23 | 1.22, jump>5 | gb|BV728865 | UniSTS:508484 | CTATTTGAGTTAAGAAGGGAGTC | CTGTGGGTAGCATCATC | SSR | CE | (GT)15 | 220 | 220 | Zhou et al. (2002) | 11908673 |  |  |  | F primer was evaluated with a 5' dye-CACGACGTTGTAAAACGAC tail. R primer was evaluated with a 5' GTTTCTT tail. |  |
| PtTX4003 | 5 | 11.3 | 16 | 6.77, jump>5 | gb|BV728866 | UniSTS:508485 | GCGATAAGCATACCTACACT | TTACTAAAATGGGGATGAAA | SSR | CE | (GT)13 | 156 | 150 | Zhou et al. (2002) | 11908673 |  |  |  | F primer was evaluated with a 5' dye-CACGACGTTGTAAAACGAC tail. R primer was evaluated with a 5' GTTTCTT tail. | Locus segregates a null allele. |
| PtTX4030 | 1 | 55.1 | 27 | 1.24 | gb|BV728838 | UniSTS:508487 | TTGGGAGGATGACTCCATTATAT | ACTATGGTTGGTCAAGTTAA | SSR | CE | (GT)21(GA)13 | 150 | 150 | Auckland et al. (2002) | n/a |  |  |  | F primer was evaluated with a 5' dye-CACGACGTTGTAAAACGAC tail. R primer was evaluated with a 5' GTTTCTT tail. |  |
| PtTX4033 | 2 | 84.9 | 26 | 2.21 | gb|BV728878 | UniSTS:508488 | ACCCATTTCCTTTTTCAAC | GGTGGCGAGGCATTATTC | SSR | CE | (CA)15 | 146 | 156 | Zhou et al. (2002) | 11908673 |  |  |  | F primer was evaluated with a 5' dye-CACGACGTTGTAAAACGAC tail. R primer was evaluated with a 5' GTTTCTT tail. |  |
| PtTX4036 | 2 | 35.2 | 20 | 0.63 | gb|BV728879 | UniSTS:508489 | TGATGGGAAGGAAAAGAATAAAC | AATGCCCCCACAACTAAAAC | SSR | CE | (CA)31 | 458 | 470 | Zhou et al. (2002) | 11908673 |  |  |  | F primer was evaluated with a 5' dye-CACGACGTTGTAAAACGAC tail. R primer was evaluated with a 5' GTTTCTT tail. |  |
| PtTX4056 | 7 | 66.3 | 6 | 0.55 | gb|BV728867 | UniSTS:508490 | TTAAGGCCAGTTCCAATACAAAAT | GAGCCCAACAACTAAAACAATGAG | SSR | CE | (GA)17 | 432 | 412 | Zhou et al. (2002) | 11908673 |  |  |  | F primer was evaluated with a 5' dye-CACGACGTTGTAAAACGAC tail. R primer was evaluated with a 5' GTTTCTT tail. |  |
| PtTX4058 | 5 | 54.9 | 35 | 0.96 | gb|BV728868 | UniSTS:508491 | AAGTGTTGGGAGAAAAATGTAAT | CTCCTTCTGTCCCTATCCTCT | SSR | CE | (GA)20 | 141 | 140 | Zhou et al. (2002) | 11908673 |  |  |  | F primer was evaluated with a 5' dye-CACGACGTTGTAAAACGAC tail. R primer was evaluated with a 5' GTTTCTT tail. |  |
| PtTX4062 | 6 | 78.8 | 23 | 2.06 | gb|BV728877 | UniSTS:508492 | TCTAGGCAATCTTTTTACCAAC | TATCATAGCCTCATCCAATACA | SSR | CE | (GT)13 | 260 | 161 | Zhou et al. (2002) | 11908673 |  |  |  | F primer was evaluated with a 5' dye-CACGACGTTGTAAAACGAC tail. R primer was evaluated with a 5' GTTTCTT tail. |  |
| PtTX4079 | 9 | 64 | 18 | 0.77 | gb|BV728839 | UniSTS:508493 | CACATTTCCCTCCAACTAAAC | GGGCATAATAGCTGGTTCTAA | SSR | CE | (CA)23 | 242 | 224 | Auckland et al. (2002) | n/a |  |  |  | F primer was evaluated with a 5' dye-CACGACGTTGTAAAACGAC tail. R primer was evaluated with a 5' GTTTCTT tail. | Locus segregates a null allele. |
| PtTX4090 | 7 | 15.5 | 13 | 0.36 | gb|BV728869 | UniSTS:508494 | ACTTTCAAGATTCACTAATG | AGTCCAGCACTCCAAGAAA | SSR | CE | (CTT)6, (CTT)8 | 188 | 190 | Zhou et al. (2002) | 11908673 |  |  |  | F primer was evaluated with a 5' dye-CACGACGTTGTAAAACGAC tail. R primer was evaluated with a 5' GTTTCTT tail. |  |
| PtTX4092 | 10 | 62.7 | 19 | 0.675 | gb|BV728870 | UniSTS:508495 | GGATGATACTTTCCATGAGTTAGG | TCTAGTCCAGATCTTGGTCCAC | SSR | CE | (GAA)21 | 158 | 155 | Zhou et al. (2002) | 11908673 |  |  |  | F primer was evaluated with a 5' dye-CACGACGTTGTAAAACGAC tail. R primer was evaluated with a 5' GTTTCTT tail. |  |
| PtTX4093 | 8 | 77.9 | 17 | 7.51 | gb|BV728871 | UniSTS:508496 | TTGCTTTGCTAATGTTGACCTG | CTAGAGTATGCCTTGAGC | SSR | CE | (CTT)16 | 322 | 305 | Zhou et al. (2002) | 11908673 |  |  |  | F primer was evaluated with a 5' dye-CACGACGTTGTAAAACGAC tail. R primer was evaluated with a 5' GTTTCTT tail. |  |
| PtTX4112 | 7 | 64.5 | 14 | 1.49 | gb|BV728872 | UniSTS:508497 | CCCTCTGTTAGCCGATGTA | AATGTTAGCCCTAGATGTTTGATG | SSR | CE | (AT)6(GT)16 | 446 | 465 | Zhou et al. (2002) | 11908673 |  |  |  | F primer was evaluated with a 5' dye-CACGACGTTGTAAAACGAC tail. R primer was evaluated with a 5' GTTTCTT tail. |  |
| PtTX4137 | 6 | 55.6 | 24 | 1 | gb|BV728873 | UniSTS:508499 | CATTGTATTAGTCCTAGCCTCTGT | GGTGCACCCAACAATGTG | SSR | CE | (GAA)21 | 188 | 178 | Zhou et al. (2002) | 11908673 |  |  |  | F primer was evaluated with a 5' dye-CACGACGTTGTAAAACGAC tail. R primer was evaluated with a 5' GTTTCTT tail. |  |
| PtTX4147 | 5 | 9.7 | 13 | 5.58 | gb|BV728840 | UniSTS:508500 | CTCCGGACAAGAGCACAGGACTC | AGCTGGGTTTGGGACCATTCAC | SSR | CE | (CAA)5 | 193 | 190 | Auckland et al. (2002) | n/a |  |  |  | F primer was evaluated with a 5' dye-CACGACGTTGTAAAACGAC tail. R primer was evaluated with a 5' GTTTCTT tail. |  |
| PtTX4181 | 1 | 70 | 24 | 1.29 | gb|BV728850 | UniSTS:508502 | CTCTCCCCTTTATTTACACATTG | AAAGATTGGTCGGTTGGTTAT | SSR | CE | (CAA)6 | 388 | 375 | Auckland et al. (2002) | n/a |  |  |  | F primer was evaluated with a 5' dye-CACGACGTTGTAAAACGAC tail. R primer was evaluated with a 5' GTTTCTT tail. |  |
| PtTX4214 | 5 | 80 | 33 | 1.53 | gb|BV728841 | UniSTS:508503 | AACATTTCCCAAGCCTCAA | ACATGGACATCAAGAAGAAGTG | SSR | CE | (CA)20 | 164 | 170 | Auckland et al. (2002) | n/a |  |  |  | F primer was evaluated with a 5' dye-CACGACGTTGTAAAACGAC tail. R primer was evaluated with a 5' GTTTCTT tail. |  |
| PtTX4215 | 11 | 54 | 12 | 0.25 | gb|BV728842 | UniSTS:508504 | CTAATCCTTCTCCTCCTCTTG | CCTATCTCTGGGGTTTGT | SSR | CE | (CA)25 | 111 | 295 | Auckland et al. (2002) | n/a |  |  |  | F primer was evaluated with a 5' dye-CACGACGTTGTAAAACGAC tail. R primer was evaluated with a 5' GTTTCTT tail. |  |
| PtTX4228 | 3 | 113.9 | 16 | 0.554 | gb|BV728843 | UniSTS:508505 | ATATCATGTTTAGGTTGGTGTG | AGTTAGGCTTTTTGTCC | SSR | CE | (CA)14 | 158 | 156 | Auckland et al. (2002) | n/a |  |  |  | F primer was evaluated with a 5' dye-CACGACGTTGTAAAACGAC tail. R primer was evaluated with a 5' GTTTCTT tail. |  |
| PtWS1\_PCBER-PT1 | 8 | 2.4 | 12 | 21.099, jump>5 | n/a | n/a | GAGTTCGGGAATGATGTTGA | CGACGGTGGTGTATTTCACA | ESTP | SSCP | n/a | n/a | n/a | Komulainen et al. (2003) | 12827250 | PCBR\_pF1R1 |  |  |  |  |
| RPtest11 | not mapped | not mapped | not mapped | not mapped | gb|BV728796 | UniSTS:516377 | AGGATGCCTATGATATGCGC | AACCATAACAAAAGCGGTCG | SSR | CE | (ATC)7 | 213 | 216 | Chagne et al. (2004) | 15448894 |  |  |  | F primer was evaluated with a 5' dye-CACGACGTTGTAAAACGAC tail. R primer was evaluated with a 5' GTTTCTT tail. |  |
| RPtest5 | not mapped | not mapped | not mapped | not mapped | gb|BV728798 | UniSTS:516379 | ACAACAATAATAACGGGGGC | ACGCTTTAGATCCTCCTGCA | SSR | CE | (AAC)6 | 197 | 199 | Chagne et al. (2004) | 15448894 |  |  |  | F primer was evaluated with a 5' dye-CACGACGTTGTAAAACGAC tail. R primer was evaluated with a 5' GTTTCTT tail. |  |
| SsrPt\_ctg1376 | 8 | 112.2 | 20 | 12.6, jump>5 | gb|BV728806 | UniSTS:516387 | CGATATTATGGATTTTGCTTGTGA | AAATGCATGCCAAACTTAAATAC | SSR | CE | (AT)20 | 145 | 128 | Chagne et al. (2004) | 15448894 |  |  |  | F primer was evaluated with a 5' dye-CACGACGTTGTAAAACGAC tail. R primer was evaluated with a 5' GTTTCTT tail. |  |
| SsrPt\_ctg7141 | 3 | 80.3 | 40 | 0.964 | gb|BV728811 | UniSTS:516393 | GAATGACGCATTATCAGGGG | TCACCTTTCTCACCTCTGCC | SSR | CE | (CCG)8 | 375 | 475 | Chagne et al. (2004) | 15448894 |  | Duplicates UniSTS marker SsrPt\_ctg4487b and SsrPt\_ctg4487a. |  | F primer was evaluated with a 5' dye-CACGACGTTGTAAAACGAC tail. R primer was evaluated with a 5' GTTTCTT tail. |  |
| SsrPt\_ctg7444 | 9 | 84.9 | 20 | 1.64 | gb|BV728812 | UniSTS:516395 | TCTTCACCATCGGTTTCTCC | TGGATCTGTCACCTCCTCATC | SSR | CE | (AT)10 | 280 | 275 | Chagne et al. (2004) | 15448894 |  | Duplicates UniSTS marker PtSIFG\_1120, estPtIFG\_AGP-3. |  | F primer was evaluated with a 5' dye-CACGACGTTGTAAAACGAC tail. R primer was evaluated with a 5' GTTTCTT tail. |  |
| SsrPt\_ctg9249 | 5 | 20.1 | 25 | 5.35, jump>5 | gb|BV728813 | UniSTS:516396 | CTGCTCCCTCAGCTCTTCC | AGACGTCACTGCCATTACCC | SSR | CE | (AAG)7 | 156 | 150 | Chagne et al. (2004) | 15448894 |  | Duplicates UniSTS marker PtSIFG\_1186. |  | F primer was evaluated with a 5' dye-CACGACGTTGTAAAACGAC tail. R primer was evaluated with a 5' GTTTCTT tail. |  |
| SsrPt\_ctg946 | 10 | 78.9 | 15 | 1.749 | gb|BV728814 | UniSTS:516397 | TATCAGGTATAGGCCTCCGC | AAATAGGAGCCCTTCTGGGA | SSR | CE | (AGG)9 | 281 | 274 | Chagne et al. (2004) | 15448894 |  |  |  | F primer was evaluated with a 5' dye-CACGACGTTGTAAAACGAC tail. R primer was evaluated with a 5' GTTTCTT tail. |  |

Compiled by C. Echt et al., January 2011, USDA Forest Service.
