# Supplementary figures and images for: An annotated genetic map of loblolly pine based on microsatellite and cDNA markers
Source: BMC Genet. 2011 Jan 26;12:17. doi: 10.1186/1471-2156-12-17 (PMC3038140; doi:10.1186/1471-2156-12-17)

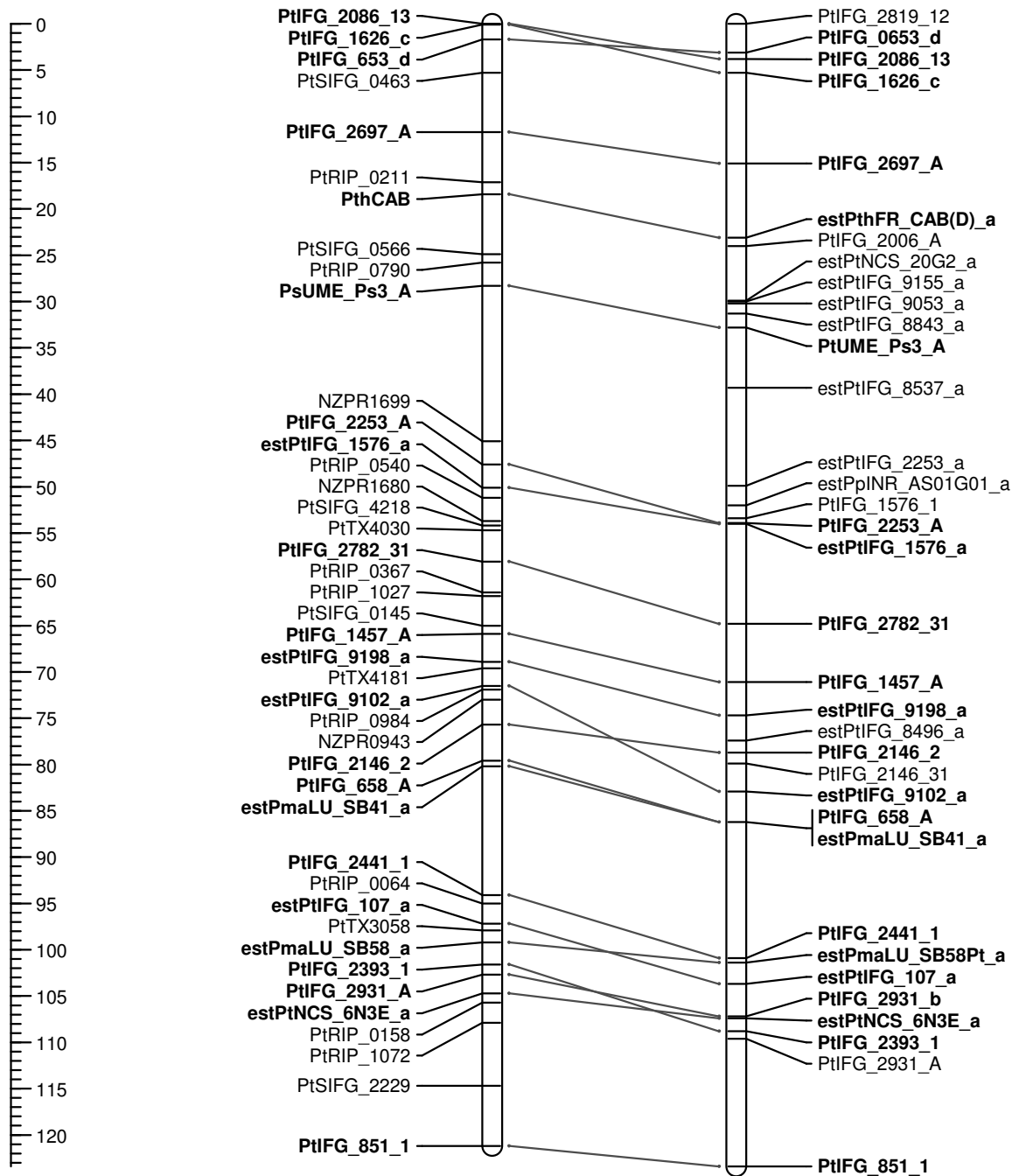

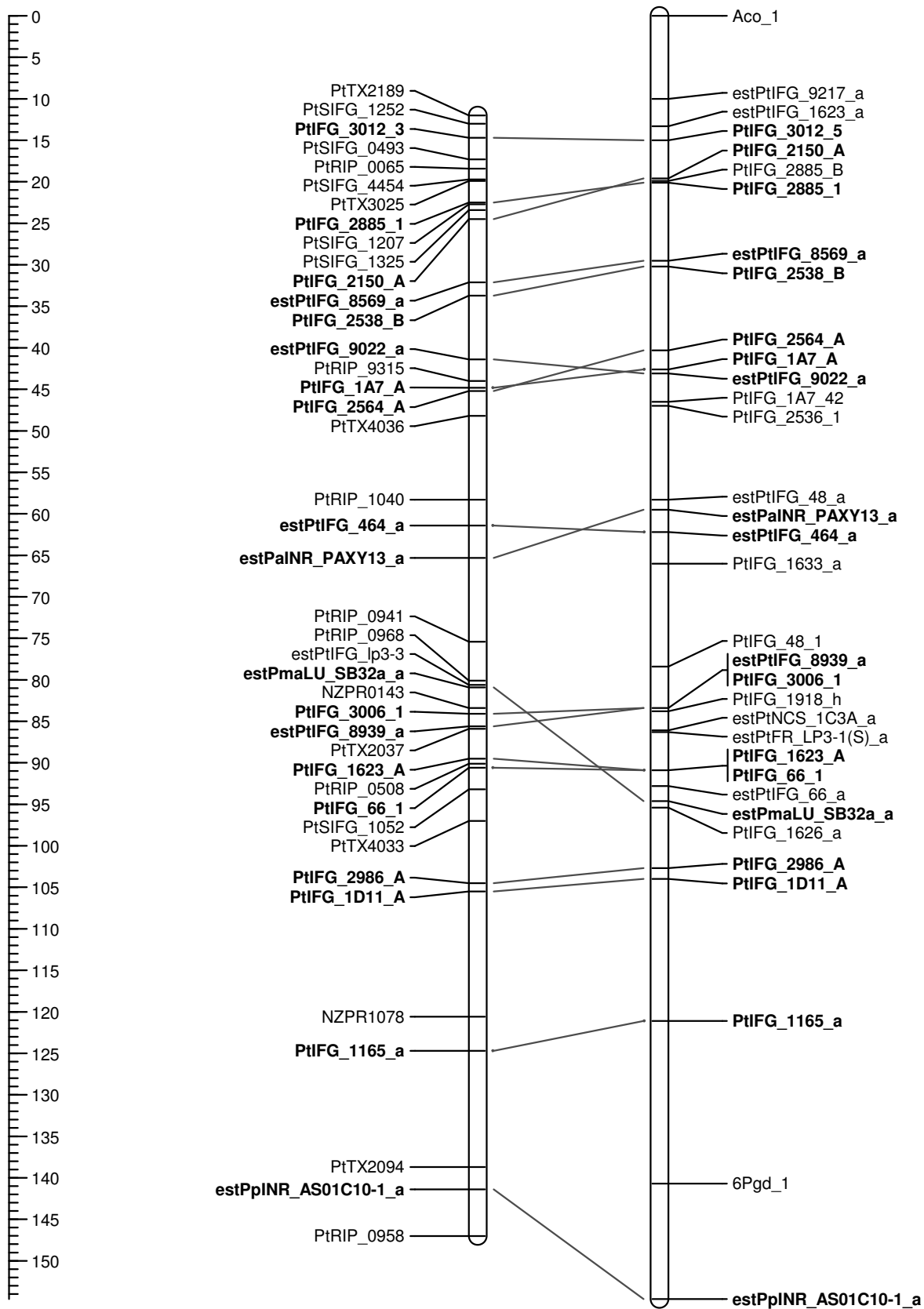

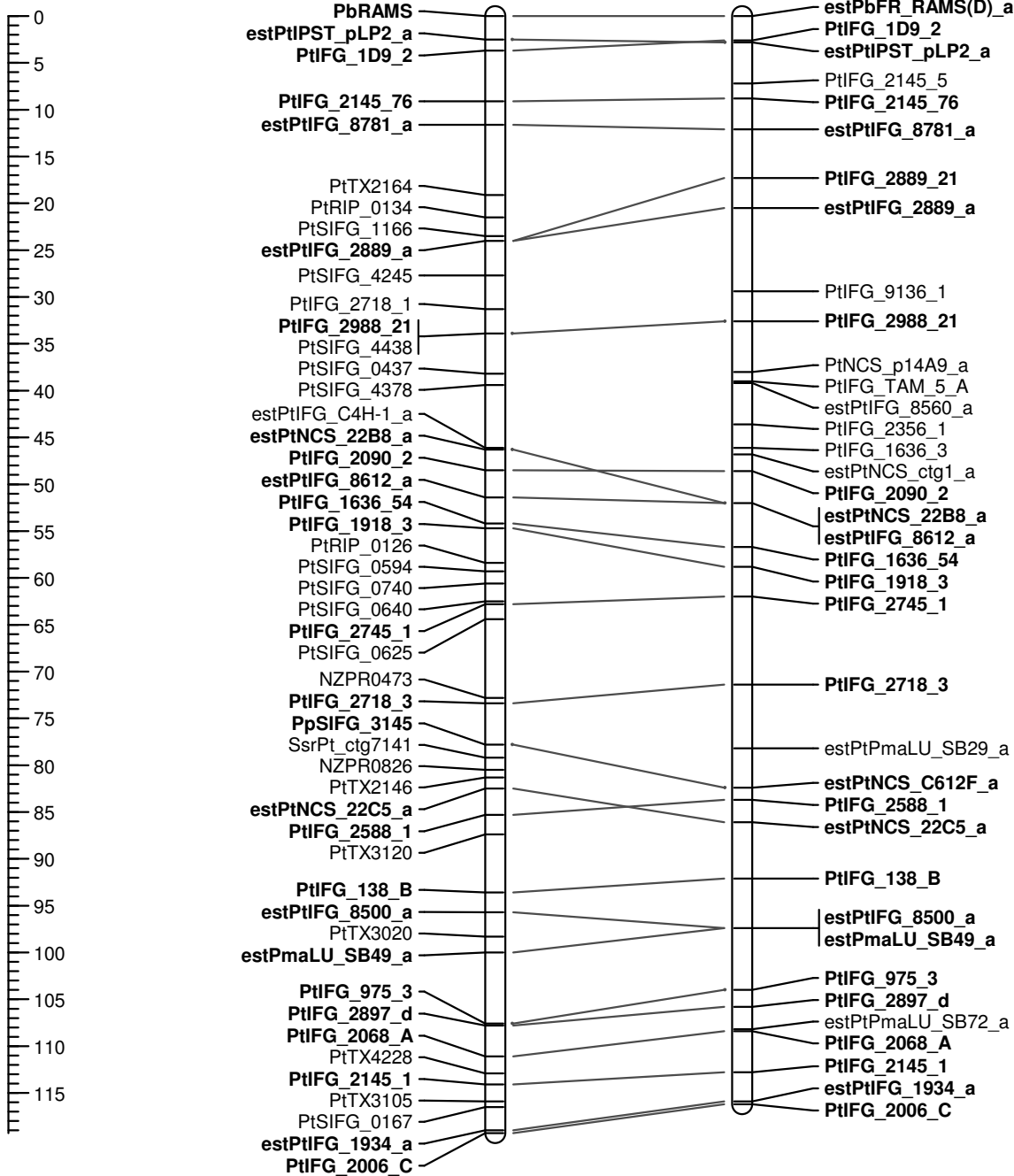

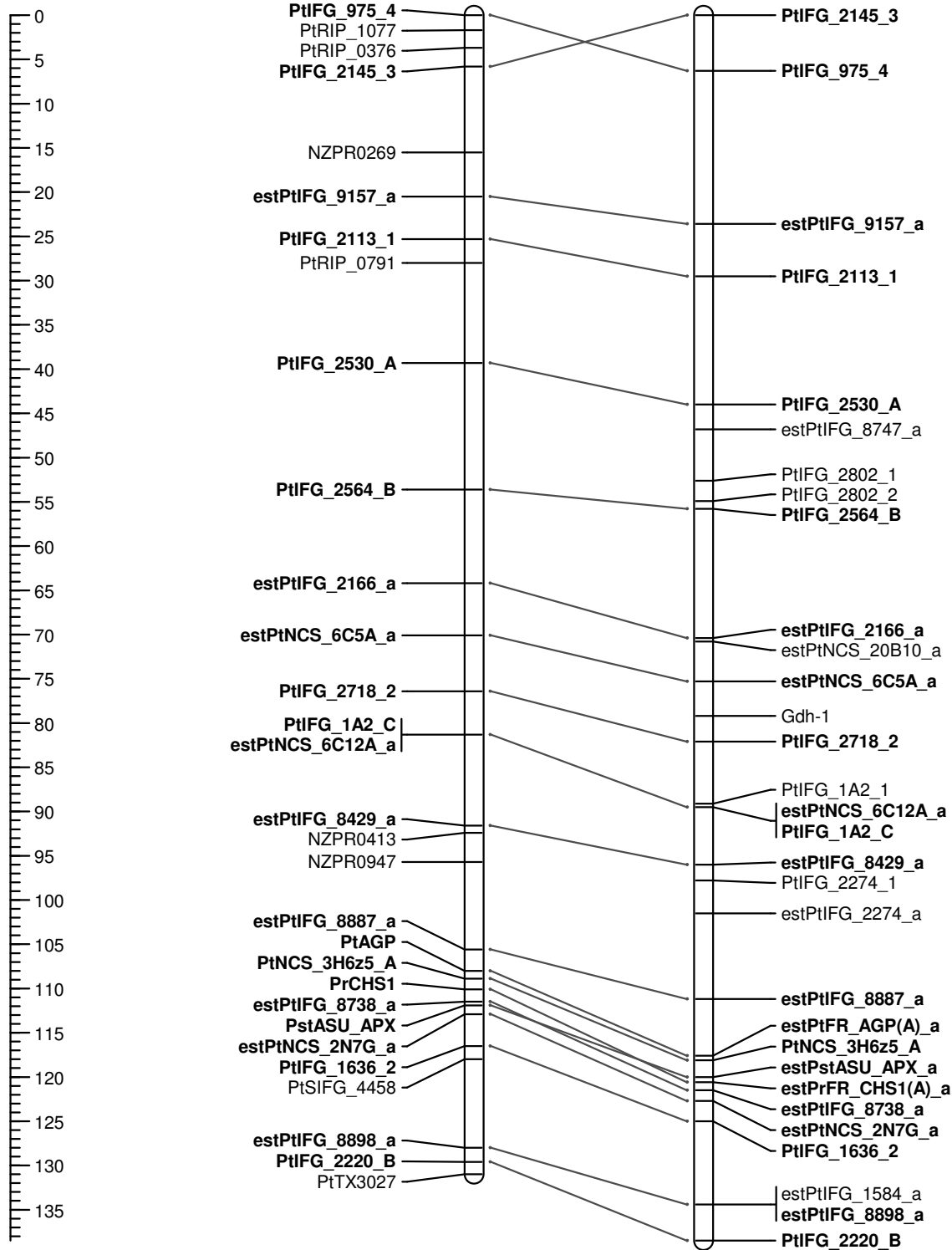

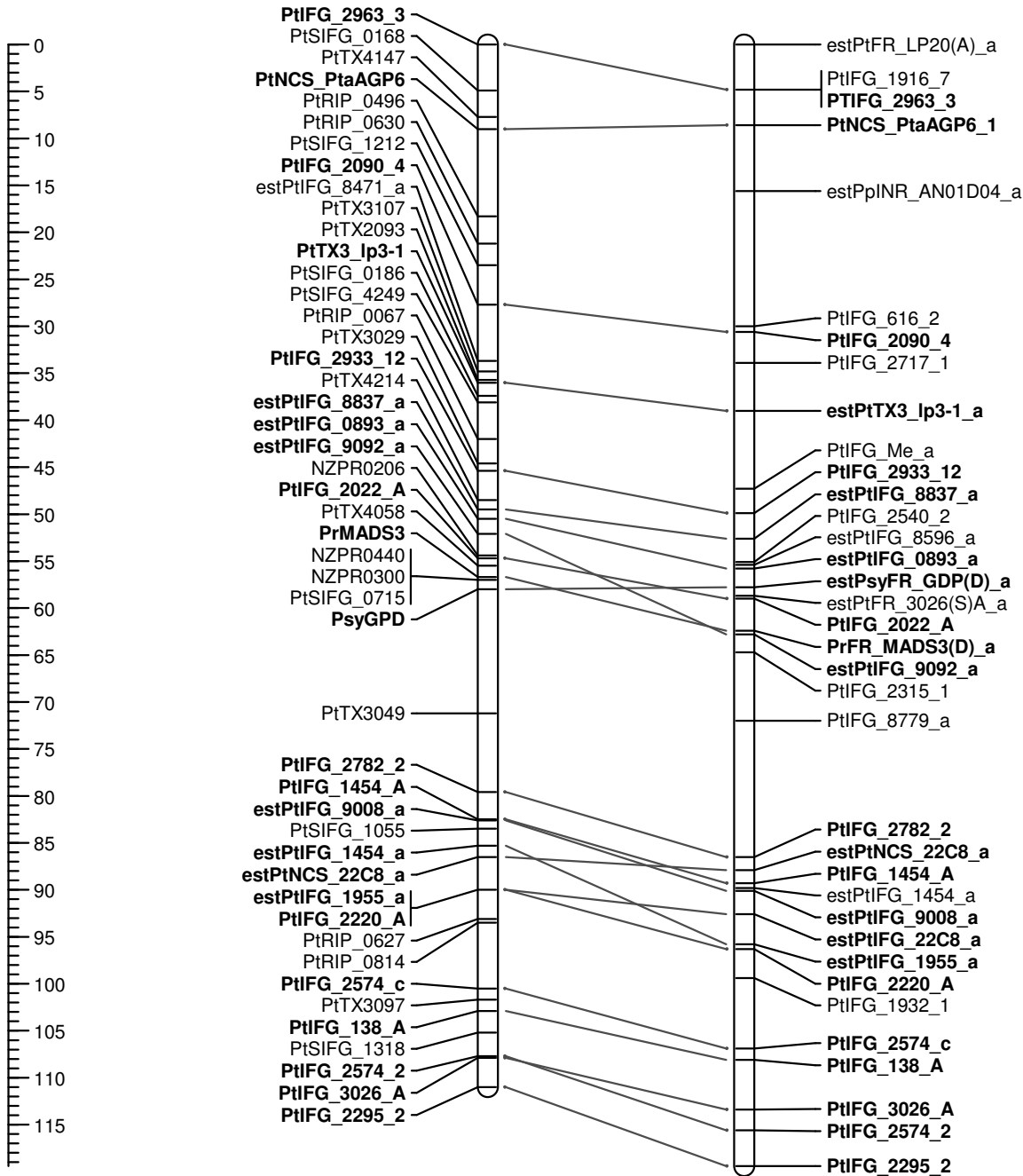

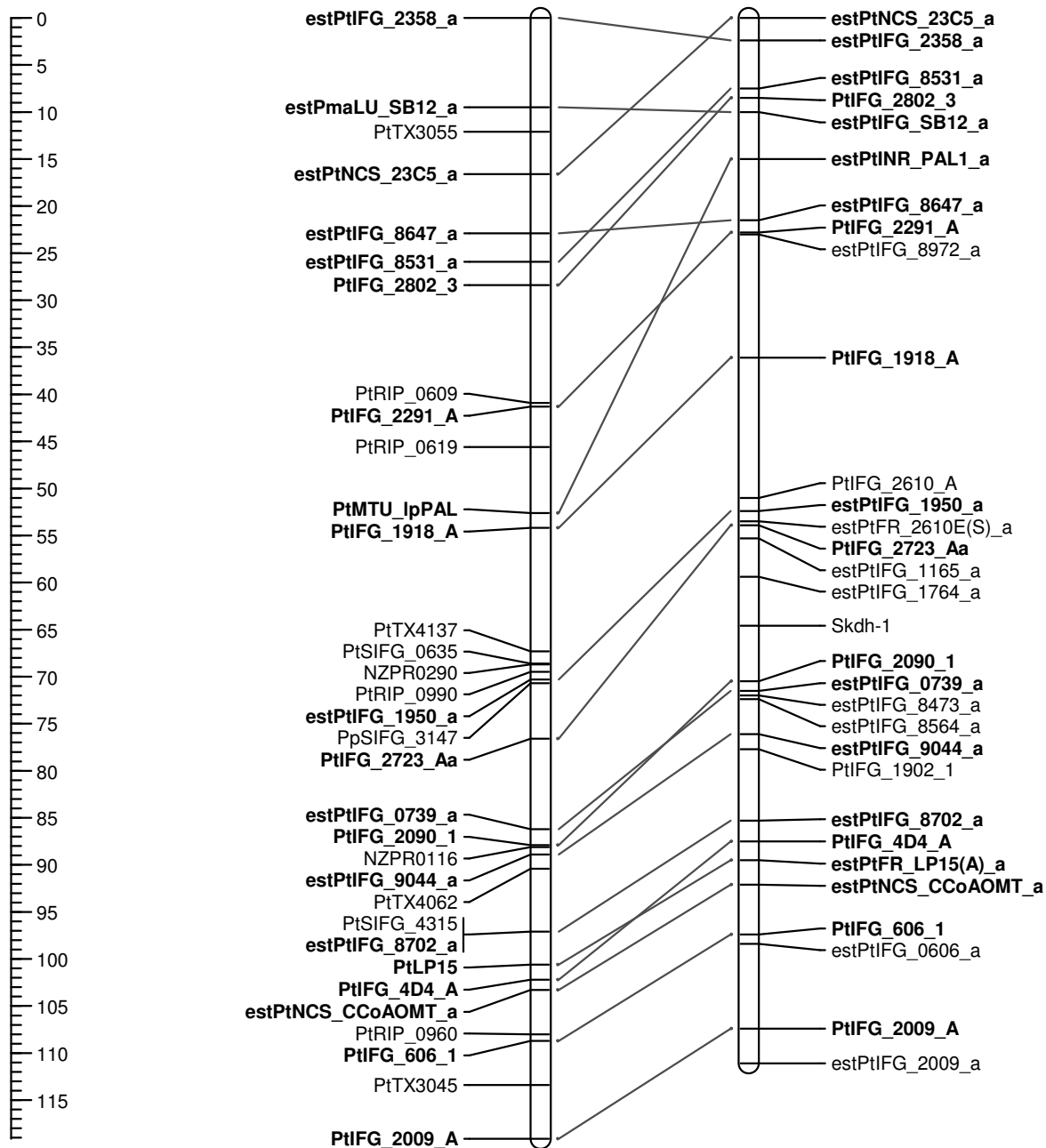

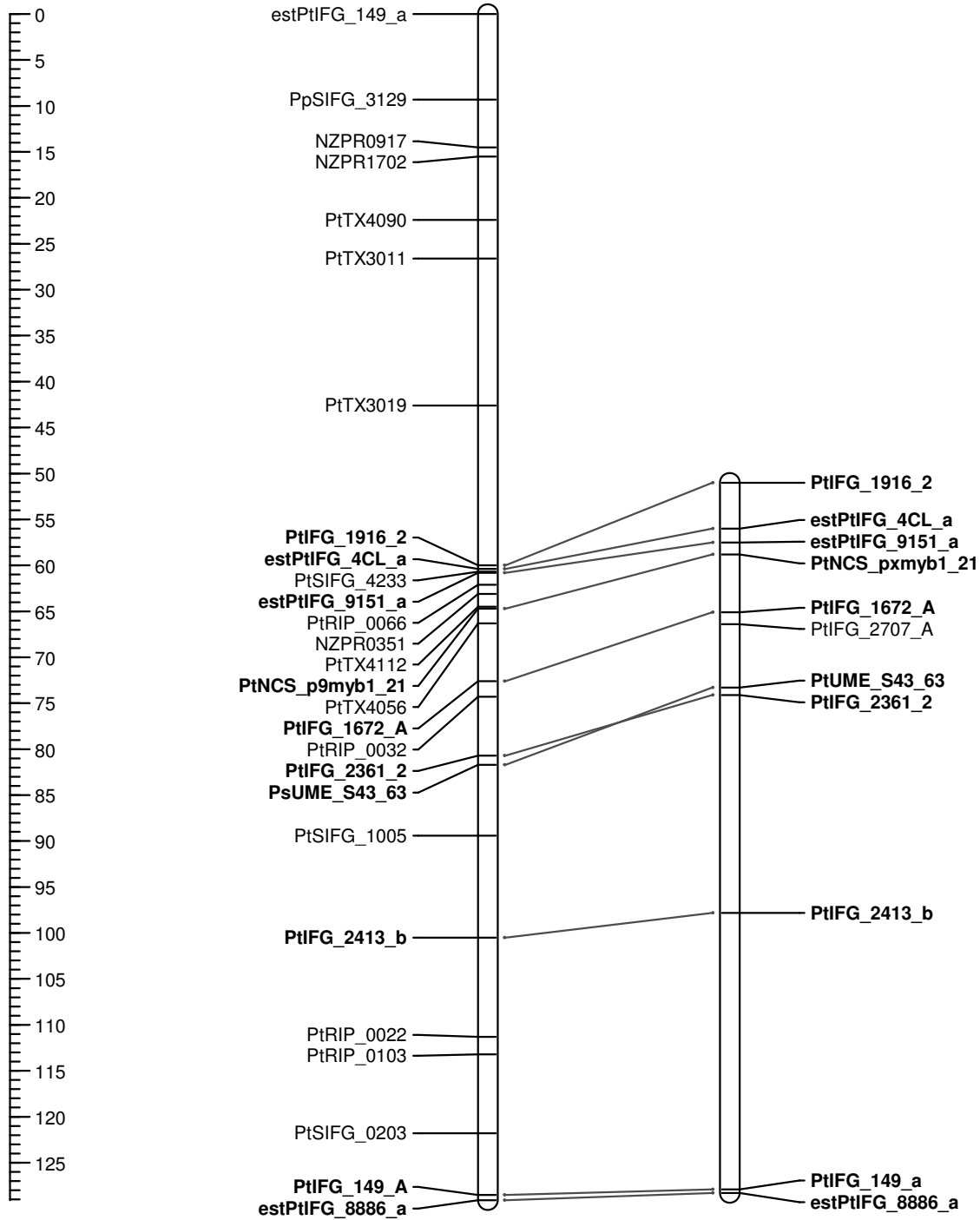

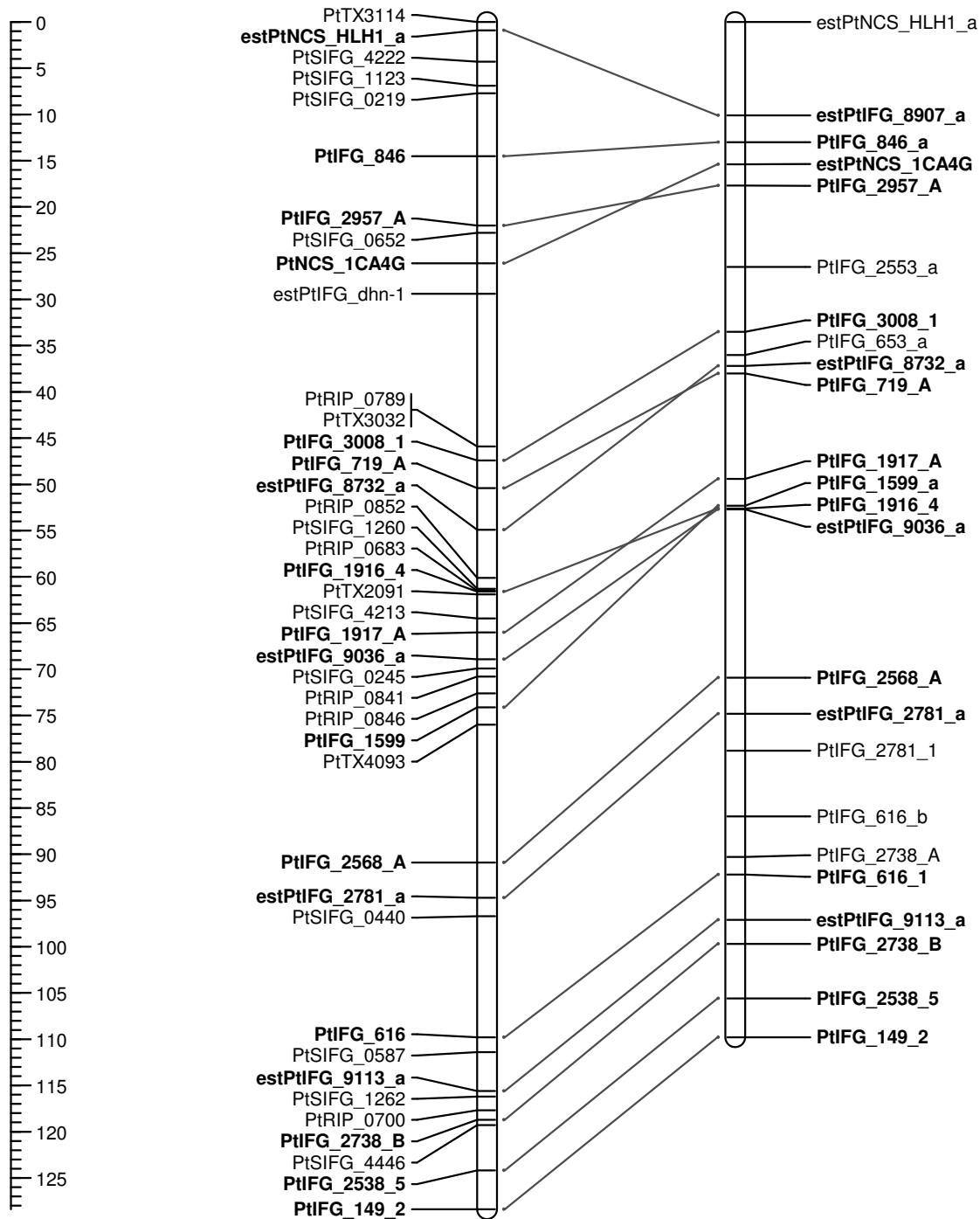

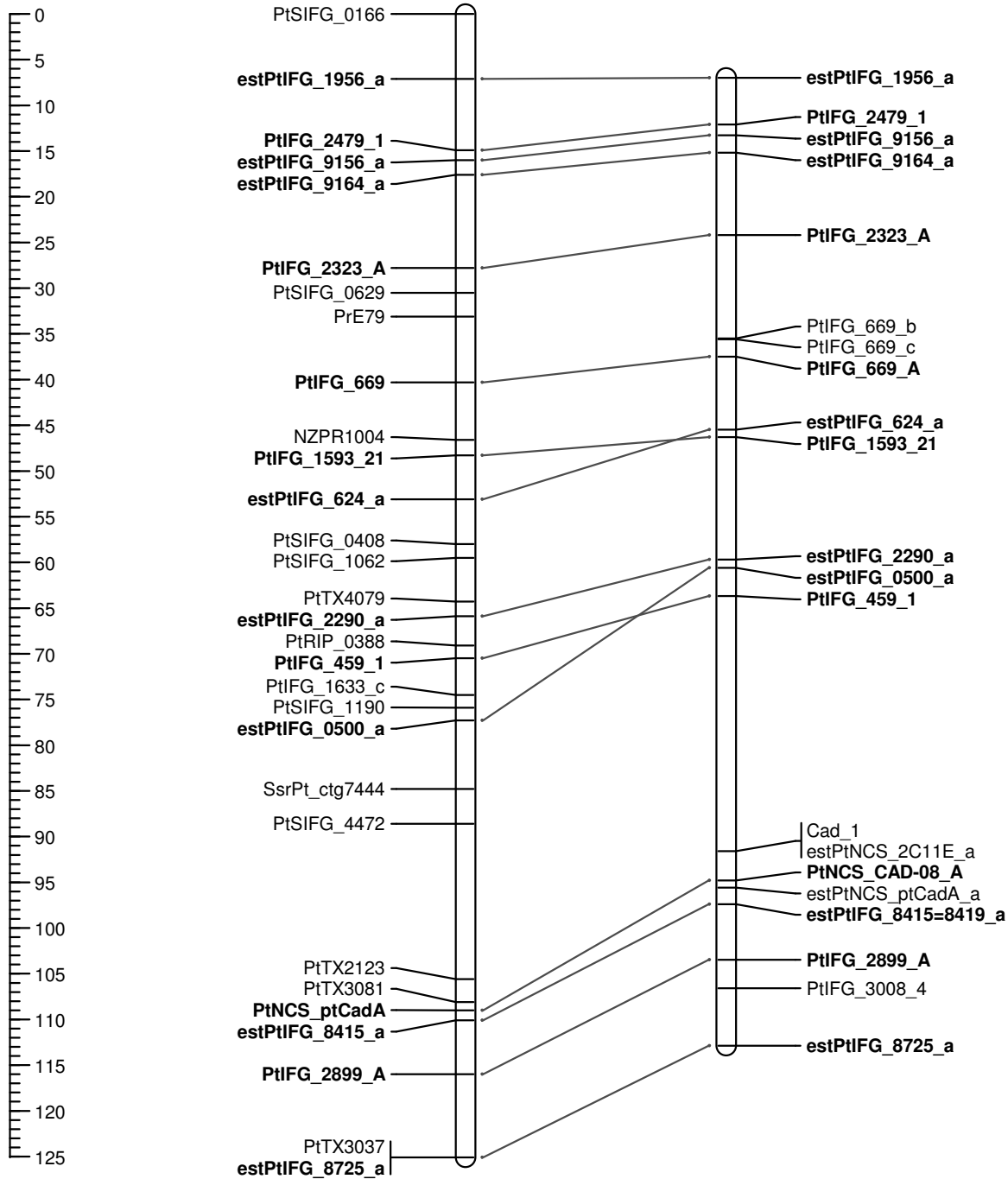

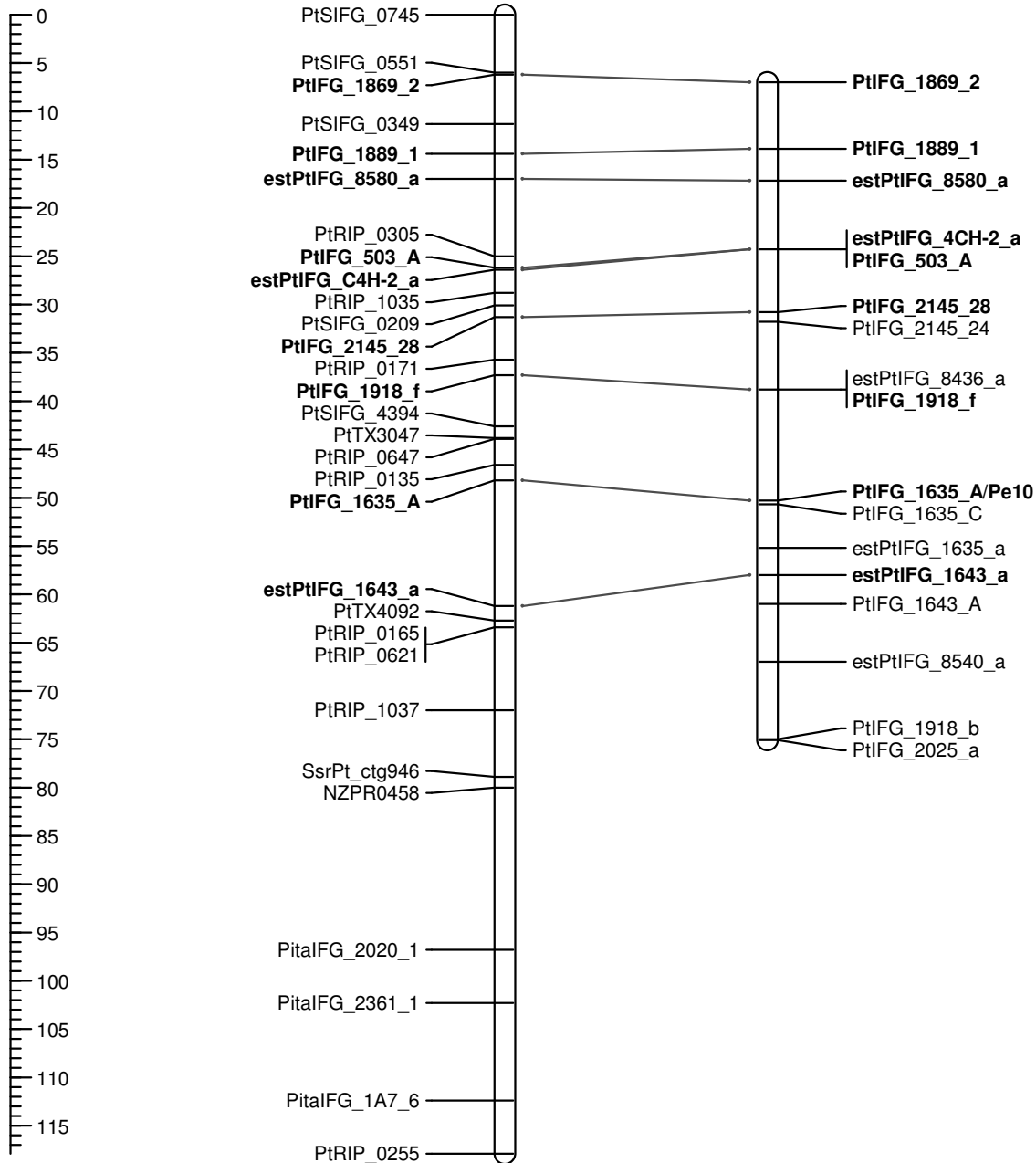

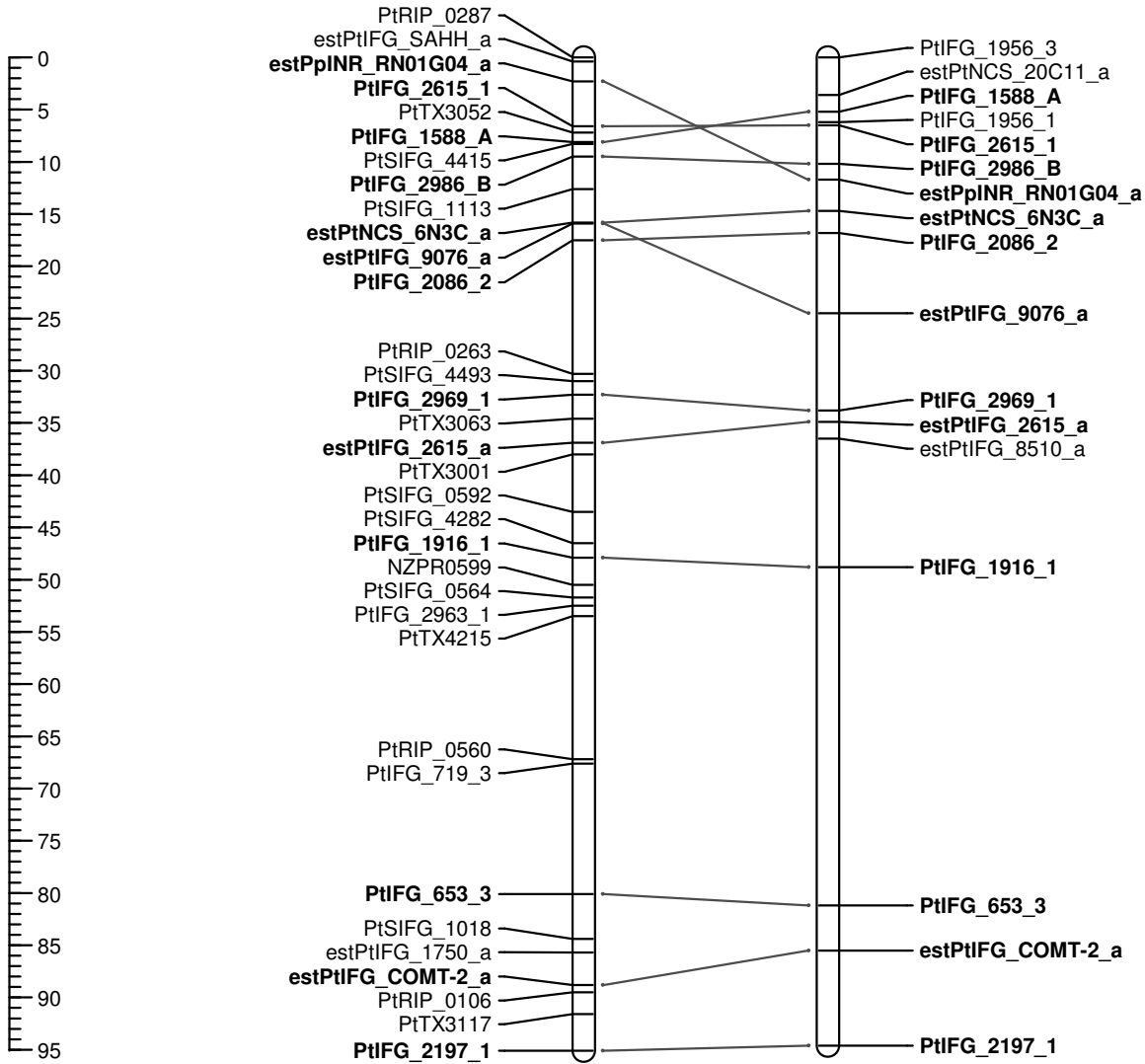

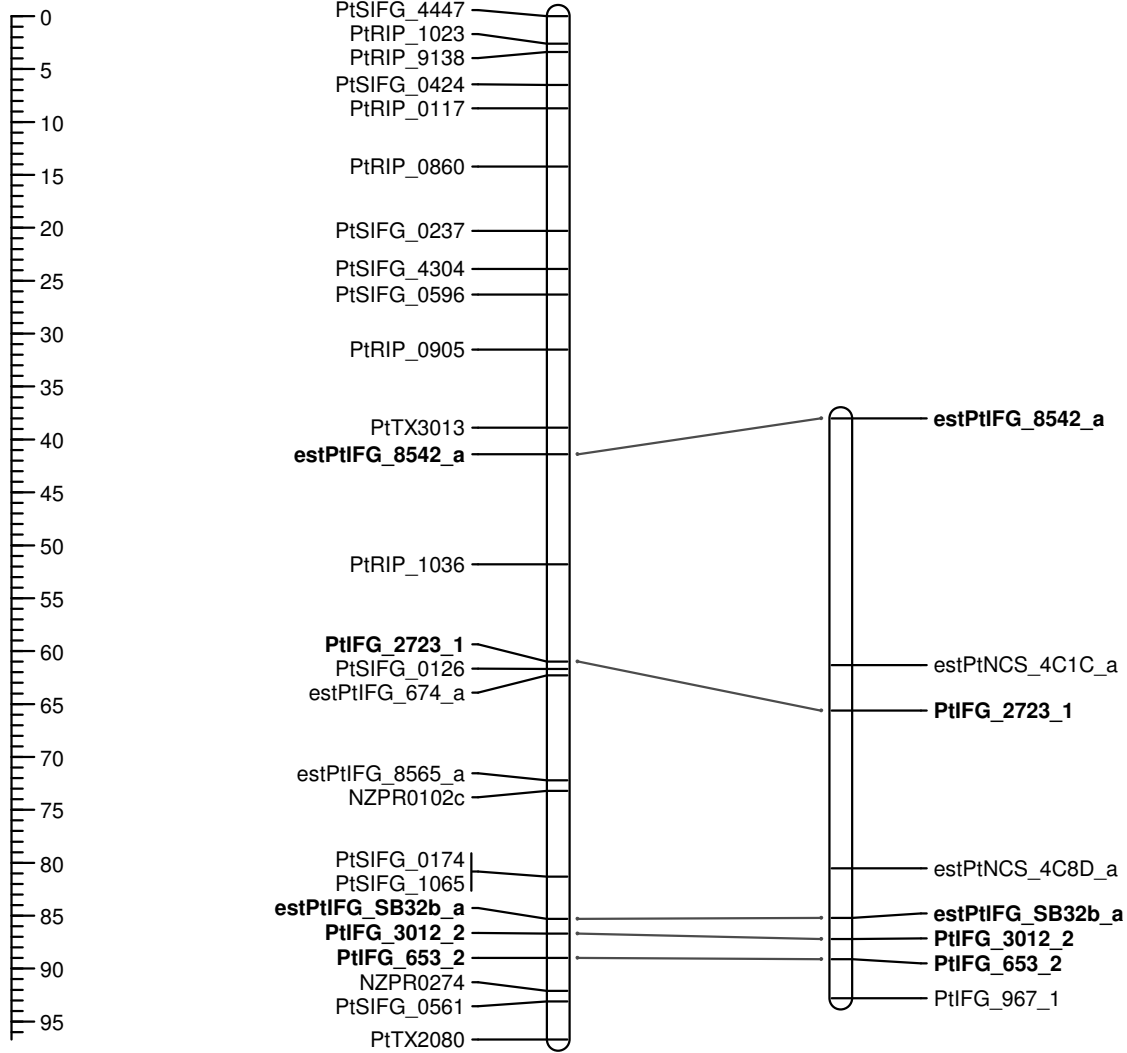

Supplement: Additional file 6 — Comparative P. taeda genetic maps: Round-2 map aligned with map of Krutovsky et al. 2004 [file 1471-2156-12-17-S6.PDF]

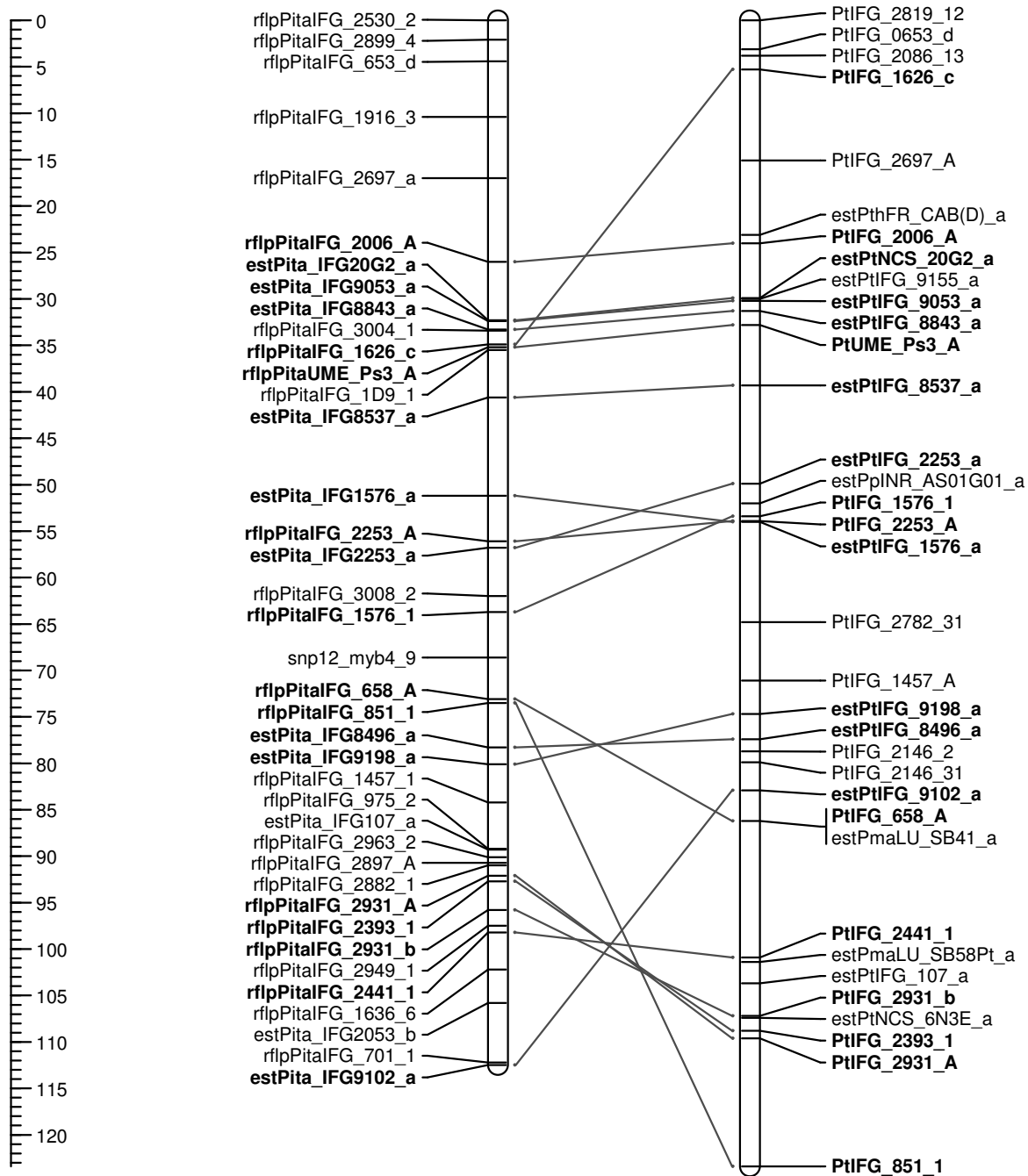

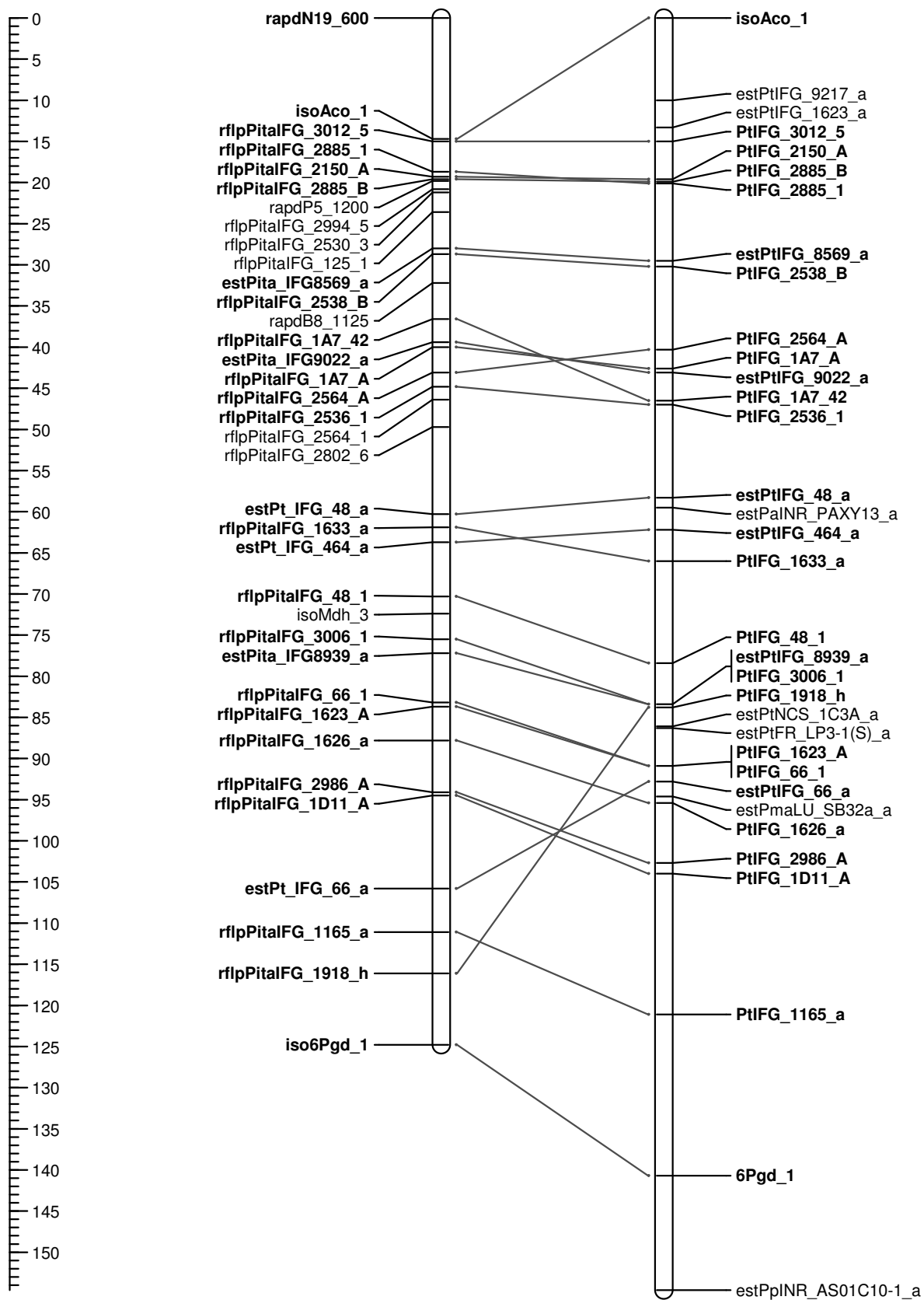

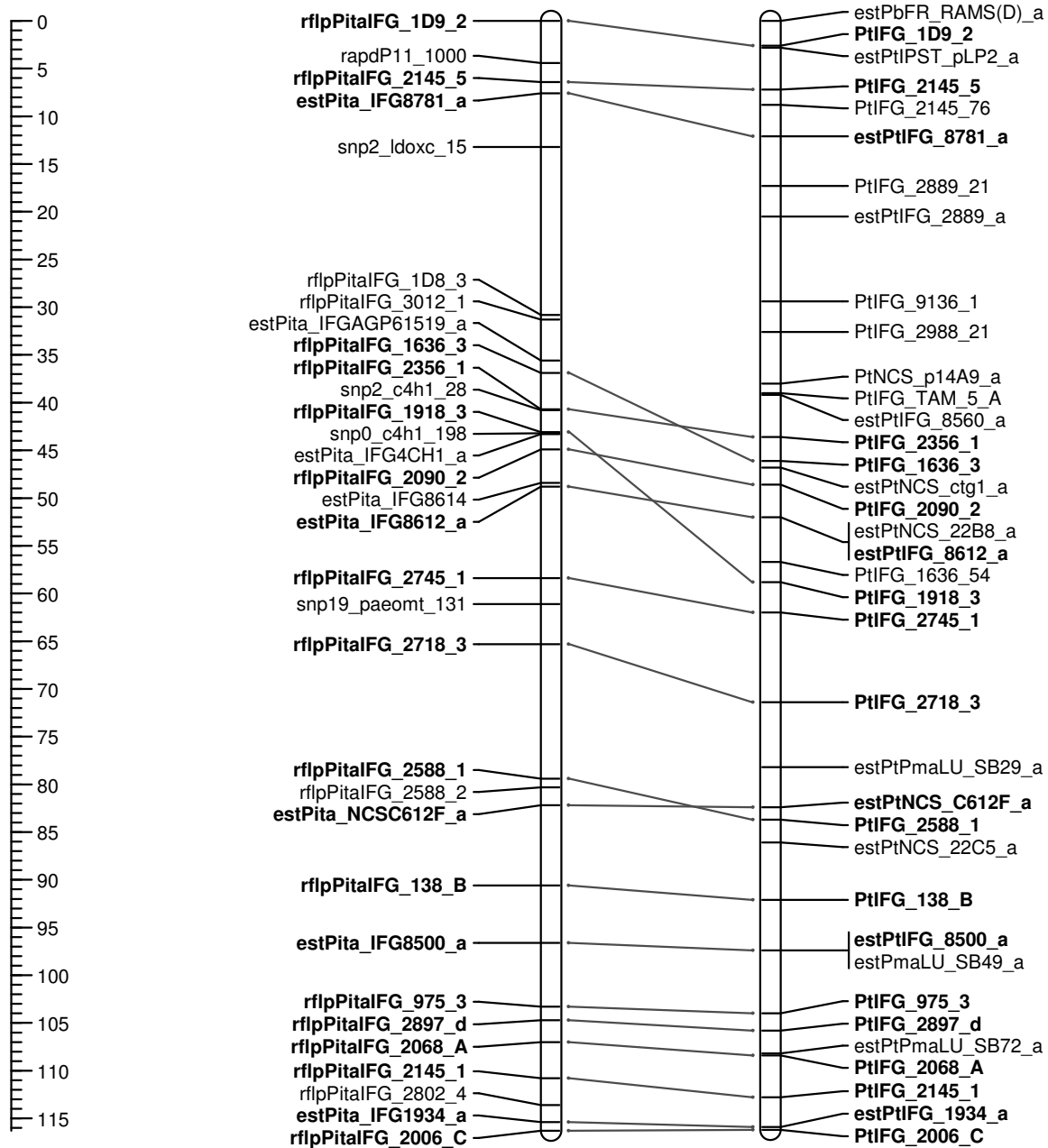

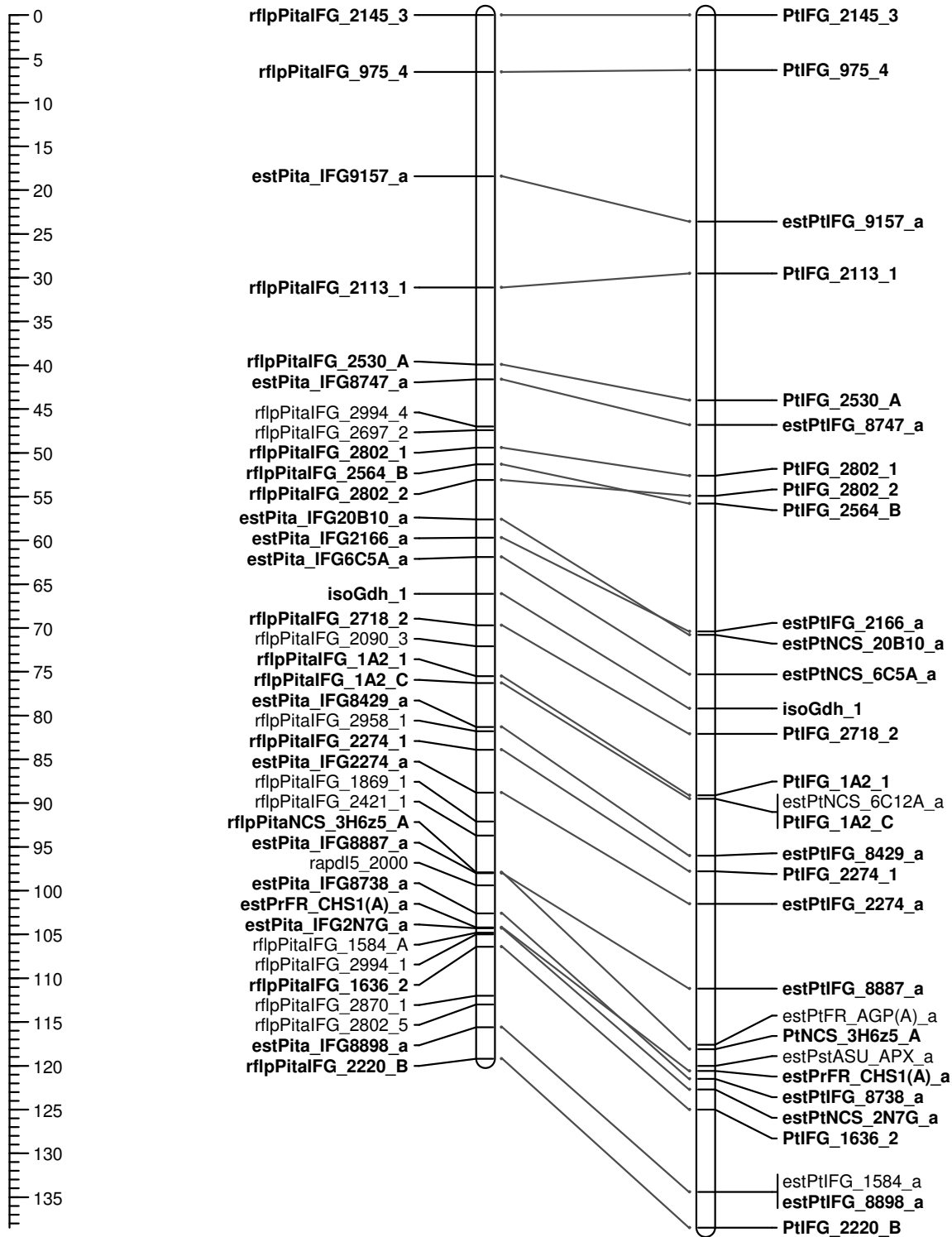

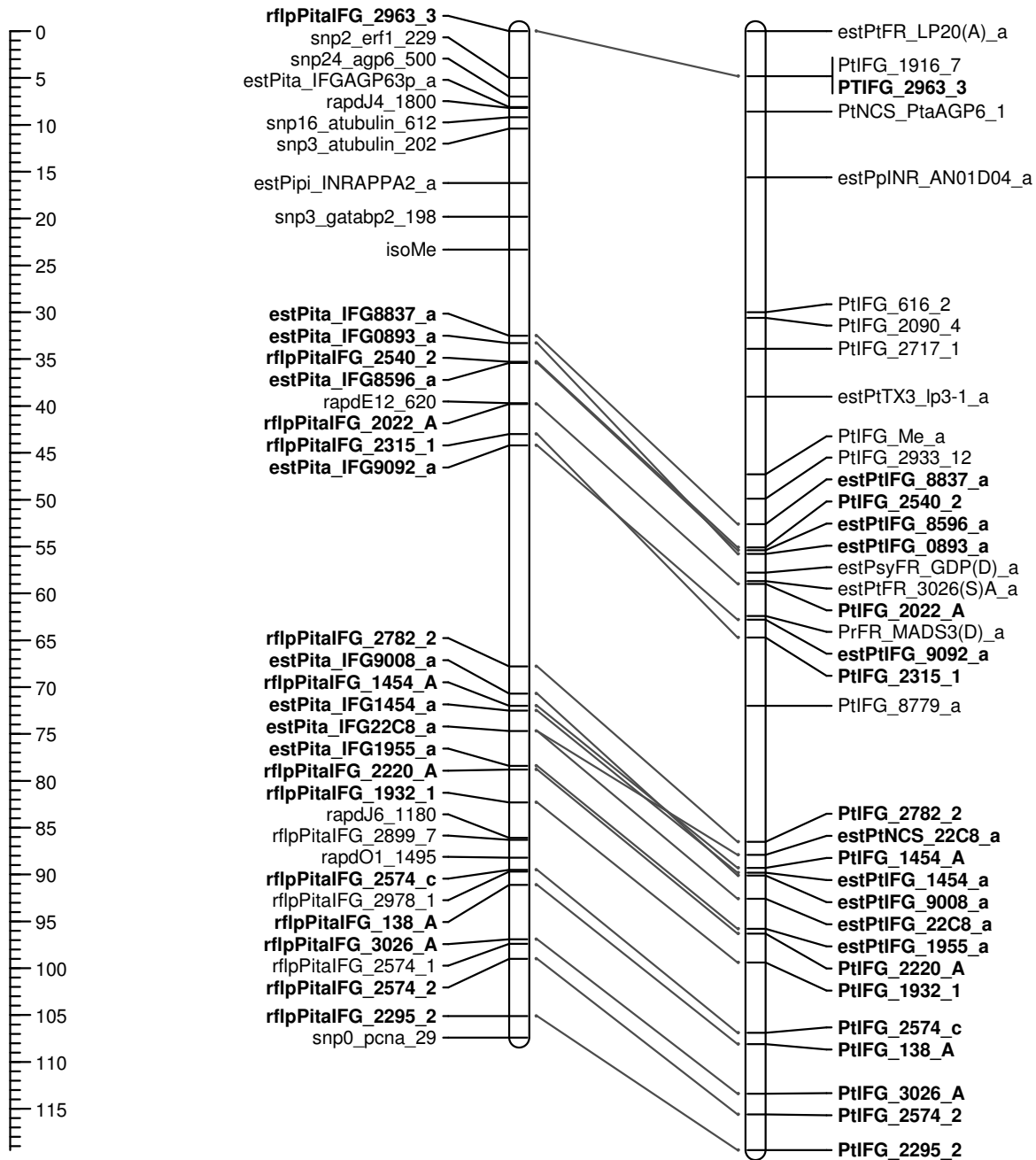

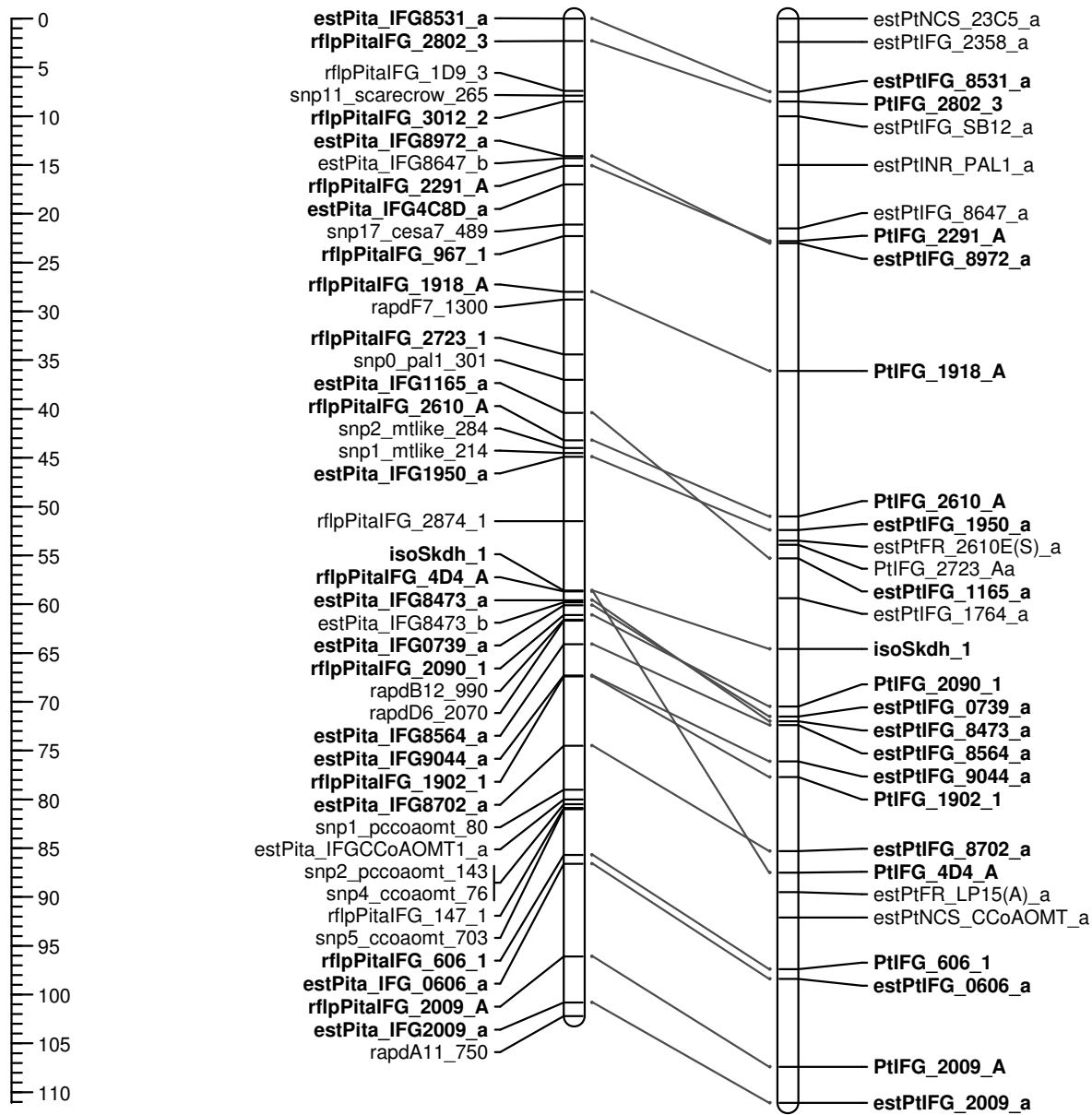

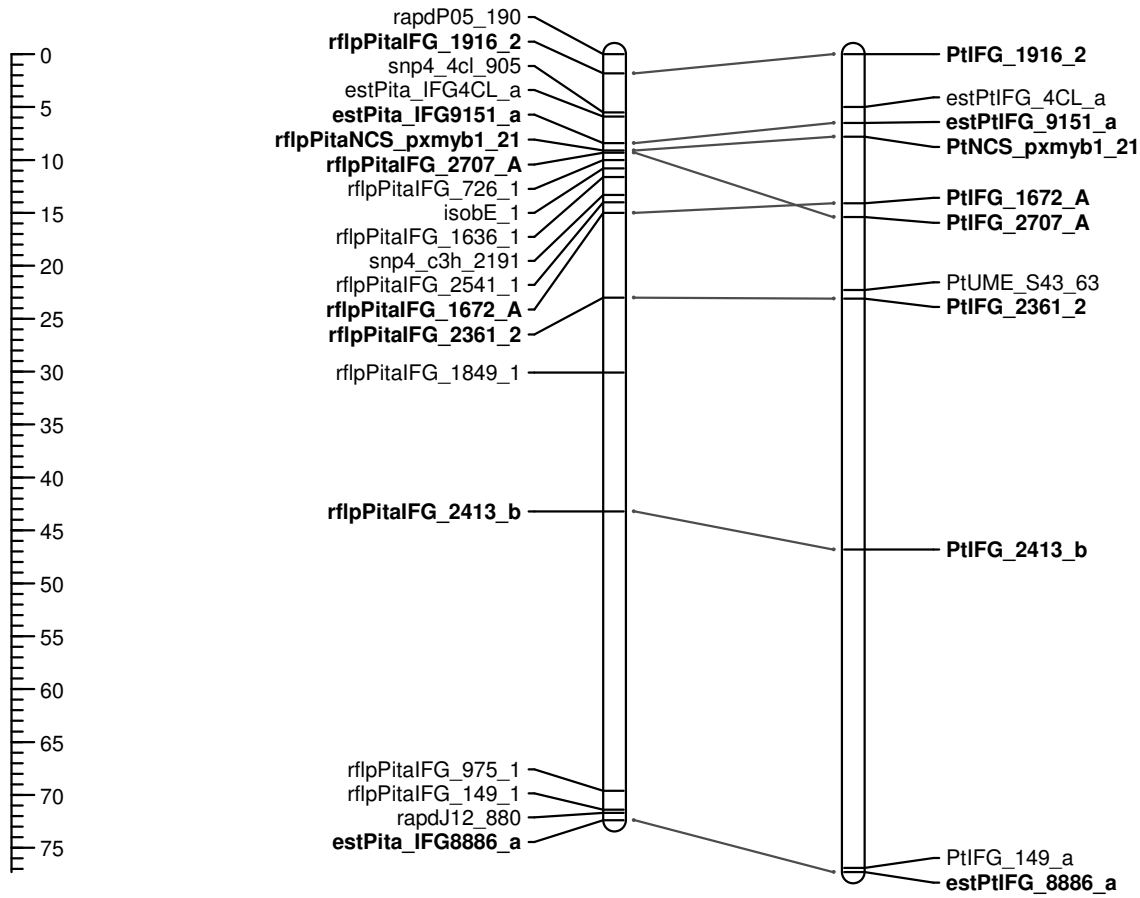

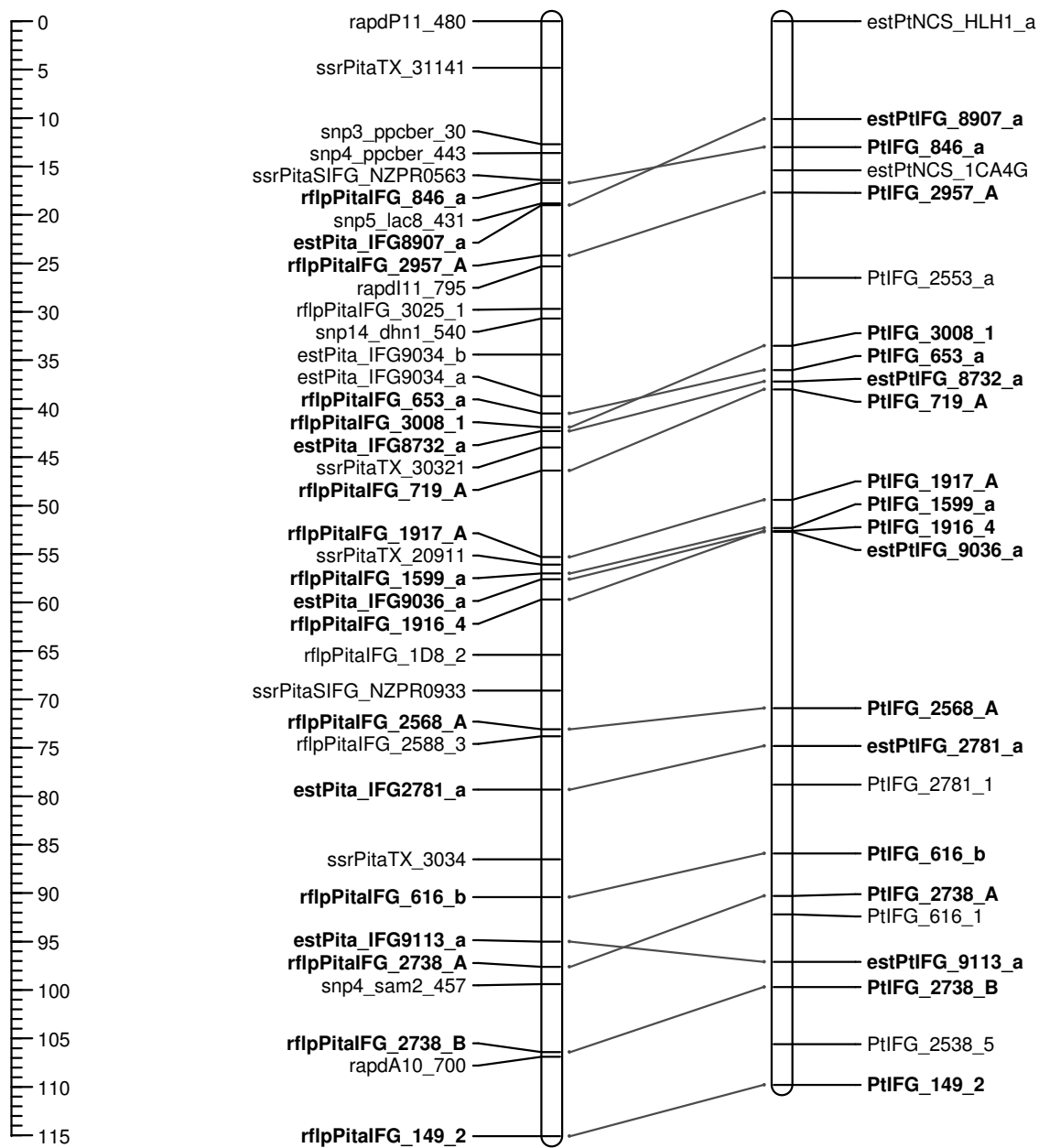

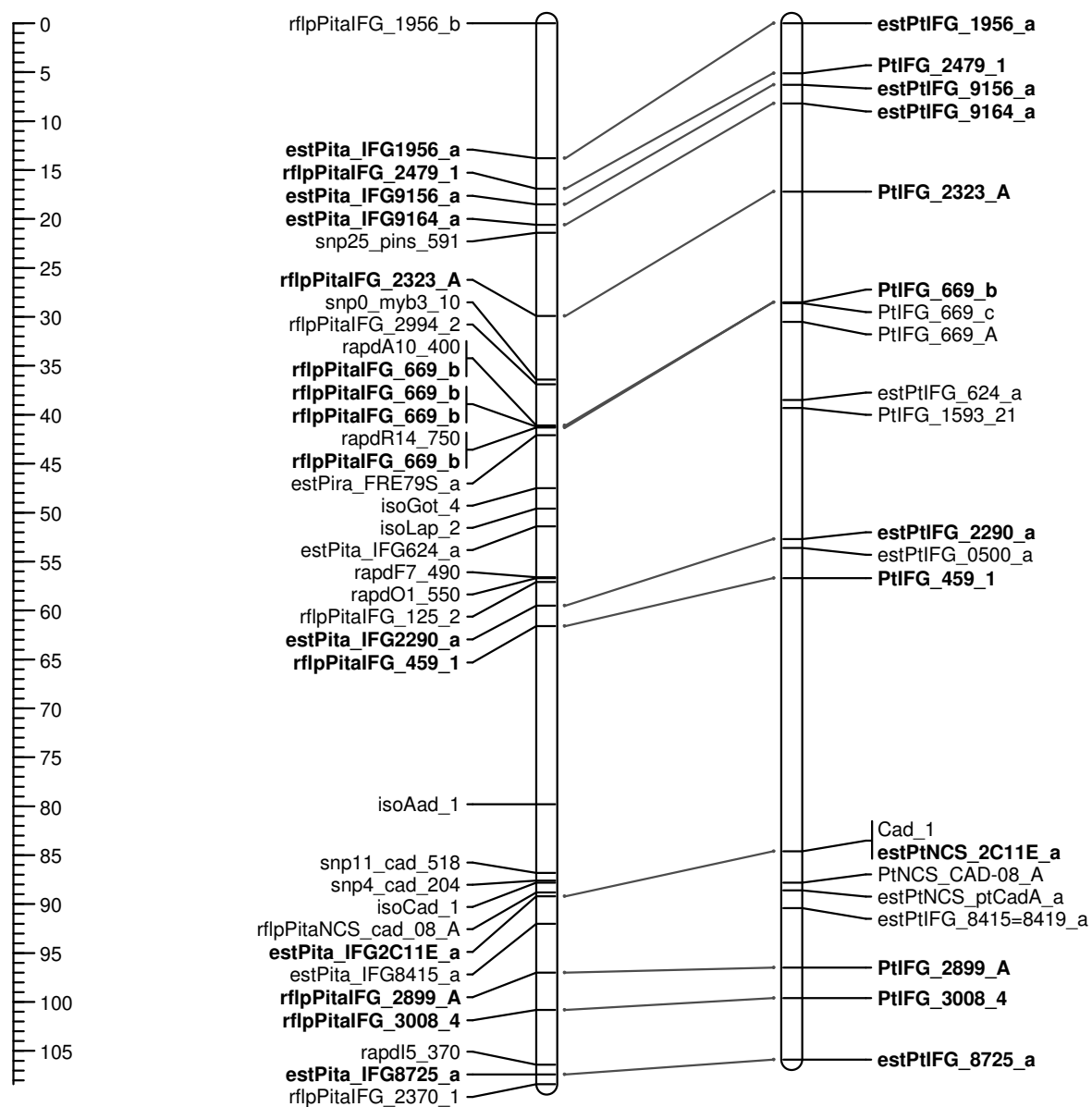

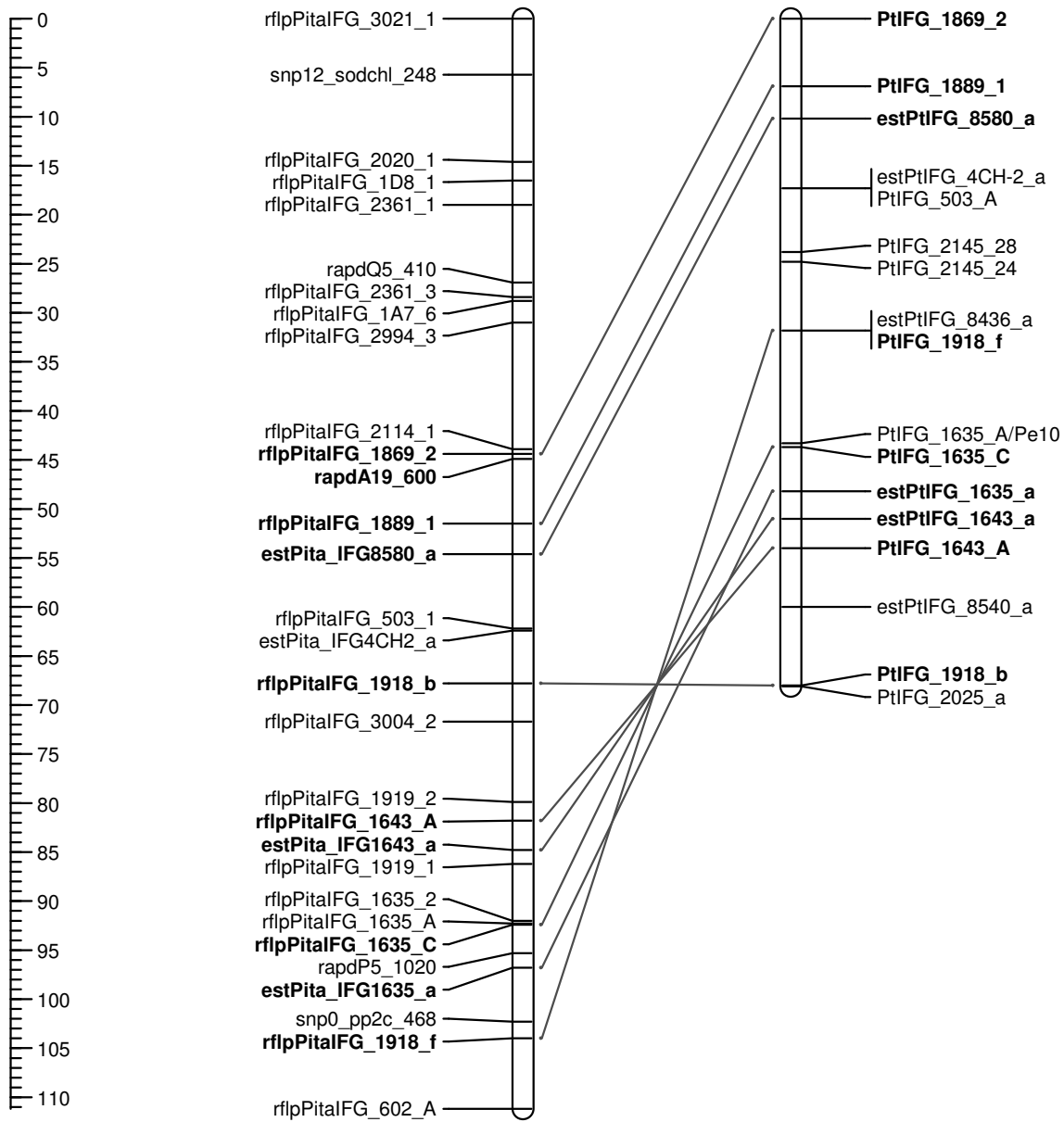

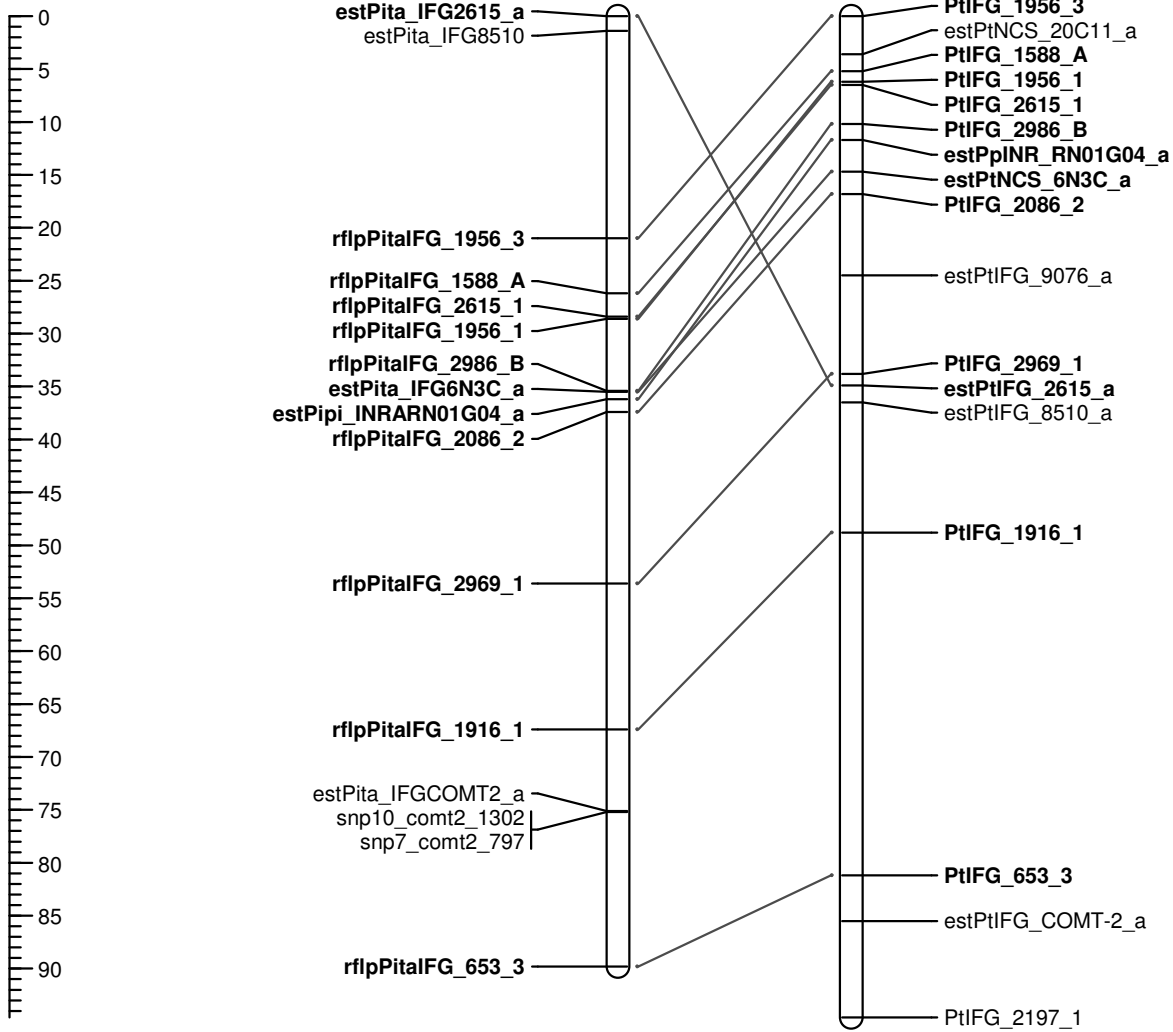

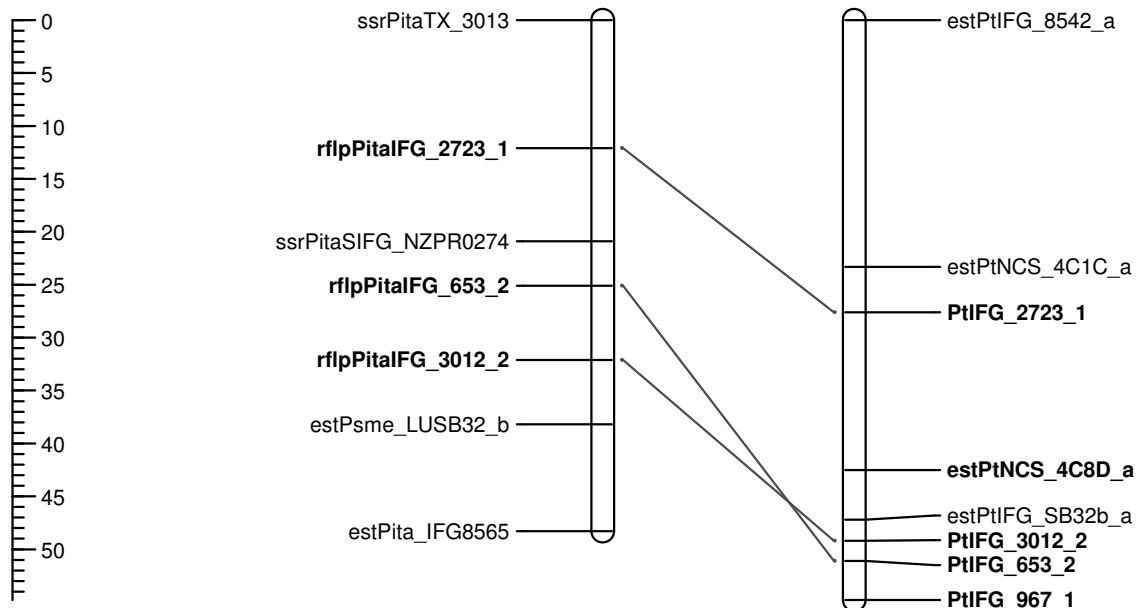

Supplement: Additional file 7 — Comparative P. taeda genetic maps: Map of Krutovsky et al. 2004 aligned with map of Eckert et al. 2009. [file 1471-2156-12-17-S7.PDF]
