# Supplementary material for: An annotated genetic map of loblolly pine based on microsatellite and cDNA markers
Source: BMC Genet. 2011 Jan 26;12:17. doi: 10.1186/1471-2156-12-17 (PMC3038140; doi:10.1186/1471-2156-12-17)
Supplement: Additional file 8 — Table of DNA sequence annotations for reported markers. Data include: marker and GenBank accession number, clone ID, species of origin, UniGene cluster ID, assigned GO term and GO lineage, assigned reference protein, marker type, P. taeda map status, and supplemental notes This HTML data table conforms to the XHTML 1.1 standard of the World Wide Web Consortium (W3C), as determined at http://validator.w3.org, and can be viewed with any web browser, as well as with Microsoft Excel or Word. [file 1471-2156-12-17-S8.HTM]

Annotations for 518 loblolly pine SSR, ESTP and RFLP marker sequences.


Additional File 8

Unless otherwise noted in the Notes column, the NCBI UniGene assignment is based on
the listed GenBank Acc and the reference protein assignment is based on the blastx
similarity of a UniGene's uniq sequence to a RefSeq protein.

All reference protein assignments are based on 45% or greater amino acid identity.
ref| = RefSeq protein database; gb| = GenBank protein database; n/a = not applicable, not available or unknown.

| Marker ID | GenBank Acc | species | clone ID | UniGene cluster ID | [GO ID] GO term | GO ontology | GO lineage level-3 | GO lineage level-4 | GO lineage level-5 | reference protein | reference protein annotation [species] | marker type | P. taeda map status | Notes |
| --- | --- | --- | --- | --- | --- | --- | --- | --- | --- | --- | --- | --- | --- | --- |
| estPaINR\_PAXY13\_a | gb|EX438659 | Picea glauca | GQ04008\_I01 | Pta.584 | [GO:0015250] water channel activity | molecular\_function | [GO:0005215] transporter activity | [GO:0022857] transmembrane transporter activity | [GO:0022891] substrate-specific transmembrane transporter activity | ref|NP\_195236 | aquaporin, plasma membrane intrinsic protein 3 [Arabidopsis thaliana] | ESTP | mapped | UniGene assignment is based on blastn similarity to the homologous Pinus taeda EST gb|CV033572; Score = 1291 bits (699), Expect = 0.0, Identities = 790/835 (94%), Gaps = 2/835 (0%). |
| estPmaLU\_SB12\_a | gb|AF051208 | Picea mariana | n/a | Pta.2976 | [GO:0003730] mRNA 3'-UTR binding | molecular\_function | [GO:0005488] binding | [GO:0003676] nucleic acid binding | [GO:0003723] RNA binding | ref|NP\_564018 | oligouridylate binding protein 1B [Arabidopsis thaliana] | ESTP | mapped | UniGene assignment is based on blastn similarity to the homologous Pinus taeda EST gb|DR070859; Score = 913 bits (494), Expect = 0.0, Identities = 634/697 (90%), Gaps = 27/697 (3%). |
| estPmaLU\_SB32\_a | gb|AF124749 | Picea glauca | n/a | n/a | [GO:0051726] regulation of cell cycle | biological\_process | [GO:0009987] cellular process | [GO:0007049] cell cycle | [GO:0051726] regulation of cell cycle | ref|NP\_001053564 | cyclin [Oryza sativa] | ESTP | mapped | RefSeq protein assignment is based on blastx similarity to the Picea glauca seq. gb|AF124749. Score = 45.4 bits (106), Expect = 0.003, Identities = 21/39 (53%), Positives = 24/39 (61%), Gaps = 0/39 (0%). |
| estPmaLU\_SB41\_a | gb|AF051231 | Picea mariana | n/a | Pta.5104 | [GO:0008308] voltage-gated anion channel activity | molecular\_function | [GO:0005215] transporter activity | [GO:0022857] transmembrane transporter activity | [GO:0022891] substrate-specific transmembrane transporter activity | ref|NP\_001049975 | porin 3, Eukaryotic porin family [Oryza sativa] | ESTP | mapped | UniGene assignment is based on blastn similarity to the homologous Pinus taeda EST gb|CO199564; Score = 928 bits (502), Expect = 0.0, Identities = 583/622 (93%), Gaps = 6/622 (0%). |
| estPmaLU\_SB49\_a | gb|AF051235 | Picea mariana | n/a | Pta.4351 | [GO:0003674] molecular\_function | molecular\_function | [Lineage ends at prior level] | [Lineage ends at prior level] | [Lineage ends at prior level] | ref|NP\_564061 | unknown protein [Arabidopsis thaliana] | ESTP | mapped | UniGene assignment is based on blastn similarity to the homologous Pinus taeda EST gb|DR385657; Score = 1079 bits (584), Expect = 0.0, Identities = 732/804 (91%), Gaps = 8/804 (0%). |
| estPmaLU\_SB58\_a | gb|AF051242 | Picea mariana | n/a | Pta.317 | [GO:0003735] structural constituent of ribosome | molecular\_function | [GO:0005198] structural molecule activity | [GO:0003735] structural constituent of ribosome | [Lineage ends at prior level] | ref|NP\_001045083 | 60S ribosomal protein L5 [Oryza sativa] | ESTP | mapped | UniGene assignment is based on blastn similarity to the homologous Pinus taeda EST gb|DR013771; Score = 658 bits (356), Expect = 0.0, Identities = 477/534 (89%), Gaps = 13/534 (2%). |
| estPpINR\_AS01C10-1\_a | gb|AL749809 | Pinus pinaster | AS01C10 | Pta.18353 | [GO:0042411] beta-carotene hydroxylase activity | molecular\_function | [GO:0003824] catalytic activity | [GO:0016491] oxidoreductase activity | [GO:0004497] monooxygenase activity | ref|NP\_200070 | beta-carotene hydroxylase 2 [Arabidopsis thaliana] | ESTP | mapped | UniGene assignment is based on blastn similarity to the homologous Pinus taeda EST gb|DR021701; Score = 822 bits (445), Expect = 0.0, Identities = 536/574 (93%), Gaps = 30/574 (5%). |
| estPpINR\_RN01G04\_a | gb|AL750371 | Pinus pinaster | RN01G04 | Pta.905 | [GO:0004674] protein serine/threonine kinase activity | molecular\_function | [GO:0003824] catalytic activity | [GO:0016740] transferase activity | [GO:0016772] transferase activity, transferring phosphorus-containing groups | ref|NP\_001058752 | casein kinase II alpha subunit [Oryza sativa] | ESTP | mapped | UniGene assignment is based on blastn similarity to the homologous Pinus taeda EST gb|DT627165; Score = 682 bits (369), Expect = 0.0, Identities = 377/381 (98%), Gaps = 0/381 (0%). |
| estPtIFG\_107\_a | gb|H75114 | Pinus taeda | 0107J | Pta.576 | [GO:0015078] hydrogen ion transmembrane transporter activity | molecular\_function | [GO:0005215] transporter activity | [GO:0022892] substrate-specific transporter activity | [GO:0022891] substrate-specific transmembrane transporter activity | ref|NP\_194953 | ATP synthase family [Arabidoposis thaliana] | ESTP | mapped |  |
| estPtIFG\_149\_a | gb|H75240 | Pinus taeda | 0149e | Pta.1913 | [GO:0004034] aldose 1-epimerase activity | molecular\_function | [GO:0003824] catalytic activity | [GO:0016853] isomerase activity | [GO:0016854] racemase and epimerase activity | ref|NP\_200543 | aldose 1-epimerase family protein [Arabidopsis thaliana] | ESTP | mapped |  |
| estPtIFG\_464\_a | gb|H75150 | Pinus taeda | 0464e | Pta.584 | [GO:0015250] water channel activity | molecular\_function | [GO:0005215] transporter activity | [GO:0022857] transmembrane transporter activity | [GO:0022891] substrate-specific transmembrane transporter activity | ref|NP\_195236 | aquaporin, plasma membrane intrinsic protein 3 [Arabidopsis thaliana] | ESTP | mapped |  |
| estPtIFG\_500\_a | gb|H75151 | Pinus taeda | 0500e | Pta.11539 | [GO:0005507] copper ion binding | molecular\_function | [GO:0005488] binding | [GO:0043167] ion binding | [GO:0043169] cation binding | ref|NP\_173459 | DNA-damage-repair/toleration protein 112, plastocyanin, chloroplast precursor [Arabidopsis thaliana] | ESTP | mapped |  |
| estPtIFG\_624\_a | gb|H75105 | Pinus taeda | 0624M | Pta.11566 | [GO:0016301] kinase activity | molecular\_function | [GO:0003824] catalytic activity | [GO:0016740] transferase activity | [GO:0016772] transferase activity, transferring phosphorus-containing groups | ref|NP\_567945 | calcium dependent protein serine/threonine kinase [Arabidopsis thaliana] | ESTP | mapped |  |
| estPtIFG\_674\_a | gb|CO160324 | Pinus taeda | FLD1\_20\_F03\_A029 | Pta.11669 | [GO:0003755] peptidyl-prolyl cis-trans isomerase activity | molecular\_function | [GO:0003824] catalytic activity | [GO:0016853] isomerase activity | [GO:0016859] cis-trans isomerase activity | ref|NP\_180557 | peptidyl-prolyl cis-trans isomerase, cyclophilin [Arbidopsis thaliana] | ESTP | mapped | Sequence of Pinus taeda clone 0674 is not available. STS primers match Pinus taeda EST gb|CO160324. |
| estPtIFG\_739\_a | gb|DR743097 | Pinus taeda | RTCU1\_13\_C02\_A029 | Pta.2876 | [GO:0004630] phospholipase D activity | molecular\_function | [GO:0003824] catalytic activity | [GO:0016787] hydrolase activity | [GO:0016788] hydrolase activity, acting on ester bonds | ref|NP\_188194 | phospholipase D alpha 1 [Arabidopsis thaliana] | ESTP | mapped | Sequence of Pinus taeda clone 0739 is not available. STS primers match Pinus taeda EST gb|DR743097. |
| estPtIFG\_893\_a | gb|CO361162 | Pinus taeda | NDL2\_3\_D09\_A029 | Pta.72 | [GO:0008289] lipid binding | molecular\_function | [GO:0005488] binding | [GO:0008289] lipid binding | [Lineage ends at prior level] | gb|AAA82182 | nonspecific lipid-transfer protein [Pinus taeda] | ESTP | mapped | Sequence of Pinus taeda clone 0893 is not available. STS primers match Pinus taeda EST gb|CO361162. UniGene assignment is based on gb|CO361162. |
| estPtIFG\_1454\_a | gb|H75120 | Pinus taeda | 1454J | n/a | [GO:0003674] molecular\_function | molecular\_function | [Lineage ends at prior level] | [Lineage ends at prior level] | [Lineage ends at prior level] | n/a | n/a | ESTP | mapped |  |
| estPtIFG\_1576\_a | gb|DR681980 | Pinus taeda | PWAA474 | Pta.22195 | [GO:0003674] molecular\_function | molecular\_function | [Lineage ends at prior level] | [Lineage ends at prior level] | [Lineage ends at prior level] | n/a | n/a | ESTP | mapped | Sequence of Pinus taeda clone 1576 is not available. STS primers match Pinus taeda EST gb|DR681980. UniGene assignment is based on gb|DR681980. |
| estPtIFG\_1643\_a | gb|H75191 | Pinus taeda | 1643e | n/a | [GO:0003674] molecular\_function | molecular\_function | [Lineage ends at prior level] | [Lineage ends at prior level] | [Lineage ends at prior level] | n/a | n/a | ESTP | mapped |  |
| estPtIFG\_1750\_a | gb|H75022 | Pinus taeda | 1750s | Pta.1650 | [GO:0009055] 2 iron, 2 sulfur cluster binding | molecular\_function | [GO:0005488] binding | [GO:0051540] metal cluster binding | [GO:0051536] iron-sulfer cluster binding | ref|NP\_172565 | ferredoxin [Arabidopsis thaliana] | ESTP | mapped |  |
| estPtIFG\_1934\_a | gb|H75112 | Pinus taeda | 1934M | Pta.575 | [GO:0016168] chlorophyll binding | molecular\_function | [GO:0005488] binding | [GO:0046906] tetrapyrrole binding | [GO:0016168] chlorophyll binding | ref|NP\_565786 | chlorophyll a/b binding protein, photosystem II [Arabidopsis thaliana] | ESTP | mapped |  |
| estPtIFG\_1950\_a | gb|H75126 | Pinus taeda | 1950J | Pta.598 | [GO:0008943] glyceraldehyde-3-phosphate dehydrogenase activity | molecular\_function | [GO:0003824] catalytic activity | [GO:0016491] oxidoreductase activity | [GO:0016903] oxidoreductase activity, acting on the aldehyde or oxo group of donors | ref|NP\_172750 | glyceraldehyde-3-phosphate dehydrogenase [Arabidopsis thaliana] | ESTP | mapped |  |
| estPtIFG\_1955\_a | gb|H75038 | Pinus taeda | 1955e | Pta.9950 | [GO:0003674] molecular\_function | molecular\_function | [Lineage ends at prior level] | [Lineage ends at prior level] | [Lineage ends at prior level] | ref|NP\_001045308 | protein kinase C conserved region 2, C2, domain containing protein [Oryza sativa] | ESTP | mapped | UniGene assignment is based on blastn similarity to the homologous Pinus taeda EST gb|DR018615; Score = 281 bits (152), Expect = 6e-75, Identities = 208/233 (89%), Gaps = 12/233 (5%). |
| estPtIFG\_1956\_a | gb|H75039 | Pinus taeda | 1956s | Pta.5686 | [GO:0004842] ubiquitin-protein ligase activity | molecular\_function | [GO:0003824] catalytic activity | [GO:0016874] ligase activity | [GO:0016879] ligase activity, forming carbon-nitrogen bonds | ref|NP\_177189 | ubiquitin-protein ligase [Arabidopsis thaliana] | ESTP | mapped |  |
| estPtIFG\_2166\_a | gb|H75060 | Pinus taeda | 2166s | Pta.515 | [GO:0004739] pyruvate dehydrogenase (acetyl-transferring) activity | molecular\_function | [GO:0003824] catalytic activity | [GO:0016491] oxidoreductase activity | [GO:0016903] oxidoreductase activity, acting on the aldehyde or oxo group of donors | ref|NP\_001048068 | pyruvate dehydrogenase E1 component subunit alpha-2, mitochondrial precursor [Oryza sativa] | ESTP | mapped |  |
| estPtIFG\_2290\_a | gb|H75067 | Pinus taeda | 2290e | Pta.1240 | [GO:0016168] chlorophyll binding | molecular\_function | [GO:0005488] binding | [GO:0046906] tetrapyrrole binding | [GO:0016168] chlorophyll binding | ref|NP\_192772 | chlorophyll a/b binding protein, photosystem II LHCP type II [Arabidopsis thaliana] | ESTP | mapped |  |
| estPtIFG\_2358\_a | gb|H75088 | Pinus taeda | 2358e | Pta.11555 | [GO:0003674] molecular\_function | molecular\_function | [Lineage ends at prior level] | [Lineage ends at prior level] | [Lineage ends at prior level] | n/a | n/a | ESTP | mapped |  |
| estPtIFG\_2615\_a | gb|H75223 | Pinus taeda | 2615s | Pta.18383 | [GO:0003674] molecular\_function | molecular\_function | [Lineage ends at prior level] | [Lineage ends at prior level] | [Lineage ends at prior level] | ref|NP\_001060847 | unknown protein [Oryza sativa] | ESTP | mapped | UniGene assignment is based on blastn similarity to the homologous Pinus taeda EST gb|CO170994, clone NDL1\_18\_G08\_A029; Score = 839 bits (454), Expect = 0.0, Identities = 466/471 (98%), Gaps = 4/471 (0%). |
| estPtIFG\_2781\_a | gb|H75231 | Pinus taeda | 2781e | Pta.11561 | [GO:0003674] molecular\_function | molecular\_function | [Lineage ends at prior level] | [Lineage ends at prior level] | [Lineage ends at prior level] | n/a | n/a | ESTP | mapped |  |
| estPtIFG\_2889\_a | gb|H75234 | Pinus taeda | 2889e | Pta.2699 | [GO:0006950] response to stress | biological\_process | [GO:0050896] response to stimulus | [GO:0006950] response to stress | [Lineage ends at prior level] | ref|NP\_001147703.1 | abscisic stress ripening protein 2 [Zea mays] | ESTP | mapped |  |
| estPtIFG\_4CL\_a | gb|U12012 | Pinus taeda | PT4CL1 | Pta.423 | [GO:0016207] 4-coumarate-CoA ligase activity | molecular\_function | [GO:0003824] catalytic activity | [GO:0016874] ligase activity | [GO:0016877] ligase activity, forming carbon-sulfur bonds | ref|NP\_188761 | 4-coumarate-CoA ligase [Arabidopsis thaliana] | ESTP | mapped |  |
| estPtIFG\_8415\_a | gb|AA739501 | Pinus taeda | 8419M | Pta.143 | [GO:0003674] molecular\_function | molecular\_function | [Lineage ends at prior level] | [Lineage ends at prior level] | [Lineage ends at prior level] | n/a | n/a | ESTP | mapped |  |
| estPtIFG\_8429\_a | gb|AA739505 | Pinus taeda | 8429M | Pta.11727 | [GO:0016779] nucleotidyltransferase activity | molecular\_function | [GO:0003824] catalytic activity | [GO:0016740] transferase activity | [GO:0016772] transferase activity, transferring phosphorus-containing groups | ref|NP\_175708 | cytidylyltransferase family, mitochondrial [Arabidopsis thaliana] | ESTP | mapped |  |
| estPtIFG\_8471\_a | gb|AA739525 | Pinus taeda | 8471M | Pta.11734 | [GO:0003674] molecular\_function | molecular\_function | [Lineage ends at prior level] | [Lineage ends at prior level] | [Lineage ends at prior level] | n/a | n/a | ESTP | mapped |  |
| estPtIFG\_8473\_a | gb|AA739526 | Pinus taeda | 8473M | Pta.204 | [GO:0003674] molecular\_function | molecular\_function | [Lineage ends at prior level] | [Lineage ends at prior level] | [Lineage ends at prior level] | ref|NP\_188286 | translationally controlled tumor protein [Arabidopsis thaliana] | ESTP | mapped |  |
| estPtIFG\_8500\_a | gb|AA739539 | Pinus taeda | 8500M | Pta.11738 | [GO:0003674] molecular\_function | molecular\_function | [Lineage ends at prior level] | [Lineage ends at prior level] | [Lineage ends at prior level] | n/a | n/a | ESTP | mapped |  |
| estPtIFG\_8531\_a | gb|AA739558 | Pinus taeda | 8531M | Pta.627 | [GO:0003743] translation initiation factor activity | molecular\_function | [GO:0005488] binding | [GO:0003676] nucleic acid binding | [GO:0008135] translation factor activity, nucleic acid binding | ref|NP\_177907 | eukaryotic translation initiation factor 5, putative [Arabidopsis thaliana] | ESTP | mapped |  |
| estPtIFG\_8542\_a | gb|AA739566 | Pinus taeda | 8542M | Pta.11743 | [GO:0003674] molecular\_function | molecular\_function | [Lineage ends at prior level] | [Lineage ends at prior level] | [Lineage ends at prior level] | ref|NP\_193557 | unknown protein [Arabidopsis thaliana] | ESTP | mapped |  |
| estPtIFG\_8564\_a | gb|AA739580 | Pinus taeda | 8564M | Pta.630 | [GO:0003735] structural constituent of ribosome | molecular\_function | [GO:0005198] structural molecule activity | [GO:0003735] structural constituent of ribosome | [Lineage ends at prior level] | ref|NP\_001067062 | 60S ribosomal protein L2, fragment [Oryza sativa] | ESTP | mapped |  |
| estPtIFG\_8565\_a | gb|AA739581 | Pinus taeda | 8565M | Pta.5681 | [GO:0005524] ATP binding | molecular\_function | [GO:0005488] binding | [GO:0000166] nucleotide binding | [GO:0017076] purine nucleotide binding | ref|NP\_198206.1 | ATP binding (BIP1) [Arabidopsis thaliana] | ESTP | mapped |  |
| estPtIFG\_8569\_a | gb|AA739585 | Pinus taeda | 8569M | n/a | [GO:0003924] GTPase activity | molecular\_function | [GO:0003824] catalytic activity | [GO:0016787] hydrolase activity | [GO:0016817] hydrolase activity, acting on acid anhydrides | ref|XP\_002324047 | tubulin alpha-7 chain [Populus trichocarpa] | ESTP | mapped | RefSeq protein assignment is based on blastx similarity to Populus trichocarpa RefSeq XP\_002324047; Score = 46.2 bits (108), Expect = 9e-04, Identities = 36/44 (81%), Positives = 36/44 (81%), Gaps = 0/44 (0%) |
| estPtIFG\_8580\_a | gb|AA739590 | Pinus taeda | 8580M | Pta.11747 | [GO:0003674] molecular\_function | molecular\_function | [Lineage ends at prior level] | [Lineage ends at prior level] | [Lineage ends at prior level] | n/a | n/a | ESTP | mapped |  |
| estPtIFG\_8612\_a | gb|AA739606 | Pinus taeda | 8612M | Pta.7849 | [GO:0009269] response to desiccation | biological\_process | [GO:0050896] response to stimulus | [GO:0006950] response to stress | [GO:0009414] response to water deprivation | ref|NP\_001042461 | late embryogenesis abundant protein [Oryza sativa] | ESTP | mapped |  |
| estPtIFG\_8647\_a | gb|AA739625 | Pinus taeda | 8647M | Pta.1091 | [GO:0015250] water channel activity | molecular\_function | [GO:0005215] transporter activity | [GO:0022857] transmembrane transporter activity | [GO:0022891] substrate-specific transmembrane transporter activity | ref|NP\_001047491 | plasma membrane integral protein [Oryza sativa] | ESTP | mapped |  |
| estPtIFG\_8702\_a | gb|AA739652 | Pinus taeda | 8702M | Pta.168 | [GO:0003756] protein disulfide isomerase activity | molecular\_function | [GO:0003824] catalytic activity | [GO:0016853] isomerase activity | [GO:0016860] intramolecular oxidoreductase activity | ref|NP\_172620 | thioredoxin-like 4 protein [Arabidopsis thaliana] | ESTP | mapped |  |
| estPtIFG\_8725\_a | gb|AA739673 | Pinus taeda | 8725M | Pta.2 | [GO:0005515] protein binding | molecular\_function | [GO:0005488] binding | [GO:0005515] protein binding | [Lineage ends at prior level] | ref|NP\_199111 | constitutive photomorphogenic homolog [Arabidopsis thaliana] | ESTP | mapped |  |
| estPtIFG\_8732\_a | gb|AA739680 | Pinus taeda | 8732M | Pta.577 | [GO:0004749] ribose phosphate diphosphokinase activity | molecular\_function | [GO:0003824] catalytic activity | [GO:0016740] transferase activity | [GO:0016772] transferase activity, transferring phosphorus-containing groups | ref|NP\_001047921 | phosphoribosyl pyrophosphate synthase isozyme 4 [Oryza sativa] | ESTP | mapped |  |
| estPtIFG\_8738\_a | gb|AA739685 | Pinus taeda | 8738M | Pta.4 | [GO:0004707] MAP kinase activity | molecular\_function | [GO:0003824] catalytic activity | [GO:0016740] transferase activity | [GO:0005057] receptor signaling protein activity | ref|NP\_181907 | MAP kinase 6 [Arabidopsis thaliana] | ESTP | mapped |  |
| estPtIFG\_8781\_a | gb|AA739709 | Pinus taeda | 8781M | Pta.11769 | [GO:0005478] intracellular transporter activity | molecular\_function | [GO:0005488] binding | [GO:0005515] protein binding | [Lineage ends at prior level] | ref|NP\_191178 | alpha-soluble attachment protein [Arabidopsis thaliana] | ESTP | mapped |  |
| estPtIFG\_8837\_a | gb|AA739754 | Pinus taeda | 8837M | Pta.7714 | [GO:0004713] protein tyrosine kinase activity | molecular\_function | [GO:0003824] catalytic activity | [GO:0016740] transferase activity | [GO:0016772] transferase activity, transferring phosphorus-containing groups | ref|NP\_001043164 | MAP kinase kinase 1 [Oryza sativa] | ESTP | mapped |  |
| estPtIFG\_8886\_a | gb|AA739785 | Pinus taeda | 8886M | Pta.265 | [GO:0005524] ATP binding | molecular\_function | [GO:0005488] binding | [GO:0000166] nucleotide binding | [GO:0017076] purine nucleotide binding | ref|NP\_567346 | nucleoside diphosphate kinase [Arabidopsis thaliana] | ESTP | mapped |  |
| estPtIFG\_8887\_a | gb|AA739786 | Pinus taeda | 8887M | Pta.16 | [GO:0003899] DNA-directed RNA polymerase activity | molecular\_function | [GO:0003824] catalytic activity | [GO:0016740] transferase activity | [GO:0016772] transferase activity, transferring phosphorus-containing groups | ref|NP\_001067421 | DNA-directed RNA polymerase II 7.6 kDa polypeptide [Oryza sativa] | ESTP | mapped |  |
| estPtIFG\_8898\_a | gb|AA739796 | Pinus taeda | 8898M | Pta.664 | [GO:0000166] nucleotide binding | molecular\_function | [GO:0005488] binding | [GO:0000166] nucleotide binding | [Lineage ends at prior level] | ref|NP\_566644 | WD-40 repeat family protein [Arabidopsis thaliana] | ESTP | mapped |  |
| estPtIFG\_8939\_a | gb|AA739822 | Pinus taeda | 8939M | Pta.88 | [GO:0003735] structural constituent of ribosome | molecular\_function | [GO:0005198] structural molecule activity | [GO:0003735] structural constituent of ribosome | [Lineage ends at prior level] | ref|NP\_178826 | 40S ribosomal protein S16 [Arabidopsis thaliana] | ESTP | mapped |  |
| estPtIFG\_8972\_a | gb|AA739836 | Pinus taeda | 8972M | n/a | [GO:0015250] water channel activity | molecular\_function | [GO:0005215] transporter activity | [GO:0022857] transmembrane transporter activity | [GO:0022891] substrate-specific transmembrane transporter activity | ref|XP\_002521292 | aquaporin, PIP2.7 [Ricinus communis] | ESTP | mapped | Reference protein assignment is based on blastx similarity to Ricinus communis RefSeq XP\_002521292; Score = 167 bits (422), Expect = 8e-40, Identities = 90/122 (73%), Positives = 97/122 (79%), Gaps = 0/122 (0%) |
| estPtIFG\_9008\_a | gb|AA739854 | Pinus taeda | 9008M | Pta.642 | [GO:0005524] ATP binding | molecular\_function | [GO:0005488] binding | [GO:0000166] nucleotide binding | [GO:0017076] purine nucleotide binding | ref|NP\_001068410 | AAA-ATPase, central region domain [Oryza sativa] | ESTP | mapped |  |
| estPtIFG\_9022\_a | gb|AA739862 | Pinus taeda | 9022M | Pta.458 | [GO:0003743] translation initiation factor activity | molecular\_function | [GO:0005488] binding | [GO:0003676] nucleic acid binding | [GO:0008135] translation factor activity, nucleic acid binding | ref|NP\_175831 | eukaryotic translation initiation factor SUI1, putative [Arabidopsis thaliana] | ESTP | mapped |  |
| estPtIFG\_9036\_a | gb|AA739871 | Pinus taeda | 9036M | Pta.2677 | [GO:0003735] structural constituent of ribosome | molecular\_function | [GO:0005198] structural molecule activity | [GO:0003735] structural constituent of ribosome | [Lineage ends at prior level] | ref|NP\_172977 | 60S ribosomal protein L37 [Arabidopsis thaliana] | ESTP | mapped |  |
| estPtIFG\_9044\_a | gb|AA739876 | Pinus taeda | 9044M | Pta.8854 | [GO:0003735] structural constituent of ribosome | molecular\_function | [GO:0005198] structural molecule activity | [GO:0003735] structural constituent of ribosome | [Lineage ends at prior level] | ref|NP\_191670 | 40S ribosomal protein S27-2 [Arabidopsis thaliana] | ESTP | mapped |  |
| estPtIFG\_9053\_a | gb|AA739883 | Pinus taeda | 9053M | Pta.204 | [GO:0003674] molecular\_function | molecular\_function | [Lineage ends at prior level] | [Lineage ends at prior level] | [Lineage ends at prior level] | ref|NP\_188286 | translationally controlled tumor protein homolog [Arabidopsis thaliana] | ESTP | mapped |  |
| estPtIFG\_9076\_a | gb|AA739897 | Pinus taeda | 9076M | Pta.4262 | [GO:0004614] phosphoglucomutase activity | molecular\_function | [GO:0003824] catalytic activity | [GO:0016853] isomerase activity | [GO:0016866] intramolecular transferase activity | ref|NP\_173732 | phosphoglucomutase, cytoplasmic [Arabidopsis thaliana] | ESTP | mapped |  |
| estPtIFG\_9092\_a | gb|AA739907 | Pinus taeda | 9092M | Pta.11 | [GO:0003674] molecular\_function | molecular\_function | [Lineage ends at prior level] | [Lineage ends at prior level] | [Lineage ends at prior level] | n/a | n/a | ESTP | mapped |  |
| estPtIFG\_9102\_a | gb|AA739915 | Pinus taeda | 9102M | Pta.11375 | [GO:0005507] copper ion binding | molecular\_function | [GO:0005488] binding | [GO:0043167] ion binding | [GO:0043169] cation binding | ref|NP\_196158 | laccase 12 [Arabidopsis thaliana] | ESTP | mapped |  |
| estPtIFG\_9113\_a | gb|AA739924 | Pinus taeda | 9113M | Pta.746 | [GO:0004478] methionine adenosyltransferase activity | molecular\_function | [GO:0003824] catalytic activity | [GO:0016740] transferase activity | [GO:0016765] transferase activity, transferring alkyl or aryl (other than methyl) groups | ref|NP\_188365 | methionine adenosyltransferase [Arabidopsis thaliana] | ESTP | mapped |  |
| estPtIFG\_9151\_a | gb|AA739953 | Pinus taeda | 9151M | Pta.468 | [GO:0005507] copper ion binding | molecular\_function | [GO:0005488] binding | [GO:0043167] ion binding | [GO:0043169] cation binding | ref|NP\_001051044 | plastocyanin-like domain containing protein [Oryza sativa] | ESTP | mapped |  |
| estPtIFG\_9156\_a | gb|AA739960 | Pinus taeda | 9156aM | Pta.109 | [GO:0003735] structural constituent of ribosome | molecular\_function | [GO:0005198] structural molecule activity | [GO:0003735] structural constituent of ribosome | [Lineage ends at prior level] | ref|NP\_001047036 | 40S ribosomal protein S14 [Oryza sativa] | ESTP | mapped |  |
| estPtIFG\_9157\_a | gb|AA739961 | Pinus taeda | 9157M | Pta.671 | [GO:0005516] calmodulin binding | molecular\_function | [GO:0005488] binding | [GO:0005515] protein binding | [GO:0005516] calmodulin binding | ref|NP\_187204 | AAA-ATPase [Arabidopsis thaliana] | ESTP | mapped |  |
| estPtIFG\_9164\_a | gb|AA739966 | Pinus taeda | 9164M | Pta.1604 | [GO:0003755] peptidyl-prolyl cis-trans isomerase activity | molecular\_function | [GO:0003824] catalytic activity | [GO:0016853] isomerase activity | [GO:0016859] cis-trans isomerase activity | ref|NP\_179251 | rotamase CyP 3, cystolic cyclophilin ABH-like [Arabidopsis thaliana] | ESTP | mapped |  |
| estPtIFG\_9198\_a | gb|AA739984 | Pinus taeda | 9198M | Pta.104 | [GO:0005516] calmodulin binding | molecular\_function | [GO:0005488] binding | [GO:0005515] protein binding | [GO:0005516] calmodulin binding | ref|NP\_001032107 | elongation factor 1-alpha [Arabidopsis thaliana] | ESTP | mapped |  |
| estPtIFG\_C4H-1\_a | gb|AY764813 | Pinus taeda | n/a | Pta.6348 | [GO:0016710] trans-cinnamate 4-monooxygenase activity | molecular\_function | [GO:0003824] catalytic activity | [GO:0016491] oxidoreductase activity | [GO:0004497] monooxygenase activity | ref|NP\_180607 | trans-cinnamate 4-hydroxylase [Arabidopsis thaliana] | ESTP | mapped | UniGene assignment is based on blastn similarity to the homologous Pinus taeda EST gb|CO161408; Score = 1624 bits (879), Expect = 0.0, Identities = 881/882 (99%), Gaps = 0/882 (0%). |
| estPtIFG\_C4H-2\_a | gb|AF096998 | Pinus taeda | n/a | Pta.11490 | [GO:0016710] trans-cinnamate 4-monooxygenase activity | molecular\_function | [GO:0003824] catalytic activity | [GO:0016491] oxidoreductase activity | [GO:0004497] monooxygenase activity | ref|NP\_180607 | cinnamate-4-hydroxylase [Arabidopsis thaliana] | ESTP | mapped |  |
| estPtIFG\_COMT-2\_a | gb|DR165351 | Pinus taeda | RTPHOS1\_4\_A06\_A029 | Pta.11336 | [GO:0008171] O-methyltransferase activity | molecular\_function | [GO:0003824] catalytic activity | [GO:0016740] transferase activity | [GO:0016741] transferase activity, transferring one-carbon groups | ref|NP\_200227 | O-methyltransferase 1 [Arabidopsis thaliana] | ESTP | mapped |  |
| estPtIFG\_lp3-3 | gb|AY867630 | Pinus taeda | n/a | Pta.447 | [GO:0006950] response to stress | biological\_process | [GO:0050896] response to stimulus | [GO:0006950] response to stress | [Lineage ends at prior level] | ref|NP\_001065841 | pollen-specific desiccation-associated LLA23 protein [Oryza sativa] | ESTP | mapped | UniGene assignment is based on blastn similarity to the homologous Pinus taeda EST gb|CF390644; Score = 287 bits (155), Expect = 3e-76, Identities = 387/489 (79%), Gaps = 55/489 (11%). |
| estPtIFG\_SAHH\_a | gb|DR745467 | Pinus taeda | RTCU1\_29\_F11\_A029 | Pta.10148 | [GO:0004013] adenosylhomocysteinase activity | molecular\_function | [GO:0003824] catalytic activity | [GO:0016787] hydrolase activity | [GO:0016801] hydrolase activity, acting on ether bonds | ref|NP\_193130 | adenosylhomocysteinase [Arbidopsis thaliana] | ESTP | mapped |  |
| estPtIFG\_SB32b\_a | gb|AF051225 | Picea mariana | n/a | n/a | [GO:0051726] regulation of cell cycle | biological\_process | [GO:0009987] cellular process | [GO:0007049] cell cycle | [GO:0051726] regulation of cell cycle | ref|NP\_001053564 | cyclin [Oryza sativa] | ESTP | mapped | RefSeq protein assignment is based on blastx similarity to the Picea mariana seq. gb|AF051225. Score = 261 bits (667), Expect = 5e-68, Identities = 129/215 (60%), Positives = 161/215 (74%), Gaps = 0/215 (0%). |
| estPtNCS\_22B8\_a | gb|AI812891 | Pinus taeda | 22B8 | Pta.11129 | [GO:0004372] glycine hydroxymethyltransferase activity | molecular\_function | [GO:0003824] catalytic activity | [GO:0016740] transferase activity | [GO:0016741] transferase activity, transferring one-carbon groups | ref|NP\_193129 | serine hydroxymethlytransferase 4 [Arabidopsis thaliana] | ESTP | mapped |  |
| estPtNCS\_22C5\_a | gb|AI812900 | Pinus taeda | 22C5 | Pta.224 | [GO:0004512] inositol-3-phosphate synthase activity | molecular\_function | [GO:0003824] catalytic activity | [GO:0016853] isomerase activity | [GO:0016872] intramolecular lyase activity | ref|NP\_973509 | inositol-3-phosphate synthase [Arabidopsis thaliana] | ESTP | mapped |  |
| estPtNCS\_22C8\_a | gb|AI812903 | Pinus taeda | 22C8 | Pta.9400 | [GO:0016688] L-ascorbate peroxidase activity | molecular\_function | [GO:0003824] catalytic activity | [GO:0016491] oxidoreductase activity | [GO:0016684] oxidoreductase activity, acting on peroxide as acceptor | ref|NP\_001030991 | L-ascorbate peroxidase 1 [Arabidopsis thaliana] | ESTP | mapped |  |
| estPtNCS\_23C5\_a | gb|AI813128 | Pinus taeda | 23C5 | Pta.8176 | [GO:0016841] ammonia-lyase activity | molecular\_function | [GO:0003824] catalytic activity | [GO:0016829] lyase activity | [GO:0016840] carbon-nitrogen lyase activity | ref|NP\_187645 | phenylalanine ammonia-lyase 4 [Aribidopsis thaliana] | ESTP | mapped |  |
| estPtNCS\_2N7G\_a | gb|AA556198 | Pinus taeda | 2N7G | Pta.10987 | [GO:0003924] GTPase activity | molecular\_function | [GO:0003824] catalytic activity | [GO:0016787] hydrolase activity | [GO:0016817] hydrolase activity, acting on acid anhydrides | ref|NP\_171974 | tubulin alpha-4 chain [Arabidopsis thaliana] | ESTP | mapped |  |
| estPtNCS\_6C12A\_a | gb|AA556806 | Pinus taeda | 6C12A | n/a | [GO:0003924] GTPase activity | molecular\_function | [GO:0003824] catalytic activity | [GO:0016787] hydrolase activity | [GO:0016817] hydrolase activity, acting on acid anhydrides | ref|NP\_001048064 | dynamin family protein [Oryza sativa] | ESTP | mapped | RefSeq protein assignment is based on blastx similarity to the GenBank seq.; Score = 277 bits (708), Expect = 4e-73, Identities = 136/191 (71%), Positives = 164/191 (85%), Gaps = 1/191 (0%). |
| estPtNCS\_6C5A\_a | gb|AA556842 | Pinus taeda | 6C5A | Pta.4462 | [GO:0010283] pinoresinol reductase activity | molecular\_function | [GO:0003824] catalytic activity | [GO:0016491] oxidoreductase activity | [GO:0016627] oxidoreductase activity, acting on the CH-CH group of donors | ref|NP\_174490 | pinoresinol-lariciresinol reductase, putative [Arabidopsis thaliana] | ESTP | mapped |  |
| estPtNCS\_6N3C\_a | gb|AA557072 | Pinus taeda | 6N3C | n/a | [GO:0004553] hydrolase activity, hydrolyzing O-glycosyl compounds | molecular\_function | [GO:0003824] catalytic activity | [GO:0016787] hydrolase activity | [GO:0016798] hydrolase activity, acting on glycosyl bonds | ref|NP\_177228 | glycosyl hydrolase 9B1 [Arabidopsis thaliana] | ESTP | mapped | RefSeq protein assignment is based on blastx similiarity to the GenBank seq.; Score = 95.1 bits (235), Expect = 2e-18, Identities = 56/86 (65%), Positives = 67/86 (77%), Gaps = 0/86 (0%) |
| estPtNCS\_6N3E\_a | gb|AA557074 | Pinus taeda | 6N3E | Pta.5807 | [GO:0016615] malate dehydrogenase activity | molecular\_function | [GO:0003824] catalytic activity | [GO:0016491] oxidoreductase activity | [GO:0016614] oxidoreductase activity, acting on CH-OH group of donors | ref|NP\_171936 | malate dehydrogenase, cytoplasmic 1 [Aribidopsis thaliana] | ESTP | mapped |  |
| estPtNCS\_CCoAOMT\_a | gb|AF036095 | Pinus taeda | n/a | Pta.11406 | [GO:0008171] O-methyltransferase activity | molecular\_function | [GO:0003824] catalytic activity | [GO:0016740] transferase activity | [GO:0016741] transferase activity, transferring one-carbon groups | ref|NP\_195131 | caffeoyl-CoA 3-O-methyltransferase, putative [Arabidopsis thaliana] | ESTP | mapped |  |
| NZPR0102c | n/a | Pinus radiata | n/a | n/a | n/a | n/a | n/a | n/a | n/a | n/a | n/a | SSR | mapped |  |
| NZPR0116 | n/a | Pinus radiata | n/a | n/a | n/a | n/a | n/a | n/a | n/a | n/a | n/a | SSR | mapped |  |
| NZPR0143 | n/a | Pinus radiata | n/a | n/a | n/a | n/a | n/a | n/a | n/a | n/a | n/a | SSR | mapped |  |
| NZPR0206 | gb|BV728907 | Pinus radiata | n/a | n/a | n/a | n/a | n/a | n/a | n/a | n/a | n/a | SSR | mapped |  |
| NZPR0269 | gb|BV728908 | Pinus radiata | n/a | n/a | n/a | n/a | n/a | n/a | n/a | n/a | n/a | SSR | mapped |  |
| NZPR0274 | gb|BV728909 | Pinus radiata | n/a | n/a | n/a | n/a | n/a | n/a | n/a | n/a | n/a | SSR | mapped |  |
| NZPR0290 | gb|BV728910 | Pinus radiata | n/a | n/a | n/a | n/a | n/a | n/a | n/a | n/a | n/a | SSR | mapped |  |
| NZPR0300 | gb|BV728911 | Pinus radiata | n/a | n/a | n/a | n/a | n/a | n/a | n/a | n/a | n/a | SSR | mapped |  |
| NZPR0351 | gb|BV728912 | Pinus radiata | n/a | n/a | n/a | n/a | n/a | n/a | n/a | n/a | n/a | SSR | mapped |  |
| NZPR0413 | gb|BV728913 | Pinus radiata | n/a | n/a | n/a | n/a | n/a | n/a | n/a | n/a | n/a | SSR | mapped |  |
| NZPR0440 | gb|BV728914 | Pinus radiata | n/a | n/a | n/a | n/a | n/a | n/a | n/a | n/a | n/a | SSR | mapped |  |
| NZPR0458 | gb|BV728929 | Pinus radiata | n/a | n/a | n/a | n/a | n/a | n/a | n/a | n/a | n/a | SSR | mapped |  |
| NZPR0473 | gb|BV728915 | Pinus radiata | n/a | n/a | n/a | n/a | n/a | n/a | n/a | n/a | n/a | SSR | mapped |  |
| NZPR0563 | gb|BV728916 | Pinus radiata | n/a | n/a | n/a | n/a | n/a | n/a | n/a | n/a | n/a | SSR | mapped |  |
| NZPR0599 | gb|BV728917 | Pinus radiata | n/a | n/a | n/a | n/a | n/a | n/a | n/a | n/a | n/a | SSR | mapped |  |
| NZPR0826 | gb|BV728918 | Pinus radiata | n/a | n/a | n/a | n/a | n/a | n/a | n/a | n/a | n/a | SSR | mapped |  |
| NZPR0917 | gb|BV728919 | Pinus radiata | n/a | n/a | n/a | n/a | n/a | n/a | n/a | n/a | n/a | SSR | mapped |  |
| NZPR0933 | gb|BV728920 | Pinus radiata | n/a | n/a | n/a | n/a | n/a | n/a | n/a | n/a | n/a | SSR | mapped |  |
| NZPR0943 | gb|BV728921 | Pinus radiata | n/a | n/a | n/a | n/a | n/a | n/a | n/a | n/a | n/a | SSR | mapped |  |
| NZPR0947 | gb|BV728922 | Pinus radiata | n/a | n/a | n/a | n/a | n/a | n/a | n/a | n/a | n/a | SSR | mapped |  |
| NZPR1004 | gb|BV728923 | Pinus radiata | n/a | n/a | n/a | n/a | n/a | n/a | n/a | n/a | n/a | SSR | mapped |  |
| NZPR1078 | gb|BV728924 | Pinus radiata | n/a | n/a | n/a | n/a | n/a | n/a | n/a | n/a | n/a | SSR | mapped |  |
| NZPR1680 | gb|BV728925 | Pinus radiata | n/a | n/a | n/a | n/a | n/a | n/a | n/a | n/a | n/a | SSR | mapped |  |
| NZPR1682 | gb|BV728926 | Pinus radiata | n/a | n/a | n/a | n/a | n/a | n/a | n/a | n/a | n/a | SSR | mapped |  |
| NZPR1699 | gb|BV728927 | Pinus radiata | n/a | n/a | n/a | n/a | n/a | n/a | n/a | n/a | n/a | SSR | mapped |  |
| NZPR1702\_b | gb|BV728928 | Pinus radiata | n/a | n/a | n/a | n/a | n/a | n/a | n/a | n/a | n/a | SSR | mapped |  |
| PbRAMS | gb|U38186 | Pinus banksiana | n/a | Pta.11623 | [GO:0004478] methionine adenosyltransferase activity | molecular\_function | [GO:0003824] catalytic activity | [GO:0016740] transferase activity | [GO:0016765] transferase activity, transferring alkyl or aryl (other than methyl) groups | ref|NP\_188365 | S-adenosylmethionine synthase [Arabidopsis thaliana] | ESTP | mapped | UniGene assignment is based on blastn similarity to the homologous Pinus taeda EST gb|DR014448; Score = 1528 bits (827), Expect = 0.0, Identities = 887/914 (97%), Gaps = 12/914 (1%). |
| PpSIFG\_3116 | gb|CT579357 | Pinus pinaster | n/a | n/a | [GO:0009228] thiamin biosynthetic process | molecular\_function | [GO:0008152] metabolic process | [GO:0044237] cellular metabolic process | [GO:0006766] vitamin metabolic process | ref|NP\_001059841 | thiazole biosynthetic enzyme 1-1, chloroplast precorsor [Oryza sativa] | SSR | not mapped | RefSeq protein assignment is based on blastx similarity to the GenBank seq.; Score = 275 bits (702), Expect = 2e-72, Identities = 134/158 (84%), Positives = 147/158 (93%), Gaps = 0/158 (0%). |
| PpSIFG\_3129 | gb|BX680424 | Pinus pinaster | RS43C10 | Pta.617 | [GO:0003700] transcription factor activity | molecular\_function | [GO:0005488] binding | [GO:0003676] nucleic acid binding | [GO:0003677] DNA binding | ref|NP\_001055941 | ethylene responsive element DNA binding factor 5 [Oryza sativa] | SSR | mapped | UniGene assignment is based on blastn similarity to the homologous Pinus taeda EST gb|CX715090; Score = 721 bits (390), Expect = 0.0, Identities = 416/428 (97%), Gaps = 4/428 (0%). |
| PpSIFG\_3145 | gb|BX254108 | Pinus pinaster | PP093E03 | Pta.14877 | [GO:0003674] molecular\_function | molecular\_function | [Lineage ends at prior level] | [Lineage ends at prior level] | [Lineage ends at prior level] | n/a | n/a | SSR | mapped | UniGene assignment is based on blastn similarity to the homologous Pinus taeda EST gb|DR116962. |
| PpSIFG\_3147 | gb|BX254283 | Pinus pinaster | PP096B11 | Pta.13015 | [GO:0003674] molecular\_function | molecular\_function | [Lineage ends at prior level] | [Lineage ends at prior level] | [Lineage ends at prior level] | ref|NP\_199801 | unknown protein [Arabidopsis thaliana] | SSR | mapped | UniGene assignment is based on blastn similarity to the homologous Pinus taeda EST gb|DR012612; Score = 1051 bits (569), Expect = 0.0, Identities = 633/662 (95%), Gaps = 12/662 (1%). |
| PpSIFG\_3168 | gb|BX679043 | Pinus pinaster | RS18H03 | n/a | [GO:0003674] molecular\_function | molecular\_function | [Lineage ends at prior level] | [Lineage ends at prior level] | [Lineage ends at prior level] | n/a | n/a | SSR | not mapped |  |
| PrCHS1 | gb|U90341 | Pinus radiata | pAW192 | n/a | [GO:0008415] acyltransferase activity | molecular\_function | [GO:0003824] catalytic activity | [GO:0016740] transferase activity | [GO:0016746] transferase activity, transferring acyl groups | ref|NP\_171707 | chalcone and stilbene synthase family protein [Arabidopsis thaliana] | ESTP | mapped | RefSeq protein assignment is based on blastx similiarity to the GenBank seq.; Score = 84.0 bits (206), Expect = 3e-14, Identities = 142/211 (67%), Positives = 173/211 (81%), Gaps = 0/211 (0%) |
| PrE79 | gb|AF049066 | Pinus radiata | n/a | n/a | [GO:0003674] molecular\_function | molecular\_function | [Lineage ends at prior level] | [Lineage ends at prior level] | [Lineage ends at prior level] | n/a | n/a | ESTP | mapped |  |
| PrMADS3 | gb|U76726 | Pinus radiata | n/a | Pta.1542 | [GO:0003677] DNA binding | molecular\_function | [GO:0005488] binding | [GO:0003676] nucleic acid binding | [GO:0003677] DNA binding | ref|NP\_001105692 | MADS-box protein [Zea mays] | ESTP | mapped | UniGene assignment is based on blastn similarity to the homologous Pinus taeda EST gb|DR058453; Score = 795 bits (430), Expect = 0.0, Identities = 435/437 (99%), Gaps = 1/437 (0%). |
| PstASU\_APX | gb|AF326783 | Pinus strobus | n/a | Pta.4666 | [GO:0016688] L-ascorbate peroxidase activity | molecular\_function | [GO:0003824] catalytic activity | [GO:0016491] oxidoreductase activity | [GO:0016684] oxidoreductase activity, acting on peroxide as acceptor | ref|NP\_001105500 | ascorbate peroxidase 2 [Zea mays] | ESTP | mapped | UniGene assignment is based on blastn similarity to the homologous Pinus taeda EST gb|DR163761; Score = 850 bits (460), Expect = 0.0, Identities = 532/568 (93%), Gaps = 0/568 (0%). |
| PsUME\_Ps3\_A | gb|X58578 | Pinus sylvestris | PS3 | Pta.145 | [GO:0004785] copper, zinc superoxide dismutase activity | molecular\_function | [GO:0003824] catalytic activity | [GO:0016491] oxidoreductase activity | [GO:0016721] oxidoreductase activity, acting on superoxide radicals as acceptor | ref|NP\_001077494 | copper/zinc superoxide dismutase 1 [Arabidopsis thaliana] | RFLP | mapped | UniGene assignment is based on blastn similarity to the homologous Pinus taeda EST gb|DR052077; Score = 1319 bits (714), Expect = 0.0, Identities = 774/803 (96%), Gaps = 4/803 (0%). |
| PsUME\_S43\_63 | n/a | Pinus sylvestris | n/a | n/a | [GO:0003674] molecular\_function | molecular\_function | [Lineage ends at prior level] | [Lineage ends at prior level] | [Lineage ends at prior level] | n/a | n/a | RFLP | mapped | No sequence available. |
| PsUPS2\_PST13 | gb|X58579 | Pinus sylvestris | PST13 | Pta.5825 | [GO:0004785] copper, zinc superoxide dismutase activity | molecular\_function | [GO:0003824] catalytic activity | [GO:0016491] oxidoreductase activity | [GO:0016721] oxidoreductase activity, acting on superoxide radicals as acceptor | ref|NP\_001062514 | superoxide dismutase [Cu/Zn], chloroplast precursor [Oryza sativa] | ESTP | not mapped | UniGene assignment is based on blastn similarity to the homologous Pinus taeda EST gb|DR163905: Score = 1153 bits (624), Expect = 0.0, Identities = 651/663 (98%), Gaps = 5/663 (0%). |
| PsyGPD | gb|L26923 | Pinus sylvestris | n/a | Pta.598 | [GO:0008943] glyceraldehyde-3-phosphate dehydrogenase activity | molecular\_function | [GO:0003824] catalytic activity | [GO:0016491] oxidoreductase activity | [GO:0016903] oxidoreductase activity, acting on the aldehyde or oxo group of donors | ref|NP\_172750 | glyceraldehyde-3-phosphate dehydrogenase [Arabidopsis thaliana] | ESTP | mapped | UniGene assignment is based on blastn similarity to the homologous Pinus taeda EST gb|CO360904; Score = 1530 bits (828), Expect = 0.0, Identities = 867/885 (97%), Gaps = 5/885 (0%). |
| PtAGP | gb|U09554 | Pinus taeda | p3H6/CDM8 | Pta.102 | [GO:0010405] arabinogalactan protein metabolic process | biological\_process | [GO:0009987] cellular process | [GO:0070882] cell wall organization or biogenesis | [GO:0044036] cell wall macromolecule metabolic process | gb|AAA74419 | arabinogalactan-like protein [Pinus taeda] | ESTP | mapped |  |
| PthCAB | gb|X13407 | Pinus thunbergii | n/a | Pta.4922 | [GO:0016168] chlorophyll binding | molecular\_function | [GO:0005488] binding | [GO:0046906] tetrapyrrole binding | [GO:0016168] chlorophyll binding | ref|NP\_189406 | photosystem II light harvesting complex gene 2.3 [Arabidopsis thaliana] | ESTP | mapped | UniGene assignment is based on blastn similarity to the homologous Pinus taeda EST gb|DR023978; Score = 1554 bits (841), Expect = 0.0, Identities = 896/921 (97%), Gaps = 12/921 (1%). |
| PitaIFG\_1A7\_6 | gb|AI812330 | Pinus taeda | 1A7 | Pta.2426 | [GO:0006950] response to stress | biological\_process | [GO:0050896] response to stimulus | [GO:0006950] response to stress | [Lineage ends at prior level] | ref|NP\_001065841 | pollen-specific desiccation-associated LLA23 protein [Oryza sativa] | RFLP | mapped |  |
| PitaIFG\_2020\_1 | gb|H75044 | Pinus taeda | 2020e | Pta.777 | [GO:0004001] adenosine kinase activity | molecular\_function | [GO:0003824] catalytic activity | [GO:0016740] transferase activity | [GO:0016772] transferase activity, transferring phosphorus-containing groups | ref|NP\_187593 | adenosine kinase 1(ADK1) [A. thaliana] | RFLP | mapped |  |
| PitaIFG\_2361\_1 | gb|H75090 | Pinus taeda | 2361s | Pta.1807 | [GO:0016168] chlorophyll binding | molecular\_function | [GO:0005488] binding | [GO:0046906] tetrapyrrole binding | [GO:0016168] chlorophyll binding | ref|NP\_176347 | chlorophyll a/b binding protein, [Arabidopsis thaliana] | RFLP | mapped |  |
| PtIFG\_1165\_a | gb|H75179 | Pinus taeda | 1165e | Pta.598 | [GO:0008943] glyceraldehyde-3-phosphate dehydrogenase activity | molecular\_function | [GO:0003824] catalytic activity | [GO:0016491] oxidoreductase activity | [GO:0016903] oxidoreductase activity, acting on the aldehyde or oxo group of donors | ref|NP\_172750 | glyceraldehyde-3-phosphate dehydrogenase [Arabidopsis thaliana] | RFLP | mapped |  |
| PtIFG\_138\_A | gb|H75134 | Pinus taeda | 0138e | Pta.11535 | [GO:0003746] translation elongation factor activity | molecular\_function | [GO:0005488] binding | [GO:0003676] nucleic acid binding | [GO:0008135] translation factor activity, nucleic acid binding | ref|NP\_193769 | Rab GTPase homolog E1b [Arabidopsis thaliana] | RFLP | mapped |  |
| PtIFG\_138\_B | gb|H75135 | Pinus taeda | 0138s | n/a | [GO:0003746] translation elongation factor activity | molecular\_function | [GO:0005488] binding | [GO:0003676] nucleic acid binding | [GO:0008135] translation factor activity, nucleic acid binding | ref|NP\_001150410 | elongation factor Ut [Zea mays] | RFLP | mapped | RefSeq protein assignment is based on blastx similarity to Zea mays RefSeq NP\_001150410; Score = 47.0 bits (110), Expect = 5e-04, Identities = 24/31 (77%), Positives = 26/31 (83%), Gaps = 1/31 (3%) |
| PtIFG\_1454\_A | gb|H75120 | Pinus taeda | 1454J | Pta.11571 | [GO:0003674] molecular\_function | molecular\_function | [Lineage ends at prior level] | [Lineage ends at prior level] | [Lineage ends at prior level] | n/a | n/a | RFLP | mapped |  |
| PtIFG\_1457\_A | gb|H75107 | Pinus taeda | 1457M | Pta.572 | [GO:0003674] molecular\_function | molecular\_function | [Lineage ends at prior level] | [Lineage ends at prior level] | [Lineage ends at prior level] | ref|NP\_001066338 | unknown protein [Oryza sativa] | RFLP | mapped |  |
| PtIFG\_149\_2 | gb|H75141 | Pinus taeda | 0149s | Pta.1913 | [GO:0004034] aldose 1-epimerase activity | molecular\_function | [GO:0003824] catalytic activity | [GO:0016853] isomerase activity | [GO:0016854] racemase and epimerase activity | ref|NP\_200543 | aldose 1-epimerase family protein [Arabidopsis thaliana] | RFLP | mapped |  |
| PtIFG\_149\_A | gb|H75140 | Pinus taeda | 0149e | Pta.1913 | [GO:0004034] aldose 1-epimerase activity | molecular\_function | [GO:0003824] catalytic activity | [GO:0016853] isomerase activity | [GO:0016854] racemase and epimerase activity | ref|NP\_200543 | aldose 1-epimerase family protein [Arabidopsis thaliana] | RFLP | mapped |  |
| PtIFG\_1588\_A | gb|H75108 | Pinus taeda | 1588M | Pta.11569 | [GO:0003674] molecular\_function | molecular\_function | [Lineage ends at prior level] | [Lineage ends at prior level] | [Lineage ends at prior level] | n/a | n/a | RFLP | mapped |  |
| PtIFG\_1593\_21 | n/a | Pinus taeda | n/a | n/a | [GO:0003674] molecular\_function | molecular\_function | [Lineage ends at prior level] | [Lineage ends at prior level] | [Lineage ends at prior level] | n/a | n/a | RFLP | mapped | No sequence available. |
| PtIFG\_1599 | gb|H75181 | Pinus taeda | 1599e | Pta.599 | [GO:0046933] hydrogen ion transporting ATP synthase activity, rotational mechanism | molecular\_function | [GO:0005215] transporter activity | [GO:0022857] transmembrane transporter activity | [GO:0022891] substrate-specific transmembrane transporter activity | ref|NP\_192703 | ATP synthase delta chain [Arabidopsis thaliana] | RFLP | mapped |  |
| PtIFG\_1623\_A | gb|H75100 | Pinus taeda | 1623T | Pta.573 | [GO:0003674] molecular\_function | molecular\_function | [Lineage ends at prior level] | [Lineage ends at prior level] | [Lineage ends at prior level] | ref|NP\_563993 | unknown protein [Arabidopsis thaliana] | RFLP | mapped |  |
| PtIFG\_1626\_c | gb|H75183 | Pinus taeda | 1626e | Pta.730 | [GO:0005200] structural constituent of cytoskeleton | molecular\_function | [GO:0005198] structural molecule activity | [GO:0005200] structural constituent of cytoskeleton | [Lineage ends at prior level] | ref|NP\_001048679 | tubulin beta-1 chain 9 [Oryza sativa] | RFLP | mapped |  |
| PtIFG\_1633\_c | gb|H75186 | Pinus taeda | 1633s | Pta.5924 | [GO:0008137] NADH dehydrogenase (ubiquinone) activity | molecular\_function | [GO:0003824] catalytic activity | [GO:0016491] oxidoreductase activity | [GO:0016651] oxidoreductase activity, acting on NADH or NADPH | ref|NP\_001056000 | NADH-ubiquinone oxidoreductase 24 kDa mitochondrial precursor [Oryza sativa] | RFLP | mapped |  |
| PtIFG\_1635\_A | gb|H75110 | Pinus taeda | 1635M | Pta.591 | [GO:0016984] ribulose-bisphosphate carboxylase activity | molecular\_function | [GO:0003824] catalytic activity | [GO:0016829] lyase activity | [GO:0016830] carbon-carbon lyase activity | ref|NP\_176880 | ribulose bisphosphate carboxylase small chain 1A [Arabidopsis thaliana] | RFLP | mapped |  |
| PtIFG\_1636\_2 | gb|H75187 | Pinus taeda | 1636s | Pta.478 | [GO:0003824] catalytic activity | molecular\_function | [GO:0003824] catalytic activity | [Lineage ends at prior level] | [Lineage ends at prior level] | ref|NP\_187837 | tetrahydrofolate dehydrogenase/cyclohydrolase, putative [Arabidopsis thaliana] | RFLP | mapped |  |
| PtIFG\_1636\_3 | gb|H75187 | Pinus taeda | 1636s | Pta.478 | [GO:0003824] catalytic activity | molecular\_function | [GO:0003824] catalytic activity | [Lineage ends at prior level] | [Lineage ends at prior level] | ref|NP\_187837 | tetrahydrofolate dehydrogenase/cyclohydrolase, putative [Arabidopsis thaliana] | RFLP | mapped |  |
| PtIFG\_1636\_54 | gb|H75187 | Pinus taeda | 1636s | Pta.478 | [GO:0003824] catalytic activity | molecular\_function | [GO:0003824] catalytic activity | [Lineage ends at prior level] | [Lineage ends at prior level] | ref|NP\_187837 | tetrahydrofolate dehydrogenase/cyclohydrolase, putative [Arabidopsis thaliana] | RFLP | mapped |  |
| PtIFG\_1672\_A | gb|H75193 | Pinus taeda | 1672e | Pta.2518 | [GO:0003674] molecular\_function | molecular\_function | [Lineage ends at prior level] | [Lineage ends at prior level] | [Lineage ends at prior level] | ref|NP\_566244 | transmembrane protein, putative [Arabidopsis thaliana] | RFLP | mapped |  |
| PtIFG\_1869\_2 | gb|H75024 | Pinus taeda | 1869e | Pta.5848 | [GO:0008233] peptidase activity | molecular\_function | [GO:0003824] catalytic activity | [GO:0016787] hydrolase activity | [GO:0008233] peptidase activity | ref|NP\_001049162 | proteasome subunit alpha type 6 [Oryza sativa] | RFLP | mapped |  |
| PtIFG\_1889\_1 | gb|H75026 | Pinus taeda | 1889s | Pta.1211 | [GO:0047918] GDP-mannose 3,5-epimerase activity | molecular\_function | [GO:0003824] catalytic activity | [GO:0016853] isomerase activity | [GO:0016854] racemase and epimerase activity | ref|NP\_198236 | GDP-mannose 3,5-epimerase [Arabidopsis thaliana] | RFLP | mapped |  |
| PtIFG\_1902\_1 | gb|H75027 | Pinus taeda | 1902s | Pta.3379 | [GO:0005515] protein binding | molecular\_function | [GO:0005488] binding | [GO:0005515] protein binding | [Lineage ends at prior level] | ref|NP\_001061856 | 14-3-3-like protein GF14 psi [Oryza sativa] | RFLP | mapped |  |
| PtIFG\_1916\_1 | gb|H75030 | Pinus taeda | 1916s | Pta.2960 | [GO:0031072] heat shock protein binding | molecular\_function | [GO:0005488] binding | [GO:0005515] protein binding | [GO:0031072] heat shock protein binding | ref|NP\_188036 | heat shock N-terminal domain-containing protein [Arabidopsis thaliana] | RFLP | mapped |  |
| PtIFG\_1916\_2 | gb|H75030 | Pinus taeda | 1916s | Pta.2960 | [GO:0031072] heat shock protein binding | molecular\_function | [GO:0005488] binding | [GO:0005515] protein binding | [GO:0031072] heat shock protein binding | ref|NP\_188036 | heat shock N-terminal domain-containing protein [Arabidopsis thaliana] | RFLP | mapped |  |
| PtIFG\_1916\_4 | gb|H75030 | Pinus taeda | 1916s | Pta.2960 | [GO:0031072] heat shock protein binding | molecular\_function | [GO:0005488] binding | [GO:0005515] protein binding | [GO:0031072] heat shock protein binding | ref|NP\_188036 | heat shock N-terminal domain-containing protein [Arabidopsis thaliana] | RFLP | mapped |  |
| PtIFG\_1917\_A | gb|H75124 | Pinus taeda | 1917J | Pta.21752 | [GO:0003735] structural constituent of ribosome | molecular\_function | [GO:0005198] structural molecule activity | [GO:0003735] structural constituent of ribosome | [Lineage ends at prior level] | ref|NP\_001077793 | 30S ribosomal protein 3, putative, plastid-specific [Arabidopsis thaliana] | RFLP | mapped | UniGene assignment is based on blastn similarity to the homologous Pinus taeda EST gb|DR110213; Score = 363 bits (196), Expect = 4e-99, Identities = 288/332 (86%), Gaps = 8/332 (2%). |
| PtIFG\_1918\_3 | gb|H75111 | Pinus taeda | 1918M | Pta.84 | [GO:0003779] actin binding | molecular\_function | [GO:0005488] binding | [GO:0005515] protein binding | [GO:0008092] cytoskeletal protein binding | ref|NP\_565719 | actin depolymerizing factor 6 [Arabidopsis thaliana] | RFLP | mapped |  |
| PtIFG\_1918\_A | gb|H75102 | Pinus taeda | 1918T | Pta.84 | [GO:0003779] actin binding | molecular\_function | [GO:0005488] binding | [GO:0005515] protein binding | [GO:0008092] cytoskeletal protein binding | ref|NP\_565719 | actin depolymerizing factor 6 [Arabidopsis thaliana] | RFLP | mapped |  |
| PtIFG\_1918\_b | gb|H75102 | Pinus taeda | 1918T | Pta.84 | [GO:0003779] actin binding | molecular\_function | [GO:0005488] binding | [GO:0005515] protein binding | [GO:0008092] cytoskeletal protein binding | ref|NP\_565719 | actin depolymerizing factor 6 [Arabidopsis thaliana] | RFLP | not mapped |  |
| PtIFG\_1918\_f | gb|H75102 | Pinus taeda | 1918T | Pta.84 | [GO:0003779] actin binding | molecular\_function | [GO:0005488] binding | [GO:0005515] protein binding | [GO:0008092] cytoskeletal protein binding | ref|NP\_565719 | actin depolymerizing factor 6 [Arabidopsis thaliana] | RFLP | mapped |  |
| PtIFG\_1918\_h | gb|H75102 | Pinus taeda | 1918T | Pta.84 | [GO:0003779] actin binding | molecular\_function | [GO:0005488] binding | [GO:0005515] protein binding | [GO:0008092] cytoskeletal protein binding | ref|NP\_565719 | actin depolymerizing factor 6 [Arabidopsis thaliana] | RFLP | mapped |  |
| PtIFG\_1A2\_C | n/a | Pinus taeda | n/a | n/a | [GO:0003674] molecular\_function | molecular\_function | [Lineage ends at prior level] | [Lineage ends at prior level] | [Lineage ends at prior level] | n/a | n/a | RFLP | mapped | No sequence available. |
| PtIFG\_1A7\_A | gb|AI812330 | Pinus taeda | 1A7 | Pta.2426 | [GO:0006950] response to stress | biological\_process | [GO:0050896] response to stimulus | [GO:0006950] response to stress | [Lineage ends at prior level] | ref|NP\_001065841 | pollen-specific desiccation-associated LLA23 protein [Oryza sativa] | RFLP | mapped |  |
| PtIFG\_1D11\_A | n/a | Pinus taeda | n/a | n/a | [GO:0003674] molecular\_function | molecular\_function | [Lineage ends at prior level] | [Lineage ends at prior level] | [Lineage ends at prior level] | n/a | n/a | RFLP | mapped | No sequence available. |
| PtIFG\_1D9\_2 | n/a | Pinus taeda | n/a | n/a | [GO:0003674] molecular\_function | molecular\_function | [Lineage ends at prior level] | [Lineage ends at prior level] | [Lineage ends at prior level] | n/a | n/a | RFLP | mapped | No sequence available. |
| PtIFG\_2006\_C | gb|H75042 | Pinus taeda | 2006s | Pta.4922 | [GO:0016168] chlorophyll binding | molecular\_function | [GO:0005488] binding | [GO:0046906] tetrapyrrole binding | [GO:0016168] chlorophyll binding | ref|NP\_189406 | chlorophyll a/b binding protein, photosystem II [Arabidopsis thaliana] | RFLP | mapped |  |
| PtIFG\_2009\_A | n/a | Pinus taeda | n/a | n/a | [GO:0003674] molecular\_function | molecular\_function | [Lineage ends at prior level] | [Lineage ends at prior level] | [Lineage ends at prior level] | n/a | n/a | RFLP | mapped | No sequence available. |
| PtIFG\_2022\_A | gb|H75128 | Pinus taeda | 2022J | Pta.11545 | [GO:0004356] glutamate-ammonia ligase activity | molecular\_function | [GO:0003824] catalytic activity | [GO:0016874] ligase activity | [GO:0016879] ligase activity, forming carbon-nitrogen bonds | ref|NP\_001048045 | glutamine synthetase [Oryza sativa] | RFLP | mapped |  |
| PtIFG\_2068\_A | gb|H75130 | Pinus taeda | 2068J | Pta.598 | [GO:0008943] glyceraldehyde-3-phosphate dehydrogenase activity | molecular\_function | [GO:0003824] catalytic activity | [GO:0016491] oxidoreductase activity | [GO:0016903] oxidoreductase activity, acting on the aldehyde or oxo group of donors | ref|NP\_172750 | glyceraldehyde-3-phosphate dehydrogenase [Arabidopsis thaliana] | RFLP | mapped |  |
| PtIFG\_2086\_13 | gb|H75047 | Pinus taeda | n/a | Pta.5885 | [GO:0005498] sterol carrier activity | molecular\_function | [GO:0005488] binding | [GO:0008289] lipid binding | [GO:0005496] steroid binding | ref|NP\_001057119 | sterol-binding domain containing protein [Oryza sativa] | RFLP | mapped |  |
| PtIFG\_2086\_2 | gb|H75047 | Pinus taeda | n/a | Pta.5885 | [GO:0005498] sterol carrier activity | molecular\_function | [GO:0005488] binding | [GO:0008289] lipid binding | [GO:0005496] steroid binding | ref|NP\_001057119 | sterol-binding domain containing protein [Oryza sativa] | RFLP | mapped |  |
| PtIFG\_2090\_1 | gb|H75048 | Pinus taeda | 2090e | Pta.6038 | [GO:0003735] structural constituent of ribosome | molecular\_function | [GO:0005198] structural molecule activity | [GO:0003735] structural constituent of ribosome | [Lineage ends at prior level] | ref|NP\_200875 | 60S ribosomal protein L12 [Arbidopsis thaliana] | RFLP | mapped | UniGene assignment is based on blastn similarity to the homologous Pinus taeda EST gb|DR116803; Score = 267 bits (144), Expect = 1e-7, Identities = 203/227 (89%), Gaps = 22/227 (9%). |
| PtIFG\_2090\_2 | gb|H75048 | Pinus taeda | 2090e | Pta.6038 | [GO:0003735] structural constituent of ribosome | molecular\_function | [GO:0005198] structural molecule activity | [GO:0003735] structural constituent of ribosome | [Lineage ends at prior level] | ref|NP\_200875 | 60S ribosomal protein L12 [Arbidopsis thaliana] | RFLP | mapped | UniGene assignment is based on blastn similarity to the homologous Pinus taeda EST gb|DR116803; Score = 267 bits (144), Expect = 1e-7, Identities = 203/227 (89%), Gaps = 22/227 (9%). |
| PtIFG\_2090\_4 | gb|H75048 | Pinus taeda | 2090e | Pta.6038 | [GO:0003735] structural constituent of ribosome | molecular\_function | [GO:0005198] structural molecule activity | [GO:0003735] structural constituent of ribosome | [Lineage ends at prior level] | ref|NP\_200875 | 60S ribosomal protein L12 [Arbidopsis thaliana] | RFLP | mapped | UniGene assignment is based on blastn similarity to the homologous Pinus taeda EST gb|DR116803; Score = 267 bits (144), Expect = 1e-7, Identities = 203/227 (89%), Gaps = 22/227 (9%). |
| PtIFG\_2113\_1 | gb|H75052 | Pinus taeda | 2113s | Pta.11554 | [GO:0004356] glutamate-ammonia ligase activity | molecular\_function | [GO:0003824] catalytic activity | [GO:0016874] ligase activity | [GO:0016879] ligase activity, forming carbon-nitrogen bonds | ref|NP\_568335 | glutamate-ammonia ligase [Arabidopsis thaliana] | RFLP | mapped |  |
| PtIFG\_2145\_1 | gb|H75056 | Pinus taeda | 2145s | Pta.2729 | [GO:0008415] acyltransferase activity | molecular\_function | [GO:0003824] catalytic activity | [GO:0016740] transferase activity | [GO:0016746] transferase activity, transferring acyl groups | ref|NP\_180232 | 3-ketoacyl-CoA synthase 11 [Arabidopsis thaliana] | RFLP | mapped |  |
| PtIFG\_2145\_28 | gb|H75056 | Pinus taeda | 2145s | Pta.2729 | [GO:0008415] acyltransferase activity | molecular\_function | [GO:0003824] catalytic activity | [GO:0016740] transferase activity | [GO:0016746] transferase activity, transferring acyl groups | ref|NP\_180232 | 3-ketoacyl-CoA synthase 11 [Arabidopsis thaliana] | RFLP | mapped |  |
| PtIFG\_2145\_3 | gb|H75056 | Pinus taeda | 2145s | Pta.2729 | [GO:0008415] acyltransferase activity | molecular\_function | [GO:0003824] catalytic activity | [GO:0016740] transferase activity | [GO:0016746] transferase activity, transferring acyl groups | ref|NP\_180232 | 3-ketoacyl-CoA synthase 11 [Arabidopsis thaliana] | RFLP | mapped |  |
| PtIFG\_2145\_76 | gb|H75056 | Pinus taeda | 2145s | Pta.2729 | [GO:0008415] acyltransferase activity | molecular\_function | [GO:0003824] catalytic activity | [GO:0016740] transferase activity | [GO:0016746] transferase activity, transferring acyl groups | ref|NP\_180232 | 3-ketoacyl-CoA synthase 11 [Arabidopsis thaliana] | RFLP | mapped |  |
| PtIFG\_2146\_2 | n/a | Pinus taeda | n/a | n/a | [GO:0003674] molecular\_function | molecular\_function | [Lineage ends at prior level] | [Lineage ends at prior level] | [Lineage ends at prior level] | n/a | n/a | RFLP | mapped | No sequence available. |
| PtIFG\_2150\_A | gb|H75058 | Pinus taeda | 2150e | n/a | [GO:0003674] molecular\_function | molecular\_function | [Lineage ends at prior level] | [Lineage ends at prior level] | [Lineage ends at prior level] | n/a | n/a | RFLP | mapped |  |
| PtIFG\_2197\_1 | n/a | Pinus taeda | n/a | n/a | [GO:0003674] molecular\_function | molecular\_function | [Lineage ends at prior level] | [Lineage ends at prior level] | [Lineage ends at prior level] | n/a | n/a | RFLP | mapped | No sequence available. |
| PtIFG\_2220\_A | n/a | Pinus taeda | n/a | Pta.19510 | [GO:0003723] RNA binding | molecular\_function | [GO:0005488] binding | [GO:0003676] nucleic acid binding | [GO:0003723] RNA binding | ref|NP\_001045195 | glycine-rich RNA binding protein [Oryza sativa] | RFLP | mapped | UniGene assignment is based on blastn similarity of available IFG sequence to Pinus taeda EST gb|DT626617; Score = 669 bits (362), Expect = 0.0 Identities = 481/560 (85%), Gaps = 2/560 (0%). |
| PtIFG\_2220\_B | n/a | Pinus taeda | n/a | Pta.19510 | [GO:0003723] RNA binding | molecular\_function | [GO:0005488] binding | [GO:0003676] nucleic acid binding | [GO:0003723] RNA binding | ref|NP\_001045195 | glycine-rich RNA binding protein [Oryza sativa] | RFLP | mapped | UniGene assignment is based on blastn similarity of available IFG sequence to Pinus taeda EST gb|DT626617; Score = 669 bits (362), Expect = 0.0 Identities = 481/560 (85%), Gaps = 2/560 (0%). |
| PtIFG\_2253\_A | gb|H75063 | Pinus taeda | 2253s | Pta.7831 | [GO:0004332] fructose-bisphosphate aldolase activity | molecular\_function | [GO:0003824] catalytic activity | [GO:0016829] lyase activity | [GO:0016830] carbon-carbon lyase activity | ref|NP\_001041857 | fructose-bisphosphate aldolase [Oryza sativa] | RFLP | mapped |  |
| PtIFG\_2291\_A | gb|H75068 | Pinus taeda | 2291e | Pta.11549 | [GO:0008483] transaminase activity | molecular\_function | [GO:0003824] catalytic activity | [GO:0016740] transferase activity | [GO:0016769] transferase activity, transferring nitrogenous groups | ref|NP\_001049852 | aminotransferase, class I and II domain [Oryza sativa] | RFLP | mapped |  |
| PtIFG\_2295\_2 | gb|H75069 | Pinus taeda | 2295s | Pta.590 | [GO:0030337] DNA polymerase processivity activity | molecular\_function | [GO:0030234] enzyme regulator activity | [GO:0030337] DNA polymerase processivity factor activity | [Lineage ends at prior level] | ref|NP\_172217 | proliferating cellular nuclear antigen [Arabidopsis thaliana] | RFLP | mapped |  |
| PtIFG\_2323\_A | gb|H75072 | Pinus taeda | 2323e | Pta.11550 | [GO:0003674] molecular\_function | molecular\_function | [Lineage ends at prior level] | [Lineage ends at prior level] | [Lineage ends at prior level] | n/a | n/a | RFLP | mapped |  |
| PtIFG\_2361\_2 | gb|H75090 | Pinus taeda | 2361s | Pta.1807 | [GO:0016168] chlorophyll binding | molecular\_function | [GO:0005488] binding | [GO:0046906] tetrapyrrole binding | [GO:0016168] chlorophyll binding | ref|NP\_176347 | chlorophyll a/b binding protein, [Arabidopsis thaliana] | RFLP | mapped |  |
| PtIFG\_2393\_1 | gb|H75095 | Pinus taeda | 2393s | Pta.18383 | [GO:0003674] molecular\_function | molecular\_function | [Lineage ends at prior level] | [Lineage ends at prior level] | [Lineage ends at prior level] | ref|NP\_001060847 | unknown protein [Oryza sativa] | RFLP | mapped | UniGene assignment is based on blastn similarity to the homologous Pinus taeda EST gb|CO172965; Score = 158 bits (85), Expect = 5e-38, Identities = 104/114 (91%), Gaps = 6/114 (5%) |
| PtIFG\_2413\_b | n/a | Pinus taeda | n/a | n/a | [GO:0003674] molecular\_function | molecular\_function | [Lineage ends at prior level] | [Lineage ends at prior level] | [Lineage ends at prior level] | n/a | n/a | RFLP | mapped | No sequence available. |
| PtIFG\_2441\_1 | n/a | Pinus taeda | n/a | Pta.18257 | [GO:0003674] molecular\_function | molecular\_function | [Lineage ends at prior level] | [Lineage ends at prior level] | [Lineage ends at prior level] | ref|NP\_197697 | unknown protein [Arabidopsis thaliana] | RFLP | mapped | UniGene assignment is based on blastn similarity to the homologous Pinus taeda EST gb|DR017785; Score = 1007 bits (545), Expect = 0.0, Identities = 616/664 (92%), Gaps = 10/664 (1%). |
| PtIFG\_2479\_1 | n/a | Pinus taeda | n/a | n/a | [GO:0003674] molecular\_function | molecular\_function | [Lineage ends at prior level] | [Lineage ends at prior level] | [Lineage ends at prior level] | n/a | n/a | RFLP | mapped | No sequence available. |
| PtIFG\_2530\_A | gb|H75202 | Pinus taeda | 2530e | Pta.11556 | [GO:0004089] carbonate dehydratase activity | molecular\_function | [GO:0003824] catalytic activity | [GO:0016829] lyase activity | [GO:0016835] carbon-oxygen lyase activity | ref|NP\_974782 | carbonic anhydrase 2 [Arabidopsis thaliana] | RFLP | mapped |  |
| PtIFG\_2538\_5 | gb|H75205 | Pinus taeda | 2538s | Pta.598 | [GO:0008943] glyceraldehyde-3-phosphate dehydrogenase activity | molecular\_function | [GO:0003824] catalytic activity | [GO:0016491] oxidoreductase activity | [GO:0016903] oxidoreductase activity, acting on the aldehyde or oxo group of donors | ref|NP\_172750 | glyceraldehyde-3-phosphate dehydrogenase [Arabidopsis thaliana] | RFLP | mapped | UniGene assignment is based on blastn similarity to the homologous Pinus taeda EST gb|DR101900; Score = 174 bits (94), Expect = 9e-43, Identities = 139/159 (87%), Gaps = 16/159 (10%). |
| PtIFG\_2538\_B | gb|H75204 | Pinus taeda | 2538e | Pta.598 | [GO:0008943] glyceraldehyde-3-phosphate dehydrogenase activity | molecular\_function | [GO:0003824] catalytic activity | [GO:0016491] oxidoreductase activity | [GO:0016903] oxidoreductase activity, acting on the aldehyde or oxo group of donors | ref|NP\_172750 | glyceraldehyde-3-phosphate dehydrogenase [Arabidopsis thaliana] | RFLP | mapped |  |
| PtIFG\_2564\_A | gb|H75212 | Pinus taeda | 2564e | Pta.594 | [GO:0016168] chlorophyll binding | molecular\_function | [GO:0005488] binding | [GO:0046906] tetrapyrrole binding | [GO:0016168] chlorophyll binding | ref|NP\_001059975 | chlorophyll a/b binding protein, LHCP type II [Oryza sativa] | RFLP | mapped |  |
| PtIFG\_2564\_B | gb|H75213 | Pinus taeda | 2564e | Pta.594 | [GO:0016168] chlorophyll binding | molecular\_function | [GO:0005488] binding | [GO:0046906] tetrapyrrole binding | [GO:0016168] chlorophyll binding | ref|NP\_001059975 | chlorophyll a/b binding protein, LHCP type II [Oryza sativa] | RFLP | mapped |  |
| PtIFG\_2568\_A | gb|H75215 | Pinus taeda | 2568s | Pta.10460 | [GO:0003735] structural constituent of ribosome | molecular\_function | [GO:0005198] structural molecule activity | [GO:0003735] structural constituent of ribosome | [Lineage ends at prior level] | ref|NP\_001049835 | 40S ribosomal protein S7 [Oryza sativa] | RFLP | mapped |  |
| PtIFG\_2574\_2 | gb|H75216 | Pinus taeda | 2574e | Pta.575 | [GO:0016168] chlorophyll binding | molecular\_function | [GO:0005488] binding | [GO:0046906] tetrapyrrole binding | [GO:0016168] chlorophyll binding | ref|NP\_565786 | chlorophyll a/b binding protein, photosystem II [Arabidopsis thaliana] | RFLP | mapped |  |
| PtIFG\_2574\_c | gb|H75217 | Pinus taeda | 2574s | Pta.575 | [GO:0016168] chlorophyll binding | molecular\_function | [GO:0005488] binding | [GO:0046906] tetrapyrrole binding | [GO:0016168] chlorophyll binding | ref|NP\_565786 | chlorophyll a/b binding protein, photosystem II [Arabidopsis thaliana] | RFLP | mapped |  |
| PtIFG\_2588\_1 | gb|H75218 | Pinus taeda | 2588e | Pta.11556 | [GO:0004089] carbonate dehydratase activity | molecular\_function | [GO:0003824] catalytic activity | [GO:0016829] lyase activity | [GO:0016835] carbon-oxygen lyase activity | ref|NP\_974782 | carbonic anhydrase 2 [Arabidopsis thaliana] | RFLP | mapped |  |
| PtIFG\_2615\_1 | gb|H75223 | Pinus taeda | 2615s | n/a | [GO:0003674] molecular\_function | molecular\_function | [Lineage ends at prior level] | [Lineage ends at prior level] | [Lineage ends at prior level] | n/a | n/a | RFLP | mapped |  |
| PtIFG\_2697\_A | gb|H75226 | Pinus taeda | 2697e | Pta.7257 | [GO:0008026] ATP-dependent helicase activity | molecular\_function | [GO:0003824] catalytic activity | [GO:0016787] hydrolase activity | [GO:0016817] hydrolase activity, acting on acid anhydrides | ref|NP\_199941 | DEAD-box protein RNA helicase 35, putative [Arabidopsis thaliana] | RFLP | mapped |  |
| PtIFG\_2718\_1 | gb|H75230 | Pinus taeda | 2718e | Pta.4966 | [GO:0003735] structural constituent of ribosome | molecular\_function | [GO:0005198] structural molecule activity | [GO:0003735] structural constituent of ribosome | [Lineage ends at prior level] | ref|NP\_175009 | ribosomal protein 1, Arabidopsis, 60S ribosomal protein L3-1, [Arabidopsis thaliana] | RFLP | mapped | UniGene assignment is based on blastn similarity to the homologous Pinus taeda EST gb|DT625372; Score = 237 bits (128), Expect = 1e-61, Identities = 185/208 (88%), Gaps = 22/208 (10%). |
| PtIFG\_2718\_2 | gb|H75230 | Pinus taeda | 2718e | Pta.4966 | [GO:0003735] structural constituent of ribosome | molecular\_function | [GO:0005198] structural molecule activity | [GO:0003735] structural constituent of ribosome | [Lineage ends at prior level] | ref|NP\_175009 | ribosomal protein 1, 60S ribosomal protein L3-1, [Arabidopsis thaliana] | RFLP | mapped | UniGene assignment is based on blastn similarity to the homologous Pinus taeda EST gb|DT625372; Score = 237 bits (128), Expect = 1e-61, Identities = 185/208 (88%), Gaps = 22/208 (10%). |
| PtIFG\_2718\_3 | gb|H75230 | Pinus taeda | 2718e | Pta.4966 | [GO:0003735] structural constituent of ribosome | molecular\_function | [GO:0005198] structural molecule activity | [GO:0003735] structural constituent of ribosome | [Lineage ends at prior level] | ref|NP\_175009 | ribosomal protein 1, Arabidopsis, 60S ribosomal protein L3-1, [Arabidopsis thaliana] | RFLP | mapped | UniGene assignment is based on blastn similarity to the homologous Pinus taeda EST gb|DT625372; Score = 237 bits (128), Expect = 1e-61, Identities = 185/208 (88%), Gaps = 22/208 (10%). |
| PtIFG\_2723\_1 | n/a | Pinus taeda | n/a | n/a | [GO:0003674] molecular\_function | molecular\_function | [Lineage ends at prior level] | [Lineage ends at prior level] | [Lineage ends at prior level] | n/a | n/a | RFLP | mapped | No sequence available. |
| PtIFG\_2723\_Aa | n/a | Pinus taeda | n/a | n/a | [GO:0003674] molecular\_function | molecular\_function | [Lineage ends at prior level] | [Lineage ends at prior level] | [Lineage ends at prior level] | n/a | n/a | RFLP | mapped | No sequence available. |
| PtIFG\_2738\_B | n/a | Pinus taeda | n/a | n/a | [GO:0003674] molecular\_function | molecular\_function | [Lineage ends at prior level] | [Lineage ends at prior level] | [Lineage ends at prior level] | n/a | n/a | RFLP | mapped | No sequence available. |
| PtIFG\_2745\_1 | n/a | Pinus taeda | n/a | n/a | [GO:0003674] molecular\_function | molecular\_function | [Lineage ends at prior level] | [Lineage ends at prior level] | [Lineage ends at prior level] | n/a | n/a | RFLP | mapped | No sequence available. |
| PtIFG\_2782\_2 | n/a | Pinus taeda | n/a | n/a | [GO:0003674] molecular\_function | molecular\_function | [Lineage ends at prior level] | [Lineage ends at prior level] | [Lineage ends at prior level] | n/a | n/a | RFLP | mapped | No sequence available. |
| PtIFG\_2782\_31 | n/a | Pinus taeda | n/a | n/a | [GO:0003674] molecular\_function | molecular\_function | [Lineage ends at prior level] | [Lineage ends at prior level] | [Lineage ends at prior level] | n/a | n/a | RFLP | mapped | No sequence available. |
| PtIFG\_2802\_3 | n/a | Pinus taeda | n/a | n/a | [GO:0003674] molecular\_function | molecular\_function | [Lineage ends at prior level] | [Lineage ends at prior level] | [Lineage ends at prior level] | n/a | n/a | RFLP | mapped | No sequence available. |
| PtIFG\_2885\_1 | n/a | Pinus taeda | n/a | n/a | [GO:0003674] molecular\_function | molecular\_function | [Lineage ends at prior level] | [Lineage ends at prior level] | [Lineage ends at prior level] | n/a | n/a | RFLP | mapped | No sequence available. |
| PtIFG\_2897\_d | gb|H75242 | Pinus taeda | 2897e | Pta.11468 | [GO:0003674] molecular\_function | molecular\_function | [Lineage ends at prior level] | [Lineage ends at prior level] | [Lineage ends at prior level] | n/a | n/a | RFLP | mapped |  |
| PtIFG\_2899\_A | gb|H75245 | Pinus taeda | 2899s | Pta.4670 | [GO:0004842] ubiquitin-protein ligase activity | molecular\_function | [GO:0003824] catalytic activity | [GO:0016874] ligase activity | [GO:0016879] ligase activity, forming carbon-nitrogen bonds | ref|NP\_001046486 | ubiquitin conjugating enzyme [Oryza sativa] | RFLP | mapped |  |
| PtIFG\_2931\_A | gb|H75250 | Pinus taeda | 2931s | n/a | [GO:0009976] tocopherol cyclase activity | molecular\_function | [GO:0003824] catalytic activity | [GO:0009975] cyclase activity | [GO:0009976] tocopherol cyclase activity | ref|NP\_567906 | tocopherol cyclase, chloroplast precursor [Arabidopsis thaliana] | RFLP | mapped | RefSeq protein assignment is based on blastx similarity to the GenBank seq.; Score = 84.7 bits (208), Expect = 1e-15, Identities = 42/75 (56%), Positives = 49/75 (65%), Gaps = 1/75 (1%). |
| PtIFG\_2933\_12 | n/a | Pinus taeda | n/a | n/a | [GO:0003674] molecular\_function | molecular\_function | [Lineage ends at prior level] | [Lineage ends at prior level] | [Lineage ends at prior level] | n/a | n/a | RFLP | mapped | No sequence available. |
| PtIFG\_2957\_A | n/a | Pinus taeda | n/a | n/a | [GO:0003674] molecular\_function | molecular\_function | [Lineage ends at prior level] | [Lineage ends at prior level] | [Lineage ends at prior level] | n/a | n/a | RFLP | mapped | No sequence available. |
| PtIFG\_2963\_1 | n/a | Pinus taeda | n/a | n/a | [GO:0003674] molecular\_function | molecular\_function | [Lineage ends at prior level] | [Lineage ends at prior level] | [Lineage ends at prior level] | n/a | n/a | RFLP | mapped | No sequence available. |
| PtIFG\_2963\_3 | n/a | Pinus taeda | n/a | n/a | [GO:0003674] molecular\_function | molecular\_function | [Lineage ends at prior level] | [Lineage ends at prior level] | [Lineage ends at prior level] | n/a | n/a | RFLP | mapped | No sequence available. |
| PtIFG\_2969\_1 | n/a | Pinus taeda | n/a | n/a | [GO:0003674] molecular\_function | molecular\_function | [Lineage ends at prior level] | [Lineage ends at prior level] | [Lineage ends at prior level] | n/a | n/a | RFLP | mapped | No sequence available. |
| PtIFG\_2986\_A | n/a | Pinus taeda | n/a | n/a | [GO:0003674] molecular\_function | molecular\_function | [Lineage ends at prior level] | [Lineage ends at prior level] | [Lineage ends at prior level] | n/a | n/a | RFLP | mapped | No sequence available. |
| PtIFG\_2986\_B | n/a | Pinus taeda | n/a | n/a | [GO:0003674] molecular\_function | molecular\_function | [Lineage ends at prior level] | [Lineage ends at prior level] | [Lineage ends at prior level] | n/a | n/a | RFLP | mapped | No sequence available. |
| PtIFG\_2988\_21 | n/a | Pinus taeda | n/a | n/a | [GO:0003674] molecular\_function | molecular\_function | [Lineage ends at prior level] | [Lineage ends at prior level] | [Lineage ends at prior level] | n/a | n/a | RFLP | mapped | No sequence available. |
| PtIFG\_3006\_1 | n/a | Pinus taeda | n/a | n/a | [GO:0003674] molecular\_function | molecular\_function | [Lineage ends at prior level] | [Lineage ends at prior level] | [Lineage ends at prior level] | n/a | n/a | RFLP | mapped | No sequence available. |
| PtIFG\_3008\_1 | gb|H75259 | Pinus taeda | 3008s | Pta.1795 | [GO:0016020] membrane | cellular\_component | [GO:0005623] cell | [GO:0044464] cell part | [GO:00160201] membrane | ref|NP\_001049916 | CAP protein family protein [Oryza sativa] | RFLP | mapped |  |
| PtIFG\_3012\_2 | gb|H75261 | Pinus taeda | 3012e | Pta.1533 | [GO:0016630] protochlorophyllide reductase activity | molecular\_function | [GO:0003824] catalytic activity | [GO:0016491] oxidoreductase activity | [GO:0016627] oxidoreductase activity, acting on the CH-CH group of donors | ref|NP\_200230 | protochlorophyllide reductase A [Arabidopsis thaliana] | RFLP | mapped |  |
| PtIFG\_3012\_3 | gb|H75262 | Pinus taeda | 3012s | Pta.1533 | [GO:0016630] protochlorophyllide reductase activity | molecular\_function | [GO:0003824] catalytic activity | [GO:0016491] oxidoreductase activity | [GO:0016627] oxidoreductase activity, acting on the CH-CH group of donors | ref|NP\_200230 | protochlorophyllide reductase A [Arabidopsis thaliana] | RFLP | mapped |  |
| PtIFG\_3021\_1 | gb|H75263 | Pinus taeda | 3021e | Pta.2130 | [GO:0005524] ATP binding | molecular\_function | [GO:0005488] binding | [GO:0000166] nucleotide binding | [GO:0017076] purine nucleotide binding | ref|NP\_001058590 | Endoplasmin homolog precursor (GRP94 homolog) [Oryza sativa] | RFLP | not mapped |  |
| PtIFG\_3026\_A | n/a | Pinus taeda | n/a | n/a | [GO:0003674] molecular\_function | molecular\_function | [Lineage ends at prior level] | [Lineage ends at prior level] | [Lineage ends at prior level] | n/a | n/a | RFLP | mapped | No sequence available. |
| PtIFG\_459\_1 | n/a | Pinus taeda | n/a | n/a | [GO:0003674] molecular\_function | molecular\_function | [Lineage ends at prior level] | [Lineage ends at prior level] | [Lineage ends at prior level] | n/a | n/a | RFLP | mapped | No sequence available. |
| PtIFG\_4D4\_A | n/a | Pinus taeda | n/a | n/a | [GO:0003674] molecular\_function | molecular\_function | [Lineage ends at prior level] | [Lineage ends at prior level] | [Lineage ends at prior level] | n/a | n/a | RFLP | mapped | No sequence available. |
| PtIFG\_503\_A | gb|H75154 | Pinus taeda | 0503s | Pta.586 | [GO:0009793] embryonic development ending in seed dormancy | biological\_process | [GO:0032502] developmental process | [GO:0009790] embryonic development | [GO:0009793] embryonic development ending in seed dormancy | ref|NP\_567213 | maternal effect embryo arrest 49 [Arabidopsis thaliana] | RFLP | mapped |  |
| PtIFG\_606\_1 | n/a | Pinus taeda | n/a | Pta.5116 | [GO:0008270] zinc ion binding | molecular\_function | [GO:0005488] binding | [GO:0043167] ion binding | [GO:0043169] cation binding | ref|NP\_001064398 | zinc-finger, RING domain containing protein [Oryza sativa] | RFLP | mapped | UniGene assignment is based on blastn similarity to the homologous Pinus taeda EST gb|CO174714; Score = 730 bits (395), Expect = 0.0, Identities = 427/445 (95%), Gaps = 6/445 (1%). |
| PtIFG\_616 | gb|H75116 | Pinus taeda | 0616J | Pta.11262 | [GO:0003674] molecular\_function | molecular\_function | [Lineage ends at prior level] | [Lineage ends at prior level] | [Lineage ends at prior level] | n/a | n/a | RFLP | mapped |  |
| PtIFG\_653\_2 | gb|H75097 | Pinus taeda | 0653T | Pta.1755 | [GO:0046873] metal ion transporter activity | molecular\_function | [GO:0005215] transporter activity | [GO:0022857] transmembrane transporter activity | [GO:0022891] substrate-specific transmembrane transporter activity | ref|NP\_179896 | natural resistance-associated macrophage protein 4 [Arabidopsis thaliana] | RFLP | mapped |  |
| PtIFG\_653\_3 | gb|H75097 | Pinus taeda | 0653T | Pta.1755 | [GO:0046873] metal ion transporter activity | molecular\_function | [GO:0005215] transporter activity | [GO:0022857] transmembrane transporter activity | [GO:0022891] substrate-specific transmembrane transporter activity | ref|NP\_179896 | natural resistance-associated macrophage protein 4 [Arabidopsis thaliana] | RFLP | mapped |  |
| PtIFG\_653\_d | gb|H75097 | Pinus taeda | 0653T | Pta.1755 | [GO:0046873] metal ion transporter activity | molecular\_function | [GO:0005215] transporter activity | [GO:0022857] transmembrane transporter activity | [GO:0022891] substrate-specific transmembrane transporter activity | ref|NP\_179896 | natural resistance-associated macrophage protein 4 [Arabidopsis thaliana] | RFLP | mapped |  |
| PtIFG\_658\_A | n/a | Pinus taeda | n/a | n/a | [GO:0003674] molecular\_function | molecular\_function | [Lineage ends at prior level] | [Lineage ends at prior level] | [Lineage ends at prior level] | n/a | n/a | RFLP | mapped | No sequence available. |
| PtIFG\_66\_1 | gb|H75018 | Pinus taeda | 0066e | Pta.11533 | [GO:0003674] molecular\_function | molecular\_function | [Lineage ends at prior level] | [Lineage ends at prior level] | [Lineage ends at prior level] | n/a | n/a | RFLP | mapped |  |
| PtIFG\_669 | gb|H75159 | Pinus taeda | 0669s | Pta.11540 | [GO:0016168] chlorophyll binding | molecular\_function | [GO:0005488] binding | [GO:0046906] tetrapyrrole binding | [GO:0016168] chlorophyll binding | ref|NP\_175092 | chlorophyll a/b binding protein, LHCP type II [Arabidopsis thaliana] | RFLP | mapped |  |
| PtIFG\_719\_3 | n/a | Pinus taeda | n/a | n/a | [GO:0003674] molecular\_function | molecular\_function | [Lineage ends at prior level] | [Lineage ends at prior level] | [Lineage ends at prior level] | n/a | n/a | RFLP | mapped | No sequence available. |
| PtIFG\_719\_A | n/a | Pinus taeda | n/a | n/a | [GO:0003674] molecular\_function | molecular\_function | [Lineage ends at prior level] | [Lineage ends at prior level] | [Lineage ends at prior level] | n/a | n/a | RFLP | mapped | No sequence available. |
| PtIFG\_846 | gb|H75117 | Pinus taeda | 0846J | Pta.5444 | [GO:0008453] alanine-glyoxylate transaminase activity | molecular\_function | [GO:0003824] catalytic activity | [GO:0016740] transferase activity | [GO:0016769] transferase activity, transferring nitrogenous groups | ref|NP\_001049115 | alanine-glyoxylate aminotransferase-like protein [Oryza sativa] | RFLP | mapped |  |
| PtIFG\_851\_1 | n/a | Pinus taeda | n/a | n/a | [GO:0003674] molecular\_function | molecular\_function | [Lineage ends at prior level] | [Lineage ends at prior level] | [Lineage ends at prior level] | n/a | n/a | RFLP | mapped | No sequence available. |
| PtIFG\_975\_3 | gb|H75119 | Pinus taeda | 0975J | Pta.9520 | [GO:0003674] molecular\_function | molecular\_function | [Lineage ends at prior level] | [Lineage ends at prior level] | [Lineage ends at prior level] | ref|NP\_001048111 | unknown protein [Oryza sativa] | RFLP | mapped |  |
| PtIFG\_975\_4 | gb|H75119 | Pinus taeda | 0975J | Pta.9520 | [GO:0003674] molecular\_function | molecular\_function | [Lineage ends at prior level] | [Lineage ends at prior level] | [Lineage ends at prior level] | ref|NP\_001048111 | unknown protein [Oryza sativa] | RFLP | mapped |  |
| estPtIFG\_dhn-1 | gb|BX255067 | Pinus pinaster | PP110C05 | n/a | [GO:0003779] actin binding | molecular\_function | [GO:0005488] binding | [GO:0005515] protein binding | [GO:0008092] cytoskeletal protein binding | n/a | n/a | ESTP | mapped | UniGene assignment is based on the homologous Pinus taeda EST gb|DR068451. |
| PtIPST\_pLP2 | n/a | Pinus taeda | n/a | n/a | [GO:0003674] molecular\_function | molecular\_function | [Lineage ends at prior level] | [Lineage ends at prior level] | [Lineage ends at prior level] | n/a | n/a | ESTP | mapped | No sequence available. |
| PtLP15 | gb|AF013803 | Pinus taeda | n/a | Pta.178 | [GO:0003677] DNA binding | molecular\_function | [GO:0005488] binding | [GO:0003676] nucleic acid binding | [GO:0003677] DNA binding | ref|NP\_175517 | histone H2A-1, putative [Arabidopsis thaliana] | ESTP | mapped |  |
| PtLP3-1 | gb|U67135 | Pinus taeda | n/a | Pta.447 | [GO:0006950] response to stress | biological\_process | [GO:0050896] response to stimulus | [GO:0006950] response to stress | [Lineage ends at prior level] | ref|NP\_001065841 | pollen-specific desiccation-associated LLA23 protein [Oryza sativa] | ESTP | mapped |  |
| PtMTU\_lpPAL | gb|U39792 | Pinus taeda | n/a | Pta.2030 | [GO:0045548] phenylalanine ammonia-lyase activity | molecular\_function | [GO:0003824] catalytic activity | [GO:0016829] lyase activity | [GO:0016840] carbon-nitrogen lyase activity | ref|NP\_190894 | phenylalanine ammonia-lyase [Arabidopsis thaliana] | ESTP | mapped | UniGene assignment is based blastn similarity to the Pinus taeda EST gb|DR093041; Score = 1604 bits (868), Expect = 0.0, Identities = 888/897 (98%), Gaps = 3/897 (0%) |
| PtNCS\_1CA4G | gb|AA556223 | Pinus taeda | 1CA4G | n/a | [GO:0004799] thymidylate synthase activity | molecular\_function | [GO:0003824] catalytic activity | [GO:0016740] transferase activity | [GO:0016741] transferase activity, transferring one-carbon groups | ref|NP\_179230 | thymidylate synthase1 [Arabidopsis thaliana] | ESTP | mapped | RefSeq protein assignment is based on blastx similarity to the GenBank seq.; Score = 64.7 bits (156), Expect = 4e-09, Identities = 28/33 (84%), Positives = 31/33 (93%), Gaps = 0/33 (0%). |
| PtNCS\_3H6z5\_A | n/a | Pinus taeda | n/a | n/a | [GO:0003674] molecular\_function | molecular\_function | [Lineage ends at prior level] | [Lineage ends at prior level] | [Lineage ends at prior level] | n/a | n/a | RFLP | mapped | No sequence available. |
| PtNCS\_HLH1 | gb|AF103808 | Pinus taeda | n/a | Pta.13198 | [GO:0003674] molecular\_function | molecular\_function | [Lineage ends at prior level] | [Lineage ends at prior level] | [Lineage ends at prior level] | n/a | n/a | ESTP | mapped | UniGene assignment is based on blastn similarity to the homologous Pinus taeda EST gb|BE761812; Score = 651 bits (352), Expect = 0.0, Identities = 377/395 (95%), Gaps = 3/395 (0%). |
| PtNCS\_p9myb1\_21 | gb|AL750878 | Pinus pinaster | RS01D05 | Pta.7264 | [GO:0003700] transcription factor activity | molecular\_function | [GO:0005488] binding | [GO:0003676] nucleic acid binding | [GO:0003677] DNA binding | ref|NP\_201531 | myb domain protein 44, myb domain protein r1 [Arabidopsis thaliana] | RFLP | mapped | UniGene assignment is based on blastn similarity to the homologous Pinus taeda EST gb|DT626587; Score = 802 bits (434), Expect = 0.0, Identities = 453/462 (98%), Gaps = 2/462 (0%). |
| PtNCS\_PtaAGP6 | gb|AF101785 | Pinus taeda | PtaAGP6 | Pta.11525 | [GO:0003674] molecular\_function | molecular\_function | [Lineage ends at prior level] | [Lineage ends at prior level] | [Lineage ends at prior level] | ref|NP\_001041796 | conserved hypothetical protein [Oryza sativa] | ESTP | mapped |  |
| PtNCS\_ptCadA | gb|Z37991 | Pinus taeda | ptCadA | Pta.428 | [GO:0045551] cinnamyl-alcohol dehydrogenase activity | molecular\_function | [GO:0003824] catalytic activity | [GO:0016491] oxidoreductase activity | [GO:0016614] oxidoreductase activity, acting on CH-OH group of donors | ref|NP\_195149 | cinnamyl alcohol dehydrogenase 5 [Arabidopsis thaliana] | ESTP | mapped |  |
| PtRIP\_0022 | gb|BV683041 | Pinus taeda | pPT22 | n/a | n/a | n/a | n/a | n/a | n/a | n/a | n/a | SSR | mapped |  |
| PtRIP\_0032 | gb|BV683044 | Pinus taeda | pPT32 | n/a | n/a | n/a | n/a | n/a | n/a | n/a | n/a | SSR | mapped |  |
| PtRIP\_0064 | gb|BV683046 | Pinus taeda | pPT64 | n/a | n/a | n/a | n/a | n/a | n/a | n/a | n/a | SSR | mapped |  |
| PtRIP\_0065 | gb|BV683047 | Pinus taeda | pPT65 | n/a | n/a | n/a | n/a | n/a | n/a | n/a | n/a | SSR | mapped |  |
| PtRIP\_0066 | gb|BV683048 | Pinus taeda | pPT66 | n/a | n/a | n/a | n/a | n/a | n/a | n/a | n/a | SSR | mapped |  |
| PtRIP\_0067 | gb|BV683049 | Pinus taeda | pPT67 | n/a | n/a | n/a | n/a | n/a | n/a | n/a | n/a | SSR | mapped |  |
| PtRIP\_0079 | gb|BV683053 | Pinus taeda | pPT79 | n/a | n/a | n/a | n/a | n/a | n/a | n/a | n/a | SSR | mapped |  |
| PtRIP\_0103 | gb|BV683057 | Pinus taeda | pPT103 | n/a | n/a | n/a | n/a | n/a | n/a | n/a | n/a | SSR | mapped |  |
| PtRIP\_0106 | gb|BV683059 | Pinus taeda | pPT106 | n/a | n/a | n/a | n/a | n/a | n/a | n/a | n/a | SSR | mapped |  |
| PtRIP\_0117 | gb|BV683060 | Pinus taeda | pPT117 | n/a | n/a | n/a | n/a | n/a | n/a | n/a | n/a | SSR | mapped |  |
| PtRIP\_0126 | gb|BV683062 | Pinus taeda | pPT126 | n/a | n/a | n/a | n/a | n/a | n/a | n/a | n/a | SSR | mapped |  |
| PtRIP\_0134 | gb|BV683065 | Pinus taeda | pPT134 | n/a | n/a | n/a | n/a | n/a | n/a | n/a | n/a | SSR | mapped |  |
| PtRIP\_0135 | gb|BV683066 | Pinus taeda | pPT135 | n/a | n/a | n/a | n/a | n/a | n/a | n/a | n/a | SSR | mapped |  |
| PtRIP\_0158 | gb|BV683068 | Pinus taeda | pPT158 | n/a | n/a | n/a | n/a | n/a | n/a | n/a | n/a | SSR | mapped |  |
| PtRIP\_0165 | gb|BV683070 | Pinus taeda | pPT165 | n/a | n/a | n/a | n/a | n/a | n/a | n/a | n/a | SSR | mapped |  |
| PtRIP\_0171 | gb|BV683072 | Pinus taeda | pPT171 | n/a | n/a | n/a | n/a | n/a | n/a | n/a | n/a | SSR | mapped |  |
| PtRIP\_0179 | gb|BV683073 | Pinus taeda | pPT179 | n/a | n/a | n/a | n/a | n/a | n/a | n/a | n/a | SSR | mapped |  |
| PtRIP\_0211 | gb|BV683076 | Pinus taeda | pPT211 | n/a | n/a | n/a | n/a | n/a | n/a | n/a | n/a | SSR | mapped |  |
| PtRIP\_0255 | gb|BV683147 | Pinus taeda | pPT255 | n/a | n/a | n/a | n/a | n/a | n/a | n/a | n/a | SSR | mapped |  |
| PtRIP\_0263 | gb|BV683148 | Pinus taeda | pPT263 | n/a | n/a | n/a | n/a | n/a | n/a | n/a | n/a | SSR | mapped |  |
| PtRIP\_0287 | gb|BV683077 | Pinus taeda | pPT287 | n/a | n/a | n/a | n/a | n/a | n/a | n/a | n/a | SSR | mapped |  |
| PtRIP\_0305 | gb|BV683080 | Pinus taeda | pPT305 | n/a | n/a | n/a | n/a | n/a | n/a | n/a | n/a | SSR | mapped |  |
| PtRIP\_0367 | gb|BV683081 | Pinus taeda | pPT367 | n/a | n/a | n/a | n/a | n/a | n/a | n/a | n/a | SSR | mapped |  |
| PtRIP\_0376 | gb|BV683083 | Pinus taeda | pPT376 | n/a | n/a | n/a | n/a | n/a | n/a | n/a | n/a | SSR | mapped |  |
| PtRIP\_0388 | gb|BV683084 | Pinus taeda | pPT388 | n/a | n/a | n/a | n/a | n/a | n/a | n/a | n/a | SSR | mapped |  |
| PtRIP\_0496 | gb|BV683151 | Pinus taeda | pPT496 | n/a | n/a | n/a | n/a | n/a | n/a | n/a | n/a | SSR | mapped |  |
| PtRIP\_0508 | gb|BV683085 | Pinus taeda | pPT508 | n/a | n/a | n/a | n/a | n/a | n/a | n/a | n/a | SSR | mapped |  |
| PtRIP\_0540 | gb|BV683152 | Pinus taeda | pPT540 | n/a | n/a | n/a | n/a | n/a | n/a | n/a | n/a | SSR | mapped |  |
| PtRIP\_0560 | gb|BV683088 | Pinus taeda | pPT560 | n/a | n/a | n/a | n/a | n/a | n/a | n/a | n/a | SSR | mapped |  |
| PtRIP\_0567 | gb|BV683089 | Pinus taeda | pPT567 | n/a | n/a | n/a | n/a | n/a | n/a | n/a | n/a | SSR | mapped |  |
| PtRIP\_0609 | gb|BV683090 | Pinus taeda | pPT609 | n/a | n/a | n/a | n/a | n/a | n/a | n/a | n/a | SSR | mapped |  |
| PtRIP\_0619 | gb|BV683091 | Pinus taeda | pPT619 | n/a | n/a | n/a | n/a | n/a | n/a | n/a | n/a | SSR | mapped |  |
| PtRIP\_0621 | gb|BV683092 | Pinus taeda | pPT621 | n/a | n/a | n/a | n/a | n/a | n/a | n/a | n/a | SSR | mapped |  |
| PtRIP\_0627 | gb|BV683093 | Pinus taeda | pPT627 | n/a | n/a | n/a | n/a | n/a | n/a | n/a | n/a | SSR | mapped |  |
| PtRIP\_0630 | gb|BV683095 | Pinus taeda | pPT630 | n/a | n/a | n/a | n/a | n/a | n/a | n/a | n/a | SSR | mapped |  |
| PtRIP\_0647 | gb|BV683097 | Pinus taeda | pPT647 | n/a | n/a | n/a | n/a | n/a | n/a | n/a | n/a | SSR | mapped |  |
| PtRIP\_0649 | gb|BV683098 | Pinus taeda | pPT649 | n/a | n/a | n/a | n/a | n/a | n/a | n/a | n/a | SSR | mapped |  |
| PtRIP\_0658 | gb|BV683099 | Pinus taeda | pPT658 | n/a | n/a | n/a | n/a | n/a | n/a | n/a | n/a | SSR | mapped |  |
| PtRIP\_0675 | gb|BV683100 | Pinus taeda | pPT675 | n/a | n/a | n/a | n/a | n/a | n/a | n/a | n/a | SSR | mapped |  |
| PtRIP\_0683 | gb|BV683101 | Pinus taeda | pPT683 | n/a | n/a | n/a | n/a | n/a | n/a | n/a | n/a | SSR | mapped |  |
| PtRIP\_0689 | gb|BV683103 | Pinus taeda | pPT689 | n/a | n/a | n/a | n/a | n/a | n/a | n/a | n/a | SSR | mapped |  |
| PtRIP\_0700 | gb|BV683106 | Pinus taeda | pPT700 | n/a | n/a | n/a | n/a | n/a | n/a | n/a | n/a | SSR | mapped |  |
| PtRIP\_0789 | gb|BV683108 | Pinus taeda | pPT789 | n/a | n/a | n/a | n/a | n/a | n/a | n/a | n/a | SSR | mapped |  |
| PtRIP\_0790 | gb|BV683109 | Pinus taeda | pPT790 | n/a | n/a | n/a | n/a | n/a | n/a | n/a | n/a | SSR | mapped |  |
| PtRIP\_0791 | gb|BV683110 | Pinus taeda | pPT791 | n/a | n/a | n/a | n/a | n/a | n/a | n/a | n/a | SSR | mapped |  |
| PtRIP\_0814 | gb|BV683112 | Pinus taeda | pPT814 | n/a | n/a | n/a | n/a | n/a | n/a | n/a | n/a | SSR | mapped |  |
| PtRIP\_0841 | gb|BV683113 | Pinus taeda | pPT841 | n/a | n/a | n/a | n/a | n/a | n/a | n/a | n/a | SSR | mapped |  |
| PtRIP\_0846 | gb|BV683114 | Pinus taeda | pPT846 | n/a | n/a | n/a | n/a | n/a | n/a | n/a | n/a | SSR | mapped |  |
| PtRIP\_0852 | gb|BV683115 | Pinus taeda | pPT852 | n/a | n/a | n/a | n/a | n/a | n/a | n/a | n/a | SSR | mapped |  |
| PtRIP\_0860 | gb|BV683116 | Pinus taeda | pPT860 | n/a | n/a | n/a | n/a | n/a | n/a | n/a | n/a | SSR | mapped |  |
| PtRIP\_0905 | gb|BV683117 | Pinus taeda | pPT905 | n/a | n/a | n/a | n/a | n/a | n/a | n/a | n/a | SSR | mapped |  |
| PtRIP\_0932 | gb|BV683119 | Pinus taeda | pPT932 | n/a | n/a | n/a | n/a | n/a | n/a | n/a | n/a | SSR | mapped |  |
| PtRIP\_0941 | gb|BV683120 | Pinus taeda | pPT941 | n/a | n/a | n/a | n/a | n/a | n/a | n/a | n/a | SSR | mapped |  |
| PtRIP\_0958 | gb|BV683122 | Pinus taeda | pPT958 | n/a | n/a | n/a | n/a | n/a | n/a | n/a | n/a | SSR | mapped |  |
| PtRIP\_0960 | gb|BV683123 | Pinus taeda | pPT960 | n/a | n/a | n/a | n/a | n/a | n/a | n/a | n/a | SSR | mapped |  |
| PtRIP\_0968 | gb|BV683124 | Pinus taeda | pPT968 | n/a | n/a | n/a | n/a | n/a | n/a | n/a | n/a | SSR | mapped |  |
| PtRIP\_0984 | gb|BV683125 | Pinus taeda | pPT984 | n/a | n/a | n/a | n/a | n/a | n/a | n/a | n/a | SSR | mapped |  |
| PtRIP\_0990 | gb|BV683126 | Pinus taeda | pPT990 | n/a | n/a | n/a | n/a | n/a | n/a | n/a | n/a | SSR | mapped |  |
| PtRIP\_1023 | gb|BV683128 | Pinus taeda | pPT1023 | n/a | n/a | n/a | n/a | n/a | n/a | n/a | n/a | SSR | mapped |  |
| PtRIP\_1027 | gb|BV683129 | Pinus taeda | pPT1027 | n/a | n/a | n/a | n/a | n/a | n/a | n/a | n/a | SSR | mapped |  |
| PtRIP\_1035 | gb|BV683130 | Pinus taeda | pPT1035 | n/a | n/a | n/a | n/a | n/a | n/a | n/a | n/a | SSR | mapped |  |
| PtRIP\_1036 | gb|BV683131 | Pinus taeda | pPT1036 | n/a | n/a | n/a | n/a | n/a | n/a | n/a | n/a | SSR | mapped |  |
| PtRIP\_1037 | gb|BV683132 | Pinus taeda | pPT1037 | n/a | n/a | n/a | n/a | n/a | n/a | n/a | n/a | SSR | mapped |  |
| PtRIP\_1040 | gb|BV683133 | Pinus taeda | pPT1040 | n/a | n/a | n/a | n/a | n/a | n/a | n/a | n/a | SSR | mapped |  |
| PtRIP\_1072 | gb|BV683135 | Pinus taeda | pPT1072 | n/a | n/a | n/a | n/a | n/a | n/a | n/a | n/a | SSR | mapped |  |
| PtRIP\_1077 | gb|BV683137 | Pinus taeda | pPT1077 | n/a | n/a | n/a | n/a | n/a | n/a | n/a | n/a | SSR | mapped |  |
| PtRIP\_9138 | gb|BV683142 | Pinus taeda | pPT9138 | n/a | n/a | n/a | n/a | n/a | n/a | n/a | n/a | SSR | mapped |  |
| PtRIP\_9315 | gb|BV683144 | Pinus taeda | pPT9315 | n/a | n/a | n/a | n/a | n/a | n/a | n/a | n/a | SSR | mapped |  |
| PtSIFG\_0100 | gb|CF666287 | Pinus taeda | RTCNT1\_22\_E09\_A029 | Pta.1497 | [GO:0004364] glutathione transferase activity | molecular\_function | [GO:0003824] catalytic activity | [GO:0016740] transferase activity | [GO:0016765] transferase activity, transferring alkyl or aryl (other than methyl) groups | ref|NP\_176176 | glutathione S-transferase [Arabidopsis thaliana] | SSR | not mapped |  |
| PtSIFG\_0126 | gb|CF671158 | Pinus taeda | RTCNT1\_55\_D08\_A029 | Pta.10824 | [GO:0016491] oxidoreductase activity | molecular\_function | [GO:0003824] catalytic activity | [GO:0016491] oxidoreductase activity | [Lineage ends at prior level] | ref|NP\_001063656 | FAD dependent oxidoreductase family protein [Oryza sativa] | SSR | mapped |  |
| PtSIFG\_0133 | gb|DR022395 | Pinus taeda | STRS1\_50\_F12\_A034 | Pta.14124 | [GO:0016004] phospholipase activator activity | molecular\_function | [GO:0030234] enzyme regulator activity | [GO:0008047] enzyme activator activity | [GO:0060229] lipase activator activity | ref|NP\_191788 | ADP-ribosylation factor A1E [Arabidopsis thaliana] | SSR | not mapped |  |
| PtSIFG\_0141 | gb|CF472666 | Pinus taeda | RTDS1\_11\_C04\_A015 | n/a | [GO:0003674] molecular\_function | molecular\_function | [Lineage ends at prior level] | [Lineage ends at prior level] | [Lineage ends at prior level] | n/a | n/a | SSR | not mapped |  |
| PtSIFG\_0145 | gb|CF390086 | Pinus taeda | RTDR2\_12\_A01\_A021 | Pta.12620 | [GO:0003674] molecular\_function | molecular\_function | [Lineage ends at prior level] | [Lineage ends at prior level] | [Lineage ends at prior level] | n/a | n/a | SSR | mapped |  |
| PtSIFG\_0166 | gb|CF385811 | Pinus taeda | RTDR1\_6\_F03\_A015 | Pta.12129 | [GO:0003674] molecular\_function | molecular\_function | [Lineage ends at prior level] | [Lineage ends at prior level] | [Lineage ends at prior level] | n/a | n/a | SSR | mapped |  |
| PtSIFG\_0167 | gb|CX712497 | Pinus taeda | RTPQ1\_2\_H04\_A032 | Pta.12061 | [GO:0003674] molecular\_function | molecular\_function | [Lineage ends at prior level] | [Lineage ends at prior level] | [Lineage ends at prior level] | n/a | n/a | SSR | mapped |  |
| PtSIFG\_0168 | gb|DR177748 | Pinus taeda | RTMNUT1\_7\_D09\_A029 | n/a | [GO:0003674] molecular\_function | molecular\_function | [Lineage ends at prior level] | [Lineage ends at prior level] | [Lineage ends at prior level] | n/a | n/a | SSR | mapped |  |
| PtSIFG\_0174 | gb|CO364633 | Pinus taeda | RTK1\_16\_H02\_A029 | Pta.6402 | [GO:0003674] molecular\_function | molecular\_function | [Lineage ends at prior level] | [Lineage ends at prior level] | [Lineage ends at prior level] | n/a | n/a | SSR | mapped |  |
| PtSIFG\_0186 | gb|CF395413 | Pinus taeda | RTDS2\_11\_E03\_A021 | Pta.332 | [GO:0008270] zinc ion binding | molecular\_function | [GO:0005488] binding | [GO:0043167] ion binding | [GO:0043169] cation binding | ref|NP\_001077694 | zinc finger A20 and AN1domain-containing stress-associated protein 2 [Arabidopsis thaliana] | SSR | mapped |  |
| PtSIFG\_0193 | gb|CF394546 | Pinus taeda | RTDS2\_6\_H10\_A021 | Pta.449 | [GO:0003735] structural constituent of ribosome | molecular\_function | [GO:0005198] structural molecule activity | [GO:0003735] structural constituent of ribosome | [Lineage ends at prior level] | ref|NP\_180766 | 40S ribosomal protein S12 [Oryza sativa] | SSR | mapped |  |
| PtSIFG\_0198 | gb|CF477014 | Pinus taeda | RTWW3\_5\_A08\_A022 | Pta.5236 | [GO:0008964] phosphoenolpyruvate carboxylase activity | molecular\_function | [GO:0003824] catalytic activity | [GO:0016829] lyase activity | [GO:0016830] carbon-carbon lyase activity | ref|NP\_175738 | phosphoenolpyruvate carboxylase [Arabdopsis thaliana} | SSR | not mapped |  |
| PtSIFG\_0203 | gb|DR070969 | Pinus taeda | RTDK1\_16\_F04\_A029 | Pta.15282 | [GO:0003674] molecular\_function | molecular\_function | [Lineage ends at prior level] | [Lineage ends at prior level] | [Lineage ends at prior level] | n/a | n/a | SSR | mapped |  |
| PtSIFG\_0206 | gb|DR160521 | Pinus taeda | RTFE1\_6\_B08\_A029 | Pta.2426 | [GO:0006950] response to stress | biological\_process | [GO:0050896] response to stimulus | [GO:0006950] response to stress | [Lineage ends at prior level] | ref|NP\_001065841 | pollen-specific desiccation-associated LLA23 protein [Oryza sativa] | SSR | mapped |  |
| PtSIFG\_0209 | gb|CO158573 | Pinus taeda | FLD1\_7\_F12\_A029 | Pta.2123 | [GO:0003674] molecular\_function | molecular\_function | [Lineage ends at prior level] | [Lineage ends at prior level] | [Lineage ends at prior level] | n/a | n/a | SSR | mapped |  |
| PtSIFG\_0219 | gb|DR097837 | Pinus taeda | STRR1\_37\_H04\_A033 | Pta.18 | [GO:0004601] peroxidase activity | molecular\_function | [GO:0003824] catalytic activity | [GO:0016491] oxidoreductase activity | [GO:0016684] oxidoreductase activity, acting on peroxide as acceptor | ref|NP\_177313.1 | peroxidase 12 [Arabidopsis thaliana] | SSR | mapped |  |
| PtSIFG\_0237 | gb|CO368359 | Pinus taeda | RTK1\_40\_D11\_A029 | Pta.6316 | [GO:0005524] ATP binding | molecular\_function | [GO:0005488] binding | [GO:0000166] nucleotide binding | [GO:0017076] purine nucleotide binding | ref|XP\_002334075.1 | ABC transporter family protein [Populus trichocarpa] | SSR | mapped |  |
| PtSIFG\_0245 | gb|DR101217 | Pinus taeda | STRR1\_71\_G07\_A033 | Pta.9202 | [GO:0005509] calcium ion binding | molecular\_function | [GO:0005488] binding | [GO:0043167] ion binding | [GO:0043169] cation binding | ref|NP\_001045400 | calmodulin 2/3/5 [Oryza sativa] | SSR | mapped |  |
| PtSIFG\_0249 | gb|CF470756 | Pinus taeda | RTDS1\_14\_F08\_A015 | Pta.16445 | [GO:0003674] molecular\_function | molecular\_function | [Lineage ends at prior level] | [Lineage ends at prior level] | [Lineage ends at prior level] | n/a | n/a | SSR | not mapped |  |
| PtSIFG\_0265 | gb|CO176556 | Pinus taeda | NDL1\_62\_E08\_A029 | Pta.17599 | [GO:0003700] transcription factor activity | molecular\_function | [GO:0005488] binding | [GO:0003676] nucleic acid binding | [GO:0003677] DNA binding | ref|NP\_001063419 | putative ZF-HD homeobox protein, Cys/His-rich dimerisation region domain containing protein [Oryza sativa] | SSR | mapped |  |
| PtSIFG\_0306 | gb|DR165426 | Pinus taeda | RTPHOS1\_5\_B04\_A029 | Pta.18781 | [GO:0003700] transcription factor activity | molecular\_function | [GO:0005488] binding | [GO:0003676] nucleic acid binding | [GO:0003677] DNA binding | ref|NP\_187687 | DNA binding / transcription factor [Arabidopsis thaliana] | SSR | not mapped |  |
| PtSIFG\_0307 | gb|CV032379 | Pinus taeda | RTNACL1\_7\_E09\_A029 | n/a | [GO:0003674] molecular\_function | molecular\_function | [Lineage ends at prior level] | [Lineage ends at prior level] | [Lineage ends at prior level] | n/a | n/a | SSR | not mapped |  |
| PtSIFG\_0338 | gb|CO169921 | Pinus taeda | NDL1\_10\_B08\_A029 | Pta.15905 | [GO:0003674] molecular\_function | molecular\_function | [Lineage ends at prior level] | [Lineage ends at prior level] | [Lineage ends at prior level] | n/a | n/a | SSR | not mapped |  |
| PtSIFG\_0349 | gb|CF401884 | Pinus taeda | RTWW1\_15\_C02\_A015 | n/a | [GO:0003674] molecular\_function | molecular\_function | [Lineage ends at prior level] | [Lineage ends at prior level] | [Lineage ends at prior level] | n/a | n/a | SSR | mapped |  |
| PtSIFG\_0371 | gb|DR068471 | Pinus taeda | RTDK1\_1\_B12\_A029 | Pta.16874 | [GO:0003674] molecular\_function | molecular\_function | [Lineage ends at prior level] | [Lineage ends at prior level] | [Lineage ends at prior level] | n/a | n/a | SSR | not mapped |  |
| PtSIFG\_0408 | gb|CF400696 | Pinus taeda | RTWW1\_7\_C08\_A015 | Pta.16295 | [GO:0005215] transporter activity | molecular\_function | [GO:0005215] transporter activity | [Lineage ends at prior level] | [Lineage ends at prior level] | ref|NP\_001050937 | nitrate transporter, putative POT family protein [Oryza sativa] | SSR | mapped |  |
| PtSIFG\_0418 | gb|CX714358 | Pinus taeda | RTPQ1\_20\_H04\_A032 | Pta.22 | [GO:0003674] molecular\_function | molecular\_function | [Lineage ends at prior level] | [Lineage ends at prior level] | [Lineage ends at prior level] | ref|XP\_001073300 | unknown protein [Ratus norvegicus] | SSR | not mapped |  |
| PtSIFG\_0424 | gb|CF392909 | Pinus taeda | RTDR3\_19\_A07\_A022 | Pta.15662 | [GO:0003674] molecular\_function | molecular\_function | [Lineage ends at prior level] | [Lineage ends at prior level] | [Lineage ends at prior level] | ref|NP\_193515 | DNA-binding protein-related [Arabidopsis thaliana] | SSR | mapped |  |
| PtSIFG\_0436 | gb|CO169703 | Pinus taeda | NDL1\_9\_B05\_A029 | Pta.8616 | [GO:0005524] ATP binding | molecular\_function | [GO:0005488] binding | [GO:0000166] nucleotide binding | [GO:0017076] purine nucleotide binding | ref|NP\_001056686 | tobacco mosaic virus helicase domain-binding protein [Oryza sativa] | SSR | not mapped |  |
| PtSIFG\_0437 | gb|DR167379 | Pinus taeda | RTPHOS1\_18\_A08\_A029 | Pta.14061 | [GO:0004462] lactoylglutathione lyase activity | molecular\_function | [GO:0003824] catalytic activity | [GO:0016829] lyase activity | [GO:0016846] carbon-sulfur lyase activity | ref|NP\_565231 | lactoylglutathione lyase family protein / glyoxalase I family protein [Arabidopsis thaliana] | SSR | mapped |  |
| PtSIFG\_0440 | gb|CF389834 | Pinus taeda | RTDR2\_5\_C04\_A021 | Pta.6258 | [GO:0003674] molecular\_function | molecular\_function | [Lineage ends at prior level] | [Lineage ends at prior level] | [Lineage ends at prior level] | n/a | n/a | SSR | mapped |  |
| PtSIFG\_0443 | gb|DR388659 | Pinus taeda | RTHG1\_29\_D12\_A029 | Pta.10949 | [GO:0005215] transporter activity | molecular\_function | [GO:0005215] transporter activity | [Lineage ends at prior level] | [Lineage ends at prior level] | ref|NP\_001058333 | cellular retinaldehyde binding/alpha-tocopherol transport family protein [Oryza sativa] | SSR | not mapped |  |
| PtSIFG\_0463 | gb|DR080700 | Pinus taeda | RTFEPL1\_24\_C08\_A029 | Pta.12215 | [GO:0003674] molecular\_function | molecular\_function | [Lineage ends at prior level] | [Lineage ends at prior level] | [Lineage ends at prior level] | n/a | n/a | SSR | mapped |  |
| PtSIFG\_0477 | gb|DR120281 | Pinus taeda | RTMG1\_28\_E05\_A029 | Pta.14955 | [GO:0003674] molecular\_function | molecular\_function | [Lineage ends at prior level] | [Lineage ends at prior level] | [Lineage ends at prior level] | ref|NP\_001044842 | Hs1pro-1 protein [Oryza sativa] | SSR | not mapped |  |
| PtSIFG\_0490 | gb|CF475807 | Pinus taeda | RTWW2\_15\_G11\_A021 | Pta.6121 | [GO:0008320] protein transmembrane transporter activity | molecular\_function | [GO:0005215] transporter activity | [GO:0022892] substrate-specific transporter activity | [GO:0008320] protein transmembrane transporter activity | ref|NP\_172429 | emp24/gp25L/p24 family protein [Oryza sativa] | SSR | not mapped |  |
| PtSIFG\_0493 | gb|CF672964 | Pinus taeda | RTCNT1\_75\_D06\_A029 | Pta.7102 | [GO:0003677] DNA binding | molecular\_function | [GO:0005488] binding | [GO:0003676] nucleic acid binding | [GO:0003677] DNA binding | ref|NP\_001046101 | homeodomain transcription factor [Oryza sativa] | SSR | mapped |  |
| PtSIFG\_0551 | gb|DR101705 | Pinus taeda | STRR1\_75\_E08\_A033 | Pta.3071 | [GO:0004449] isocitrate dehydrogenase (NAD+) activity | molecular\_function | [GO:0003824] catalytic activity | [GO:0016491] oxidoreductase activity | [GO:0016614] oxidoreductase activity, acting on CH-OH group of donors | ref|NP\_568113 | isocitrate dehydrogenase, putative [Arabidopsis thaliana] | SSR | mapped |  |
| PtSIFG\_0561 | gb|CX645741 | Pinus taeda | COLD1\_5\_B12\_A029 | n/a | [GO:0003674] molecular\_function | molecular\_function | [Lineage ends at prior level] | [Lineage ends at prior level] | [Lineage ends at prior level] | n/a | n/a | SSR | mapped |  |
| PtSIFG\_0563 | gb|CF478909 | Pinus taeda | RTWW3\_15\_H09\_A022 | Pta.6051 | [GO:0003674] molecular\_function | molecular\_function | [Lineage ends at prior level] | [Lineage ends at prior level] | [Lineage ends at prior level] | n/a | n/a | SSR | not mapped |  |
| PtSIFG\_0564 | gb|CF474913 | Pinus taeda | RTWW2\_8\_A04\_A021 | n/a | [GO:0003677] DNA binding | molecular\_function | [GO:0005488] binding | [GO:0003676] nucleic acid binding | [GO:0003677] DNA binding | ref|NP\_190848 | zinc finger family protein [Arabidopsis thaliana] | SSR | mapped | RefSeq protein assignment is based on blastx similarity to the GenBank seq.; Score = 108 bits (271), Expect = 2e-22, Identities = 46/67 (68%), Positives = 56/67 (83%), Gaps = 0/67 (0%). |
| PtSIFG\_0566 | gb|DR024231 | Pinus taeda | STRS1\_63\_A11\_A034 | Pta.13203 | [GO:0003674] molecular\_function | molecular\_function | [Lineage ends at prior level] | [Lineage ends at prior level] | [Lineage ends at prior level] | n/a | n/a | SSR | mapped |  |
| PtSIFG\_0573 | gb|DR177163 | Pinus taeda | RTMNUT1\_3\_B09\_A029 | n/a | [GO:0003674] molecular\_function | molecular\_function | [Lineage ends at prior level] | [Lineage ends at prior level] | [Lineage ends at prior level] | n/a | n/a | SSR | not mapped |  |
| PtSIFG\_0587 | gb|CF388496 | Pinus taeda | RTDR2\_3\_C09\_A021 | Pta.14472 | [GO:0003674] molecular\_function | molecular\_function | [Lineage ends at prior level] | [Lineage ends at prior level] | [Lineage ends at prior level] | n/a | n/a | SSR | mapped |  |
| PtSIFG\_0592 | gb|CF665817 | Pinus taeda | RTCNT1\_18\_E06\_A029 | Pta.9637 | [GO:0009408] response to heat | biological\_process | [GO:0050896] response to stimulus | [GO:0006950] response to stress | [GO:0009408] response to heat | ref|NP\_194497 | heat shock protein 21 [Arabidopsis thaliana] | SSR | mapped |  |
| PtSIFG\_0594 | gb|DR088945 | Pinus taeda | RTAL1\_5\_D04\_A029 | Pta.12080 | [GO:0003674] molecular\_function | molecular\_function | [Lineage ends at prior level] | [Lineage ends at prior level] | [Lineage ends at prior level] | n/a | n/a | SSR | mapped |  |
| PtSIFG\_0596 | gb|CF479238 | Pinus taeda | RTWW3\_24\_G06\_A022 | n/a | [GO:0003674] molecular\_function | molecular\_function | [Lineage ends at prior level] | [Lineage ends at prior level] | [Lineage ends at prior level] | n/a | n/a | SSR | mapped |  |
| PtSIFG\_0625 | gb|CO175900 | Pinus taeda | NDL1\_57\_G12\_A029 | n/a | [GO:0030001] metal ion transport | molecular\_function | [GO:0051234] establishment of localization | [GO:0006810] transport | [GO:0006811] ion transport | ref|NP\_001061768 | copper chaperone homolog [Oryza sativa] | SSR | mapped | RefSeq protein assignment is based on blastx similarity to the GenBank seq.; Score = 107 bits (266), Expect = 5e-22 Identities = 49/62 (79%), Positives = 60/62 (96%), Gaps = 0/62 (0%). |
| PtSIFG\_0629 | gb|DR025049 | Pinus taeda | STRS1\_69\_F10\_A034 | Pta.6346 | [GO:0009939] hydrolase activity | molecular\_function | [GO:0003824] catalytic activity | [GO:0016787] hydrolase activity | [Lineage ends at prior level] | ref|NP\_198084 | GA binding protein [Arabidopsis thaliana] | SSR | mapped |  |
| PtSIFG\_0635 | gb|CF477975 | Pinus taeda | RTWW3\_17\_H07\_A022 | Pta.7989 | [GO:0003674] molecular\_function | molecular\_function | [Lineage ends at prior level] | [Lineage ends at prior level] | [Lineage ends at prior level] | ref|NP\_001047557 | unknown protein [Oryza sativa] | SSR | mapped |  |
| PtSIFG\_0636 | gb|DR024751 | Pinus taeda | STRS1\_67\_F03\_A034 | Pta.10977 | [GO:0009828] plant-type cell wall organization | biological\_process | [GO:0009987] cellular process | [GO:0070882] cell wall organization or biogenesis | [GO:0007047] cell wall organization | ref|NP\_001051731 | alpha-expansin OsEXPA7 [Oryza sativa] | SSR | not mapped |  |
| PtSIFG\_0639 | gb|DR161521 | Pinus taeda | RTFE1\_12\_G05\_A029 | Pta.104 | [GO:0003674] molecular\_function | molecular\_function | [Lineage ends at prior level] | [Lineage ends at prior level] | [Lineage ends at prior level] | ref|NP\_563969 | unknown protein [Arabidopsis thaliana] | SSR | not mapped |  |
| PtSIFG\_0640 | gb|CF402547 | Pinus taeda | RTWW1\_21\_C11\_A015 | Pta.14949 | [GO:0004601] peroxidase activity | molecular\_function | [GO:0003824] catalytic activity | [GO:0016491] oxidoreductase activity | [GO:0016684] oxidoreductase activity, acting on peroxide as acceptor | ref|NP\_177835 | peroxidase, putative [Arabidopsis thaliana] | SSR | mapped |  |
| PtSIFG\_0652 | gb|CF402877 | Pinus taeda | RTWW1\_23\_H09\_A015 | Pta.15615 | [GO:0003676] nucleic acid binding | molecular\_function | [GO:0005488] binding | [GO:0003676] nucleic acid binding | [Lineage ends at prior level] | ref|NP\_001067437 | zinc finger CCHC-type domain containing protein [Oryza sativa] | SSR | mapped |  |
| PtSIFG\_0653 | gb|DR387173 | Pinus taeda | RTHG1\_20\_A05\_A029 | Pta.6038 | [GO:0003575] structural constituent of ribosome | molecular\_function | [GO:0005198] structural molecule activity | [GO:0003735] structural constituent of ribosome | [Lineage ends at prior level] | ref|NP\_001047832 | 60S ribosomal protein L12 [Oryza sativa] | SSR | not mapped |  |
| PtSIFG\_0662 | gb|DR016425 | Pinus taeda | STRS1\_10\_B07\_A034 | Pta.11848 | [GO:0003677] DNA binding | molecular\_function | [GO:0005488] binding | [GO:0003676] nucleic acid binding | [GO:0003677] DNA binding | ref|NP\_001059050 | basic helix-loop-helix dimerisation region bHLH domain containing protein [Oryza sativa] | SSR | not mapped |  |
| PtSIFG\_0668 | gb|CX712304 | Pinus taeda | RTPQ1\_1\_E02\_A032 | Pta.17072 | [GO:0003674] molecular\_function | molecular\_function | [Lineage ends at prior level] | [Lineage ends at prior level] | [Lineage ends at prior level] | n/a | n/a | SSR | not mapped |  |
| PtSIFG\_0715 | gb|CO176651 | Pinus taeda | NDL1\_63\_H04\_A029 | Pta.18310 | [GO:0003674] molecular\_function | molecular\_function | [Lineage ends at prior level] | [Lineage ends at prior level] | [Lineage ends at prior level] | n/a | n/a | SSR | mapped |  |
| PtSIFG\_0737 | gb|CF476612 | Pinus taeda | RTWW3\_2\_B12\_A022 | Pta.14051 | [GO:0003674] molecular\_function | molecular\_function | [Lineage ends at prior level] | [Lineage ends at prior level] | [Lineage ends at prior level] | ref|NP\_001078202 | LIGHT SENSITIVE HYPOCOTYLS 4 (LSH4) [Arabidopsis thaliana] | SSR | not mapped |  |
| PtSIFG\_0740 | gb|DR025602 | Pinus taeda | STRS1\_72\_D06\_A034 | Pta.7440 | [GO:0005509] calcium ion binding | molecular\_function | [GO:0005488] binding | [GO:0043167] ion binding | [GO:0043169] cation binding | ref|NP\_195592 | calcium-binding EF hand family protein [Arabidopsis thaliana] | SSR | mapped |  |
| PtSIFG\_0745 | gb|CO364441 | Pinus taeda | RTK1\_15\_E12\_A029 | Pta.16166 | [GO:0003674] molecular\_function | molecular\_function | [Lineage ends at prior level] | [Lineage ends at prior level] | [Lineage ends at prior level] | n/a | n/a | SSR | mapped |  |
| PtSIFG\_1005 | gb|AI812884 | Pinus taeda | 22B11 | Pta.11928 | [GO:0003674] molecular\_function | molecular\_function | [Lineage ends at prior level] | [Lineage ends at prior level] | [Lineage ends at prior level] | ref|NP\_177032 | unknown protein [Arabidopsis thaliana] | SSR | mapped |  |
| PtSIFG\_1008 | gb|BQ695936 | Pinus taeda | NXPV\_034\_E06 | Pta.7222 | [GO:0003674] molecular\_function | molecular\_function | [Lineage ends at prior level] | [Lineage ends at prior level] | [Lineage ends at prior level] | n/a | n/a | SSR | not mapped |  |
| PtSIFG\_1018 | gb|BQ696488 | Pinus taeda | NXPV\_041\_H02 | Pta.260 | [GO:0004568] chitinase activity | molecular\_function | [GO:0003824] catalytic activity | [GO:0016787] hydrolase activity | [GO:0016798] hydrolase activity, acting on glycosyl bonds | ref|NP\_172076 | chitinase [Arabidopsis thaliana] | SSR | mapped |  |
| PtSIFG\_1030 | gb|AW981840 | Pinus taeda | PC19C12 | n/a | [GO:0003674] molecular\_function | molecular\_function | [Lineage ends at prior level] | [Lineage ends at prior level] | [Lineage ends at prior level] | n/a | n/a | SSR | not mapped |  |
| PtSIFG\_1032 | gb|BI397639 | Pinus taeda | NXPV\_102\_F09 | Pta.12090 | [GO:0003674] molecular\_function | molecular\_function | [Lineage ends at prior level] | [Lineage ends at prior level] | [Lineage ends at prior level] | n/a | n/a | SSR | not mapped |  |
| PtSIFG\_1052 | gb|AW056658 | Pinus taeda | ST54C06 | Pta.2245 | [GO:0003674] molecular\_function | molecular\_function | [Lineage ends at prior level] | [Lineage ends at prior level] | [Lineage ends at prior level] | n/a | n/a | SSR | mapped |  |
| PtSIFG\_1055 | gb|AW697582 | Pinus taeda | ST59C11 | n/a | [GO:0003674] molecular\_function | molecular\_function | [Lineage ends at prior level] | [Lineage ends at prior level] | [Lineage ends at prior level] | n/a | n/a | SSR | mapped |  |
| PtSIFG\_1060 | gb|AW697707 | Pinus taeda | ST65F12 | Pta.1906 | [GO:0003723] RNA binding | molecular\_function | [GO:0005488] binding | [GO:0003676] nucleic acid binding | [GO:0003723] RNA binding | ref|NP\_196239 | RNA binding protein, putative [Arabidopsis thaliana] | SSR | not mapped |  |
| PtSIFG\_1062 | gb|AW225917 | Pinus taeda | ST75C03 | n/a | [GO:0003674] molecular\_function | molecular\_function | [Lineage ends at prior level] | [Lineage ends at prior level] | [Lineage ends at prior level] | n/a | n/a | SSR | mapped |  |
| PtSIFG\_1065 | gb|AW226075 | Pinus taeda | ST77A10 | n/a | [GO:0003674] molecular\_function | molecular\_function | [Lineage ends at prior level] | [Lineage ends at prior level] | [Lineage ends at prior level] | n/a | n/a | SSR | mapped |  |
| PtSIFG\_1066 | gb|AW226133 | Pinus taeda | ST77G01 | n/a | [GO:0003674] molecular\_function | molecular\_function | [Lineage ends at prior level] | [Lineage ends at prior level] | [Lineage ends at prior level] | n/a | n/a | SSR | not mapped |  |
| PtSIFG\_1102 | gb|BQ700433 | Pinus taeda | NXRV105\_F03 | Pta.12798 | [GO:0003674] molecular\_function | molecular\_function | [Lineage ends at prior level] | [Lineage ends at prior level] | [Lineage ends at prior level] | n/a | n/a | SSR | not mapped |  |
| PtSIFG\_1110 | gb|AW888197 | Pinus taeda | NXNV\_126\_A10 | n/a | [GO:0003674] molecular\_function | molecular\_function | [Lineage ends at prior level] | [Lineage ends at prior level] | [Lineage ends at prior level] | n/a | n/a | SSR | not mapped |  |
| PtSIFG\_1113 | gb|BQ702924 | Pinus taeda | NXSI\_134\_C12 | Pta.11893 | [GO:0003674] molecular\_function | molecular\_function | [Lineage ends at prior level] | [Lineage ends at prior level] | [Lineage ends at prior level] | n/a | n/a | SSR | mapped |  |
| PtSIFG\_1123 | gb|BQ634623 | Pinus taeda | NXRV071\_C04 | Pta.12740 | [GO:0003674] molecular\_function | molecular\_function | [Lineage ends at prior level] | [Lineage ends at prior level] | [Lineage ends at prior level] | n/a | n/a | SSR | mapped |  |
| PtSIFG\_1166 | gb|DR388699 | Pinus taeda | RTHG1\_30\_A04\_A029 | Pta.9173 | [GO:0016787] hydrolase activity | molecular\_function | [GO:0003824] catalytic activity | [GO:0016787] hydrolase activity | [Lineage ends at prior level] | ref|NP\_001061676 | NUDIX hydrolase domain containing protein [Oryza sativa] | SSR | mapped |  |
| PtSIFG\_1184 | gb|BQ700196 | Pinus taeda | NXRV102\_D11 | n/a | [GO:0003674] molecular\_function | molecular\_function | [Lineage ends at prior level] | [Lineage ends at prior level] | [Lineage ends at prior level] | n/a | n/a | SSR | not mapped |  |
| PtSIFG\_1185 | gb|DR056188 | Pinus taeda | RTCA1\_28\_D06\_A029 | Pta.13664 | [GO:0003674] molecular\_function | molecular\_function | [Lineage ends at prior level] | [Lineage ends at prior level] | [Lineage ends at prior level] | n/a | n/a | SSR | not mapped |  |
| PtSIFG\_1190 | gb|BQ655604 | Pinus taeda | NXRV096\_G03 | n/a | [GO:0003674] molecular\_function | molecular\_function | [Lineage ends at prior level] | [Lineage ends at prior level] | [Lineage ends at prior level] | n/a | n/a | SSR | mapped |  |
| PtSIFG\_1207 | gb|BM158590 | Pinus taeda | NXLV\_036\_F01 | n/a | [GO:0003674] molecular\_function | molecular\_function | [Lineage ends at prior level] | [Lineage ends at prior level] | [Lineage ends at prior level] | n/a | n/a | SSR | mapped |  |
| PtSIFG\_1212 | gb|BQ197323 | Pinus taeda | NXLV112\_E03 | Pta.13801 | [GO:0003674] molecular\_function | molecular\_function | [Lineage ends at prior level] | [Lineage ends at prior level] | [Lineage ends at prior level] | n/a | n/a | SSR | mapped |  |
| PtSIFG\_1217 | gb|CF393828 | Pinus taeda | RTDS2\_1\_F07\_A021 | Pta.11614 | [GO:0004527] exonuclease activity | molecular\_function | [GO:0003824] catalytic activity | [GO:0016787] hydrolase activity | [GO:0016788] hydrolase activity, acting on ester bonds | ref|NP\_001042752 | exonuclease domain containing protein [Oryza sativa] | SSR | not mapped |  |
| PtSIFG\_1241 | gb|DR015587 | Pinus taeda | STRS1\_4\_A04\_A034 | Pta.13123 | [GO:0003723] RNA binding | molecular\_function | [GO:0005488] binding | [GO:0003676] nucleic acid binding | [GO:0003723] RNA binding | ref|NP\_001044971 | RNA recognition motif [Oryza sativa] | SSR | not mapped |  |
| PtSIFG\_1252 | gb|BQ697807 | Pinus taeda | NXPV\_061\_A12 | Pta.14196 | [GO:0003674] molecular\_function | molecular\_function | [Lineage ends at prior level] | [Lineage ends at prior level] | [Lineage ends at prior level] | n/a | n/a | SSR | mapped |  |
| PtSIFG\_1260 | gb|CD021007 | Pinus taeda | NXNV\_128\_A04 | n/a | [GO:0003674] molecular\_function | molecular\_function | [Lineage ends at prior level] | [Lineage ends at prior level] | [Lineage ends at prior level] | n/a | n/a | SSR | mapped |  |
| PtSIFG\_1262 | gb|DR011911 | Pinus taeda | HEAT1\_8\_E08\_A029 | Pta.6567 | [GO:0003674] molecular\_function | molecular\_function | [Lineage ends at prior level] | [Lineage ends at prior level] | [Lineage ends at prior level] | n/a | n/a | SSR | mapped |  |
| PtSIFG\_1295 | gb|BG040898 | Pinus taeda | NXSI\_116\_D08 | Pta.7101 | [GO:0003674] molecular\_function | molecular\_function | [Lineage ends at prior level] | [Lineage ends at prior level] | [Lineage ends at prior level] | ref|NP\_565716 | unknown protein [Arabidopsis thaliana] | SSR | not mapped |  |
| PtSIFG\_1318 | gb|DT634927 | Pinus taeda | PIMGB30 | Pta.5296 | [GO:0008270] zinc ion binding | molecular\_function | [GO:0005488] binding | [GO:0043167] ion binding | [GO:0043169] cation binding | ref|NP\_565271 | UBX domain-containing protein-2 [Arabidopsis thaliana] | SSR | mapped |  |
| PtSIFG\_1325 | gb|CF475353 | Pinus taeda | RTWW2\_13\_A05\_A021 | Pta.13709 | [GO:0008536] Ran GTPase binding | molecular\_function | [GO:0005488] binding | [GO:0005515] protein binding | [GO:0019899] enzyme binding | ref|NP\_173417 | regulator of chromosome condensation family protein [Arabidopsis thaliana] | SSR | mapped |  |
| PtSIFG\_2229 | gb|CO410345 | Pinus taeda | PIAL188 | Pta.5403 | [GO:0009073] aromatic amino acid family biosynthetic process, shikimate pathway | molecular\_function | [GO:0008152] metabolic process | [GO:0044237] cellular metabolic process | [GO:0006766] vitamin metabolic process | ref|NP\_001060314 | phospho-2-dehydro-3-deoxyheptonate aldolase 1, chloroplast precursor [Oryza sativa] | SSR | mapped |  |
| PtSIFG\_2461 | gb|DT626577 | Pinus taeda | PIMAC32 | Pta.19202 | [GO:0003674] molecular\_function | molecular\_function | [Lineage ends at prior level] | [Lineage ends at prior level] | [Lineage ends at prior level] | n/a | n/a | SSR | not mapped |  |
| PtSIFG\_2510 | gb|DN463709 | Pinus taeda | RPIJ226 | n/a | [GO:0003677] DNA binding | molecular\_function | [GO:0005488] binding | [GO:0003676] nucleic acid binding | [GO:0003677] DNA binding | ref|XP\_002528315 | r2r3-myb transcription factor, putative [Ricinus communis] | SSR | not mapped | RefSeq protein assignment is based on blastx similarity to the GenBank seq.; Score = 196 bits (498), Expect = 2e-48, Identities = 88/105 (83%), Positives = 98/105 (93%), Gaps = 0/105 (0%) |
| PtSIFG\_4102 | gb|DR096547 | Pinus taeda | STRR1\_28\_E01\_A033 | n/a | [GO:0003674] molecular\_function | molecular\_function | [Lineage ends at prior level] | [Lineage ends at prior level] | [Lineage ends at prior level] | n/a | n/a | SSR | not mapped |  |
| PtSIFG\_4110 | gb|CV032058 | Pinus taeda | RTNACL1\_5\_B05\_A029 | n/a | [GO:0003674] molecular\_function | molecular\_function | [Lineage ends at prior level] | [Lineage ends at prior level] | [Lineage ends at prior level] | n/a | n/a | SSR | not mapped |  |
| PtSIFG\_4133 | gb|CF395323 | Pinus taeda | RTDS2\_11\_G01\_A021 | Pta.15890 | [GO:0003674] molecular\_function | molecular\_function | [Lineage ends at prior level] | [Lineage ends at prior level] | [Lineage ends at prior level] | n/a | n/a | SSR | not mapped |  |
| PtSIFG\_4160 | gb|CO159086 | Pinus taeda | FLD1\_11\_F07\_A029 | n/a | [GO:0003674] molecular\_function | molecular\_function | [Lineage ends at prior level] | [Lineage ends at prior level] | [Lineage ends at prior level] | n/a | n/a | SSR | not mapped |  |
| PtSIFG\_4177 | gb|CN784862 | Pinus taeda | PIAAR55 | Pta.13875 | [GO:0003674] molecular\_function | molecular\_function | [Lineage ends at prior level] | [Lineage ends at prior level] | [Lineage ends at prior level] | n/a | n/a | SSR | not mapped |  |
| PtSIFG\_4192 | gb|DR160121 | Pinus taeda | RTFE1\_3\_G12\_A029 | Pta.17452 | [GO:0003824] catalytic activity | molecular\_function | [GO:0003824] catalytic activity | [Lineage ends at prior level] | [Lineage ends at prior level] | ref|NP\_001056169 | protein phosphatase type 2C [Oryza sativa] | SSR | not mapped |  |
| PtSIFG\_4202 | gb|DR165611 | Pinus taeda | RTPHOS1\_6\_F02\_A029 | Pta.18176 | [GO:0003674] molecular\_function | molecular\_function | [Lineage ends at prior level] | [Lineage ends at prior level] | [Lineage ends at prior level] | ref|NP\_563958 | unknown protein [Arabidopsis thaliana] | SSR | not mapped |  |
| PtSIFG\_4207 | gb|CF386469 | Pinus taeda | RTDR1\_14\_A10\_A015 | Pta.15071 | [GO:0003674] molecular\_function | molecular\_function | [Lineage ends at prior level] | [Lineage ends at prior level] | [Lineage ends at prior level] | n/a | n/a | SSR | not mapped |  |
| PtSIFG\_4213 | gb|CF668041 | Pinus taeda | RTCNT1\_34\_B11\_A029 | Pta.13660 | [GO:0003674] molecular\_function | molecular\_function | [Lineage ends at prior level] | [Lineage ends at prior level] | [Lineage ends at prior level] | n/a | n/a | SSR | mapped |  |
| PtSIFG\_4218 | gb|CO175197 | Pinus taeda | NDL1\_53\_E02\_A029 | Pta.18433 | [GO:0003674] molecular\_function | molecular\_function | [Lineage ends at prior level] | [Lineage ends at prior level] | [Lineage ends at prior level] | n/a | n/a | SSR | mapped |  |
| PtSIFG\_4222 | gb|DR110547 | Pinus taeda | RTS1\_11\_B10\_A029 | n/a | [GO:0003674] molecular\_function | molecular\_function | [Lineage ends at prior level] | [Lineage ends at prior level] | [Lineage ends at prior level] | n/a | n/a | SSR | mapped |  |
| PtSIFG\_4232 | gb|CO364859 | Pinus taeda | RTK1\_22\_E12\_A029 | n/a | [GO:0003700] transcription factor activity | molecular\_function | [GO:0005488] binding | [GO:0003676] nucleic acid binding | [GO:0003677] DNA binding | ref|NP\_001063215 | AP2-1 protein, fragment [Oryza sativa] | SSR | not mapped |  |
| PtSIFG\_4233 | gb|CO361093 | Pinus taeda | NDL2\_2\_E05\_A029 | n/a | [GO:0005507] copper ion binding | molecular\_function | [GO:0005488] binding | [GO:0043167] ion binding | [GO:0043169] cation binding | ref|NP\_178388 | plantacyanin [Arabidopsis thaliana] | SSR | mapped | RefSeq protein assignment is based on blastx similarity to the GenBank seq.; Score = 108 bits (269), Expect = 4e-22, Identities = 54/99 (54%), Positives = 65/99 (65%), Gaps = 2/99 (2%). |
| PtSIFG\_4239 | gb|CF470350 | Pinus taeda | RTDS1\_17\_B07\_A015 | Pta.16426 | [GO:0003674] molecular\_function | molecular\_function | [Lineage ends at prior level] | [Lineage ends at prior level] | [Lineage ends at prior level] | ref|NP\_001060604 | unknown protein [Oryza sativa] | SSR | not mapped |  |
| PtSIFG\_4245 | gb|CF386139 | Pinus taeda | RTDR1\_8\_B05\_A015 | Pta.990 | [GO:0003674] molecular\_function | molecular\_function | [Lineage ends at prior level] | [Lineage ends at prior level] | [Lineage ends at prior level] | n/a | n/a | SSR | mapped |  |
| PtSIFG\_4249 | gb|CF395415 | Pinus taeda | RTDS2\_11\_F12\_A021 | n/a | [GO:0003674] molecular\_function | molecular\_function | [Lineage ends at prior level] | [Lineage ends at prior level] | [Lineage ends at prior level] | n/a | n/a | SSR | mapped |  |
| PtSIFG\_4282 | gb|CX646966 | Pinus taeda | COLD1\_12\_D04\_A029 | n/a | [GO:0003674] molecular\_function | molecular\_function | [Lineage ends at prior level] | [Lineage ends at prior level] | [Lineage ends at prior level] | n/a | n/a | SSR | mapped |  |
| PtSIFG\_4304 | gb|BM492561 | Pinus taeda | NXRV\_028\_B02 | n/a | [GO:0003674] molecular\_function | molecular\_function | [Lineage ends at prior level] | [Lineage ends at prior level] | [Lineage ends at prior level] | n/a | n/a | SSR | mapped |  |
| PtSIFG\_4315 | gb|BQ634773 | Pinus taeda | NXRV073\_A11 | n/a | [GO:0003674] molecular\_function | molecular\_function | [Lineage ends at prior level] | [Lineage ends at prior level] | [Lineage ends at prior level] | n/a | n/a | SSR | mapped |  |
| PtSIFG\_4378 | gb|CX715556 | Pinus taeda | RTPQ1\_35\_A07\_A032 | Pta.3299 | [GO:0016629] 12-oxophytodienoate reductase activity | molecular\_function | [GO:0003824] catalytic activity | [GO:0016491] oxidoreductase activity | [GO:0016627] oxidoreductase activity, acting on the CH-CH group of donors | ref|NP\_177794 | 12-oxophytodienoate reductase 1 [Arabidopsis thaliana] | SSR | mapped |  |
| PtSIFG\_4380 | gb|CO201222 | Pinus taeda | RTCNT2\_4\_H09\_A029 | Pta.15586 | [GO:0008234] cysteine-type peptidase activity | molecular\_function | [GO:0003824] catalytic activity | [GO:0016787] hydrolase activity | [GO:0008233] peptidase activity | ref|NP\_680113 | cysteine-type peptidase [Arabidopsis thaliana] | SSR | not mapped |  |
| PtSIFG\_4391 | gb|DR019194 | Pinus taeda | STRS1\_28\_H10\_A034 | Pta.423 | [GO:0016207] 4-coumarate-CoA ligase activity | molecular\_function | [GO:0003824] catalytic activity | [GO:0016874] ligase activity | [GO:0016877] ligase activity, forming carbon-sulfur bonds | ref|NP\_188761 | 4-coumarate-CoA ligase enzyme [Arabidopsis thaliana] | SSR | not mapped |  |
| PtSIFG\_4393 | gb|CX715983 | Pinus taeda | RTPQ1\_39\_E01\_A032 | Pta.16652 | [GO:0003674] molecular\_function | molecular\_function | [Lineage ends at prior level] | [Lineage ends at prior level] | [Lineage ends at prior level] | ref|NP\_195384 | unknown protein [Arabidopsis thaliana] | SSR | not mapped |  |
| PtSIFG\_4394 | gb|DR385346 | Pinus taeda | RTHG1\_8\_D04\_A029 | Pta.12687 | [GO:0003674] molecular\_function | molecular\_function | [Lineage ends at prior level] | [Lineage ends at prior level] | [Lineage ends at prior level] | ref|NP\_176881 | LOB domain protein 40 / lateral organ boundaries domain protein 40 [Arabidopsis thaliana] | SSR | mapped |  |
| PtSIFG\_4407 | gb|CO168018 | Pinus taeda | FLD1\_72\_F01\_A029 | n/a | [GO:0003674] molecular\_function | molecular\_function | [Lineage ends at prior level] | [Lineage ends at prior level] | [Lineage ends at prior level] | n/a | n/a | SSR | not mapped |  |
| PtSIFG\_4415 | gb|DR092449 | Pinus taeda | STRR1\_1\_A09\_A033 | Pta.20587 | [GO:0009740] gibberellic acid mediated signaling | biological\_process | [GO:0009987] cellular process | [GO:0007154] cell communication | [GO:0007165] signal transduction | ref|NP\_197027 | gibberellin regulated protein 4 [Arabidopsis thaliana] | SSR | mapped |  |
| PtSIFG\_4438 | gb|CF668559 | Pinus taeda | RTCNT1\_37\_F09\_A029 | Pta.17149 | [GO:0003674] molecular\_function | molecular\_function | [Lineage ends at prior level] | [Lineage ends at prior level] | [Lineage ends at prior level] | n/a | n/a | SSR | mapped |  |
| PtSIFG\_4446 | gb|CF474477 | Pinus taeda | RTWW2\_9\_H08\_A021 | Pta.9940 | [GO:0005509] calcium ion binding | molecular\_function | [GO:0005488] binding | [GO:0043167] ion binding | [GO:0043169] cation binding | ref|NP\_001053764 | calcium-binding EF-hand family protein [Oryza sativa] | SSR | mapped |  |
| PtSIFG\_4447 | gb|DR020650 | Pinus taeda | STRS1\_38\_H11\_A034 | Pta.336 | [GO:0016298] lipase activity | molecular\_function | [GO:0003824] catalytic activity | [GO:0016787] hydrolase activity | [GO:0016788] hydrolase activity, acting on ester bonds | ref|NP\_001064968 | epoxide hydrolase, putative [Oryza sativa] | SSR | mapped |  |
| PtSIFG\_4454 | gb|DR101834 | Pinus taeda | STRR1\_76\_E02\_A033 | Pta.6343 | [GO:0009739] gibberellic acid mediated signaling | biological\_process | [GO:0009987] cellular process | [GO:0007154] cell communication | [GO:0007165] signal transduction | ref|NP\_001055630 | gibberellin regulated protein 2 precursor [Oryza sativa] | SSR | mapped |  |
| PtSIFG\_4458 | gb|CF476608 | Pinus taeda | RTWW3\_2\_C07\_A022 | Pta.16829 | [GO:0003677] DNA binding | molecular\_function | [GO:0005488] binding | [GO:0003676] nucleic acid binding | [GO:0003677] DNA binding | ref|NP\_001043797 | WRKY transcription factor 34 [Oryza sativa] | SSR | mapped |  |
| PtSIFG\_4472 | gb|DR052128 | Pinus taeda | RTCA1\_2\_C11\_A029 | Pta.16857 | [GO:0005524] ATP binding | molecular\_function | [GO:0005488] binding | [GO:0000166] nucleotide binding | [GO:0017076] purine nucleotide binding | ref|NP\_001059451 | adenylate kinase family protein [Oryza sativa] | SSR | mapped |  |
| PtSIFG\_4493 | gb|DR047947 | Pinus taeda | RTBOR1\_5\_E02\_A029 | Pta.12365 | [GO:0003674] molecular\_function | molecular\_function | [Lineage ends at prior level] | [Lineage ends at prior level] | [Lineage ends at prior level] | n/a | n/a | SSR | mapped |  |
| PtSIFG\_4502 | gb|CF473835 | Pinus taeda | RTWW2\_19\_C07\_A021 | Pta.16639 | [GO:0003824] catalytic activity | molecular\_function | [GO:0003824] catalytic activity | [Lineage ends at prior level] | [Lineage ends at prior level] | ref|NP\_001056894 | esterase/lipase/thioesterase domain containing protein [Oryza sativa] | SSR | mapped |  |
| PtTX2037 | gb|AF143959 | Pinus taeda | 1213r | n/a | n/a | n/a | n/a | n/a | n/a | n/a | n/a | SSR | mapped |  |
| PtTX2080 | gb|AF286600 | Pinus taeda | 6j11f | n/a | n/a | n/a | n/a | n/a | n/a | n/a | n/a | SSR | mapped |  |
| PtTX2091 | gb|AF333776 | Pinus taeda | 10g3tot | n/a | n/a | n/a | n/a | n/a | n/a | n/a | n/a | SSR | mapped |  |
| PtTX2093 | gb|AF286602 | Pinus taeda | 10i22tot | n/a | n/a | n/a | n/a | n/a | n/a | n/a | n/a | SSR | mapped |  |
| PtTX2094 | gb|AF286603 | Pinus taeda | 10m23tot | n/a | n/a | n/a | n/a | n/a | n/a | n/a | n/a | SSR | mapped |  |
| PtTX2123 | gb|AF459422 | Pinus taeda | 18b6f | n/a | n/a | n/a | n/a | n/a | n/a | n/a | n/a | SSR | mapped |  |
| PtTX2146 | gb|AF143963 | Pinus taeda | 18b11f | n/a | n/a | n/a | n/a | n/a | n/a | n/a | n/a | SSR | mapped |  |
| PtTX2164 | gb|AF143964 | Pinus taeda | 18a16r | n/a | n/a | n/a | n/a | n/a | n/a | n/a | n/a | SSR | mapped |  |
| PtTX2189 | gb|AF393980 | Pinus taeda | 16o15tot | n/a | n/a | n/a | n/a | n/a | n/a | n/a | n/a | SSR | mapped |  |
| PtTX3\_lp3-1 | gb|U52865 | Pinus taeda | n/a | Pta.12396 | [GO:0006950] response to stress | biological\_process | [GO:0050896] response to stimulus | [GO:0006950] response to stress | [Lineage ends at prior level] | ref|NP\_0010658412 | Pollen-specific desiccation-associated LLA23 protein [Oryza sativa] | ESTP | mapped | UniGene assignment is based on blastn similarity to the homologous Pinus taeda EST gb|DR094888; Score = 784 bits (424), Expect = 0.0, Identities = 434/439 (98%), Gaps = 0/439 (0%). |
| PtTX3001 | gb|AF393981 | Pinus taeda | 33h23f | n/a | n/a | n/a | n/a | n/a | n/a | n/a | n/a | SSR | mapped |  |
| PtTX3011 | gb|AF143965 | Pinus taeda | 33n1f | n/a | n/a | n/a | n/a | n/a | n/a | n/a | n/a | SSR | mapped |  |
| PtTX3013 | gb|AF143966 | Pinus taeda | 59k24f | n/a | n/a | n/a | n/a | n/a | n/a | n/a | n/a | SSR | mapped |  |
| PtTX3019 | gb|AF277846 | Pinus taeda | 60e22r | n/a | n/a | n/a | n/a | n/a | n/a | n/a | n/a | SSR | mapped |  |
| PtTX3020 | gb|AF143969 | Pinus taeda | 64f20con | n/a | n/a | n/a | n/a | n/a | n/a | n/a | n/a | SSR | mapped |  |
| PtTX3025 | gb|AF143970 | Pinus taeda | 76g10f | n/a | n/a | n/a | n/a | n/a | n/a | n/a | n/a | SSR | mapped |  |
| PtTX3027 | gb|AF441508 | Pinus taeda | 60l4r | n/a | n/a | n/a | n/a | n/a | n/a | n/a | n/a | SSR | mapped |  |
| PtTX3029 | gb|AF441509 | Pinus taeda | 58b2f | n/a | n/a | n/a | n/a | n/a | n/a | n/a | n/a | SSR | mapped |  |
| PtTX3032 | gb|AF143973 | Pinus taeda | 28l13g | n/a | n/a | n/a | n/a | n/a | n/a | n/a | n/a | SSR | mapped |  |
| PtTX3034 | gb|AF143974 | Pinus taeda | 57g14con | n/a | n/a | n/a | n/a | n/a | n/a | n/a | n/a | SSR | mapped |  |
| PtTX3037 | gb|AF143975 | Pinus taeda | 75j7f | n/a | n/a | n/a | n/a | n/a | n/a | n/a | n/a | SSR | mapped |  |
| PtTX3045 | gb|AF333781 | Pinus taeda | 120f10r | n/a | n/a | n/a | n/a | n/a | n/a | n/a | n/a | SSR | mapped |  |
| PtTX3047 | gb|AF286606 | Pinus taeda | 120d8r | n/a | n/a | n/a | n/a | n/a | n/a | n/a | n/a | SSR | mapped |  |
| PtTX3049 | gb|AF286607 | Pinus taeda | 120d12r | n/a | n/a | n/a | n/a | n/a | n/a | n/a | n/a | SSR | mapped |  |
| PtTX3052 | gb|AF441514 | Pinus taeda | 47a7f | n/a | n/a | n/a | n/a | n/a | n/a | n/a | n/a | SSR | mapped |  |
| PtTX3055 | gb|AF286608 | Pinus taeda | 48m13f | n/a | n/a | n/a | n/a | n/a | n/a | n/a | n/a | SSR | mapped |  |
| PtTX3058 | gb|AF441516 | Pinus taeda | 34b20f | n/a | n/a | n/a | n/a | n/a | n/a | n/a | n/a | SSR | mapped |  |
| PtTX3063 | gb|AF286610 | Pinus taeda | 48f7f | n/a | n/a | n/a | n/a | n/a | n/a | n/a | n/a | SSR | mapped |  |
| PtTX3081 | gb|AF441520 | Pinus taeda | 65n18f | n/a | n/a | n/a | n/a | n/a | n/a | n/a | n/a | SSR | mapped |  |
| PtTX3097 | gb|AF441525 | Pinus taeda | 62c1f | n/a | n/a | n/a | n/a | n/a | n/a | n/a | n/a | SSR | mapped |  |
| PtTX3105 | gb|AF333784 | Pinus taeda | 64c1f | n/a | n/a | n/a | n/a | n/a | n/a | n/a | n/a | SSR | mapped |  |
| PtTX3107 | gb|AF333785 | Pinus taeda | 64f1f | n/a | n/a | n/a | n/a | n/a | n/a | n/a | n/a | SSR | mapped |  |
| PtTX3110 | gb|AF441530 | Pinus taeda | 63o18f | n/a | n/a | n/a | n/a | n/a | n/a | n/a | n/a | SSR | mapped |  |
| PtTX3114 | gb|AF441531 | Pinus taeda | 75p23f | n/a | n/a | n/a | n/a | n/a | n/a | n/a | n/a | SSR | mapped |  |
| PtTX3116 | gb|AF333787 | Pinus taeda | 70l7f | n/a | n/a | n/a | n/a | n/a | n/a | n/a | n/a | SSR | mapped |  |
| PtTX3117 | gb|AF441533 | Pinus taeda | 49h3r | n/a | n/a | n/a | n/a | n/a | n/a | n/a | n/a | SSR | mapped |  |
| PtTX3120 | gb|AF441534 | Pinus taeda | 61i19r | n/a | n/a | n/a | n/a | n/a | n/a | n/a | n/a | SSR | mapped |  |
| PtTX4001 | gb|AF286619 | Pinus taeda | 161c20f | n/a | n/a | n/a | n/a | n/a | n/a | n/a | n/a | SSR | mapped |  |
| PtTX4003 | gb|AF286620 | Pinus taeda | 162c8f | n/a | n/a | n/a | n/a | n/a | n/a | n/a | n/a | SSR | mapped |  |
| PtTX4030 | gb|AF324775 | Pinus taeda | 162b4f | n/a | n/a | n/a | n/a | n/a | n/a | n/a | n/a | SSR | mapped |  |
| PtTX4033 | gb|BV728878 | Pinus taeda | 162i24r | n/a | n/a | n/a | n/a | n/a | n/a | n/a | n/a | SSR | mapped |  |
| PtTX4036 | gb|AF286626 | Pinus taeda | 161d13r | n/a | n/a | n/a | n/a | n/a | n/a | n/a | n/a | SSR | mapped |  |
| PtTX4056 | gb|AF324776 | Pinus taeda | 159g4 | n/a | n/a | n/a | n/a | n/a | n/a | n/a | n/a | SSR | mapped |  |
| PtTX4058 | gb|AY330146 | Pinus taeda | 159n5r | n/a | n/a | n/a | n/a | n/a | n/a | n/a | n/a | SSR | mapped |  |
| PtTX4062 | gb|AF286628 | Pinus taeda | 161d18r | n/a | n/a | n/a | n/a | n/a | n/a | n/a | n/a | SSR | mapped |  |
| PtTX4079 | gb|AF441549 | Pinus taeda | 162h5f | n/a | n/a | n/a | n/a | n/a | n/a | n/a | n/a | SSR | mapped |  |
| PtTX4090 | gb|AF324780 | Pinus taeda | 167i18r | n/a | n/a | n/a | n/a | n/a | n/a | n/a | n/a | SSR | mapped |  |
| PtTX4092 | gb|AF324781 | Pinus taeda | 167m16r | n/a | n/a | n/a | n/a | n/a | n/a | n/a | n/a | SSR | mapped |  |
| PtTX4093 | gb|AF324782 | Pinus taeda | 167j23r | n/a | n/a | n/a | n/a | n/a | n/a | n/a | n/a | SSR | mapped |  |
| PtTX4112 | gb|AF324785 | Pinus taeda | 162g16f | n/a | n/a | n/a | n/a | n/a | n/a | n/a | n/a | SSR | mapped |  |
| PtTX4137 | gb|AF324787 | Pinus taeda | 167m16 | n/a | n/a | n/a | n/a | n/a | n/a | n/a | n/a | SSR | mapped |  |
| PtTX4147 | gb|AF324789 | Pinus taeda | 169b13 | n/a | n/a | n/a | n/a | n/a | n/a | n/a | n/a | SSR | mapped |  |
| PtTX4181 | gb|BV728850 | Pinus taeda | 161b9f | n/a | n/a | n/a | n/a | n/a | n/a | n/a | n/a | SSR | mapped |  |
| PtTX4214 | gb|AF455077 | Pinus taeda | 179c9 | n/a | n/a | n/a | n/a | n/a | n/a | n/a | n/a | SSR | mapped |  |
| PtTX4215 | gb|AF455078 | Pinus taeda | 179a23 | n/a | n/a | n/a | n/a | n/a | n/a | n/a | n/a | SSR | mapped |  |
| PtTX4228 | gb|AF455080 | Pinus taeda | 179f19 | n/a | n/a | n/a | n/a | n/a | n/a | n/a | n/a | SSR | mapped |  |
| PtWS1\_PCBER-PT1 | gb|AF242490 | Pinus taeda | n/a | Pta.376 | [GO:0032442] phenylcoumaran benzylic ether reductase activity | molecular\_function | [GO:0003824] catalytic activity | [GO:0016491] oxidoreductase activity | [GO:0016614] oxidoreductase activity, acting on CH-OH group of donors | gb|AAF64173 | phenylcoumaran benzylic ether reductase PT1 [Pinus taeda] | ESTP | mapped |  |
| RPtest11 | gb|AA557106 | Pinus taeda | 6N7H | Pta.563 | [GO:0008080] N-acetyltransferase activity | molecular\_function | [GO:0003824] catalytic activity | [GO:0016740] transferase activity | [GO:0016746] transferase activity, transferring acyl groups | ref|NP\_196882 | GCN5-related N-acetyltransferase, putative [Arabidopsis thaliana] | SSR | not mapped |  |
| RPtest5 | gb|AA556655 | Pinus taeda | 2C9F | Pta.5366 | [GO:0003674] molecular\_function | molecular\_function | [Lineage ends at prior level] | [Lineage ends at prior level] | [Lineage ends at prior level] | ref|NP\_189345 | unknown protein [Arabidopsis thaliana] | SSR | not mapped |  |
| SsrPt\_ctg1376 | gb|CD024501 | Pinus taeda | NXRV\_043\_G05 | n/a | [GO:0003674] molecular\_function | molecular\_function | [Lineage ends at prior level] | [Lineage ends at prior level] | [Lineage ends at prior level] | n/a | n/a | SSR | mapped |  |
| SsrPt\_ctg7141 | gb|DT626103 | Pinus taeda | PIMA711 | Pta.10195 | [GO:0003674] molecular\_function | molecular\_function | [Lineage ends at prior level] | [Lineage ends at prior level] | [Lineage ends at prior level] | n/a | n/a | SSR | mapped |  |
| SsrPt\_ctg7444 | gb|CD028062 | Pinus taeda | NXNV001F01 | Pta.204 | [GO:0003674] molecular\_function | molecular\_function | [Lineage ends at prior level] | [Lineage ends at prior level] | [Lineage ends at prior level] | ref|NP\_001068405 | Translationally controlled tumor protein family [Oryza sativa] | SSR | mapped |  |
| SsrPt\_ctg9249 | gb|BE761900 | Pinus taeda | NXCI\_071\_C08 | Pta.5267 | [GO:0003700] transcription factor activity | molecular\_function | [GO:0005488] binding | [GO:0003676] nucleic acid binding | [GO:0003677] DNA binding | ref|NP\_175627 | ATHB-15 [Arabidopsis thaliana] | SSR | mapped |  |
| SsrPt\_ctg946 | gb|CX714486 | Pinus taeda | RTPQ1\_22\_F12\_A032 | Pta.11295 | [GO:0003674] molecular\_function | molecular\_function | [Lineage ends at prior level] | [Lineage ends at prior level] | [Lineage ends at prior level] | n/a | n/a | SSR | mapped |  |

Compiled by C. Echt et al., January 2011, USDA Forest Service.
